# Supplementary material for: Pervasive epistasis exposes intramolecular networks in adaptive enzyme evolution
Source: Nat Commun. 2023 Dec 21;14:8508. doi: 10.1038/s41467-023-44333-5 (PMC10739712; doi:10.1038/s41467-023-44333-5)
Supplement: Supplementary file 1 — Supplementary Information [file 41467_2023_44333_MOESM1_ESM.docx]

**Supplementary Information**

**
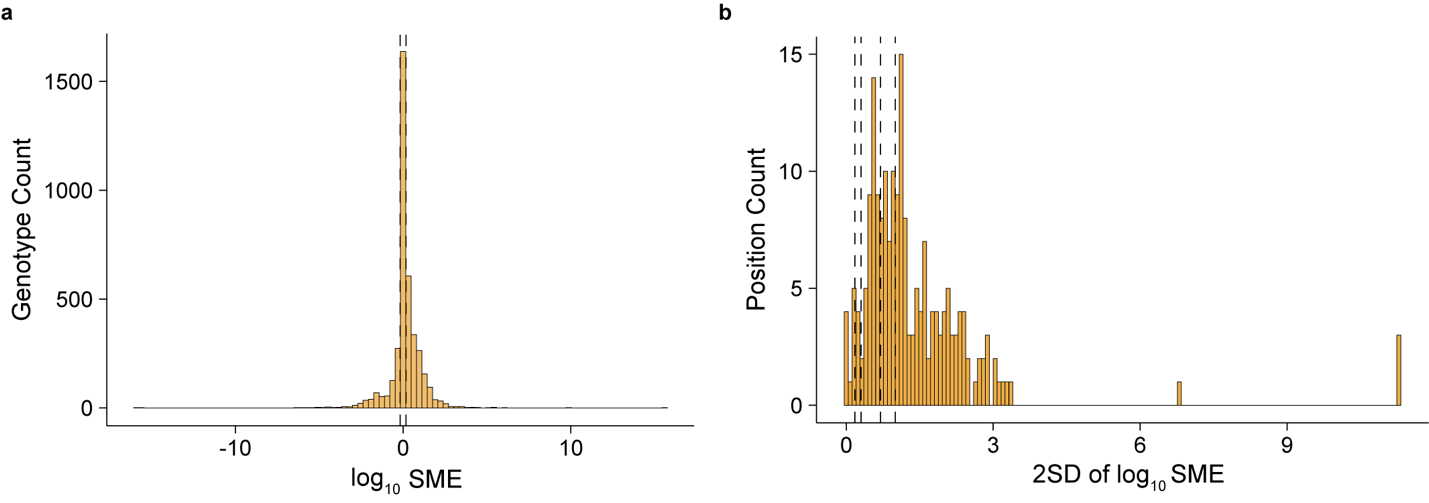
**

**Supplementary Fig. 1 | Expanded histograms from Figs. 2a and 3b. a,** Distribution of single mutational effects (log_10_ SME) at every position across all genotypes for every combinatorial landscape. Dashed lines represent the 1.5-fold significance threshold that distinguishes negative, neutral, and positive effects, respectively. **b,** Distribution of log_10_ 2 SD of single mutational effects (SMEs) for all mutational positions, with annotated dashed lines representing 1.5-fold, 2-fold, 5-fold, and 10-fold significance thresholds, respectively.


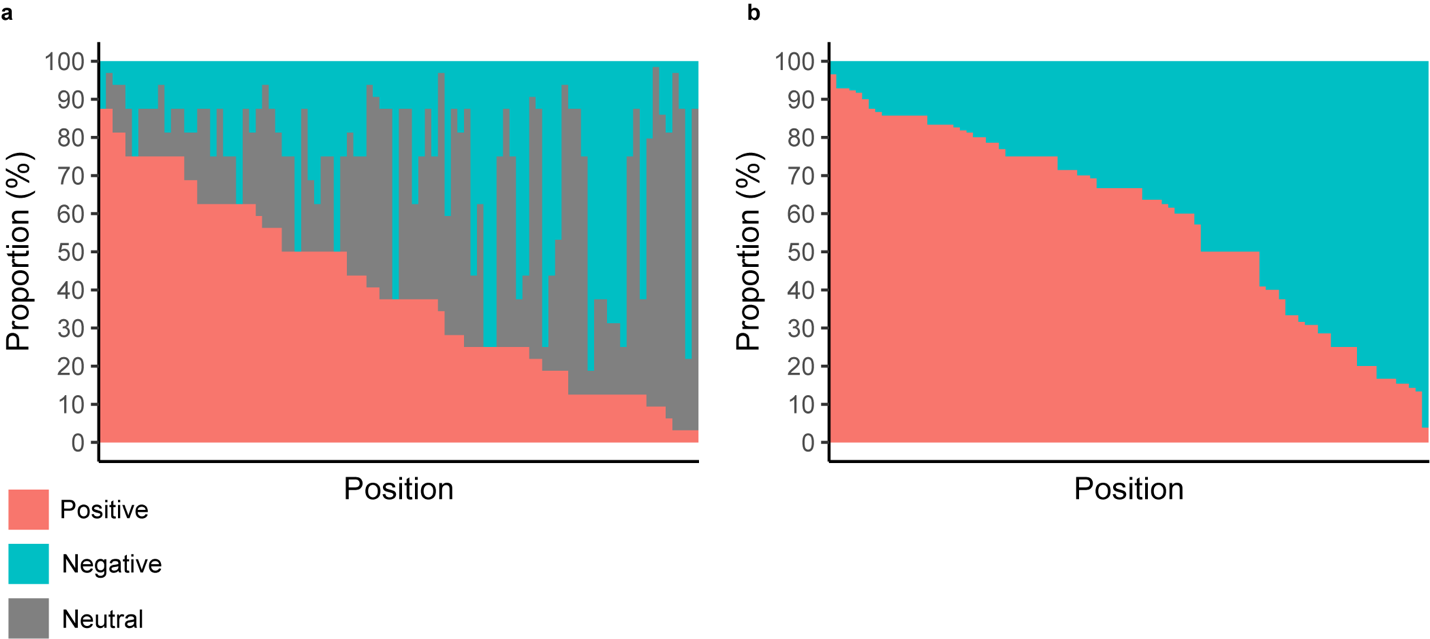


**Supplementary Fig. 2 | Proportions of sign distributions for all positions in the positive-negative category. a,** Sorted in descending order for positive SMEs, including positive, negative, and neutral proportions for each position. **b,** Sorted in descending order for positive SMEs wherein neutral SMEs were eliminated from all positions in the positive-negative category.


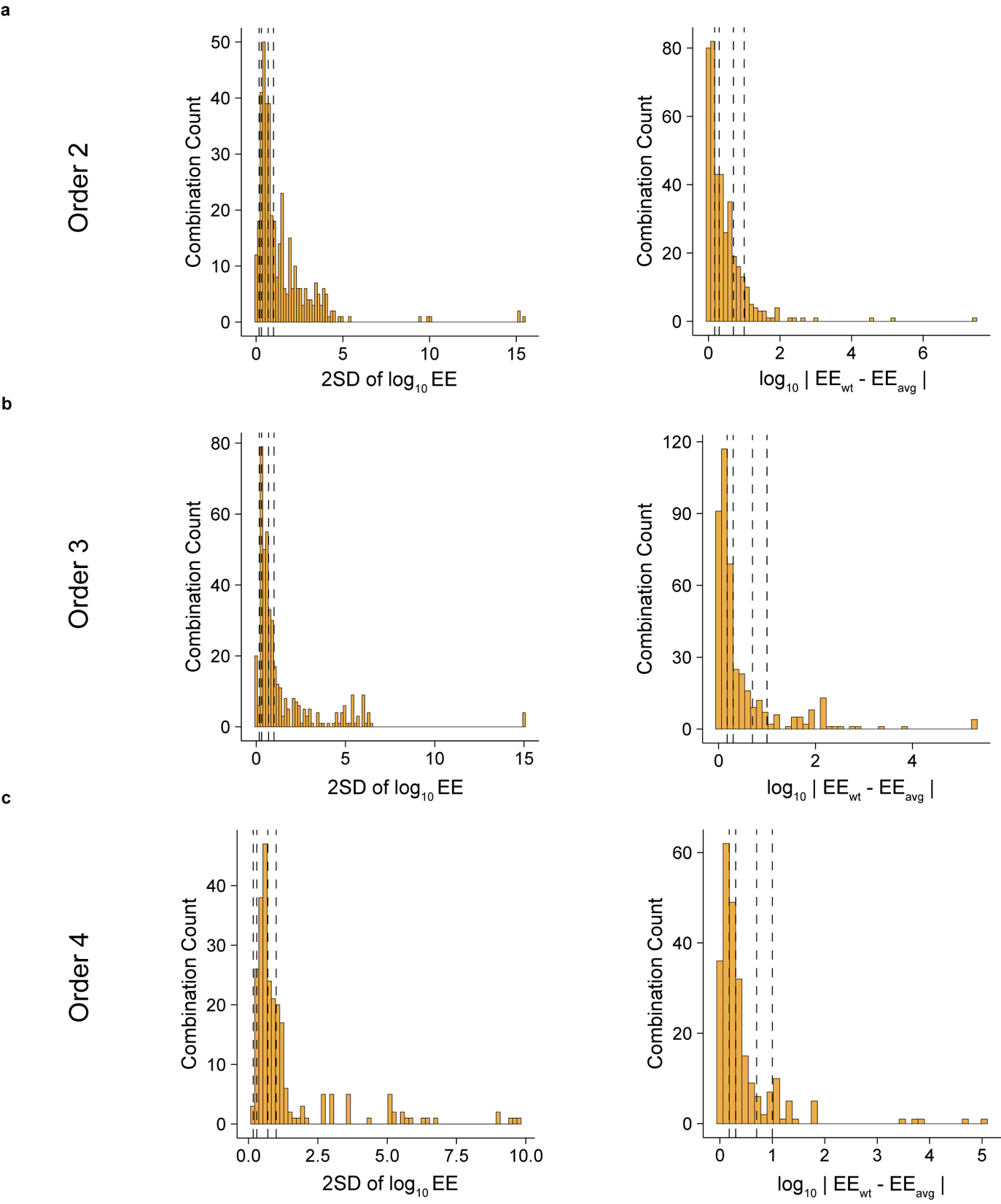


**Supplementary Fig. 3 | Expanded histograms from Figs. 3a and 3c.** Distribution of log_10_ 2 SD of epistatic effects (EEs) at each order for all mutational combinations, with annotated dashed lines representing 1.5-fold, 2-fold, 5-fold, and 10-fold significance thresholds, respectively as well as distribution of the absolute difference between wt EE and avg EE at each combination, with annotated dashed lines represent 1.5-, 2-, 5-, and 10-fold significance thresholds, respectively, for **a**, order 2, **b,** order 3, and **c,** order 4.


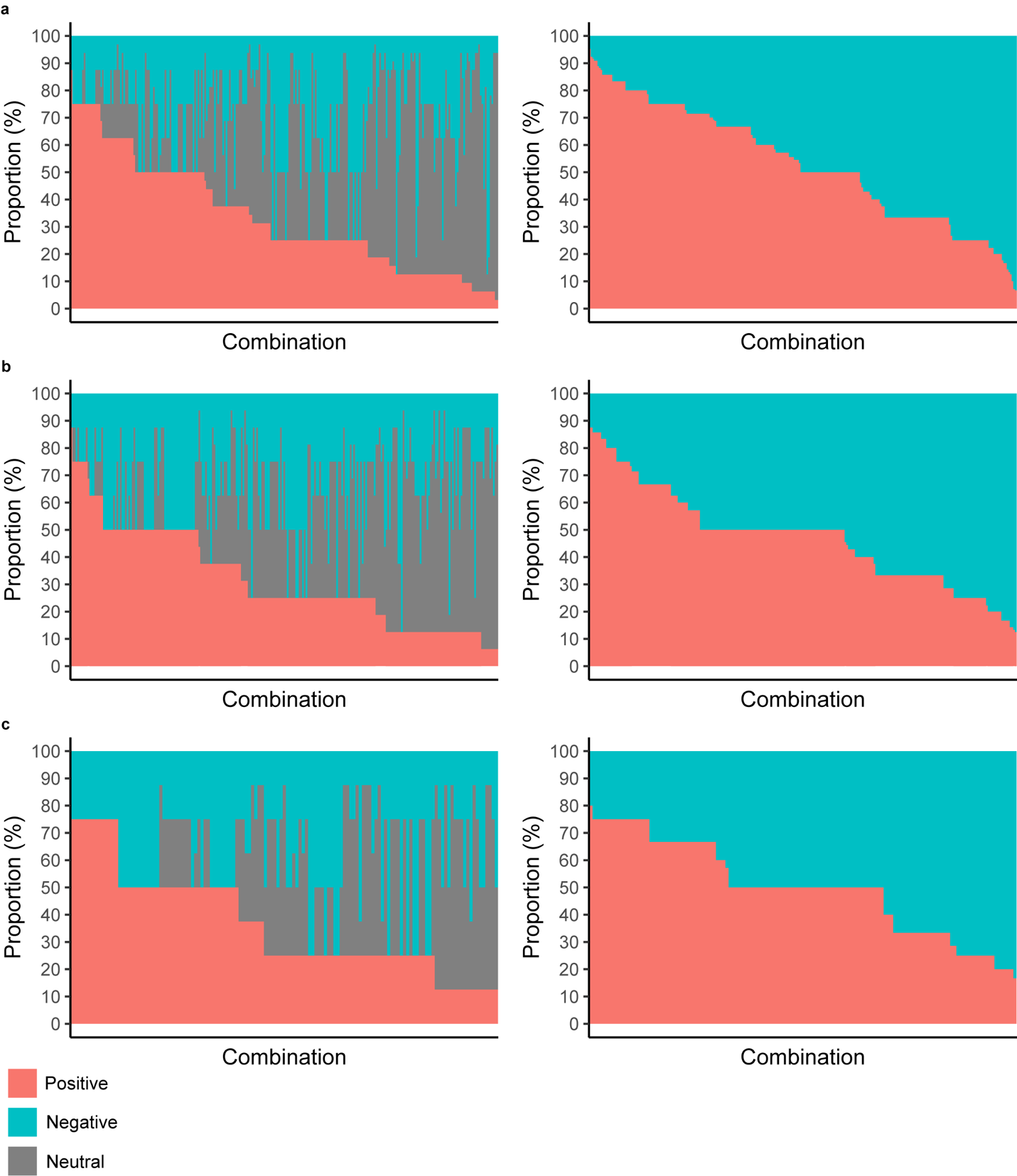


**Supplementary Fig. 4 | Proportions of sign distributions for all combinations in the positive-negative category across all orders.** Proportions sorted in descending order for positive EEs, including positive, negative, and neutral as well as proportions with neutral EEs removed for **a,** pairwise combinations, **b,** three-way combinations and **c,** four-way combinations.


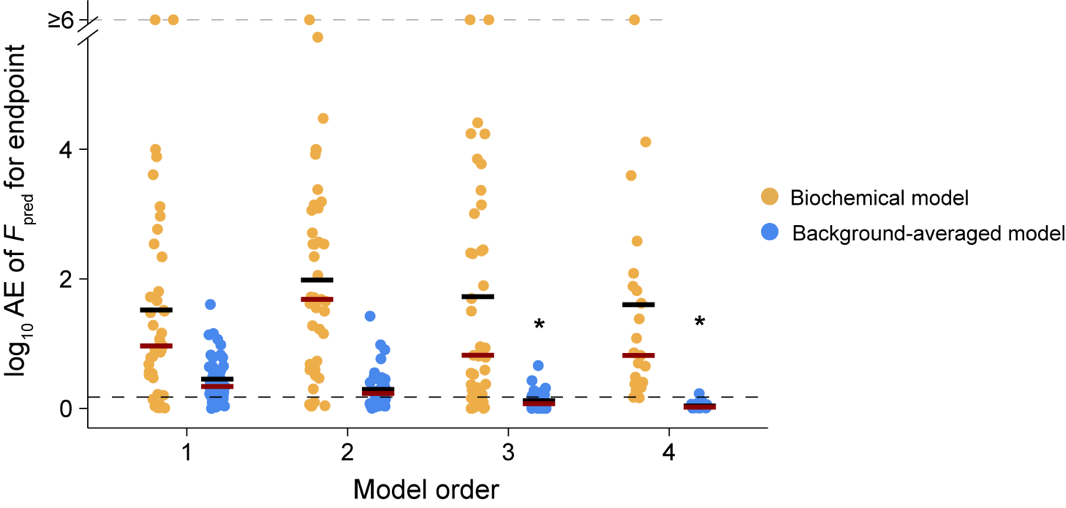


**Supplementary Fig. 5 | Apparent idiosyncrasy distorts endpoint functional predictions**. The log_10_ of the absolute error (AE) of the predicted function (*F*_pred_) at the 1^st^, 2^nd^ and 3^rd^ orders (n=45), and the 4^th^ one (n=23). The means and medians of the absolute error from the biochemical (yellow) and background-averaged (blue) are shown as black and red bars, respectively. The 1.5-fold significance threshold is depicted as a dashed line, with an asterisk (*) marking the model and order with a significant mean (one-sided t-test; p = 9.67 x 10^-7^ and 1.83 x 10^-12^ for background-averaged model order 3 and 4, respectively).


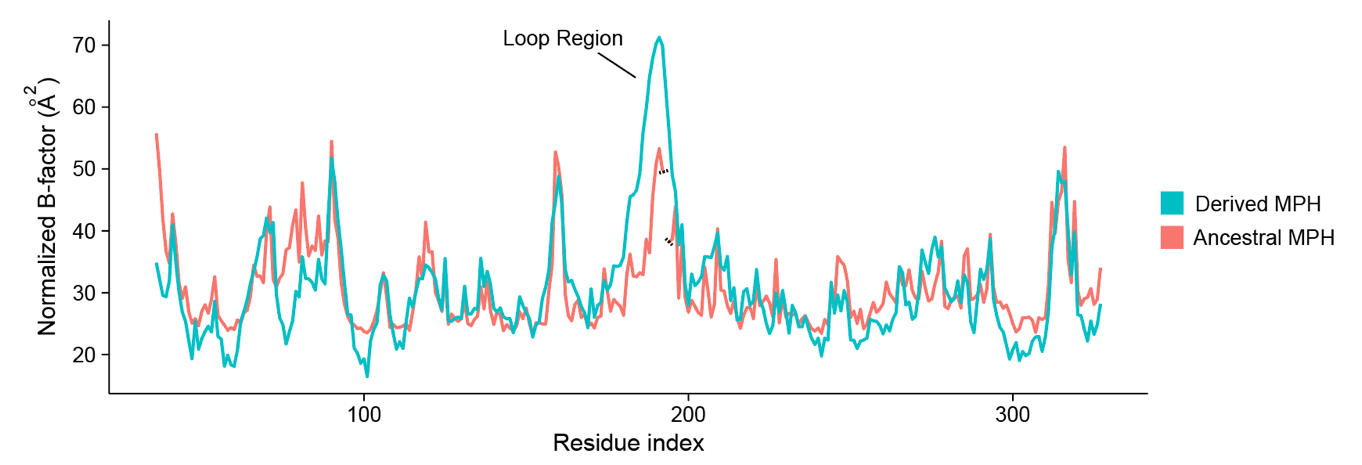


**Supplementary Fig. 6 | Evolution of novel function in MPH shows increased loop disorder.** A plot of normalized B-factor for MPH chain A shows increased disorder in the derived MPH (PDB: 1P9E) in the loop region (res. 181–201) compared to the ancestral MPH (PDB: 6C2C).

| **Supplementary Table 1 \| Percent of positions (1^st^ order) and combinations (2^nd^, 3^rd^, and 4^th^ order) that show a 2 SD for SMEs or EEs above the listed thresholds.** | | | | |
| --- | --- | --- | --- | --- |
|  | Threshold | | | |
| Order | 1.5-fold | 2-fold | 5-fold | 10-fold |
| 1 | 95.5% (189/198) | 92.9% (184/198) | 71.2% (141/198) | 55.6% (110/198) |
| 2 | 95.2% (376/395) | 88.9% (351/395) | 58.0% (229/395) | 43.5% (172/395) |
| 3 | 94.1% (397/422) | 85.1% (359/422) | 48.8% (206/422) | 34.8% (147/422) |
| 4 | 99.6% (244/245*) | 91.8% (225/245*) | 50.2% (123/245*) | 32.2% (79/245*) |
| ***** 4^th^ order considers 245 out of the total 263 combinations as some landscapes have a single 4^th^ order data point and a 2 SD cannot be computed | | | | |

| **Supplementary Table 2 \| Percent of positions (1^st^ order) and combinations (2^nd^, 3^rd^, and 4^th^ order) that show a 2 SD for SMEs or EEs above the listed thresholds in the reduced landscape dataset.** | | | | |
| --- | --- | --- | --- | --- |
|  | Threshold | | | |
| Order | 1.5-fold | 2-fold | 5-fold | 10-fold |
| 1 | 89.5% (51/57) | 84.2% (48/57) | 66.7% (38/57) | 45.6% (26/57) |
| 2 | 91.9% (114/124) | 75.8% (94/124) | 45.2% (56/124) | 35.5% (44/124) |
| 3 | 91.8% (135/147) | 76.2% (112/147) | 34.7% (51/147) | 25.2% (37/147) |
| 4 | 99.0% (99/100*) | 85.0% (85/100*) | 37.0% (37/100*) | 20.0% (20/100*) |
| ***** 4^th^ order considers 100 out of the total 102 combinations as some landscapes have a single 4^th^ order data point and a 2 SD cannot be computed | | | | |

| **Supplementary Table 3 \| Sign variation of the positions’ (1^st^ order) and combinations’ (2^nd^, 3^rd^, and 4^th^ orders) SMEs and EEs.** | | | | | | |
| --- | --- | --- | --- | --- | --- | --- |
| Order | Negative | Neutral | Positive | Negative-Neutral | Neutral-Positive | Negative-Positive |
| 1 | 1.5% (3/198) | 2.5% (5/198) | 10.1% (20/198) | 17.2% (34/198) | 22.2% (44/198) | 46.5% (92/198) |
| 2 | 0.8% (3/395) | 2.8% (11/395) | 2.5% (10/395) | 11.1% (44/395) | 17.2% (68/395) | 65.6% (259/395) |
| 3 | 2.8% (12/422) | 3.1% (13/422) | 8.8% (37/422) | 12.1% (51/422) | 13.8% (58/422) | 59.5% (251/422) |
| 4 | 5.7% (14/245*) | 1.2% (3/245*) | 3.3% (8/245*) | 19.2% (47/245*) | 15.5% (38/245*) | 55.1% (135/245*) |

| **Supplementary Table 4 \| Sign variation of the positions’ (1^st^ order) and combinations’ (2^nd^, 3^rd^, and 4^th^ orders) SMEs and EEs in the reduced landscape dataset.** | | | | | | |
| --- | --- | --- | --- | --- | --- | --- |
| Order | Negative | Neutral | Positive | Negative-Neutral | Neutral-Positive | Negative-Positive |
| 1 | 3.5% (2/57) | 3.5% (2/57) | 14.0% (8/57) | 15.8% (9/57) | 40.4% (23/57) | 22.8% (13/57) |
| 2 | 0.0% (0/124) | 2.4% (3/124) | 4.0% (5/124) | 16.9% (21/124) | 23.4% (29/124) | 53.2% (66/124) |
| 3 | 4.8% (7/147) | 2.7% (4/147) | 4.8% (7/147) | 19.0% (28/147) | 18.4% (27/147) | 50.3% (74/147) |
| 4 | 7.0% (7/100*) | 1.0% (1/100*) | 5.0% (5/100*) | 18.0% (18/100*) | 20.0% (20/100*) | 49.0% (49/100*) |

| **Supplementary Table 5 \| Percent of positions and combinations that show a deviation between wt SME and avg SME or wt EE and avg EE above the listed thresholds.** | | | | |
| --- | --- | --- | --- | --- |
|  | Threshold | | | |
| Order | 1.5-fold | 2-fold | 5-fold | 10-fold |
| 1 | 66.7% (132/198) | 52.5% (104/198) | 24.2% (48/198) | 11.6% (23/198) |
| 2 | 59.5% (235/395) | 47.8% (189/395) | 20.5% (81/395) | 9.6% (38/395) |
| 3 | 51.7% (218/422) | 34.4% (145/422) | 19.0% (80/422) | 12.6% (53/422) |
| 4 | 60.4% (148/245*) | 40.0% (98/245*) | 16.3% (40/245*) | 11.0% (27/245*) |

| **Supplementary Table 6 \| Percent of positions and combinations that show a deviation beteween wt SME and avg SME or wt EE and avg EE above the listed thresholds in the reduced landscape dataset.** | | | | |
| --- | --- | --- | --- | --- |
|  | Threshold | | | |
| Order | 1.5-fold | 2-fold | 5-fold | 10-fold |
| 1 | 70.2% (40/57) | 52.6% (30/57) | 26.3% (15/57) | 8.8% (5/57) |
| 2 | 46.8% (58/124) | 37.1% (46/124) | 12.9% (16/124) | 5.6% (7/124) |
| 3 | 38.1% (56/147) | 22.4% (33/147) | 5.4% (8/147) | 2.7% (4/147) |
| 4 | 51.0% (51/100*) | 35.0% (35/100*) | 15.0% (15/100*) | 7.0% (7/100*) |

| **Supplementary Table 7 \| Percent of SMEs and EEs that show a deviation from the average SME or EE at their position or combination, respectively, above the listed thresholds.** | | | | |
| --- | --- | --- | --- | --- |
|  | Threshold | | | |
| Order | 1.5-fold | 2-fold | 5-fold | 10-fold |
| 1 | 58.0% (2283/3936) | 41.5% (1635/3936) | 15.7% (619/3936) | 8.8% (348/3936) |
| 2 | 51.4% (2356/4576) | 35.3% (1615/4576) | 14.4% (659/4576) | 8.0% (368/4576) |
| 3 | 50.9% (1499/2944) | 33.8% (996/2944) | 13.5% (397/2944) | 8.9% (262/2944) |
| 4 | 60.2% (674/1120) | 40.6% (455/1120) | 11.9% (133/1120) | 7.6% (85/1120) |

| **Supplementary Table 8 \| Percent of SMEs and EEs that show a deviation from the average SME or EE at their position or combination, respectively, above the listed thresholds in the reduced landscape dataset.** | | | | |
| --- | --- | --- | --- | --- |
|  | Threshold | | | |
| Order | 1.5-fold | 2-fold | 5-fold | 10-fold |
| 1 | 54.0% (777/1440) | 37.7% (543/1440) | 10.7% (154/1440) | 4.9% (71/1440) |
| 2 | 43.6% (777/1784) | 28.1% (502/1784) | 8.2% (152/1784) | 3.7% (66/1784) |
| 3 | 41.7% (510/1224) | 24.6% (301/1224) | 6.9% (84/1224) | 3.3% (41/1224) |
| 4 | 49.0% (245/500) | 30.2% (151/500) | 8.0% (40/500) | 3.4% (17/500) |

| **Supplementary Table 9 \| Mean and median absolute error of the models’ prediction for the derived variant at each model order.** | | | | |
| --- | --- | --- | --- | --- |
| Model | 1^st^ Order | 2^nd^ Order | 3^rd^ Order | 4^th^ Order |
| Background-averaged | 2.8-fold \| 2.2-fold | 2.0-fold \| 1.7-fold | 1.3-fold \| 1.2-fold | 1.1-fold \| 1.0-fold |
| Biochemical | 33.0-fold \| 9.2-fold | 95.8-fold \| 48.3-fold | 53.3-fold \| 6.6-fold | 39.9-fold \| 6.6-fold |

| **Supplementary Table 10 \| Mean and median absolute error of the models’ prediction for the derived variant at each model order in the reduced landscape dataset.** | | | | |
| --- | --- | --- | --- | --- |
| Model | 1^st^ Order | 2^nd^ Order | 3^rd^ Order | 4^th^ Order |
| Background-averaged | 2.2-fold \| 1.8-fold | 1.8-fold \| 2.2-fold | 1.1-fold \| 1.1-fold | 1.1-fold \| 1.1-fold |
| Biochemical | 69.5-fold \| 14.6-fold | 64.4-fold \| 18.9-fold | 6.0-fold \| 2.1-fold | 15.9-fold \| 17.1-fold |

| **Supplementary Table 11 \| Biochemical model absolute error in predicted log-transformed function (log_10_ *F*) of each variant in the most accessible path by model order.** | | | | | | | |
| --- | --- | --- | --- | --- | --- | --- | --- |
|  |  | **Order** | | | | | |
| **Predicted Variant Mutations** | **Trajectory** | 1^st^ | 2^nd^ | 3^rd^ | 4^th^ | 5^th^ | 6^th^ |
| 2 | DHFR IC_75_ | ***0.03*** | - | - | - | - | - |
| 3 |  | 0.46 | ***0.12*** | - | - | - | - |
| 2 | DHFR G-Traj. | **0.73** | - | - | - | - | - |
| 3 |  | 1.58 | ***0.13*** | - | - | - | - |
| 4 |  | 2.72 | 1.46 | ***0.05*** | - | - | - |
| 5 |  | 3.89 | 2.34 | 0.82 | **0.32** | - | - |
| 2 | DHFR R-Traj | **0.81** | - | - | - | - | - |
| 3 |  | 1.68 | ***0.09*** | - | - | - | - |
| 4 |  | 2.40 | 0.66 | **0.54** | - | - | - |
| 5 |  | 2.97 | 2.57 | ***0.16*** | 0.27 | - | - |
| 2 | MPH Zn PTM | ***0.06*** | - | - | - | - | - |
| 3 |  | 0.69 | ***0.00*** | - | - | - | - |
| 4 |  | 0.55 | 1.90 | **0.23** | - | - | - |
| 5 |  | 0.88 | 3.38 | 0.85 | **0.70** | - | - |
| 2 | NfsA 20_39 | **0.19** | - | - | - | - | - |
| 3 |  | 0.21 | **0.16** | - | - | - | - |
| 4 |  | 0.21 | 0.23 | ***0.08*** | - | - | - |
| 5 |  | **0.22** | 0.45 | 0.57 | 1.07 | - | - |
| 2 | NfsA 36_37 | **0.25** | - | - | - | - | - |
| 3 |  | 0.41 | ***0.17*** | - | - | - | - |
| 4 |  | 0.47 | ***0.06*** | *0.14* | - | - | - |
| 5 |  | 0.54 | ***0.01*** | 0.13 | 0.40 | - | - |
| 6 |  | 0.56 | 0.32 | 0.26 | 0.43 | ***0.04*** | - |
| 7 |  | 0.57 | ***0.09*** | 0.16 | 0.16 | 1.54 | 1.23 |
| 2 | OXA-48 CAZ Traj. 1 | **1.17** | - | - | - | - | - |
| 3 |  | 1.49 | **0.71** | - | - | - | - |
| 4 |  | 1.52 | 0.68 | **0.22** | - | - | - |
| 2 | OXA-48 CAZ Traj. 2 | **0.19** | - | - | - | - | - |
| 3 |  | 0.47 | **0.41** | - | - | - | - |
| 4 |  | 0.92 | 0.64 | **0.20** | - | - | - |
| 5 |  | 0.95 | 0.73 | 0.52 | ***0.01*** | - | - |
| 6 |  | 0.90 | 0.51 | 0.59 | 0.39 | ***0.05*** | - |
| 2 | OXA-48 CAZ Traj. 3 | **0.98** | - | - | - | - | - |
| 3 |  | 1.21 | **0.11** | - | - | - | - |
| 4 |  | 1.22 | 0.18 | ***0.05*** | - | - | - |
| 2 | PTE 2NH | **0.33** | - | - | - | - | - |
| 3 |  | **0.60** | 1.64 | - | - | - | - |
| 4 |  | 0.81 | 1.44 | **0.69** | - | - | - |
| 2 | TEM MIC | **2.98** | - | - | - | - | - |
| 3 |  | 3.77 | **1.10** | - | - | - | - |
| 4 |  | 3.90 | 3.22 | **0.80** | - | - | - |
| 5 |  | 4.00 | 3.19 | **1.70** | 1.89 | - | - |

**Supplementary Table 12 | Proportions for all patterns of change in EE_wt_ in the reduced landscape dataset**

| Pattern | | Rewiring | |
| --- | --- | --- | --- |
| No epistasis | 17.1% (55/321) |  |  |
| No change in epistasis | 23.4% (50/321) |  |  |
| New epistasis | 24.9% (75/321) |  |  |
| Change in epistasis | 43.9% (141/321) | Constructive | 29.1% (41/141) |
|  |  | Disruptive | 70.9% (100/141) |
