# Supplementary material for: Pervasive epistasis exposes intramolecular networks in adaptive enzyme evolution
Source: Nat Commun. 2023 Dec 21;14:8508. doi: 10.1038/s41467-023-44333-5 (PMC10739712; doi:10.1038/s41467-023-44333-5)
Supplement: Supplementary file 3 — Description of Additional Supplementary Files [file 41467_2023_44333_MOESM3_ESM.pdf]

Description of Additional Supplementary Files

File Name: Supplementary Data 1
Description: The graphical representation of genotype-phenotype maps for all fitness landscapes.

File Name: Supplementary Data 2
Description: Pooled data from log10 transformation and wt-normalization of enzyme functional data.

File Name: Supplementary Data 3
Description: The evaluation of two non-linear transformations on the comprehensive dataset.

File Name: Supplementary Data 4
Description: The comprehensive single mutational effect dataset for all analyzed fitness landscapes.

File Name: Supplementary Data 5
Description: Box plot representation of all positional and combinatorial distributions of single mutational and epistatic effects.

File Name: Supplementary Data 6
Description: The comprehensive epistatic effect dataset for all analyzed fitness landscapes.
