# Supplementary material for: Pervasive epistasis exposes intramolecular networks in adaptive enzyme evolution
Source: Nat Commun. 2023 Dec 21;14:8508. doi: 10.1038/s41467-023-44333-5 (PMC10739712; doi:10.1038/s41467-023-44333-5)
Supplement: Supplementary file 4 — Supplementary Data 1 [file 41467_2023_44333_MOESM4_ESM.pdf]

Supplementary Data 1

Representations of 41 fitness landscapes used in the study. Landscapes depict fold-change in activity from their WT background. The Mira *et al.* TEM landscapes depict $10^{\mu 1 - \mu 2}$, where μ1 is the genotype's growth rate and μ2 is the WT growth rate.

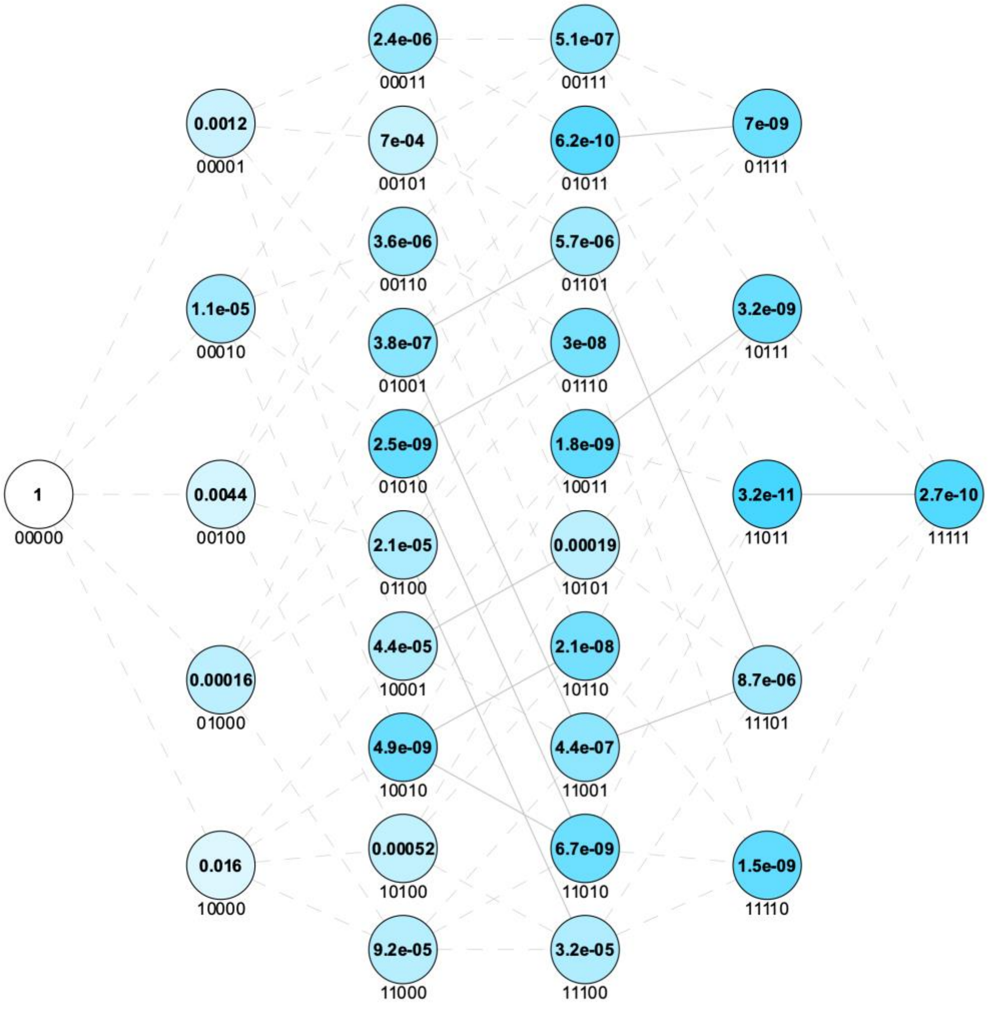

Landscape 1 | Fitness Landscape for Alkaline Phosphatase (AP) from Sunden *et al.*

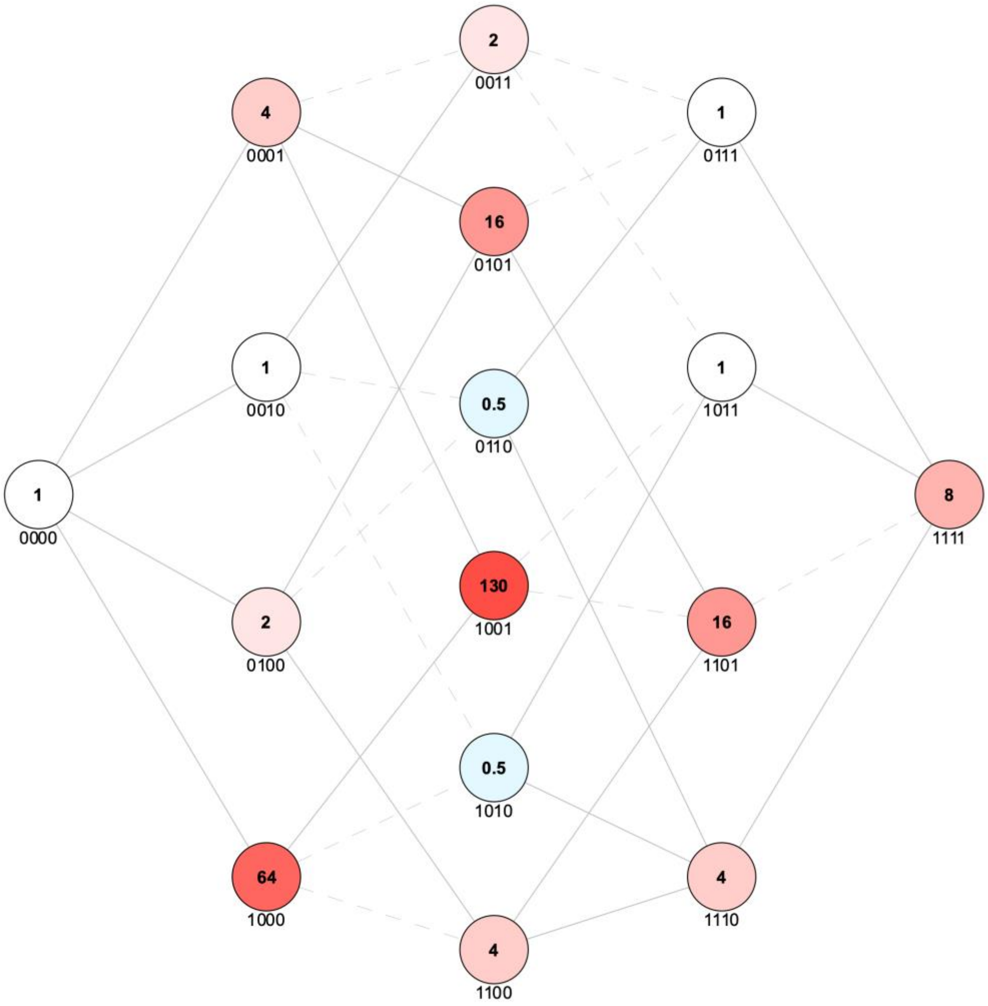

Landscape 2 | Fitness Landscape of Dihydrofolate Reductase (DHFR) for inhibitor c57 from Lozovsky *et al.*

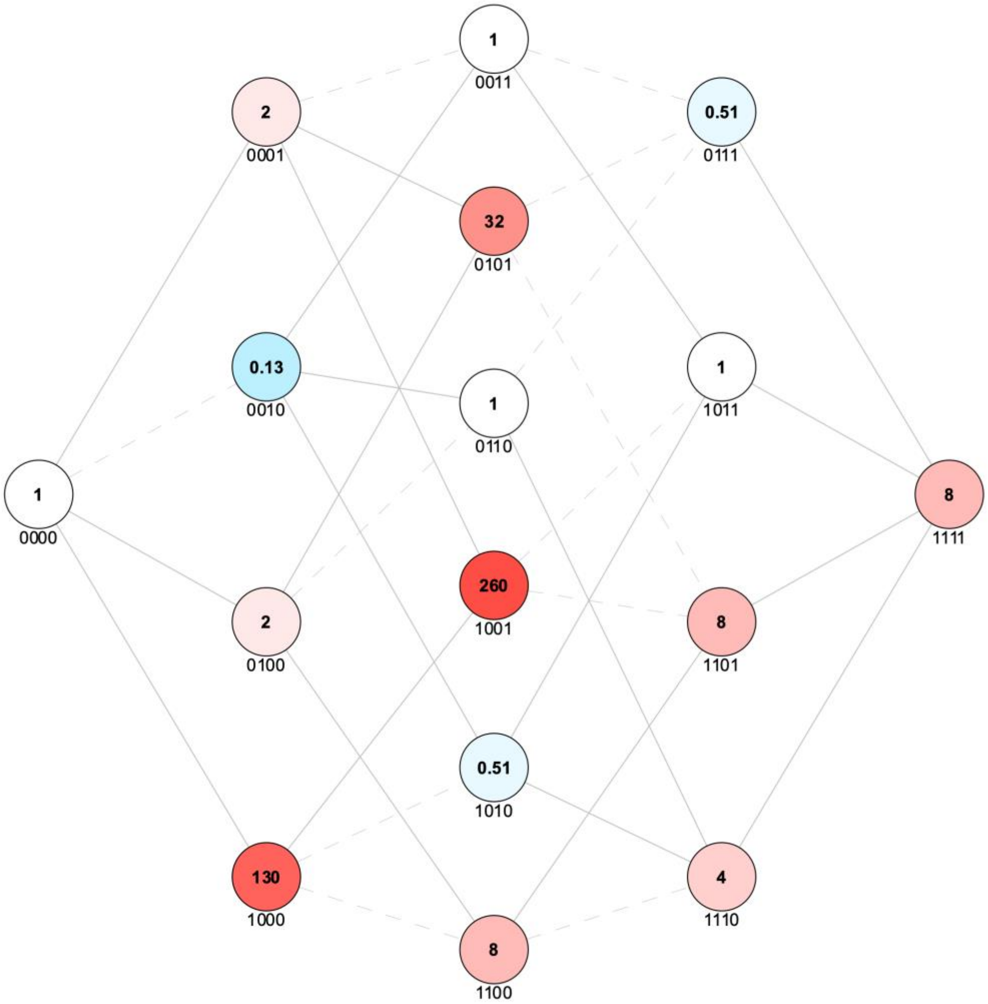

Landscape 3 | Fitness Landscape of Dihydrofolate Reductase (DHFR) for inhibitor c58 from Lozovsky *et al.*

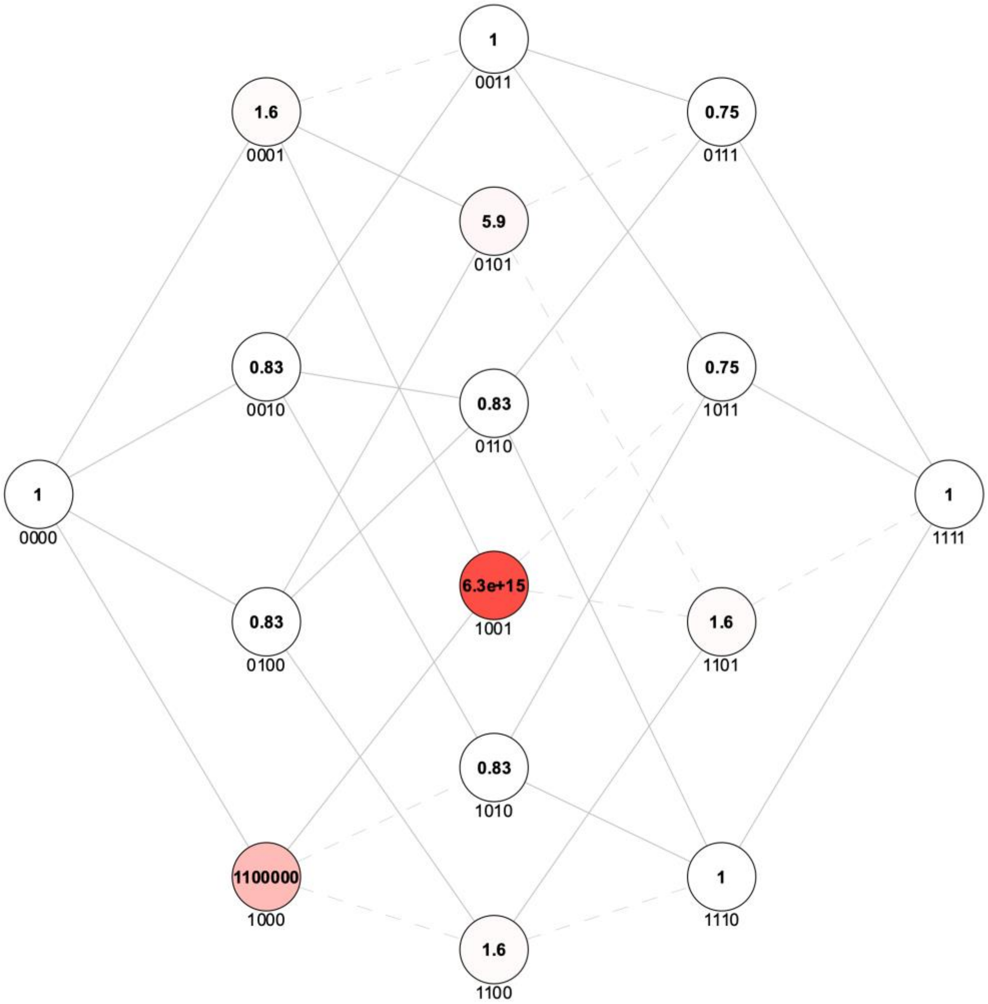

Landscape 4 | Fitness Landscape of Dihydrofolate Reductase (DHFR) for inhibitor c59 from Lozovsky *et al.*

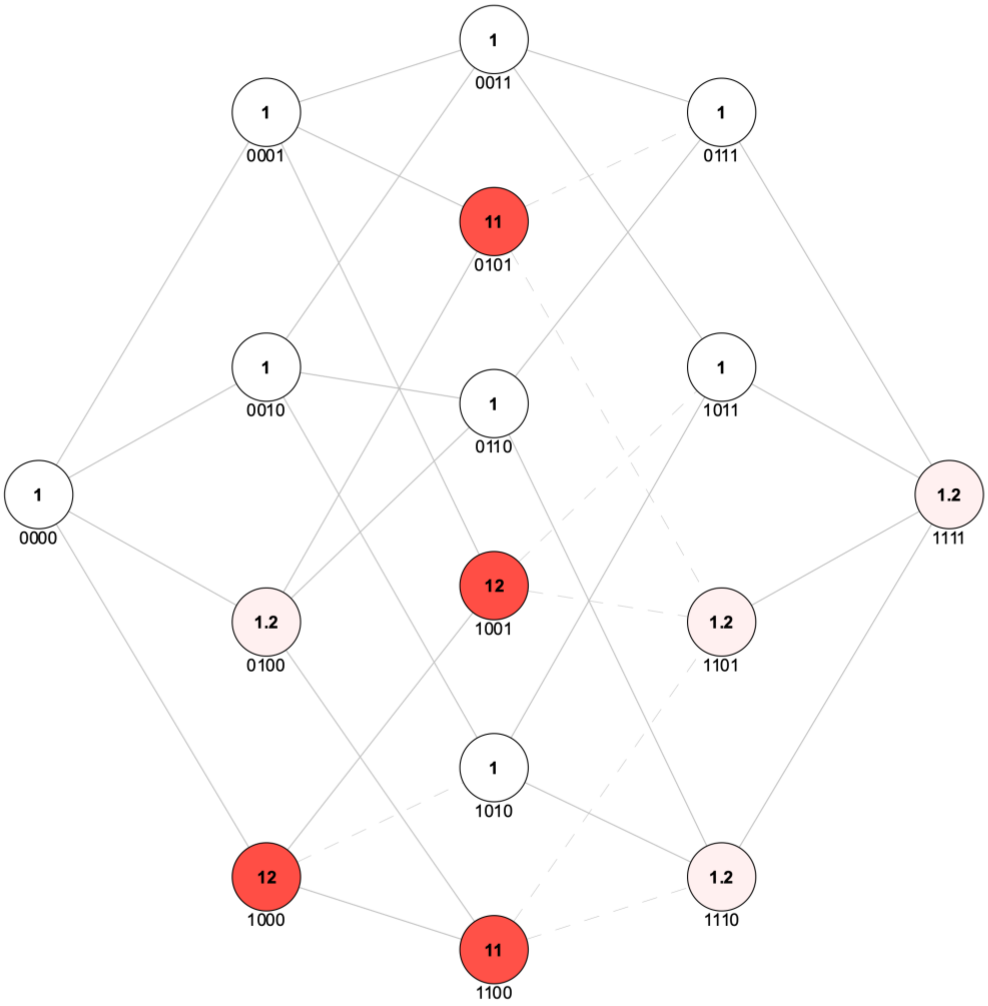

Landscape 5 | Fitness Landscape of Dihydrofolate Reductase (DHFR) for inhibitor c60 from Lozovsky *et al.*

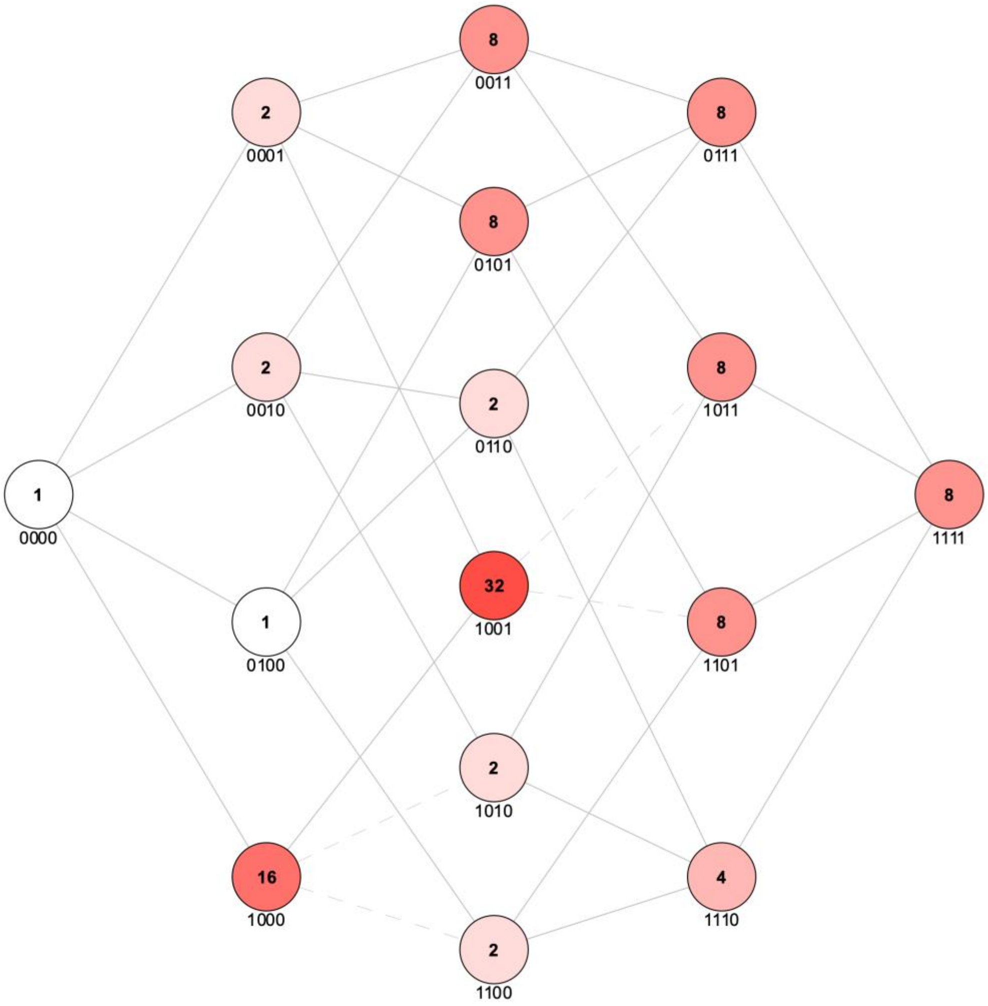

Landscape 6 | Fitness Landscape of Dihydrofolate Reductase (DHFR) for inhibitor c61 from

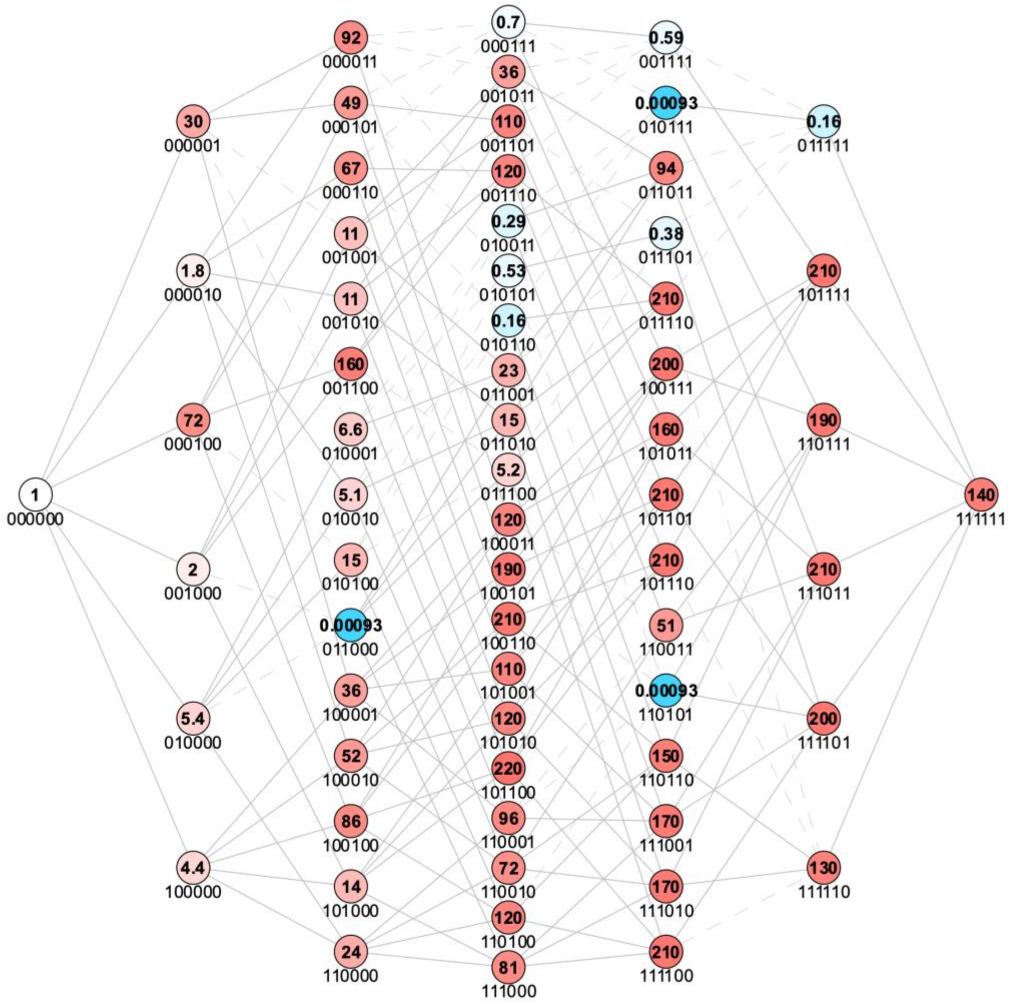

Landscape 7 | Fitness Landscape of Dihydrofolate Reductase (DHFR) from Palmer *et al.*

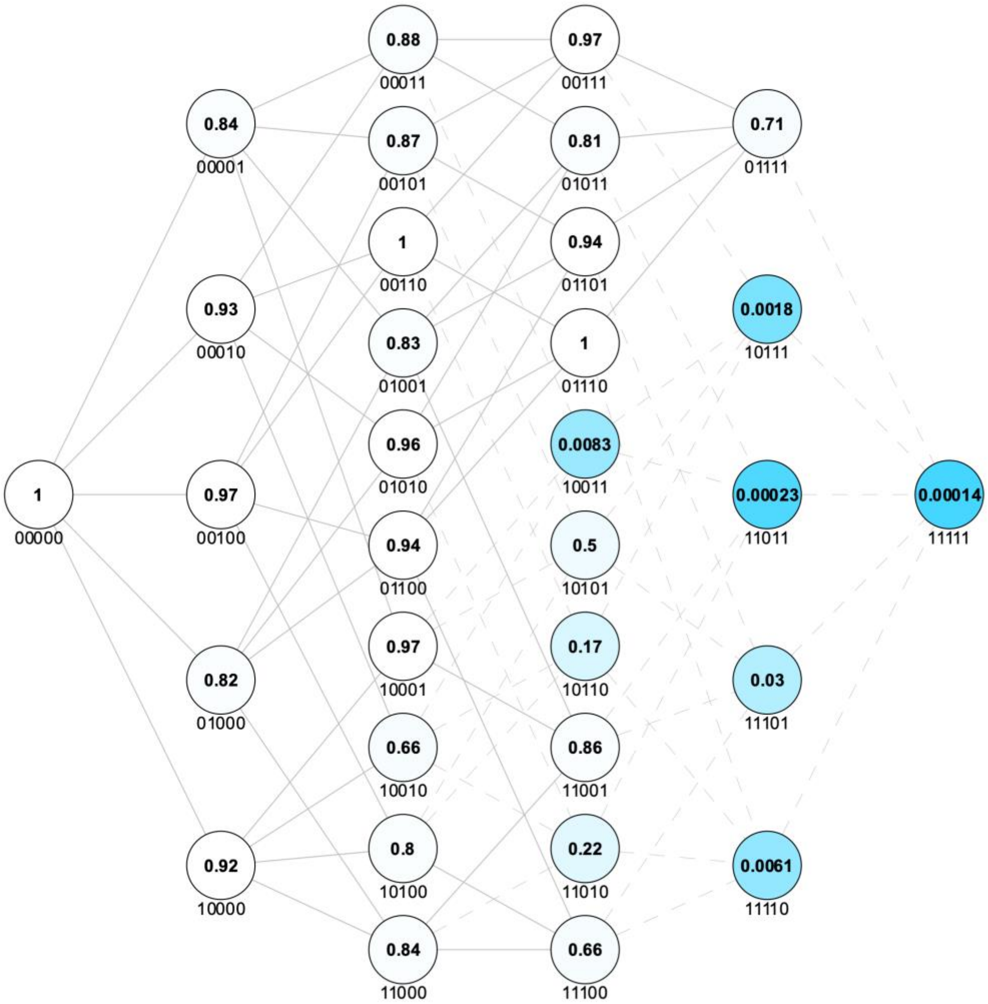

Landscape 8 | Fitness Landscape of Dihydrofolate Reductase (DHFR) of k_{cat} in the glycine trajectory from Tamer *et al.*

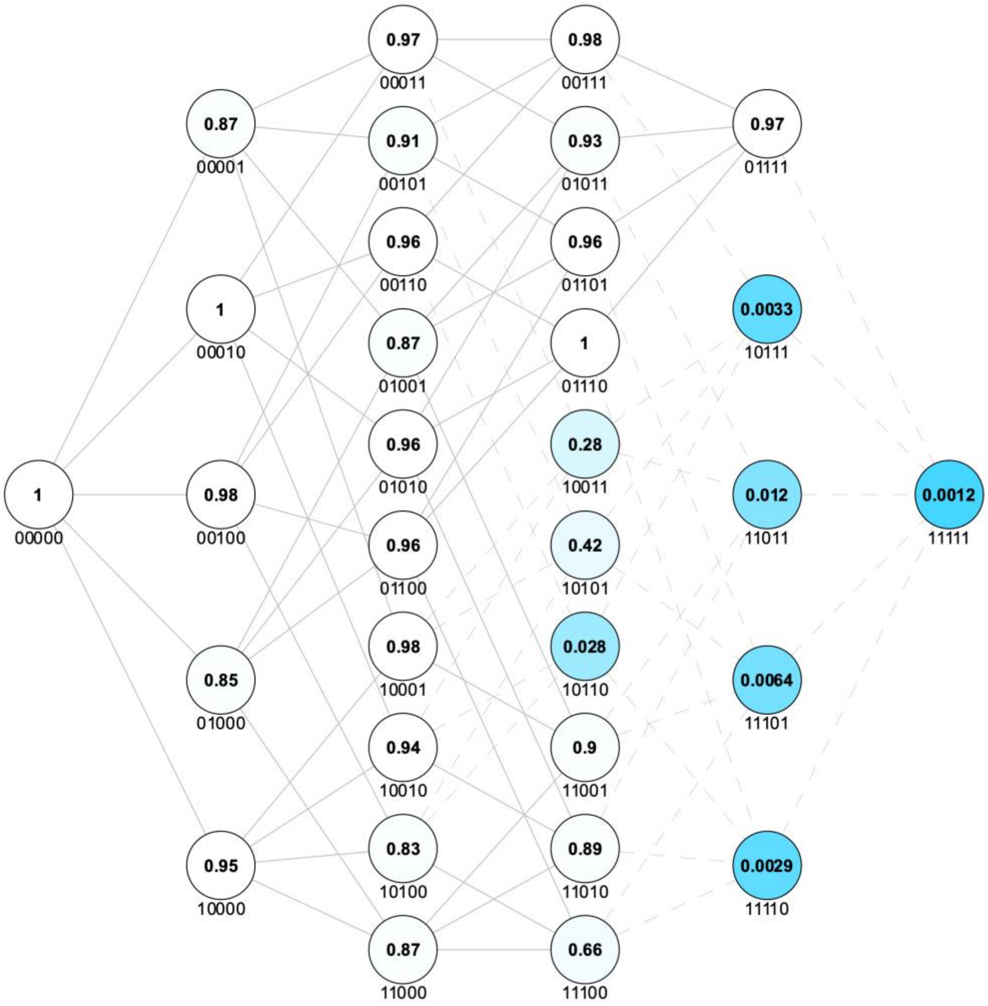

Landscape 9 | Fitness Landscape of Dihydrofolate Reductase (DHFR) of k_{cat} in the arginine trajectory from Tamer *et al.*

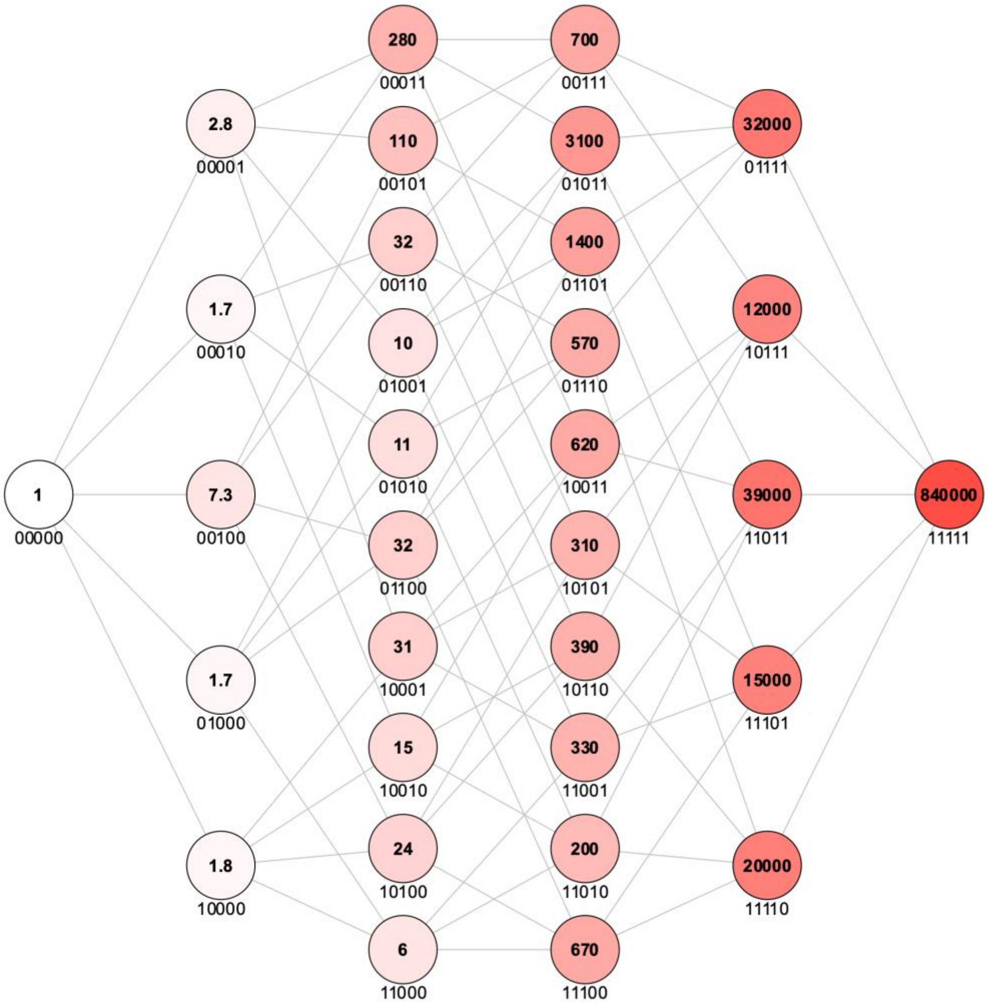

Landscape 10 | Fitness Landscape of Dihydrofolate Reductase (DHFR) of K_I in the glycine trajectory from Tamer *et al.*

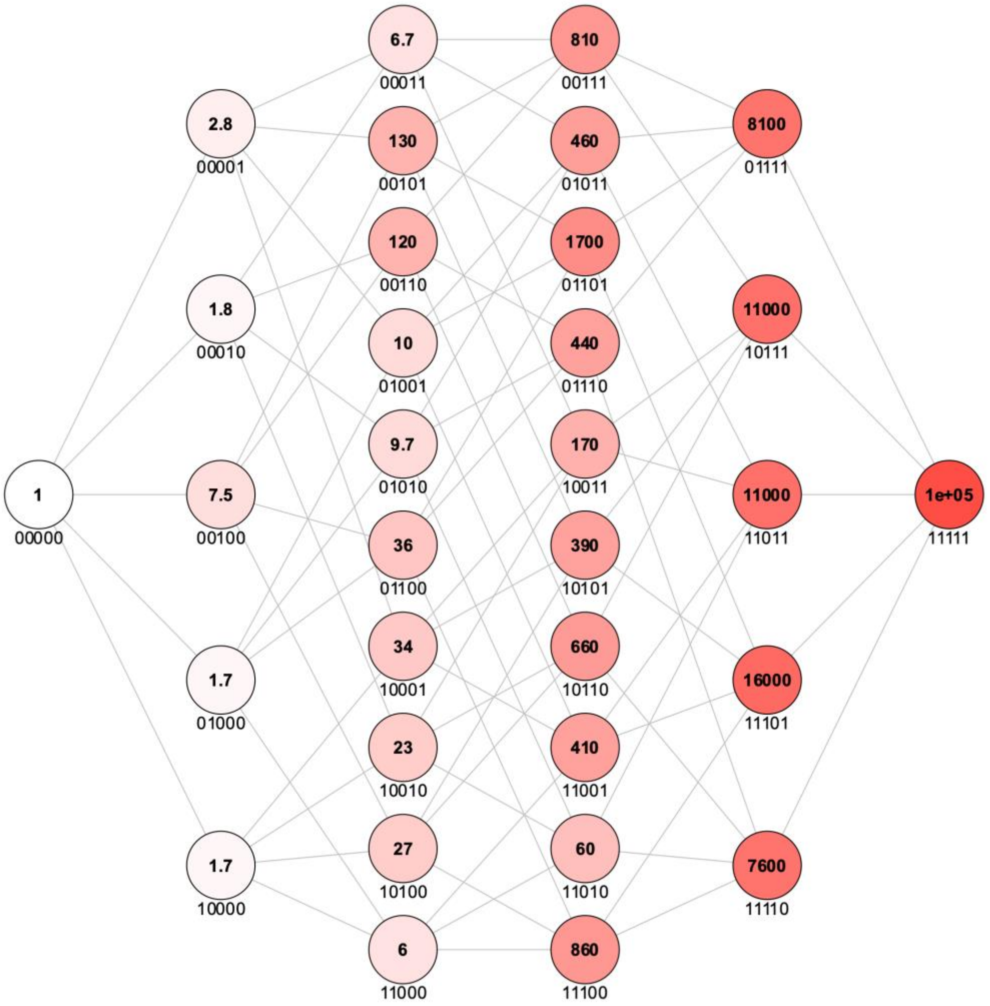

Landscape 11 | Fitness Landscape of Dihydrofolate Reductase (DHFR) of K_I in the arginine trajectory from Tamer *et al.*

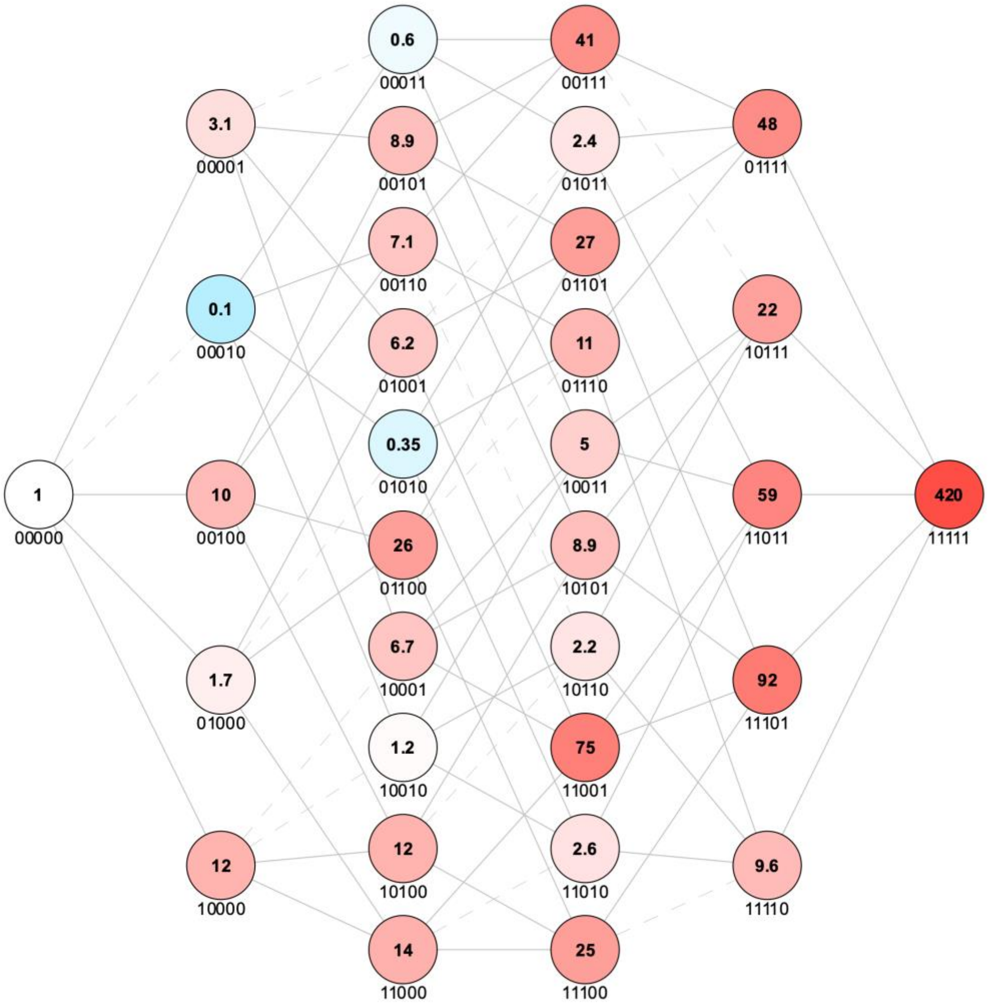

Landscape 12 | Fitness Landscape of Methyl Parathion Hydrolase (MPH) for methyl parathion in calcium metal conditions from Anderson *et al.*

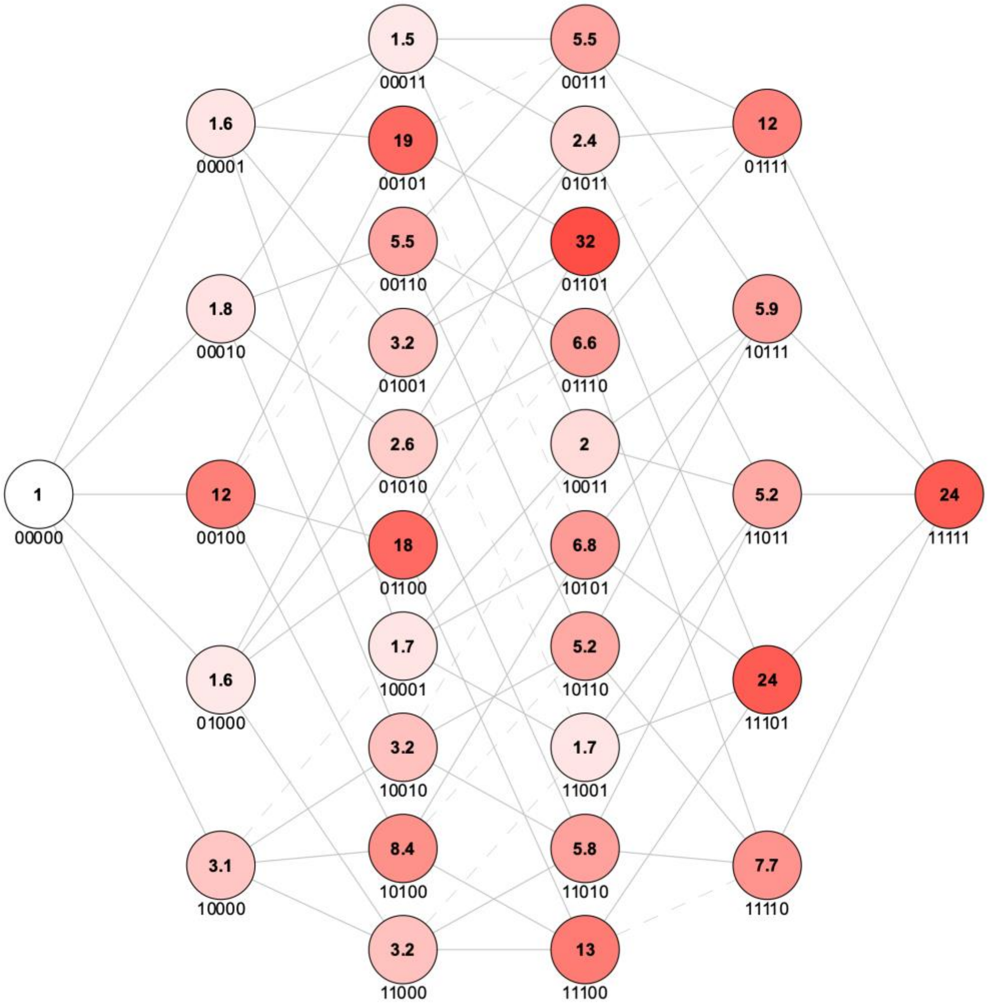

Landscape 13 | Fitness Landscape of Methyl Parathion Hydrolase (MPH) for methyl parathion in cadmium metal conditions from Anderson *et al.*

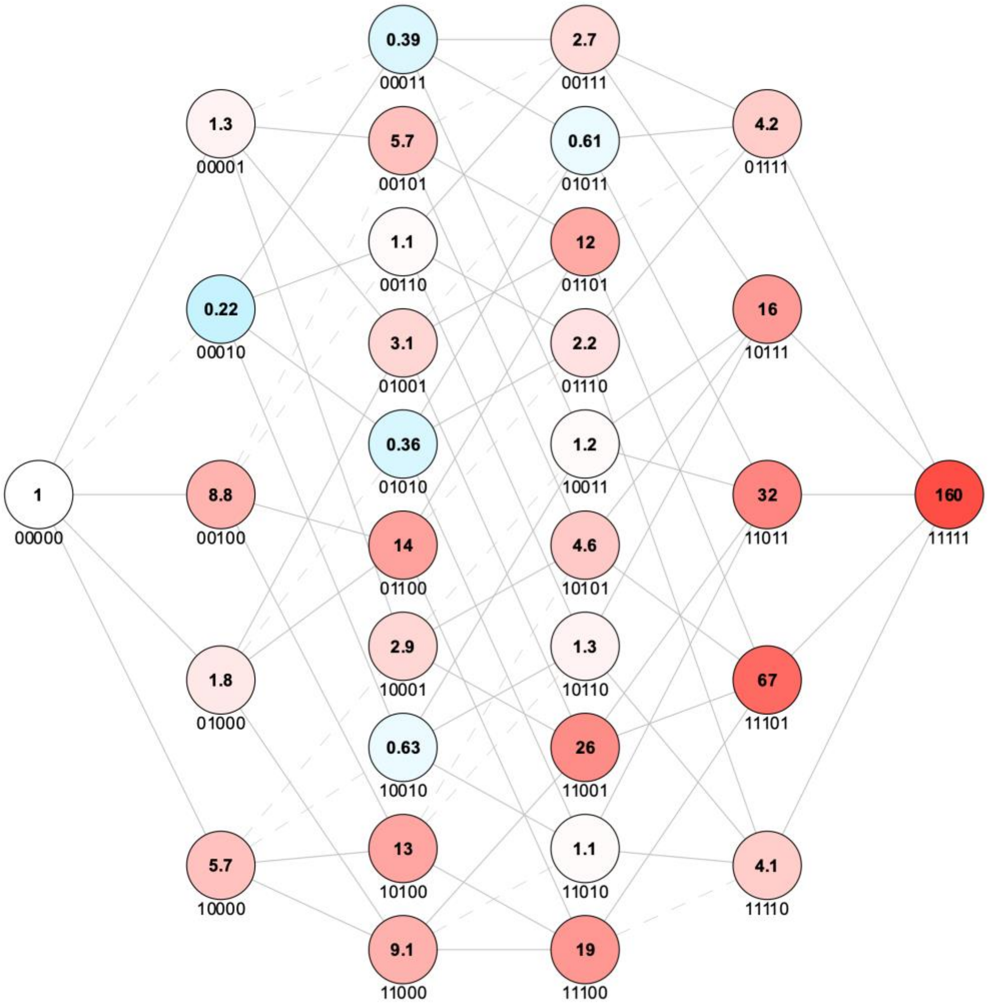

Landscape 14 | Fitness Landscape of Methyl Parathion Hydrolase (MPH) for methyl parathion in cobalt metal conditions from Anderson *et al.*

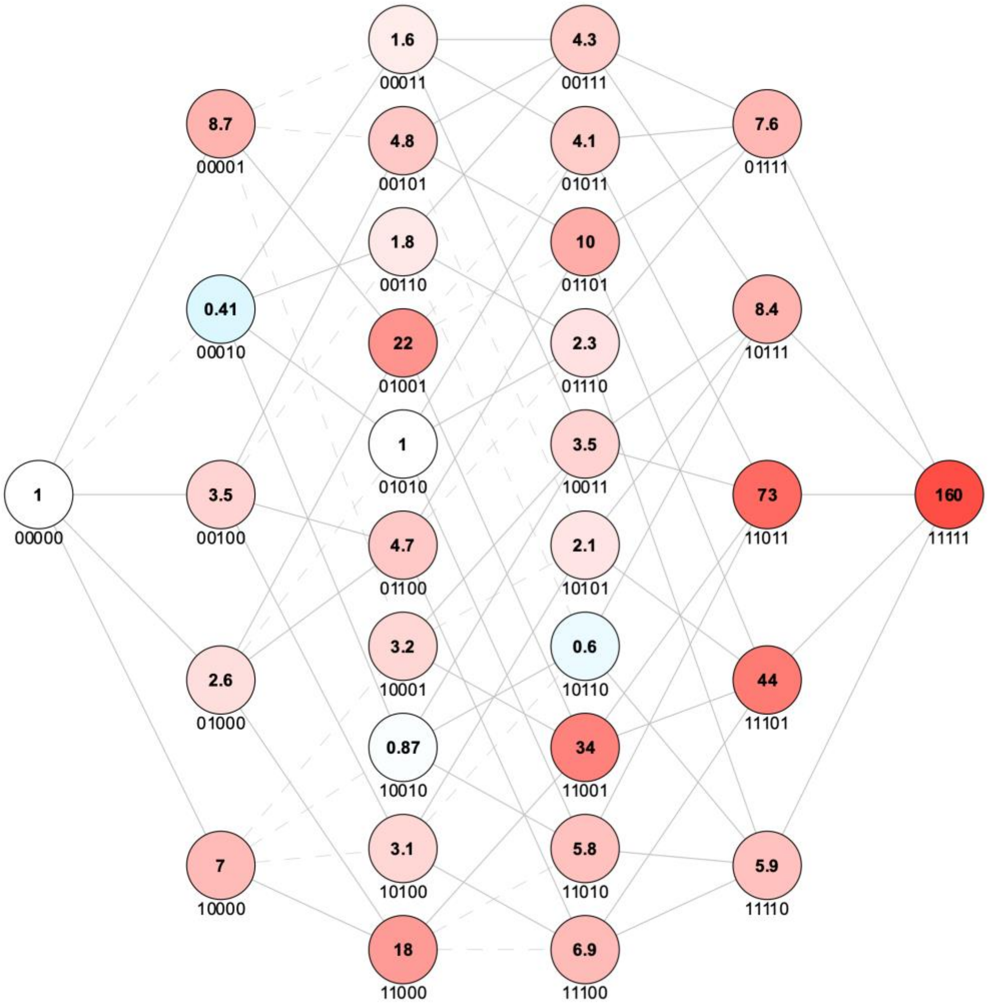

Landscape 15 | Fitness Landscape of Methyl Parathion Hydrolase (MPH) for methyl parathion in copper metal conditions from Anderson *et al.*

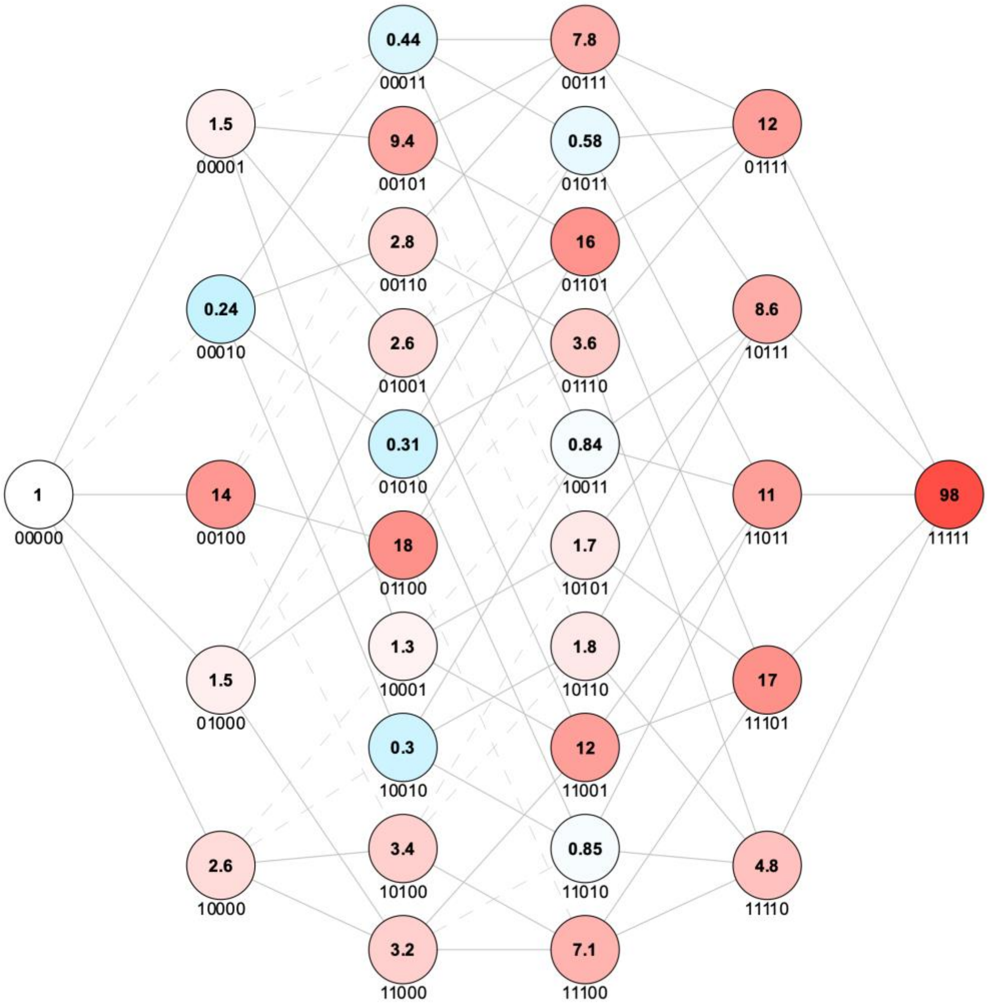

Landscape 16 | Fitness Landscape of Methyl Parathion Hydrolase (MPH) for methyl parathion in magnesium metal conditions from Anderson *et al.*

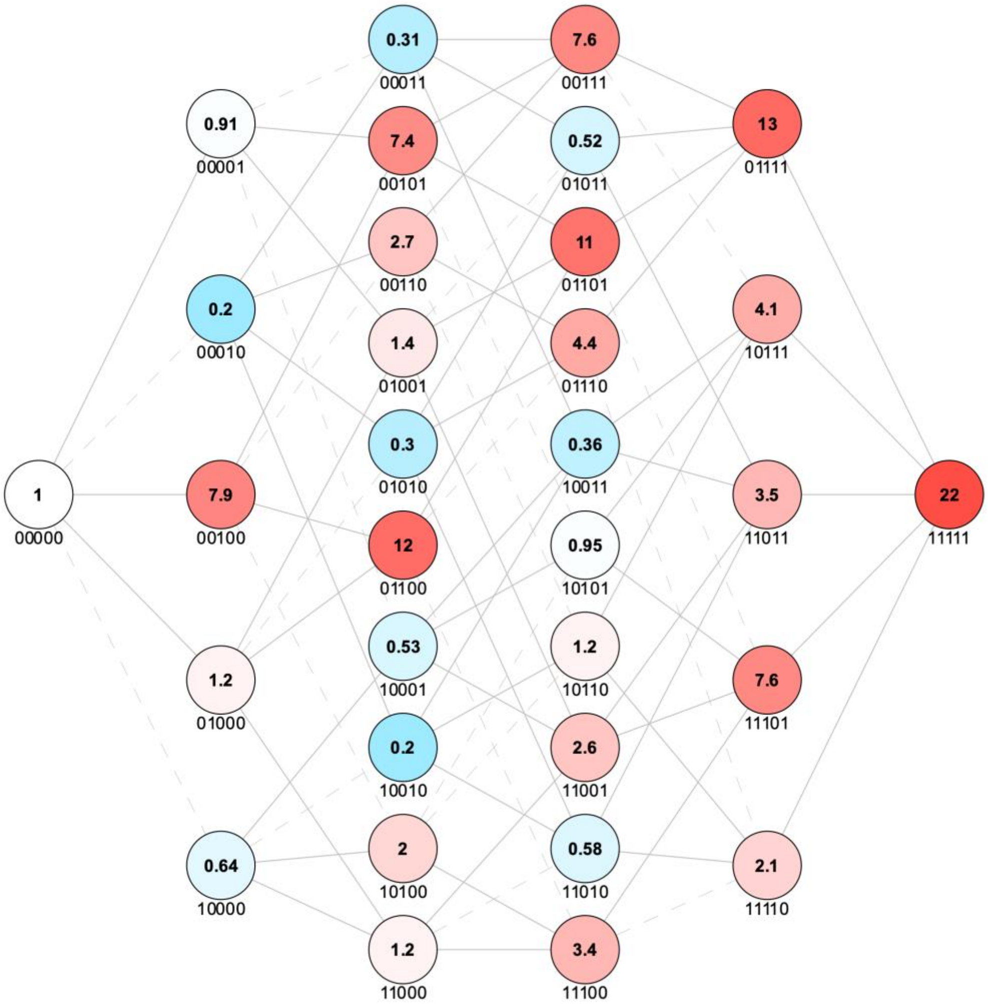

Landscape 17 | Fitness Landscape of Methyl Parathion Hydrolase (MPH) for methyl parathion in manganese metal conditions from Anderson *et al.*

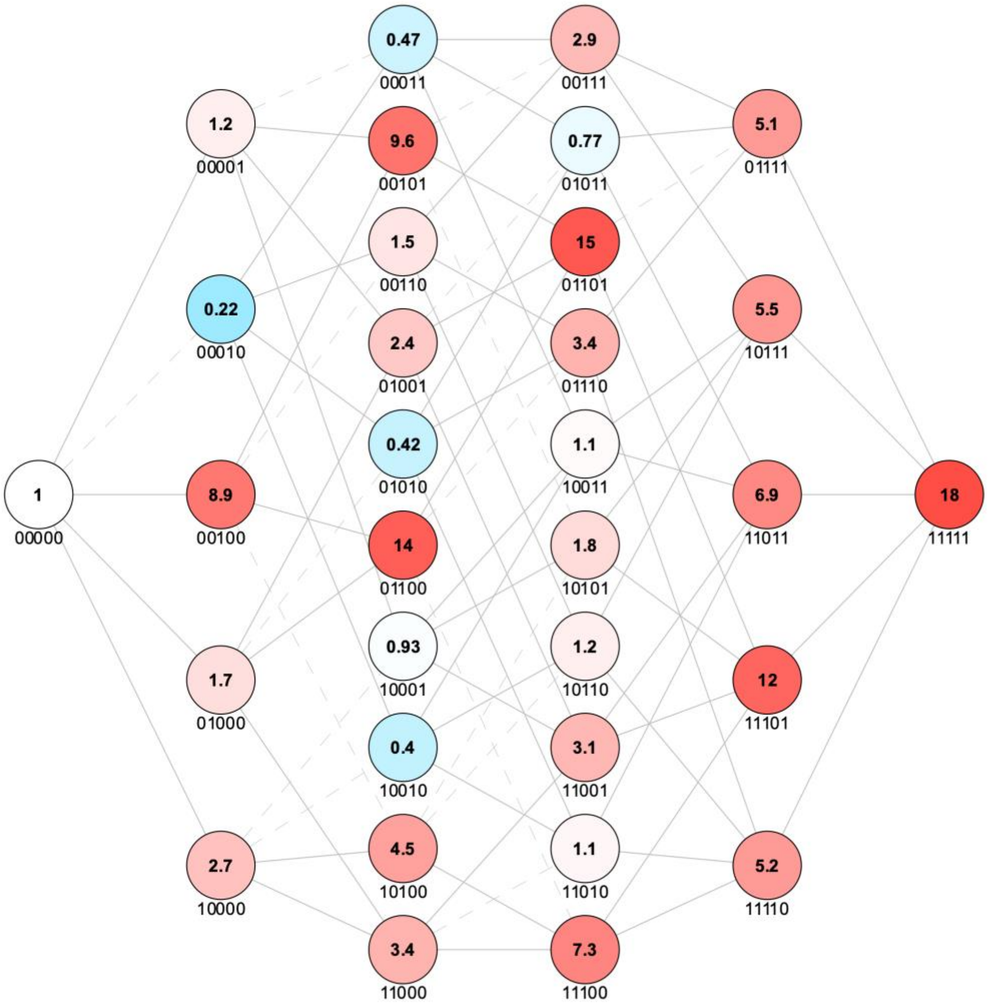

Landscape 18 | Fitness Landscape of Methyl Parathion Hydrolase (MPH) for methyl parathion in nickel metal conditions from Anderson *et al.*

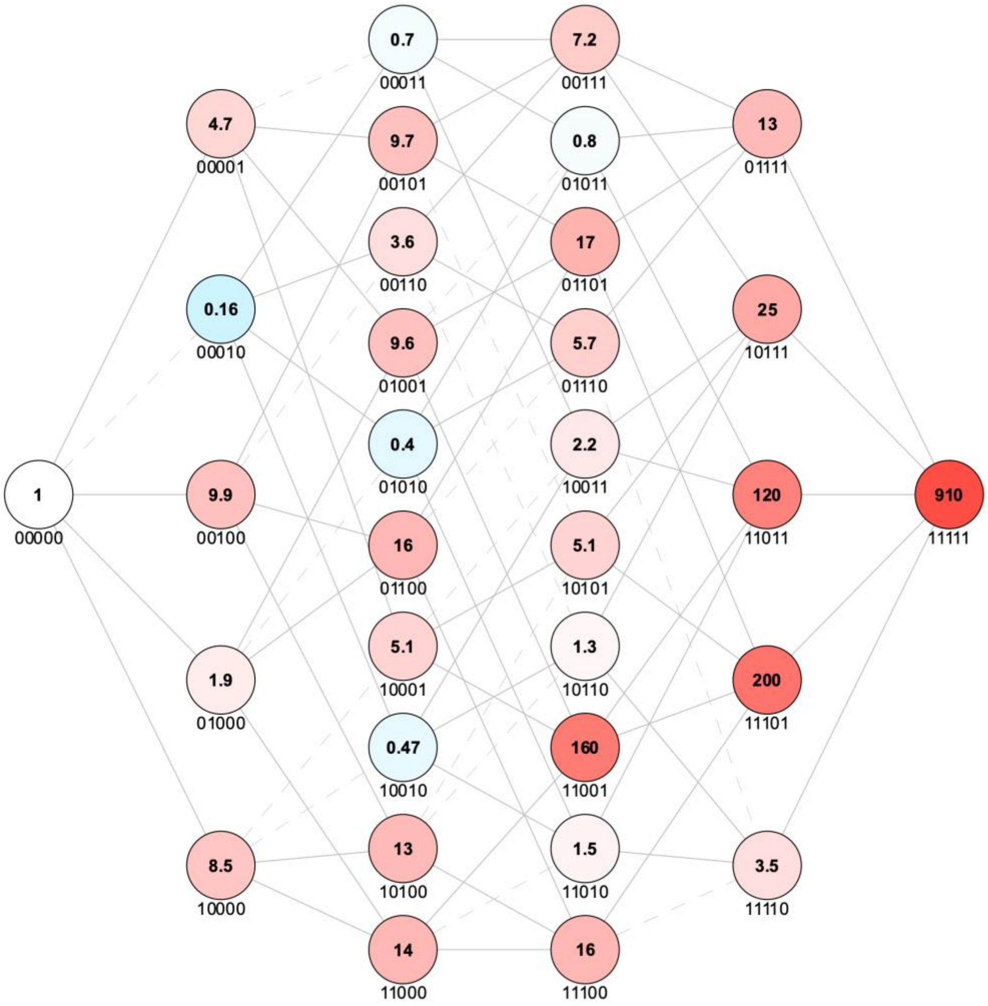

Landscape 19 | Fitness Landscape of Methyl Parathion Hydrolase (MPH) for methyl parathion in zinc metal conditions from Anderson *et al.*

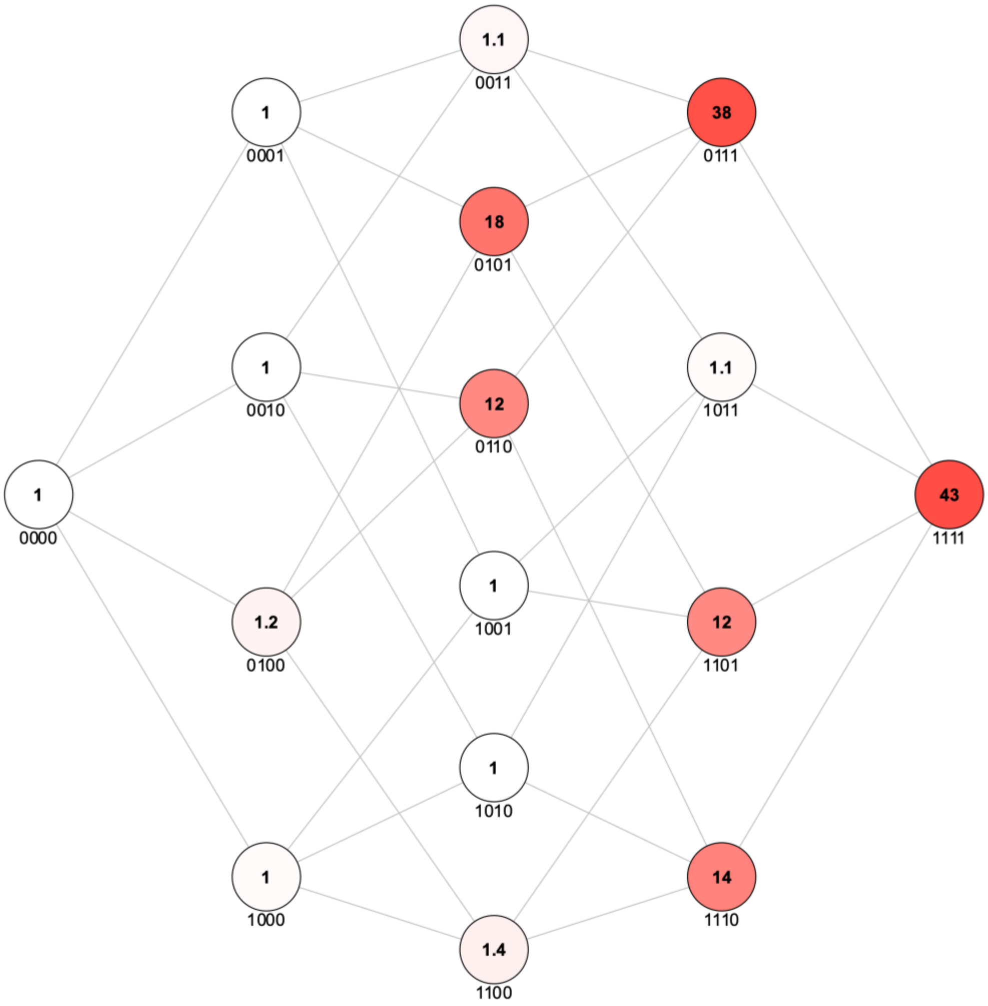

Landscape 20 | Fitness Landscape of beta lactamase OXA-48 for ceftazidime hydrolysis in trajectory 1 from Fröhlich *et al.*

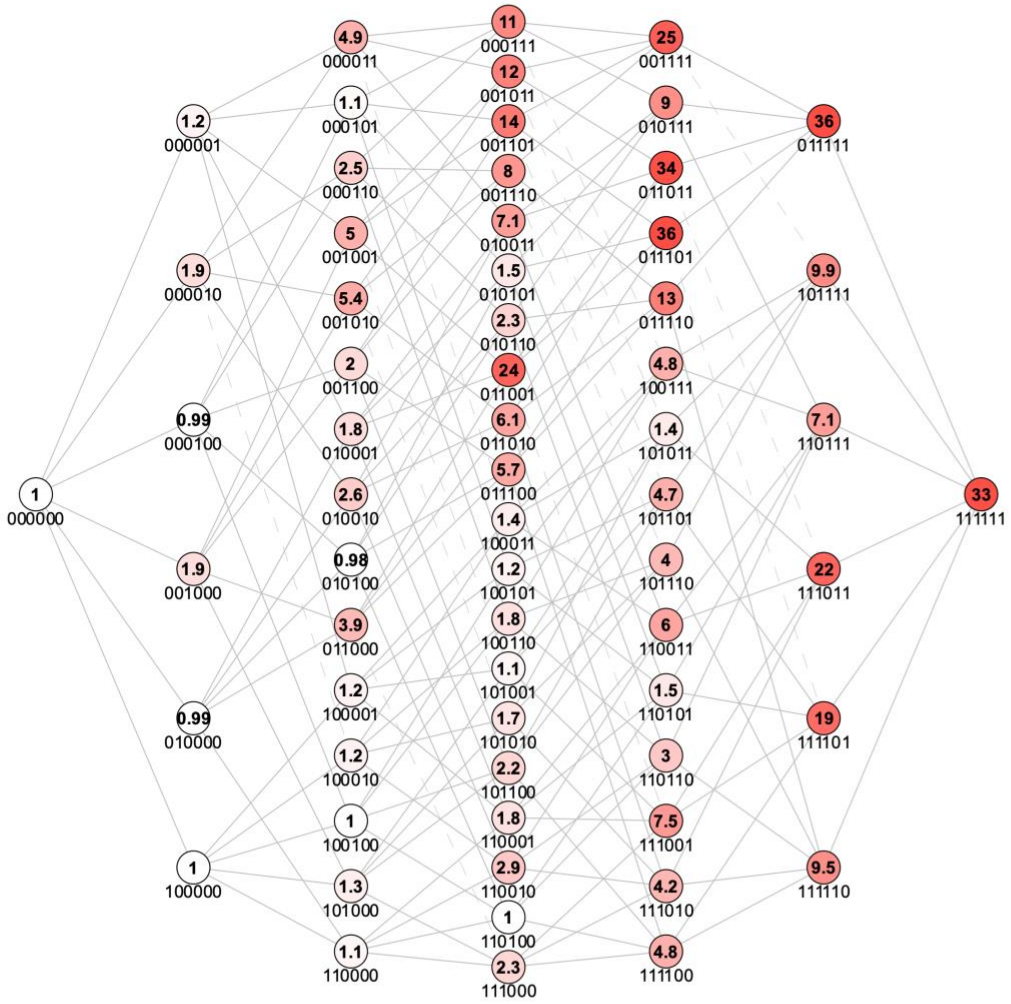

Landscape 21 | Fitness Landscape of beta lactamase OXA-48 for ceftazidime hydrolysis in trajectory 2 from Fröhlich *et al.*

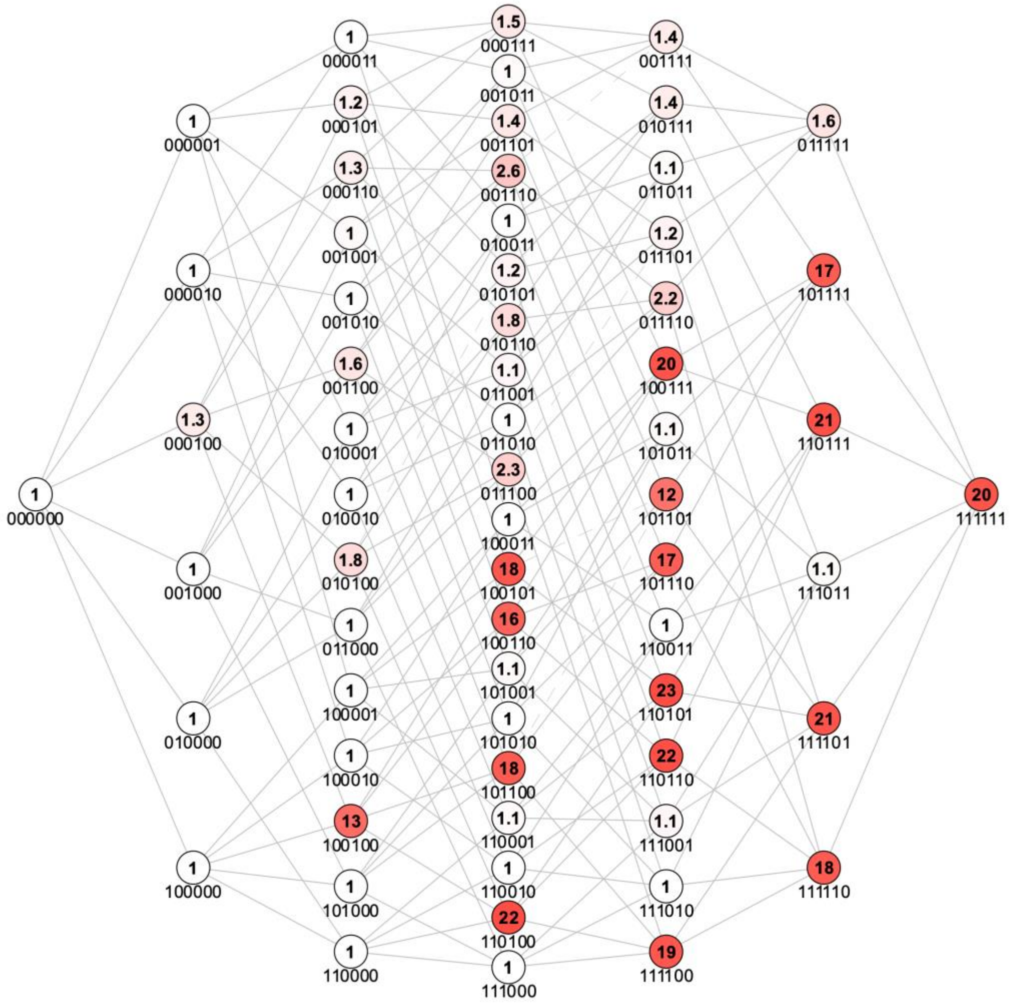

Landscape 22 | Fitness Landscape of beta lactamase OXA-48 for ceftazidime hydrolysis in trajectory 3 from Fröhlich *et al.*

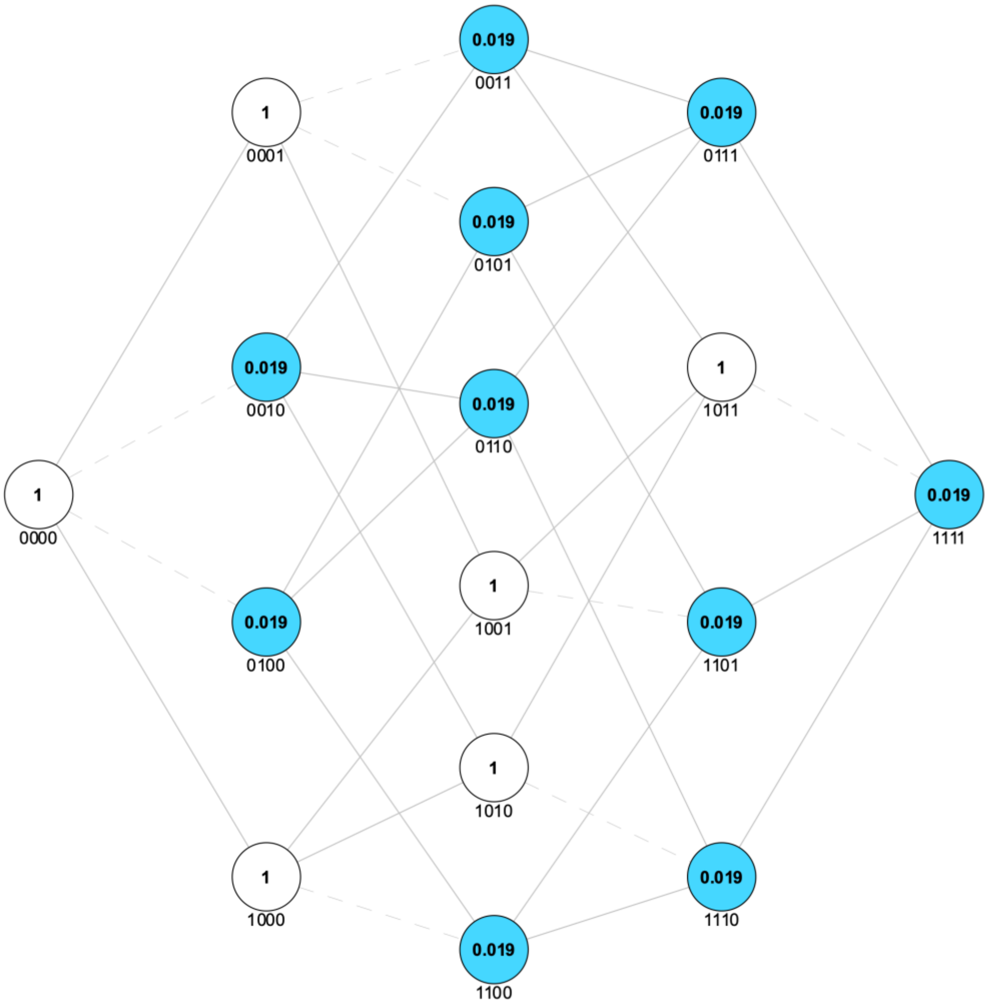

Landscape 23 | Fitness Landscape of beta lactamase OXA-48 for piperacillin hydrolysis in trajectory 1 from Fröhlich *et al.*

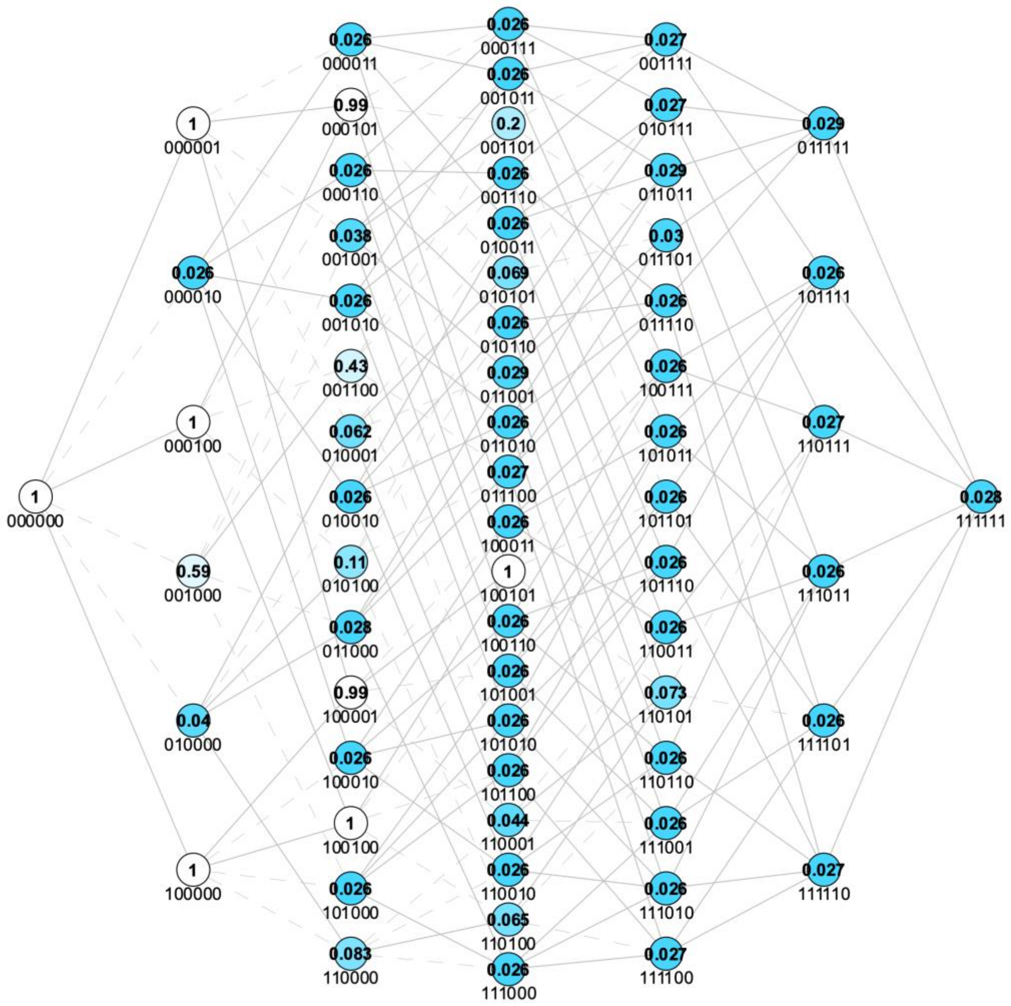

Landscape 24 | Fitness Landscape of beta lactamase OXA-48 for piperacillin hydrolysis in trajectory 2 from Fröhlich *et al.*

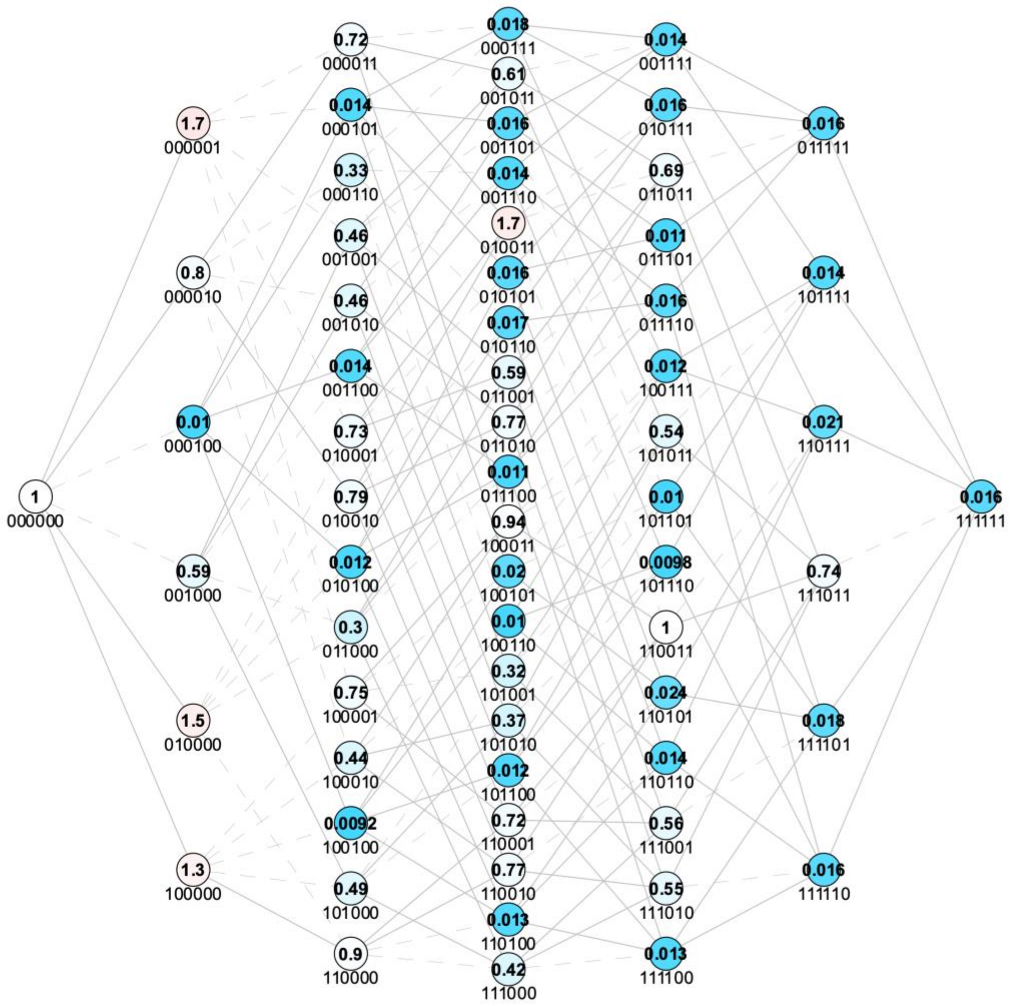

Landscape 25 | Fitness Landscape of beta lactamase OXA-48 for piperacillin hydrolysis in trajectory 3 from Fröhlich *et al.*

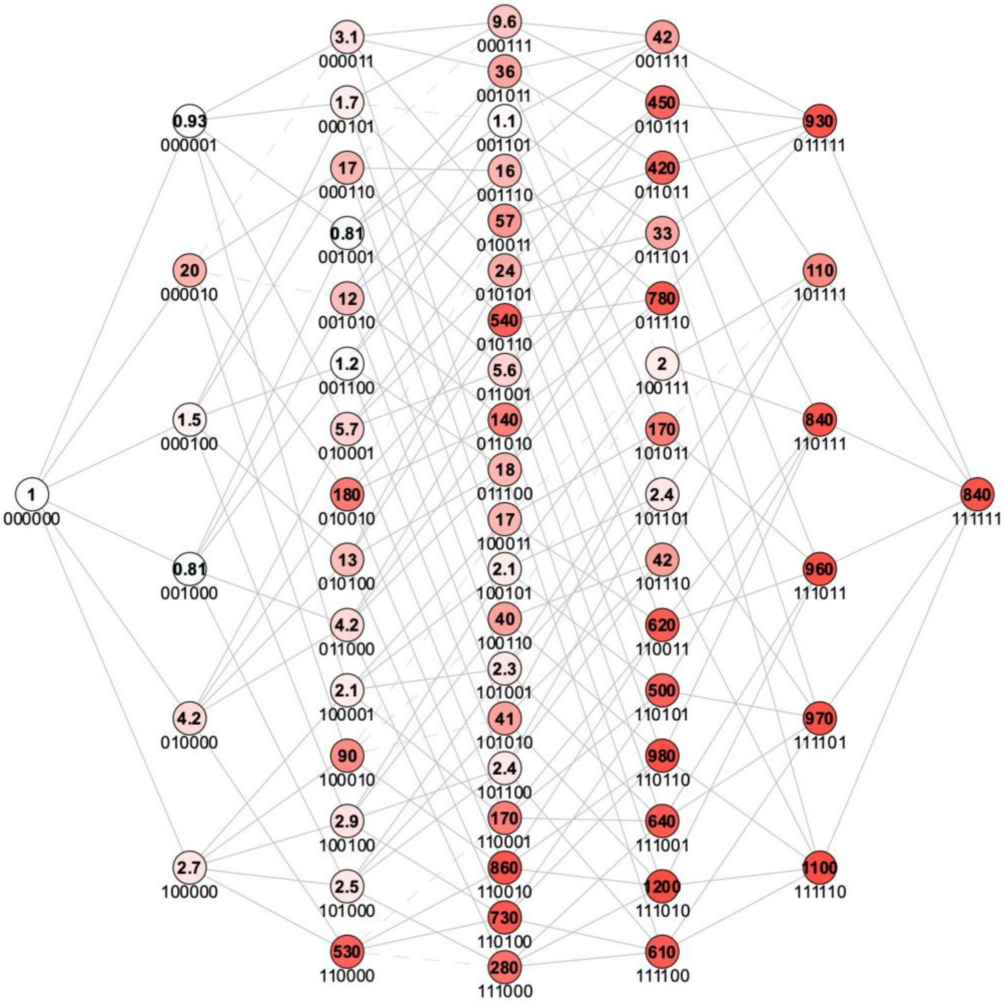

Landscape 26 | Fitness Landscape of phosphotriesterase (PTE) for 2-napthyl hexanoate (2NH) hydrolysis from this publication

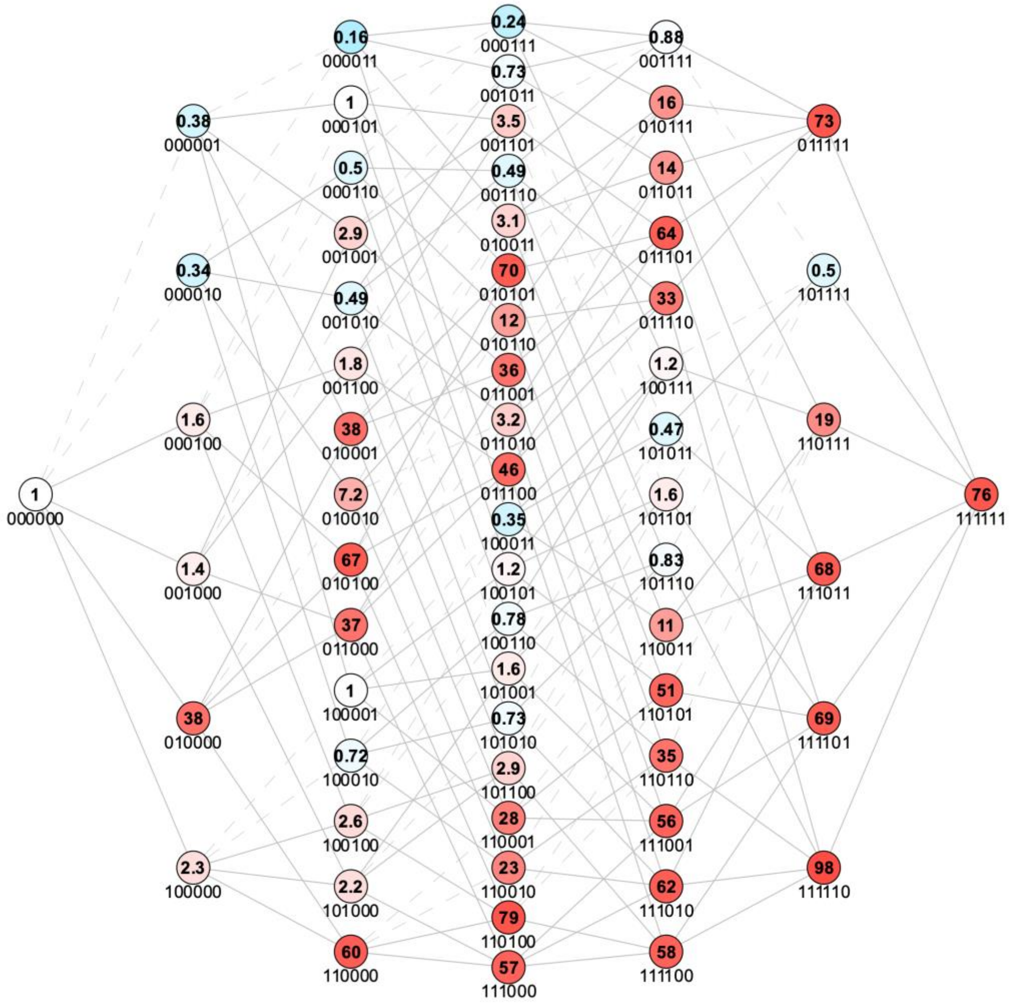

Landscape 27 | Fitness Landscape of phosphotriesterase (PTE) for butyrate hydrolysis from Miton *et al.*

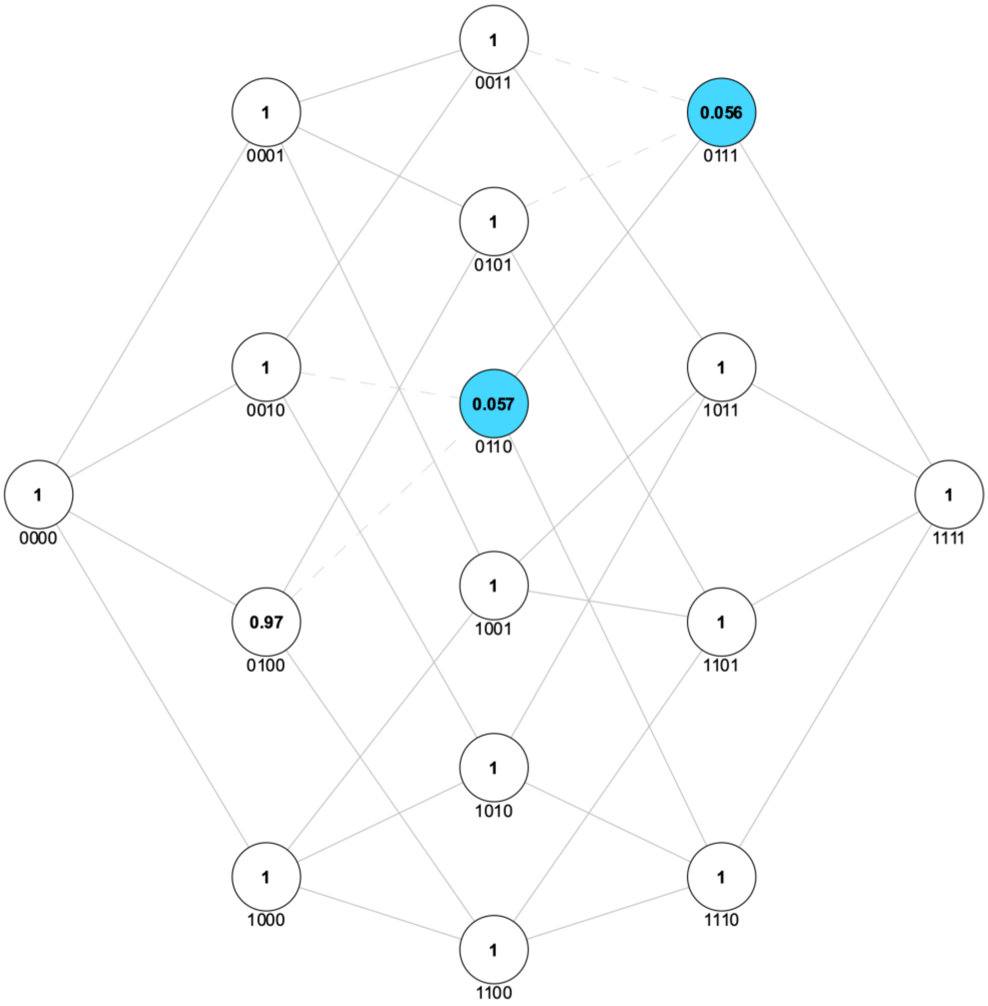

Landscape 28 | Fitness Landscape of beta lactamase TEM in AM antibiotic from Mira *et al.*

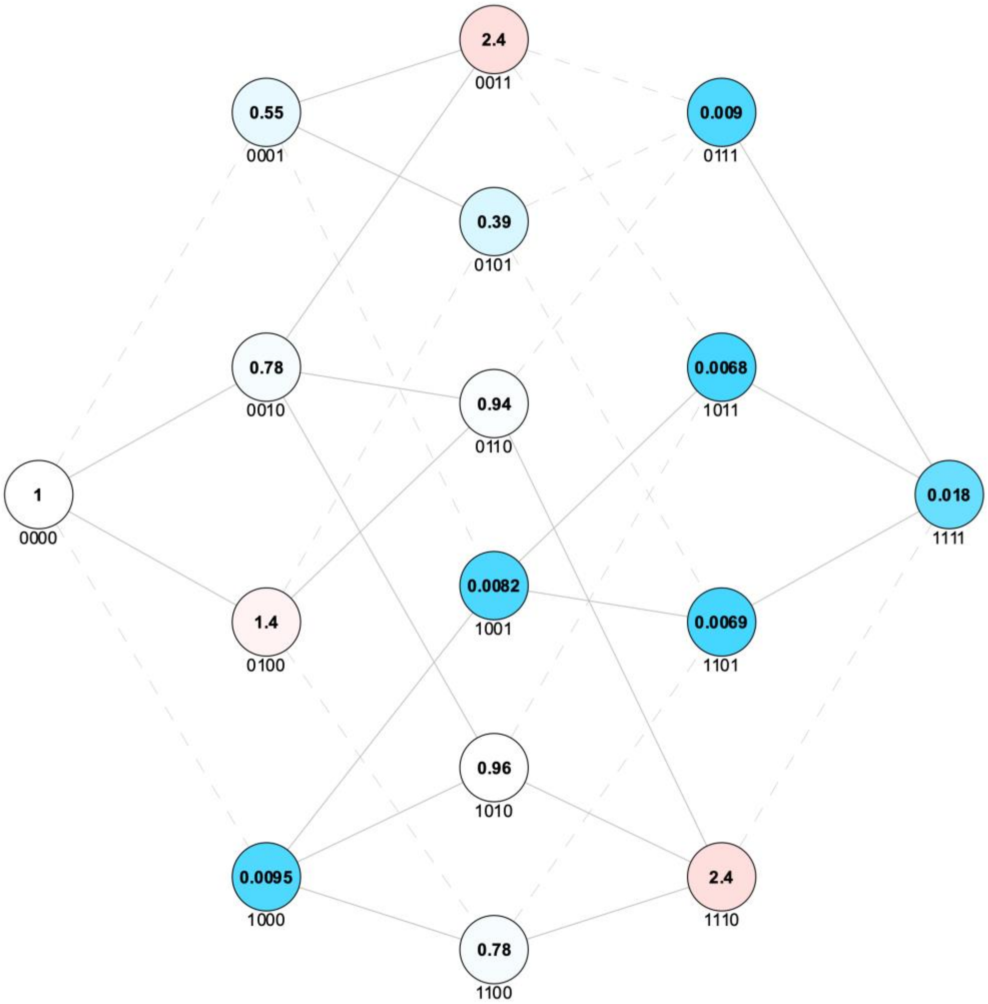

Landscape 29 | Fitness Landscape of beta lactamase TEM in CEC antibiotic from Mira *et al.*

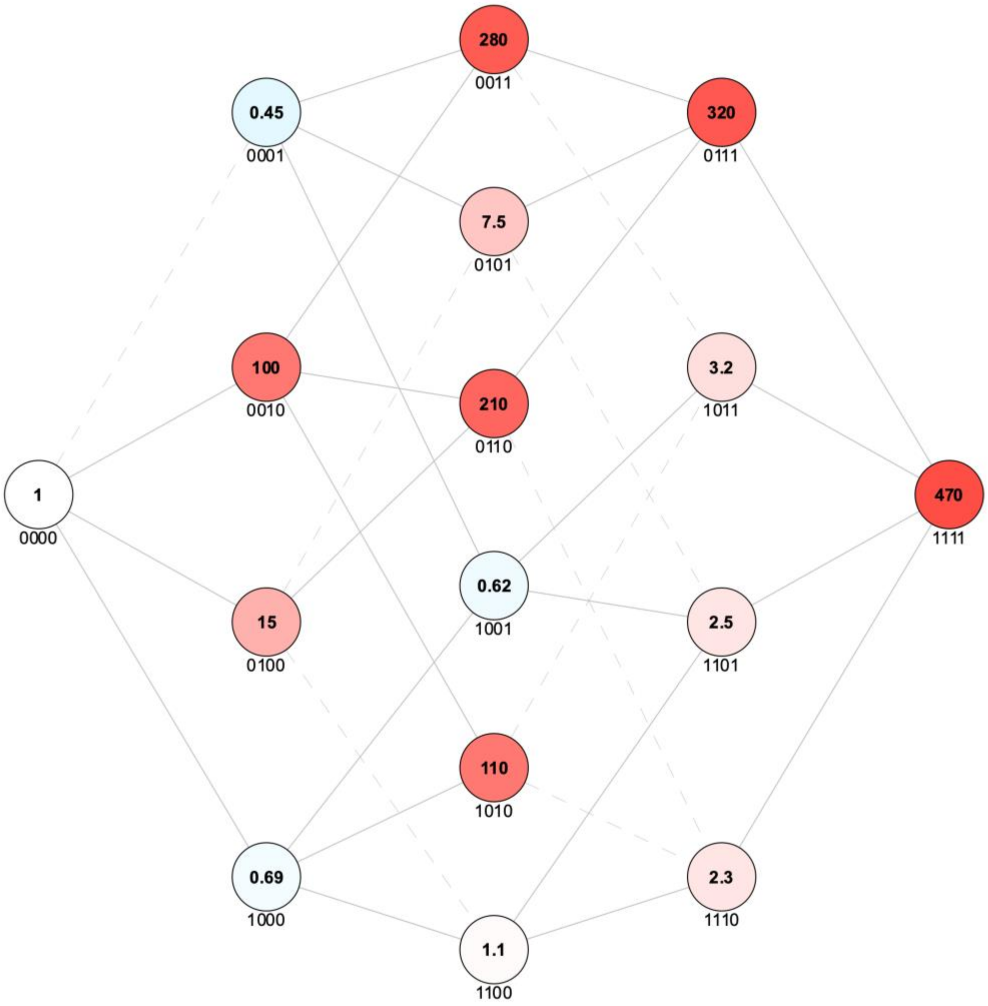

Landscape 30 | Fitness Landscape of beta lactamase TEM in CPD antibiotic from Mira *et al.*

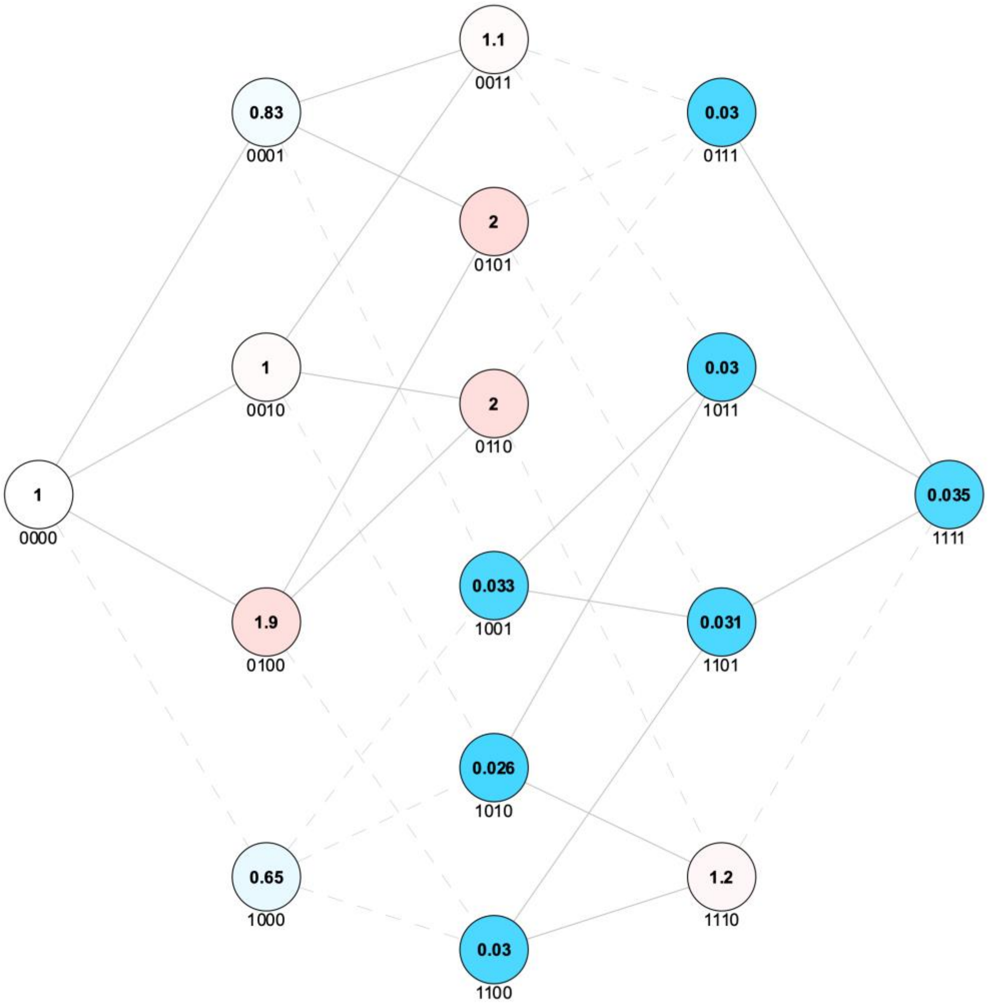

Landscape 31 | Fitness Landscape of beta lactamase TEM in CPR antibiotic from Mira *et al.*

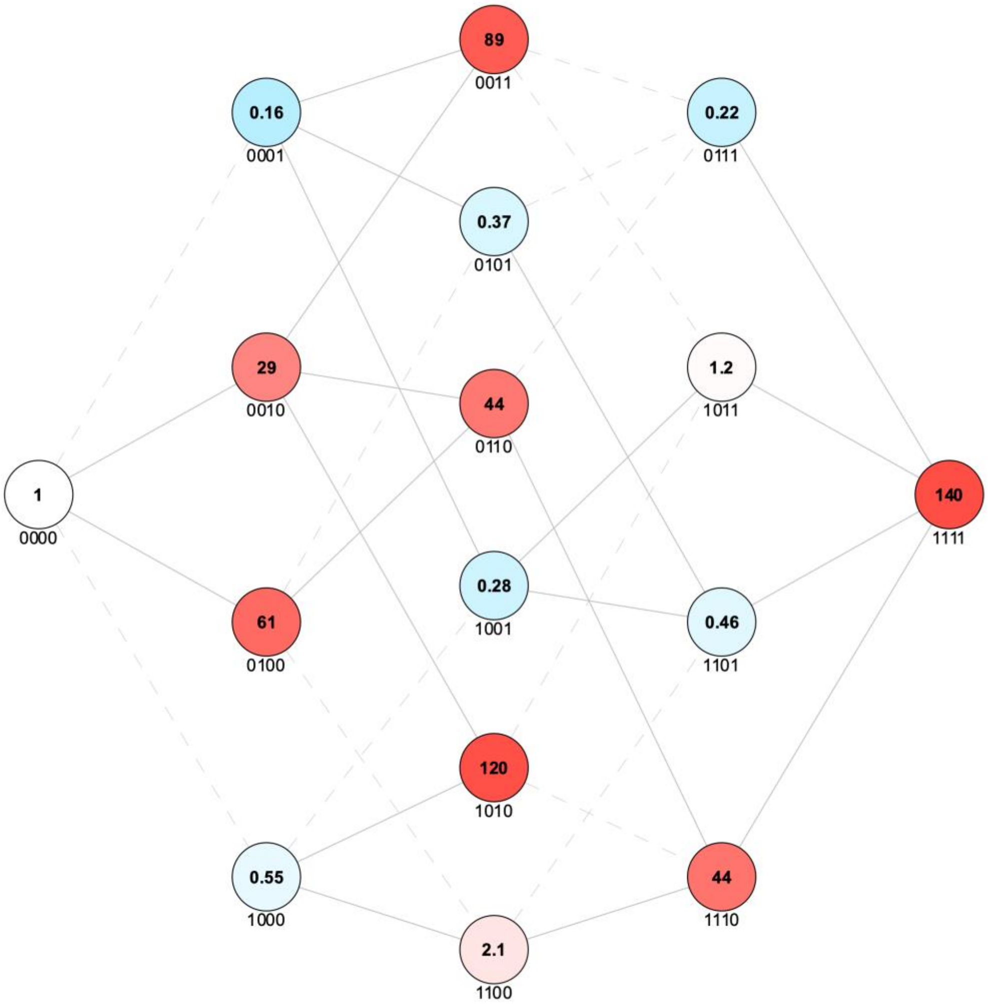

Landscape 32 | Fitness Landscape of beta lactamase TEM in CRO antibiotic from Mira *et al.*

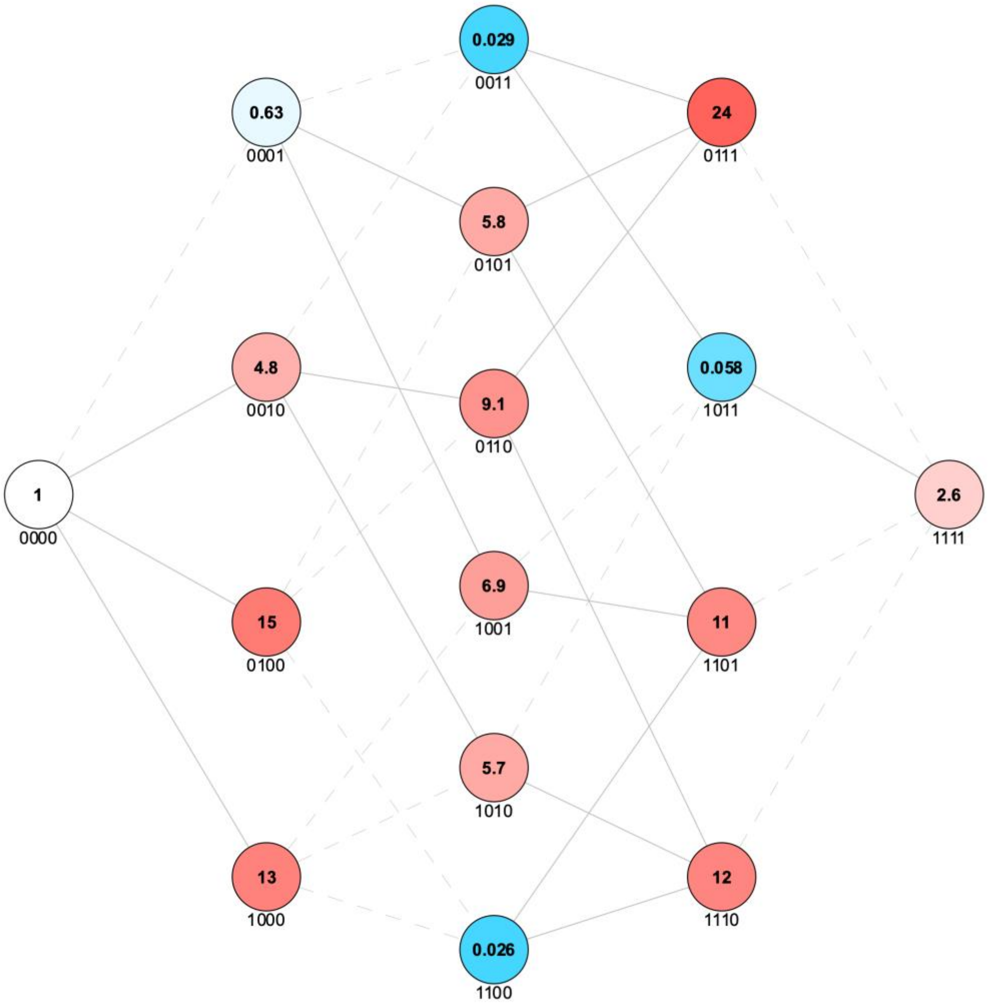

Landscape 33 | Fitness Landscape of beta lactamase TEM in CTT antibiotic from Mira *et al.*

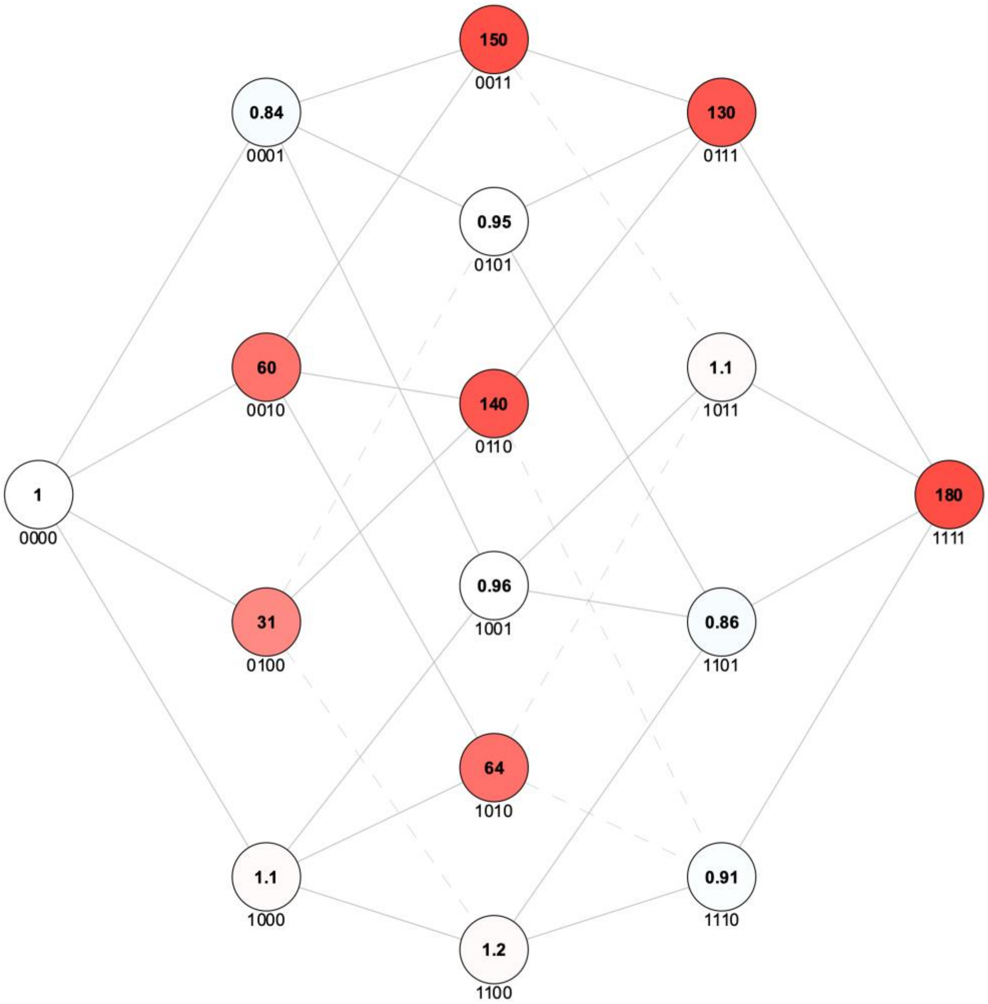

Landscape 34 | Fitness Landscape of beta lactamase TEM in CTX antibiotic from Mira *et al.*

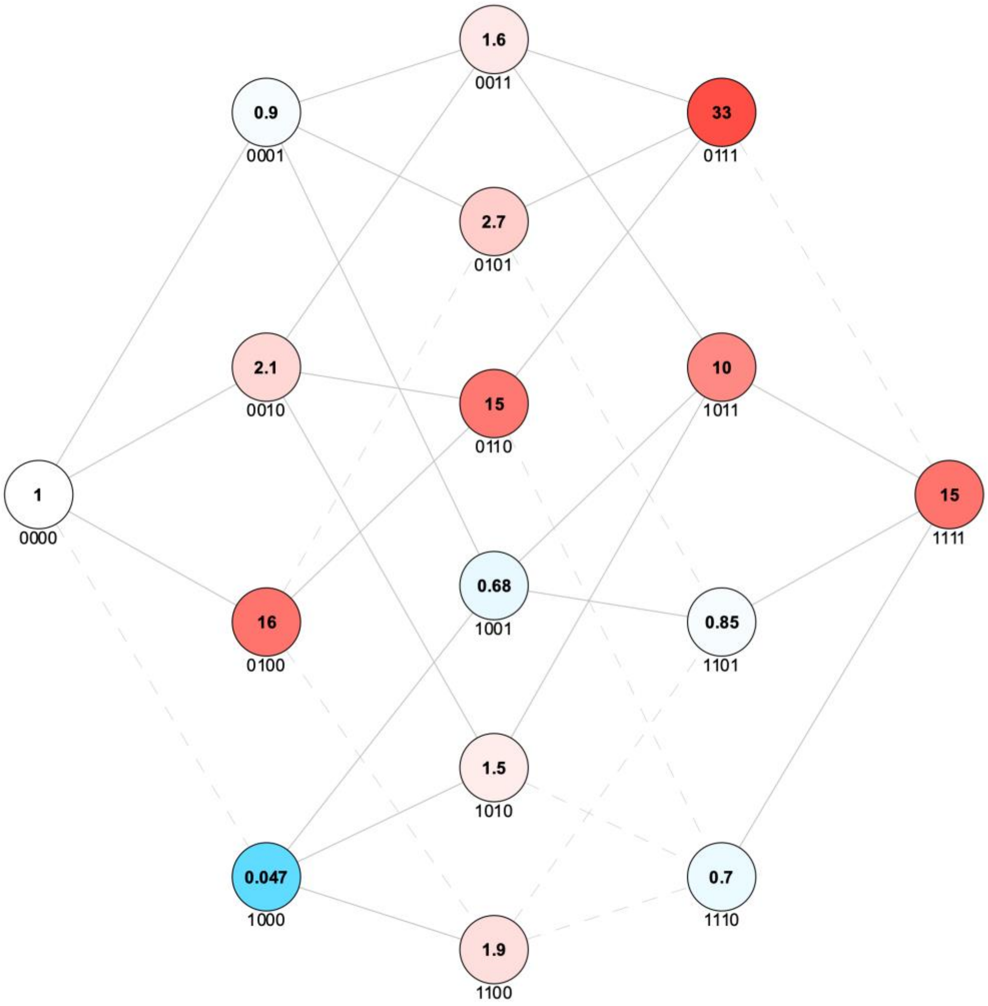

Landscape 35 | Fitness Landscape of beta lactamase TEM in CXM antibiotic from Mira et al.

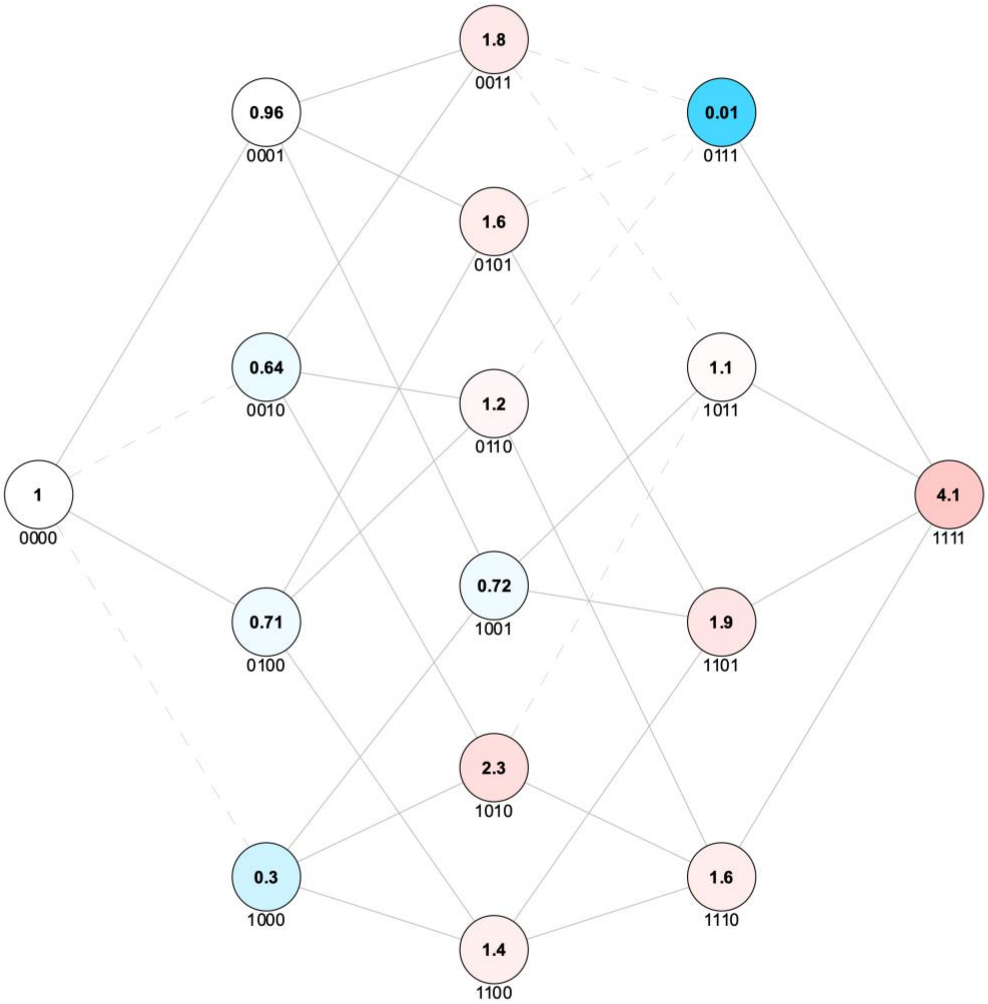

Landscape 36 | Fitness Landscape of beta lactamase TEM in FEP antibiotic from Mira et al.

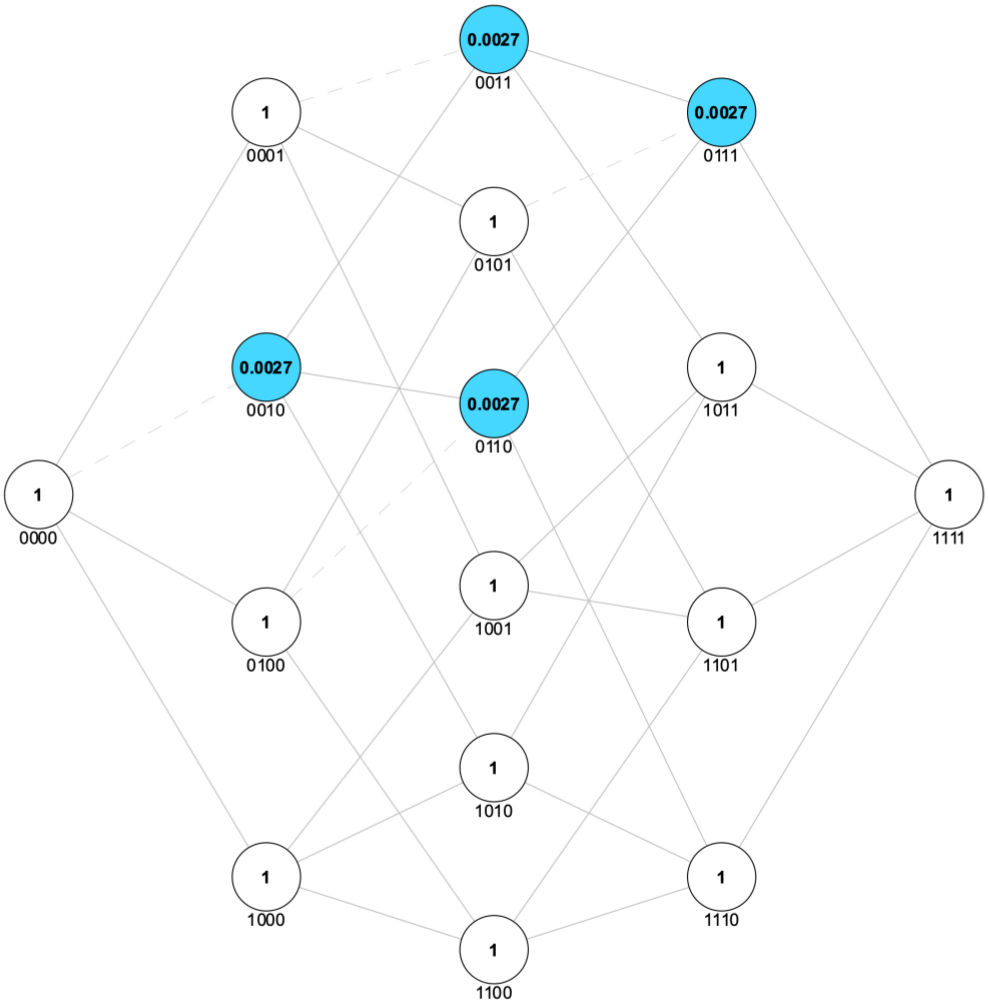

Landscape 37 | Fitness Landscape of beta lactamase TEM in SAM antibiotic from Mira et al.

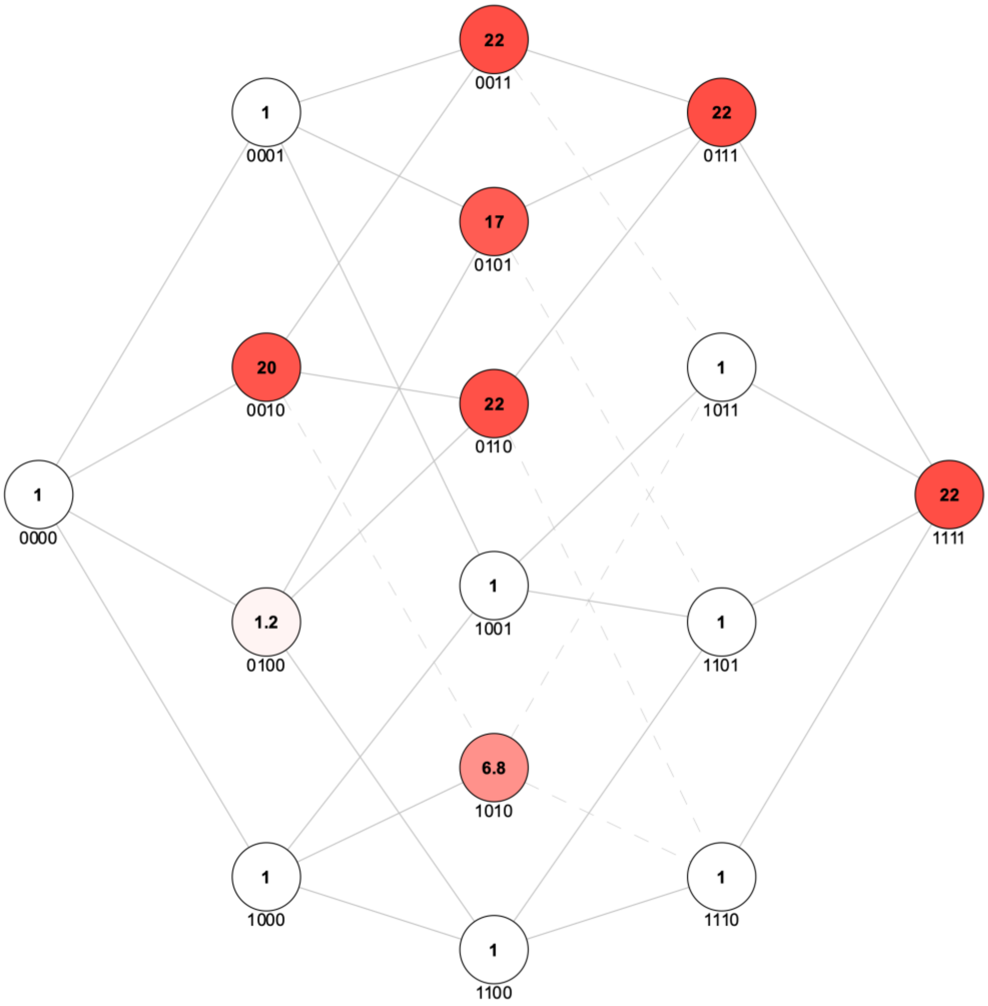

Landscape 38 | Fitness Landscape of beta lactamase TEM in ZOX antibiotic from Mira et al.

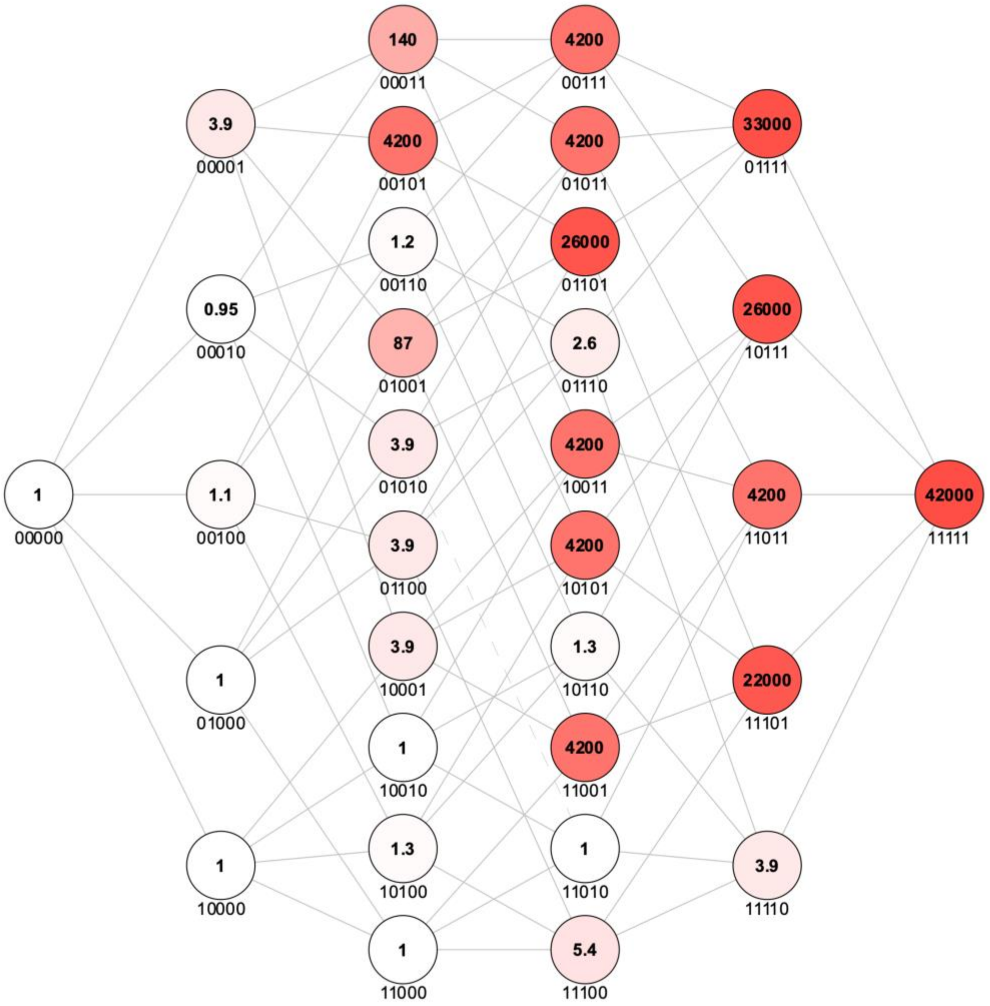

Landscape 39 | Fitness Landscape of beta lactamase TEM from Weinreich *et al.*

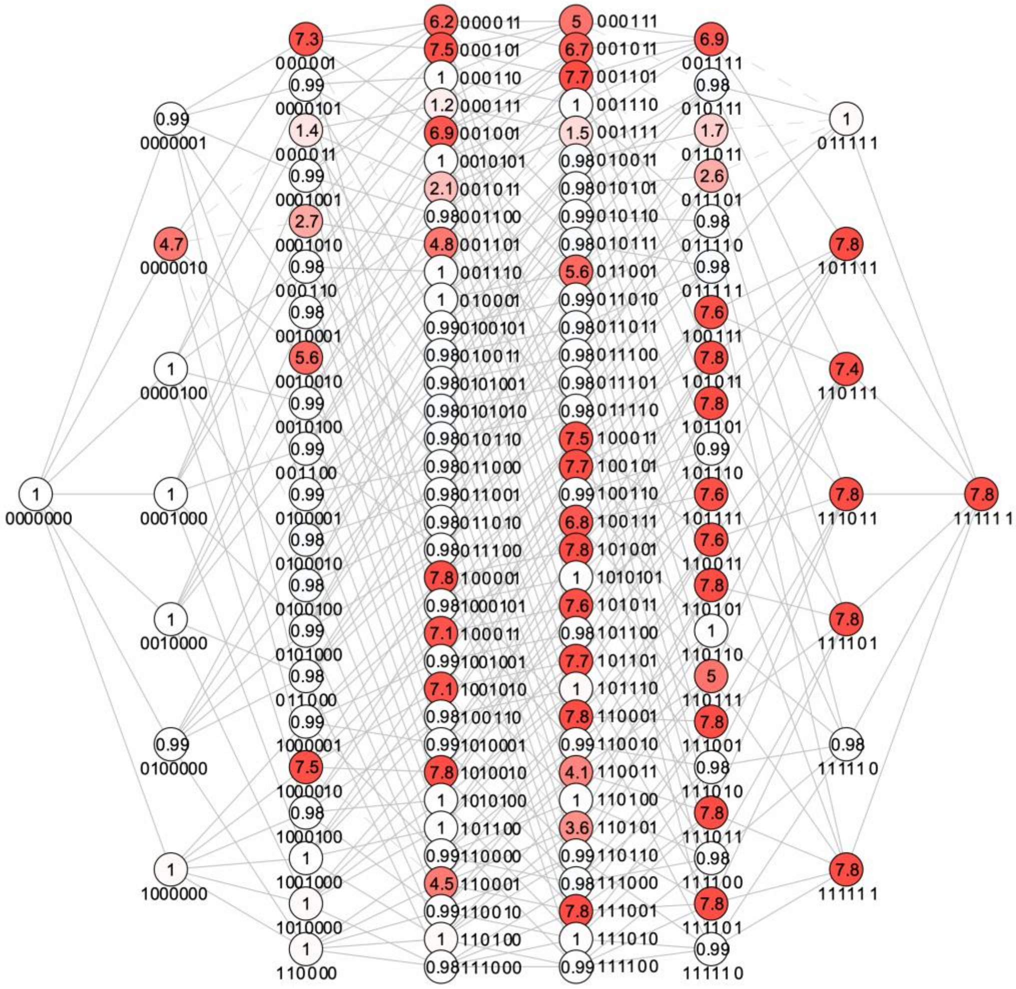

Landscape 40 | Fitness Landscape of nitroreductase NfsA in the 20_39 trajectory from Hall *et al.*

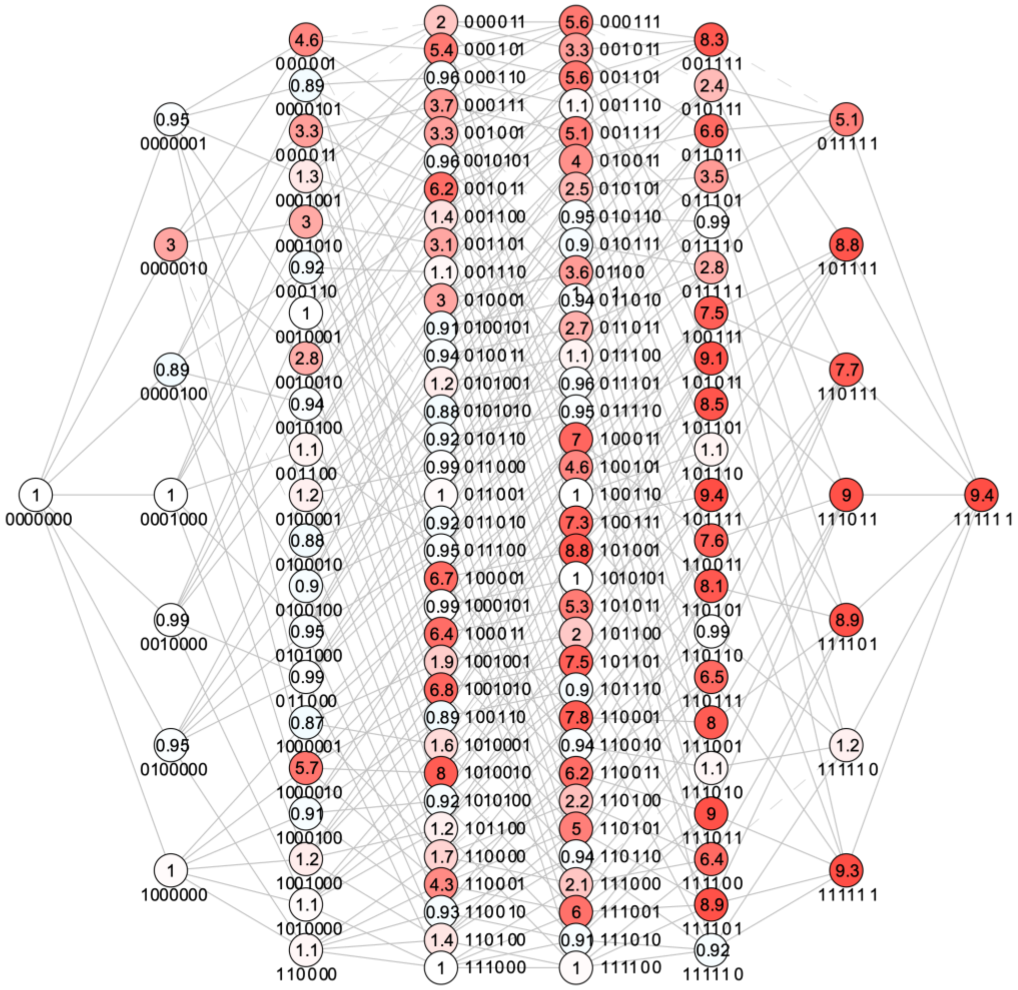

Landscape 41 | Fitness Landscape of nitroreductase NfsA in the 36_37 trajectory from Hall *et al.*
