# Supplementary material for: Pervasive epistasis exposes intramolecular networks in adaptive enzyme evolution
Source: Nat Commun. 2023 Dec 21;14:8508. doi: 10.1038/s41467-023-44333-5 (PMC10739712; doi:10.1038/s41467-023-44333-5)
Supplement: Supplementary file 6 — Supplementary Data 3 [file 41467_2023_44333_MOESM6_ESM.pdf]

Supplementary Data 3

The following analysis outlines the exploration of putative non-linear transformations, then details how the four-parameter transform was utilized.

Non-linear transformation evaluation

Assessing degrees for splines

We began by evaluating the use of cubic splines with a penalty score encouraging monotonicity using a general additive model in the `mgcv` package. We iteratively increased the number of degrees from 1-5, and evaluated the fits using AIC to select the strongest model for each landscape, then logged the number of degrees that should be used for each transform.

Visual spline model evaluation

We then visually evaluated the cubic spline transformations (in blue) for each landscape with the x-axis representing additive effects, as predicted by the first-order background-averaged model, and the y-axis representing observed effects.

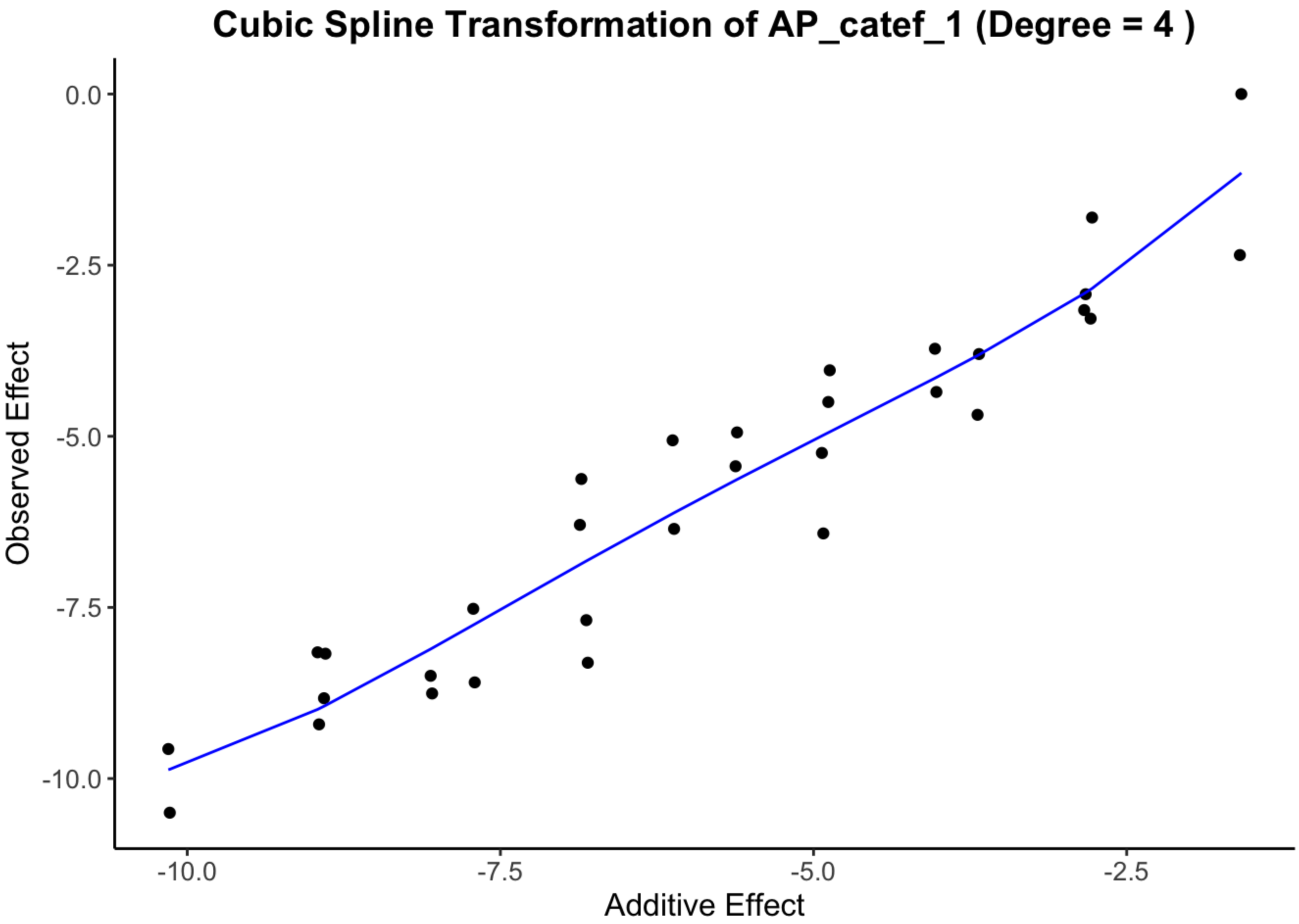
Cubic Spline Transformation of AP_catef_1 (Degree = 4)
Observed Effect
0.0
-2.5
-5.0
-7.5
-10.0
-10.0
-7.5
-5.0
-2.5
Additive Effect

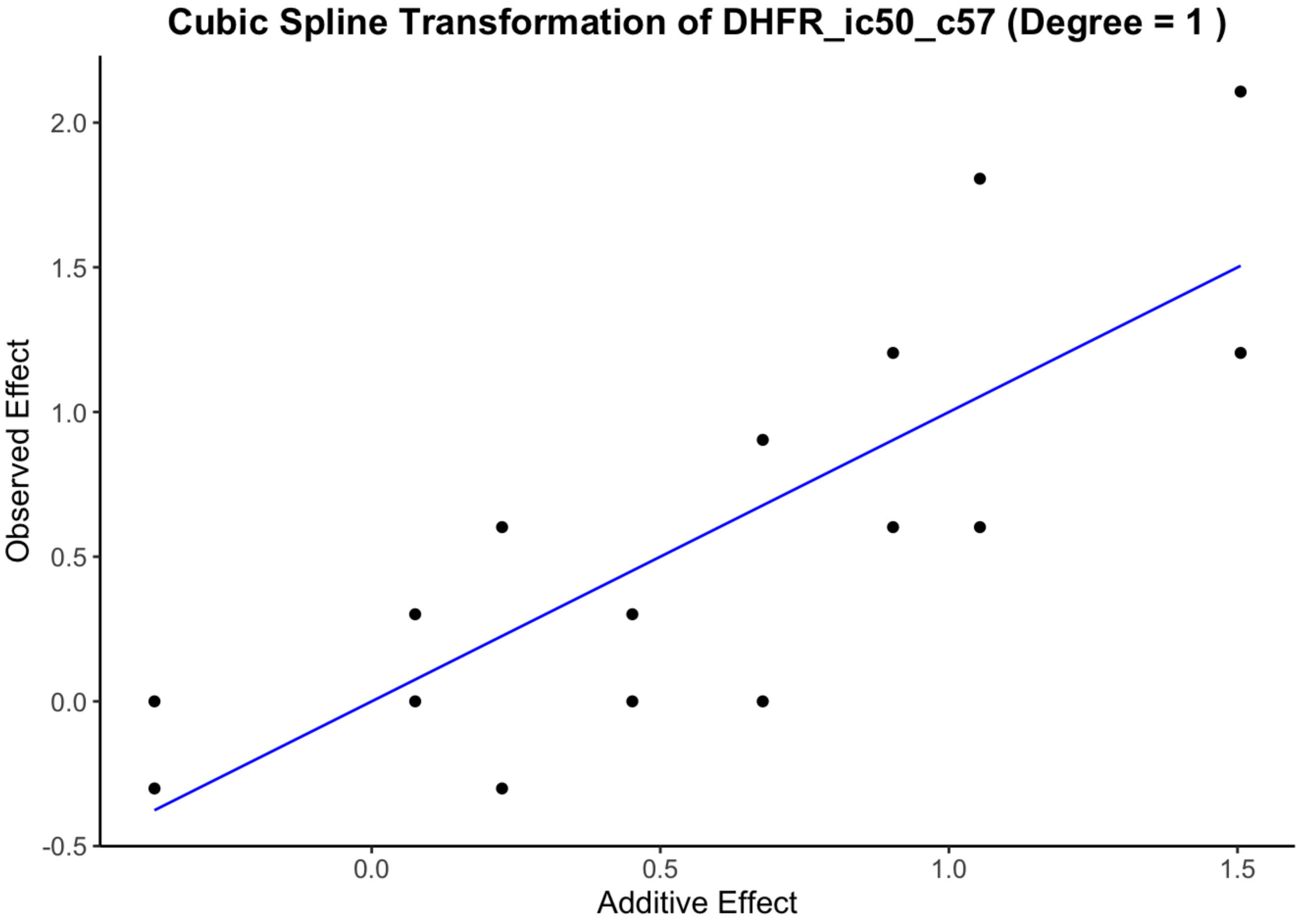
Cubic Spline Transformation of DHFR_ic50_c57 (Degree = 1)
Observed Effect
2.0
1.5
1.0
0.5
0.0
-0.5
0.0
0.5
1.0
1.5
Additive Effect

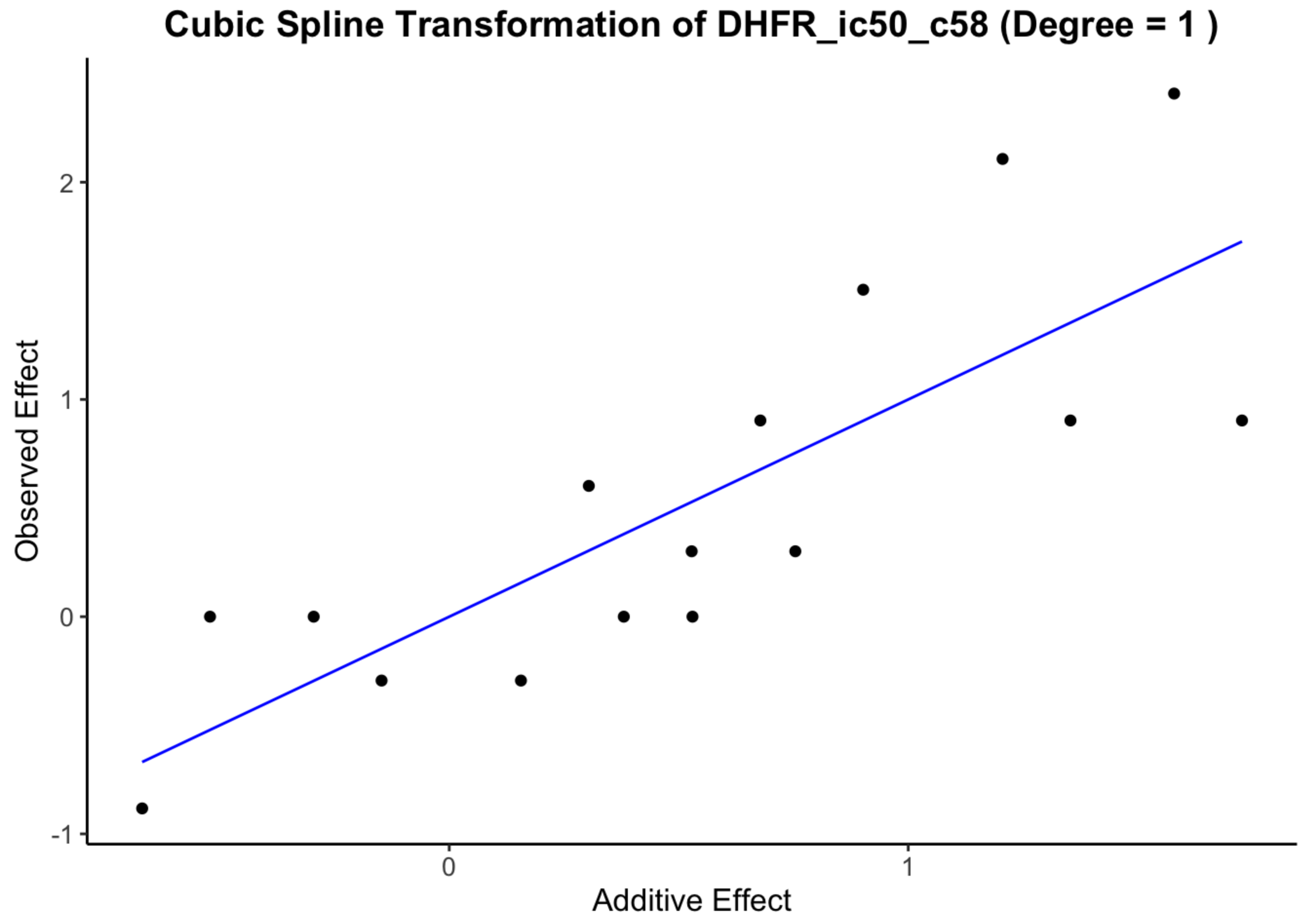

Cubic Spline Transformation of DHFR_ic50_c58 (Degree = 1)
Observed Effect
Additive Effect
2
1
0
-1
0
1

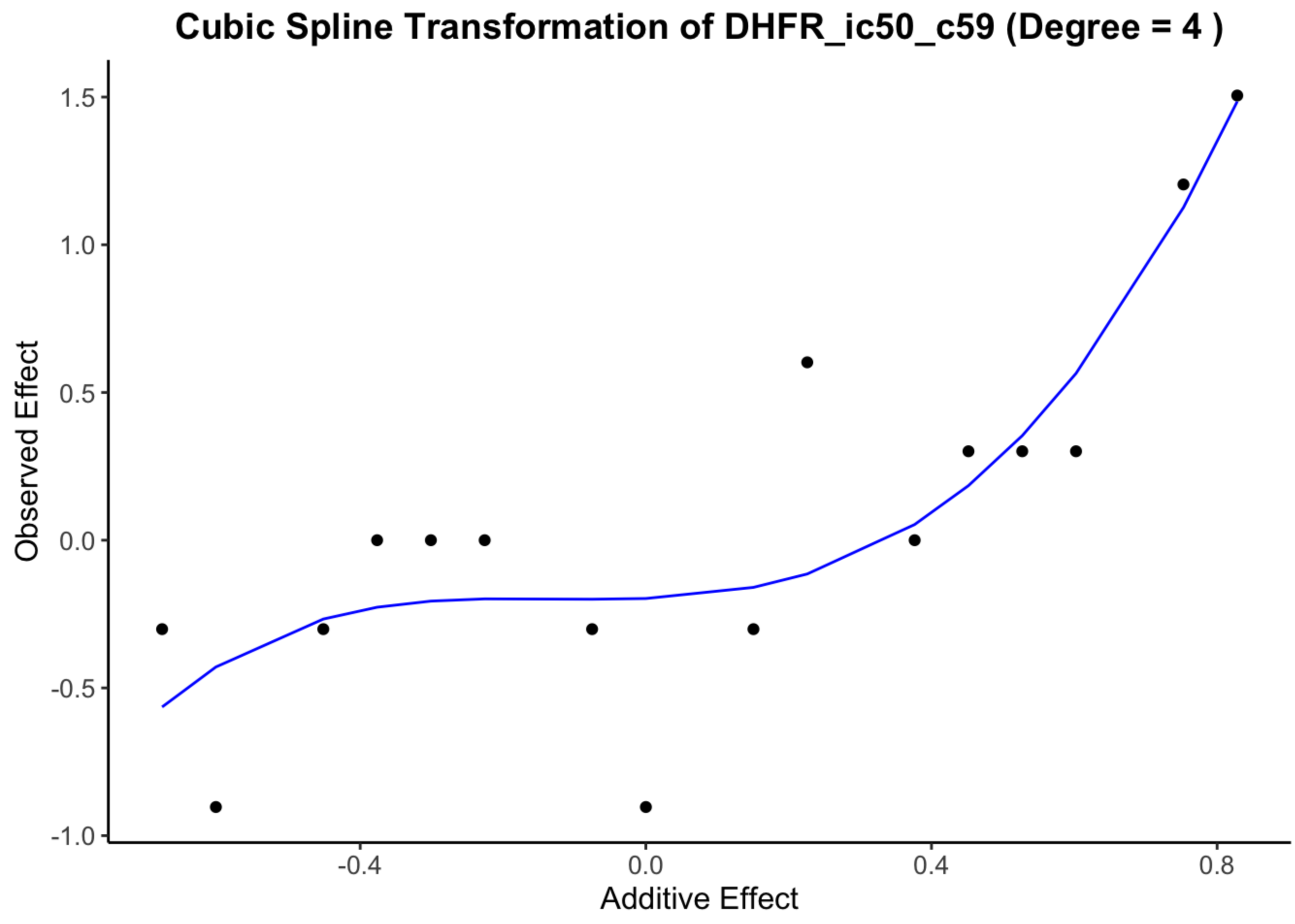

Cubic Spline Transformation of DHFR_ic50_c59 (Degree = 4)
Observed Effect
Additive Effect
1.5
1.0
0.5
0.0
-0.5
-1.0
-0.4
0.0
0.4
0.8

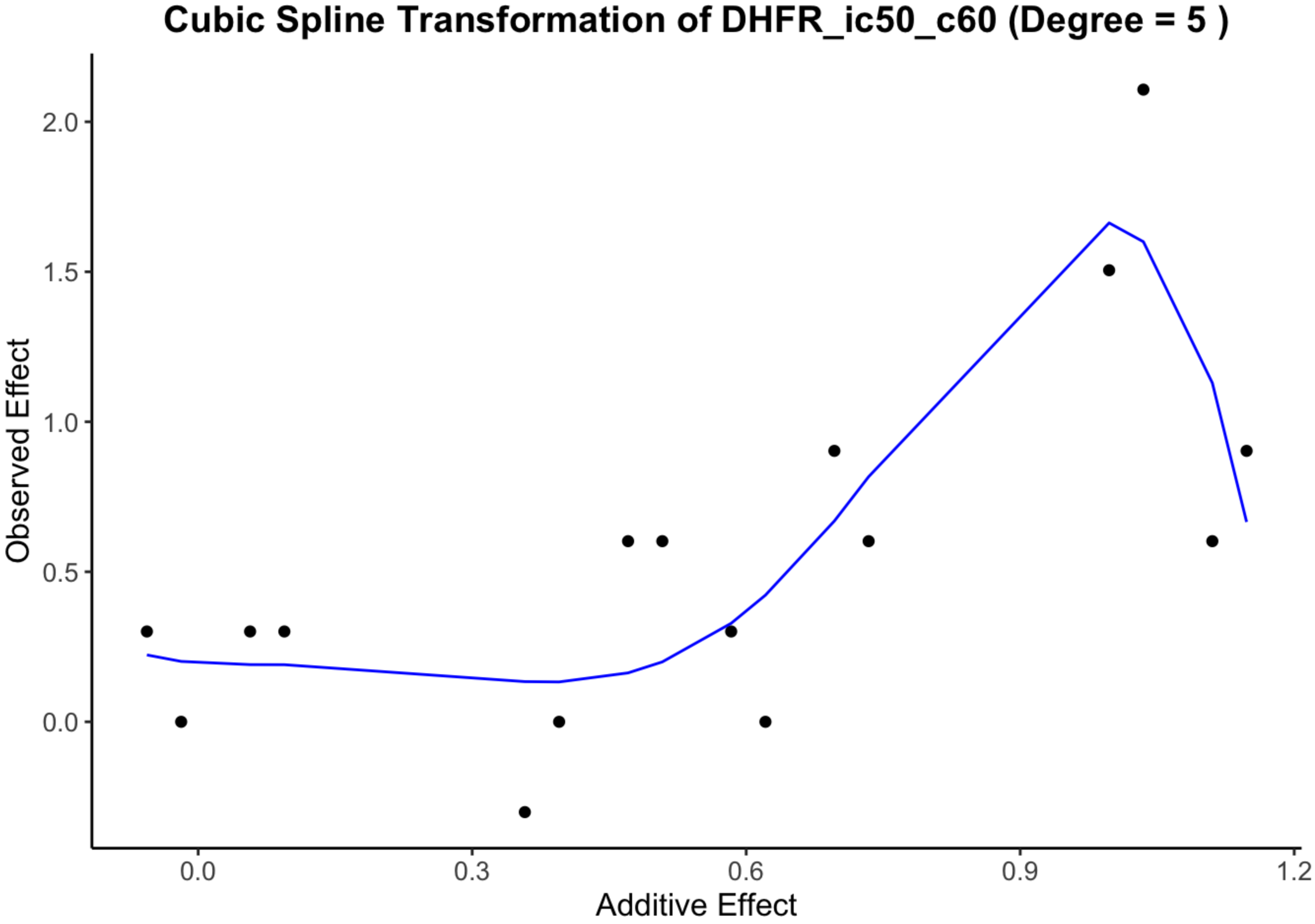
Cubic Spline Transformation of DHFR_ic50_c60 (Degree = 5)
Observed Effect
Additive Effect
2.0
1.5
1.0
0.5
0.0
0.0
0.3
0.6
0.9
1.2

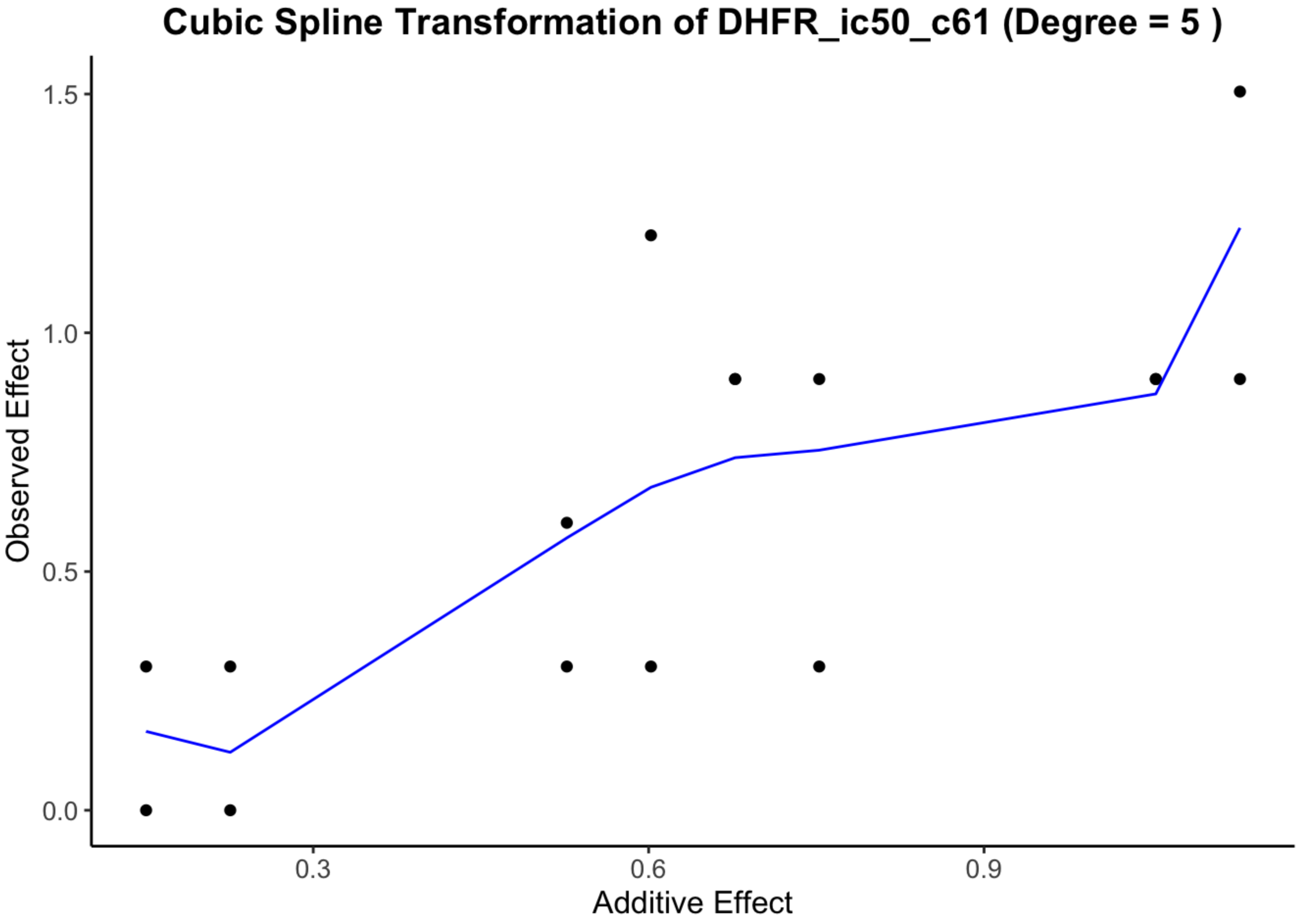
Cubic Spline Transformation of DHFR_ic50_c61 (Degree = 5)
Observed Effect
Additive Effect
1.5
1.0
0.5
0.0
0.3
0.6
0.9

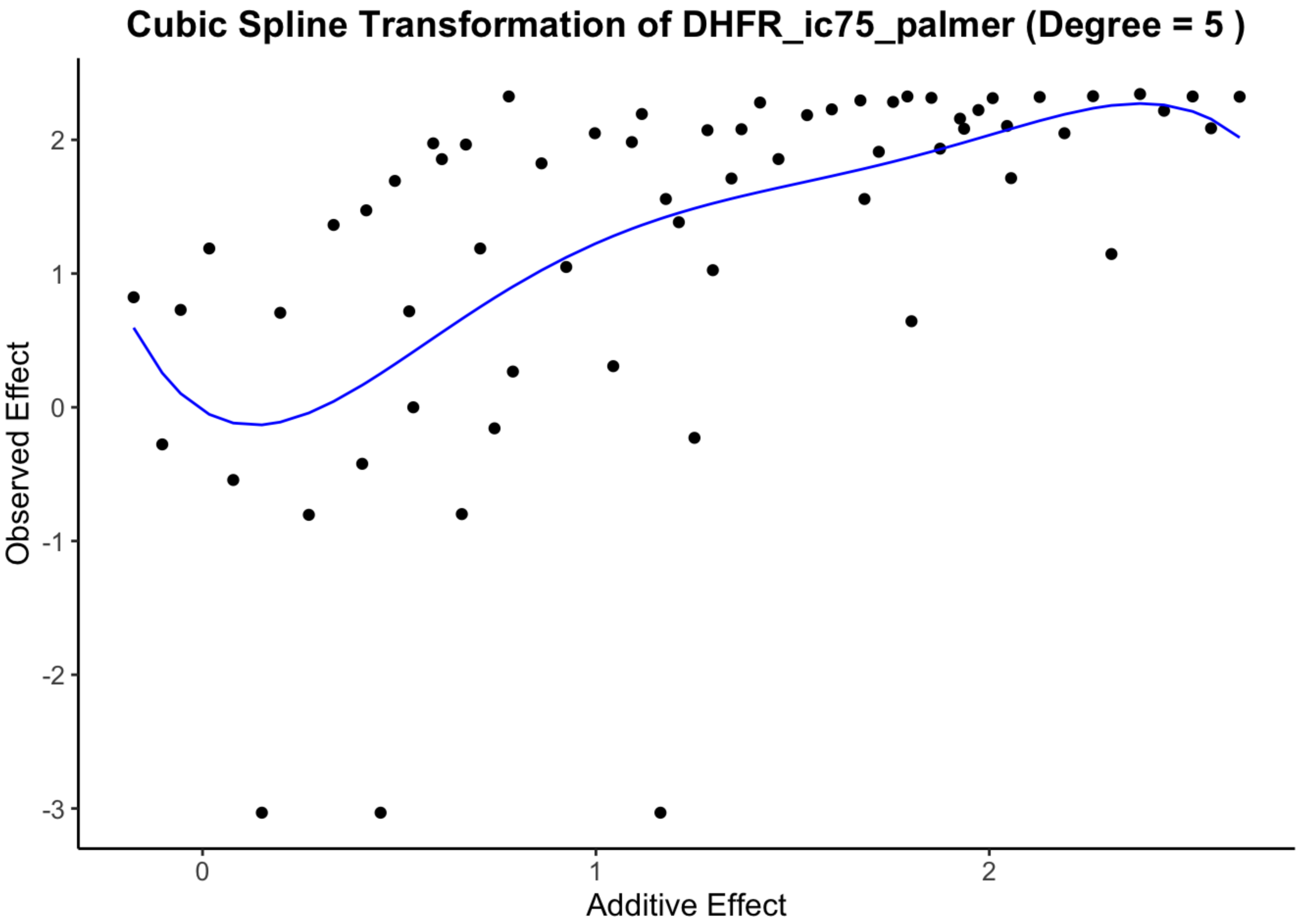
Cubic Spline Transformation of DHFR_ic75_palmer (Degree = 5)
Observed Effect
Additive Effect
2
1
0
-1
-2
-3
0
1
2

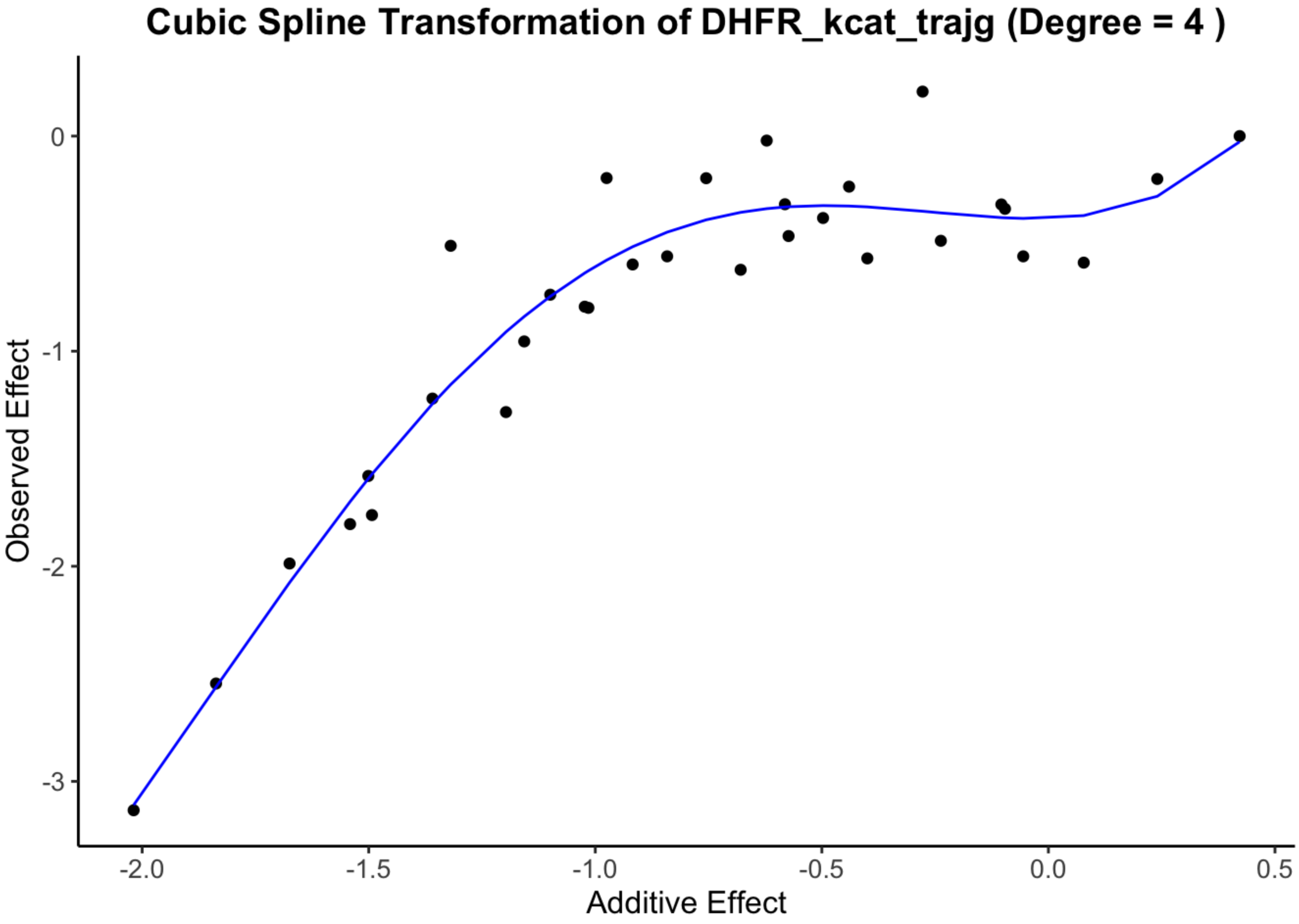
Cubic Spline Transformation of DHFR_kcat_trajg (Degree = 4)
Observed Effect
Additive Effect
0
-1
-2
-3
-2.0
-1.5
-1.0
-0.5
0.0
0.5

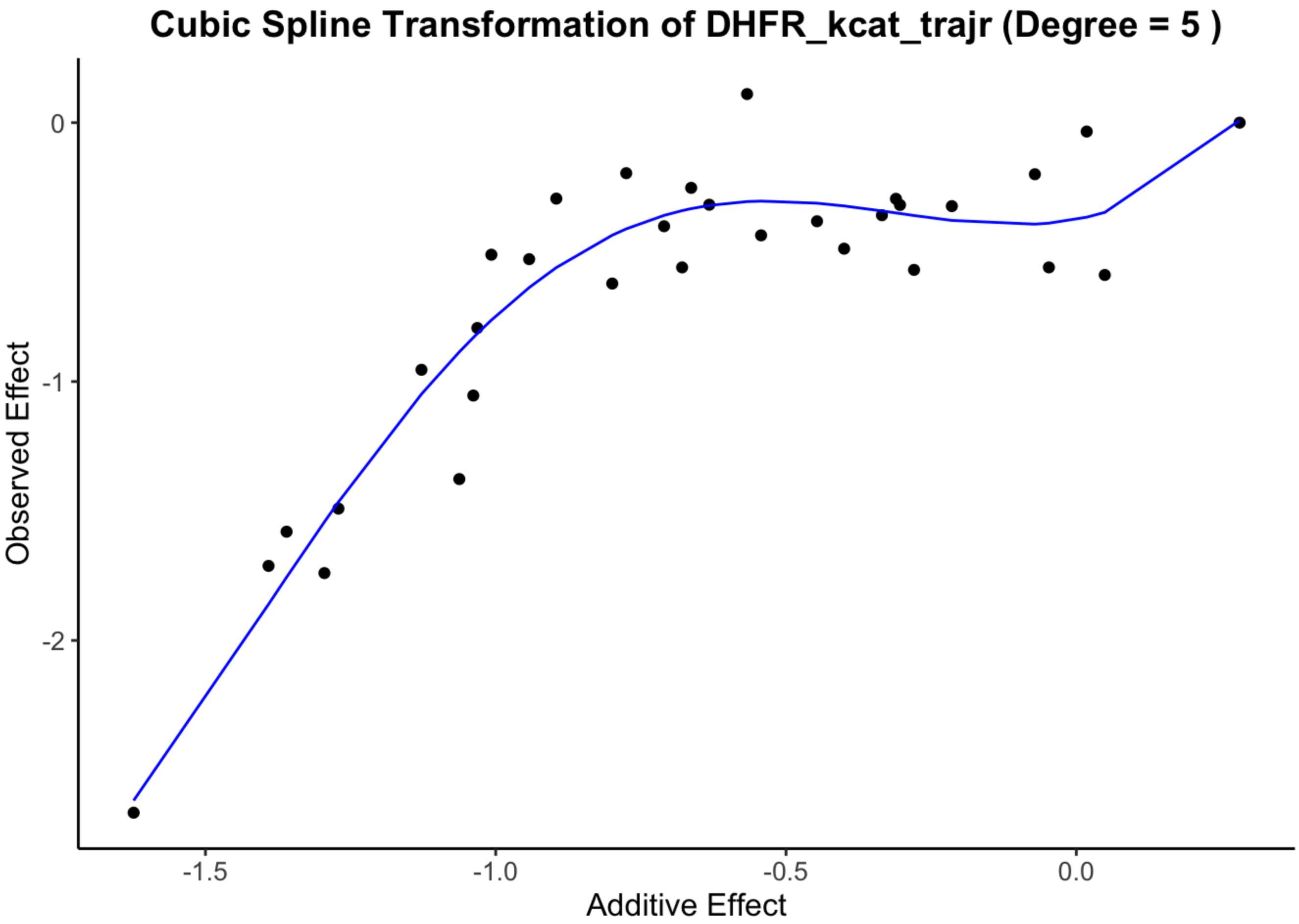
Cubic Spline Transformation of DHFR_kcat_trajr (Degree = 5)
Observed Effect
0
-1
-2
-1.5
-1.0
-0.5
0.0
Additive Effect

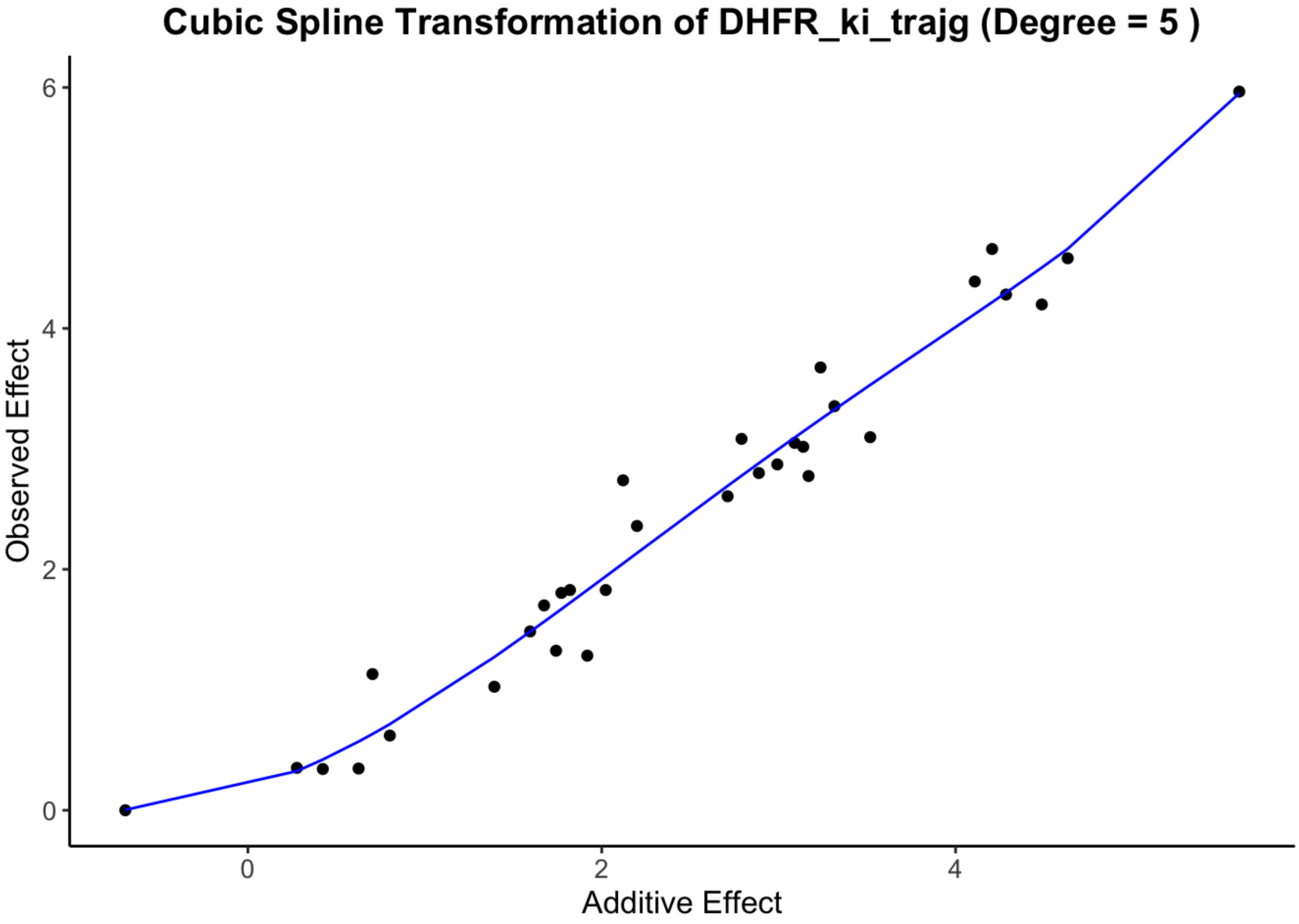
Cubic Spline Transformation of DHFR_ki_trajg (Degree = 5)
Observed Effect
6
4
2
0
0
2
4
Additive Effect

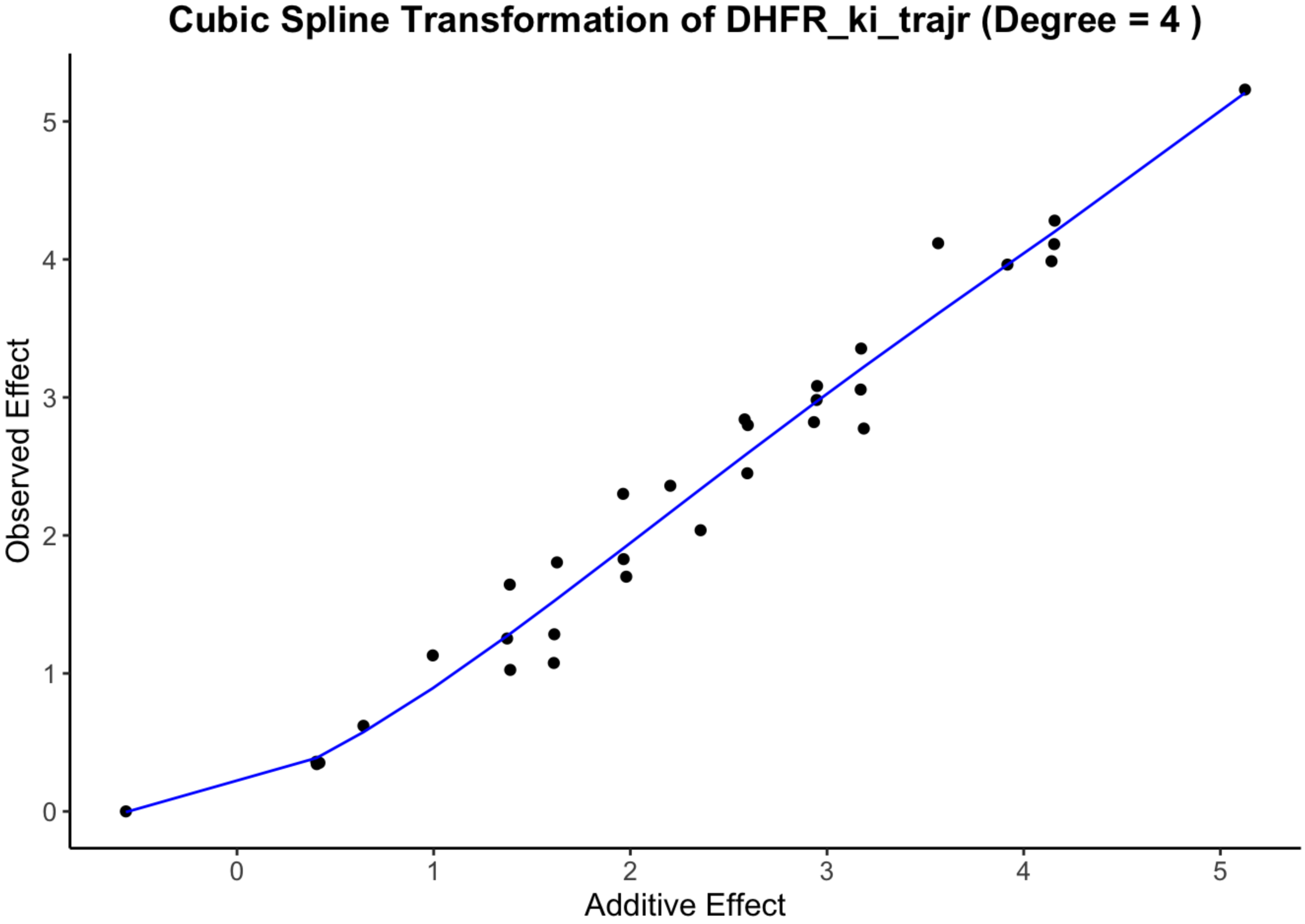
Cubic Spline Transformation of DHFR_ki_trajr (Degree = 4)
Observed Effect
Additive Effect
0
1
2
3
4
5
0
1
2
3
4
5

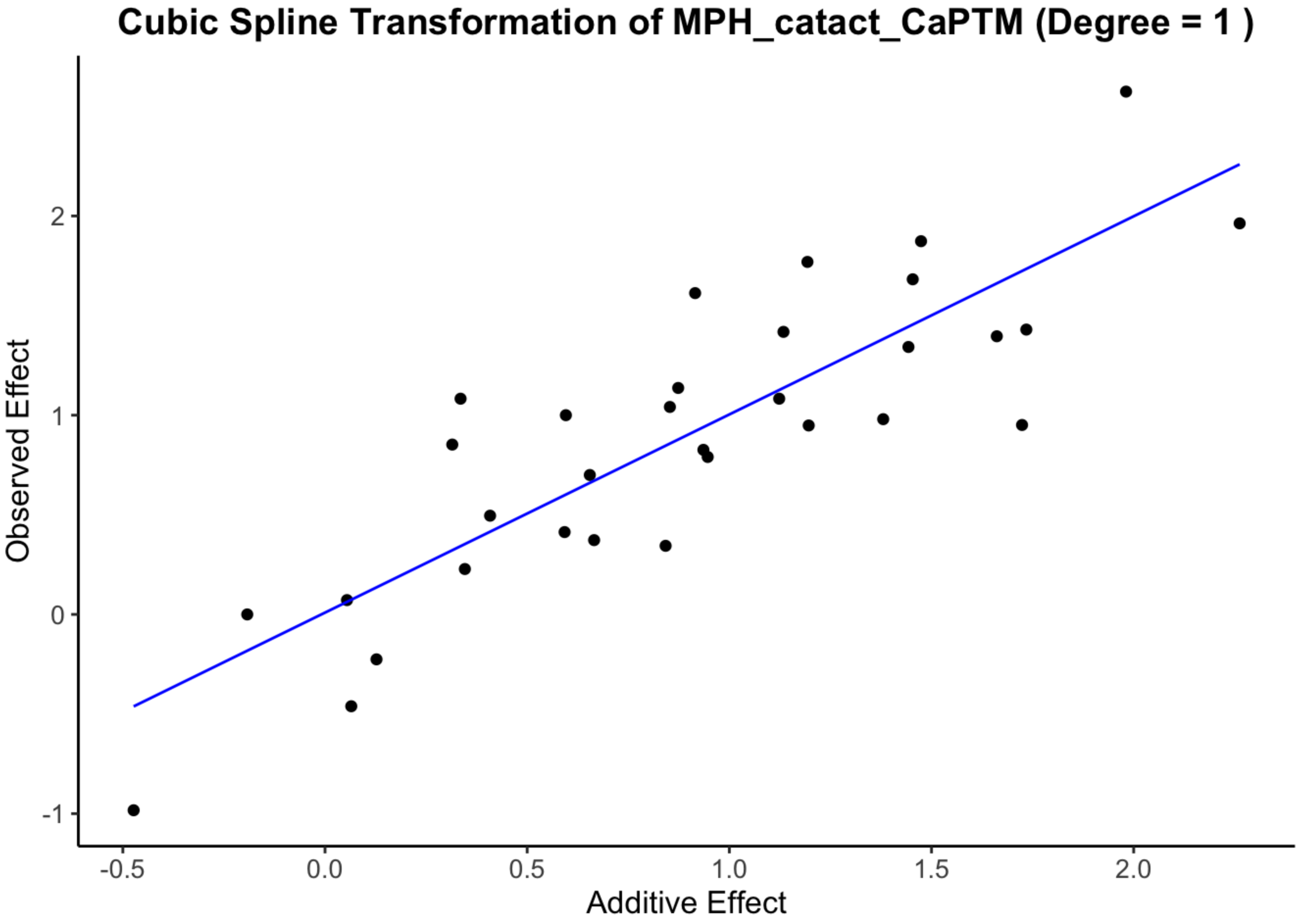
Cubic Spline Transformation of MPH_catact_CaPTM (Degree = 1)
Observed Effect
Additive Effect
-1
0
1
2
-0.5
0.0
0.5
1.0
1.5
2.0

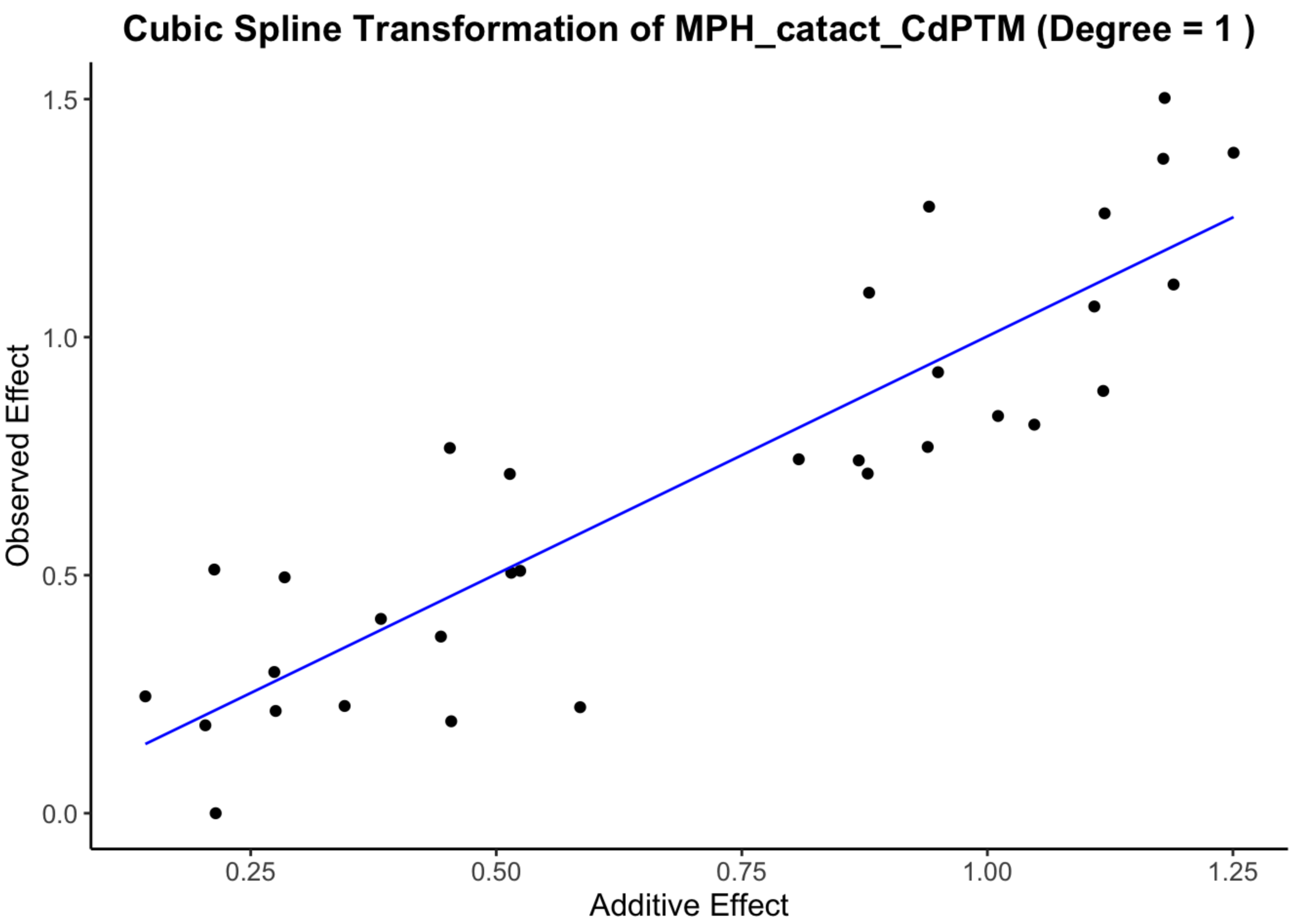
Cubic Spline Transformation of MPH_catact_CdPTM (Degree = 1)
Observed Effect
Additive Effect
0.0
0.5
1.0
1.5
0.25
0.50
0.75
1.00
1.25

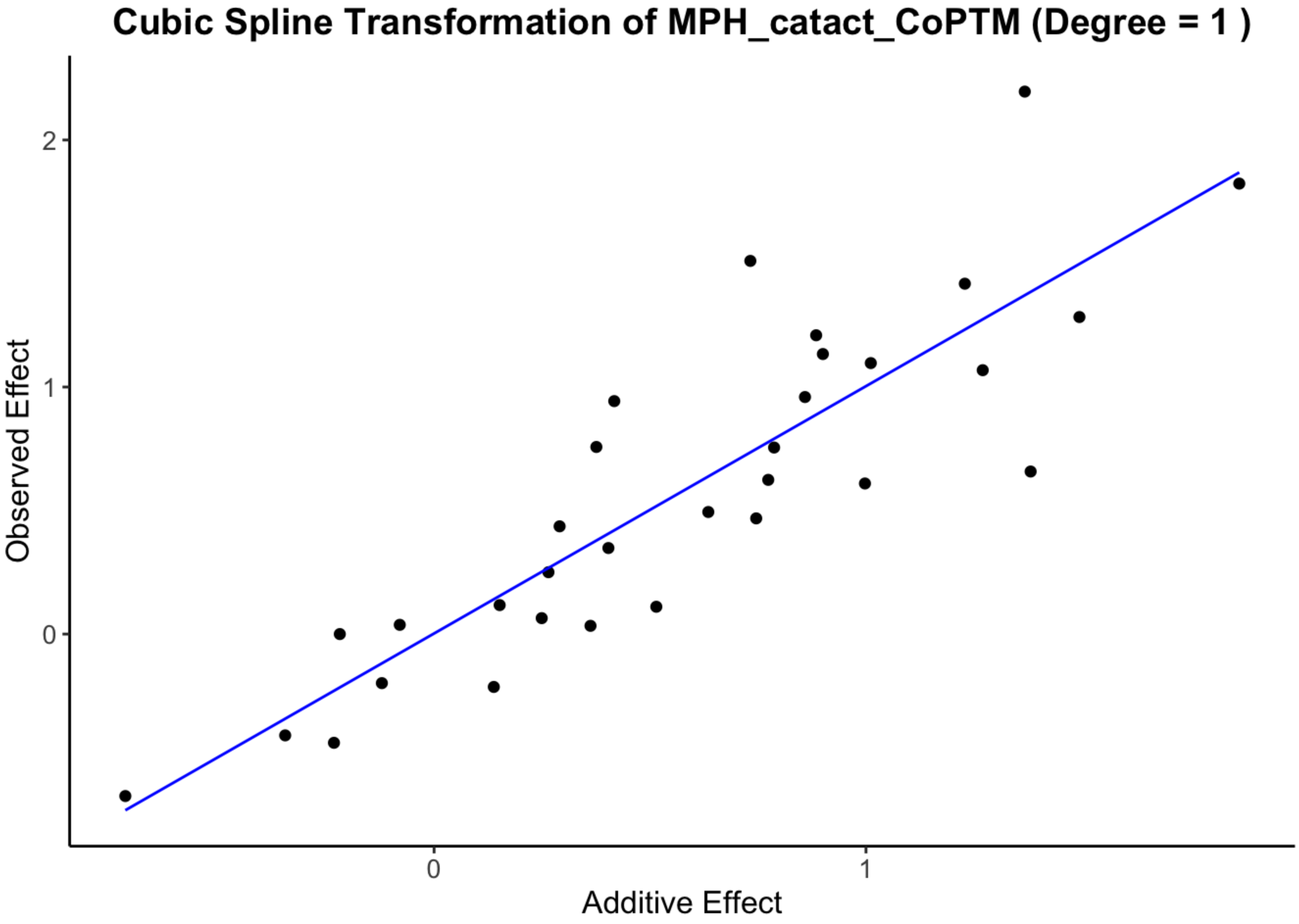
Cubic Spline Transformation of MPH_catact_CoPTM (Degree = 1)
Observed Effect
Additive Effect
0
1
2
0
1

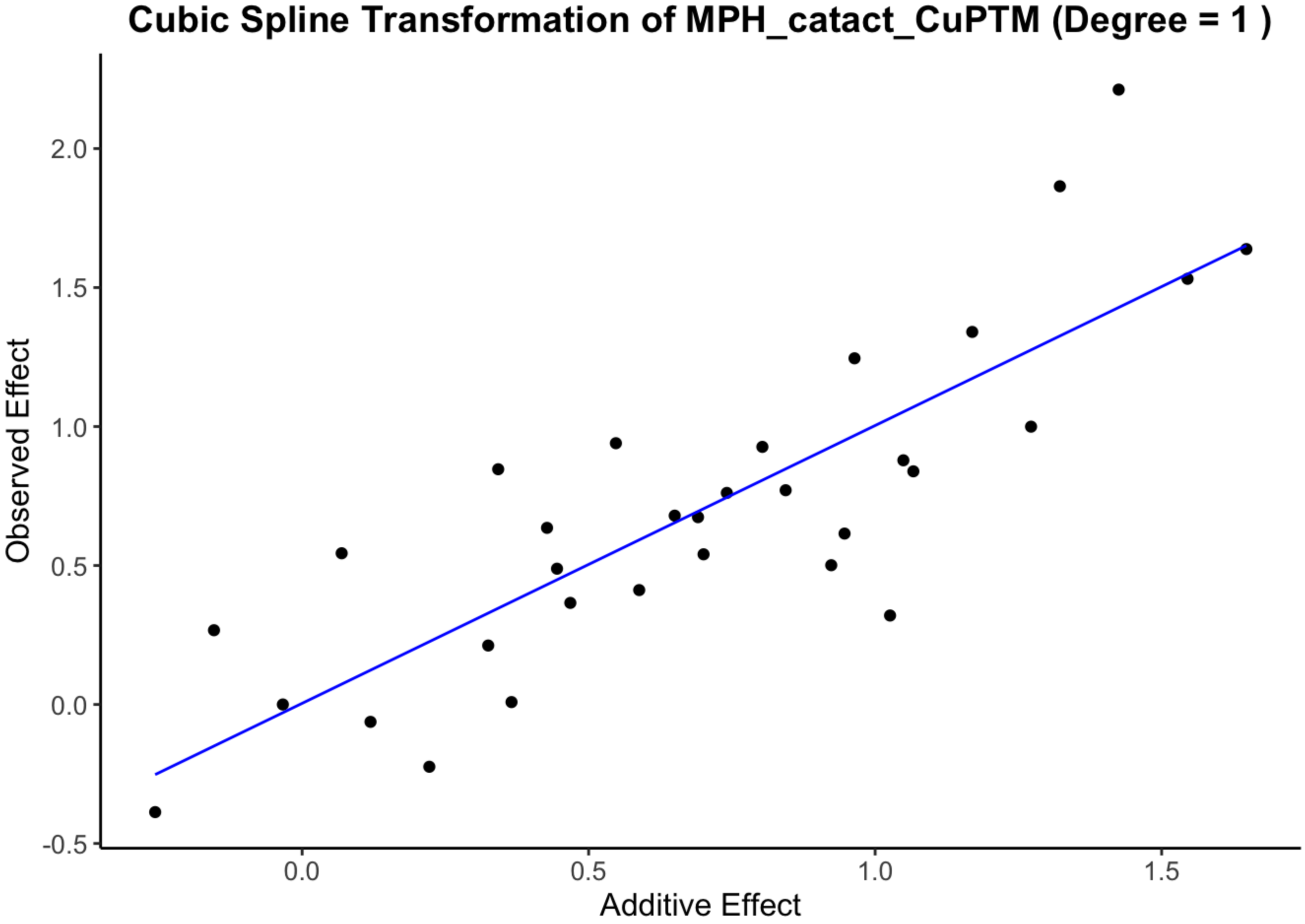
Cubic Spline Transformation of MPH_catact_CuPTM (Degree = 1)
Observed Effect
-0.5
0.0
0.5
1.0
1.5
2.0
Additive Effect
0.0
0.5
1.0
1.5

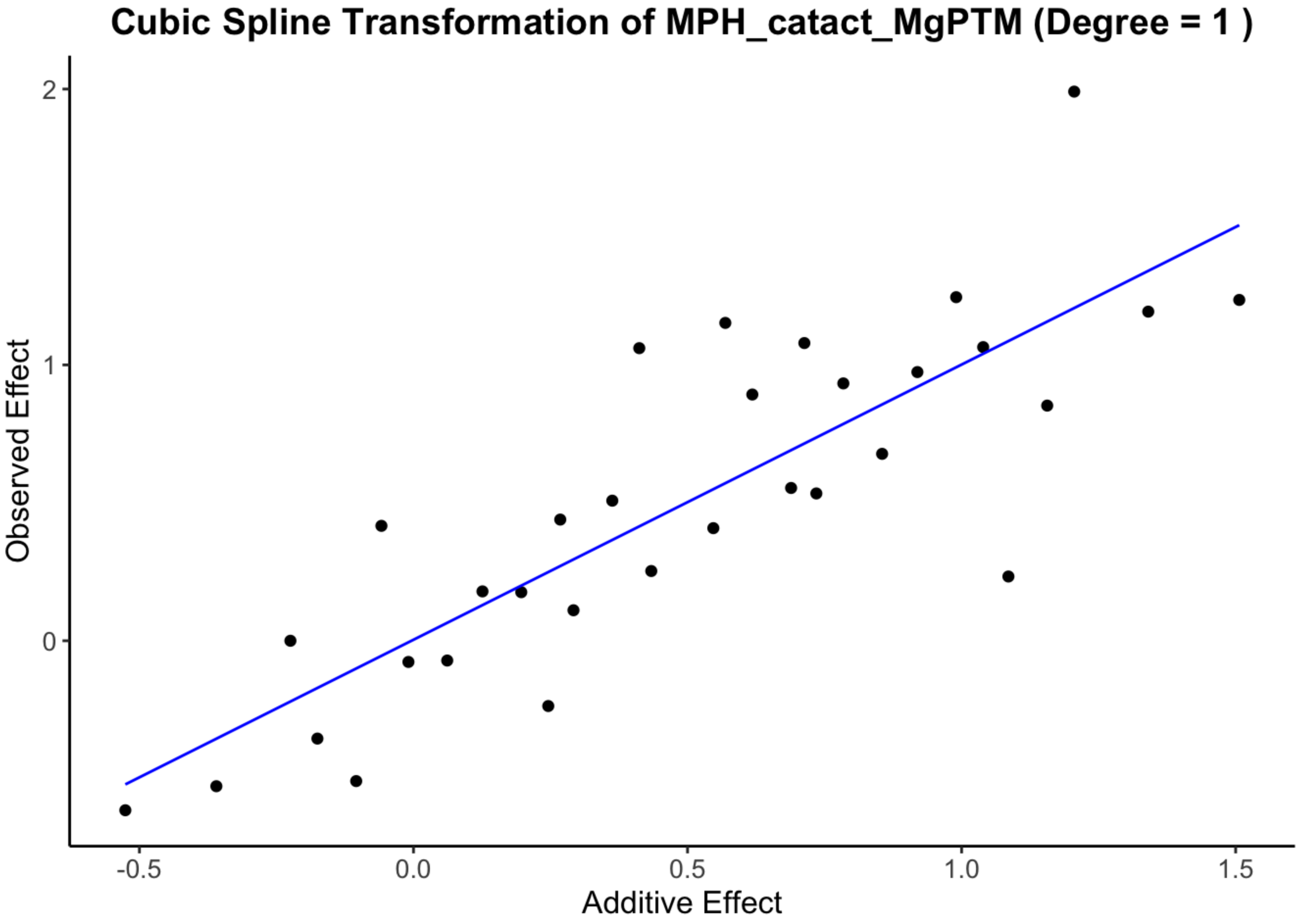
Cubic Spline Transformation of MPH_catact_MgPTM (Degree = 1)
Observed Effect
0
1
2
Additive Effect
-0.5
0.0
0.5
1.0
1.5

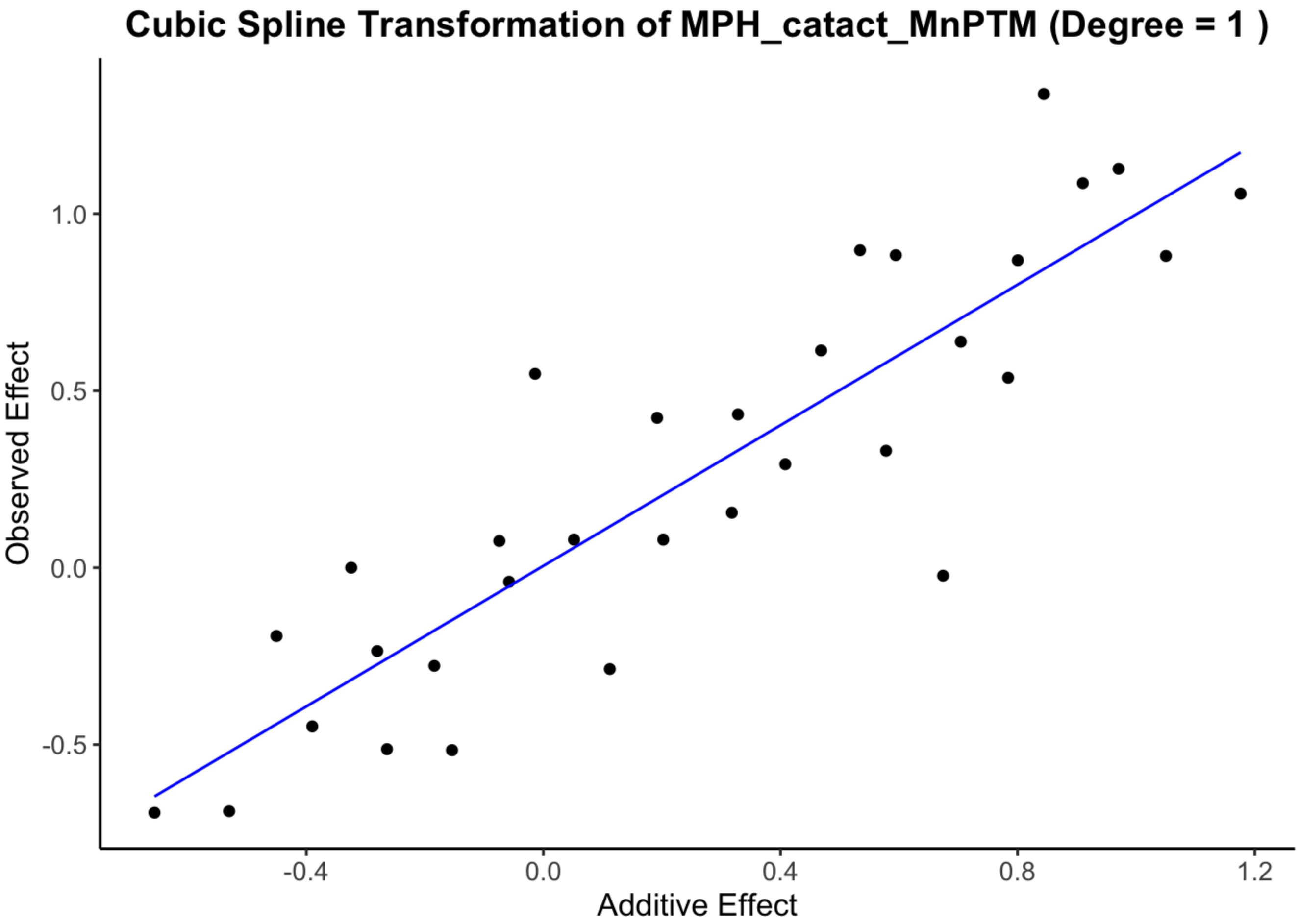
Cubic Spline Transformation of MPH_catact_MnPTM (Degree = 1)
Observed Effect
Additive Effect
1.0
0.5
0.0
-0.5
-0.4
0.0
0.4
0.8
1.2

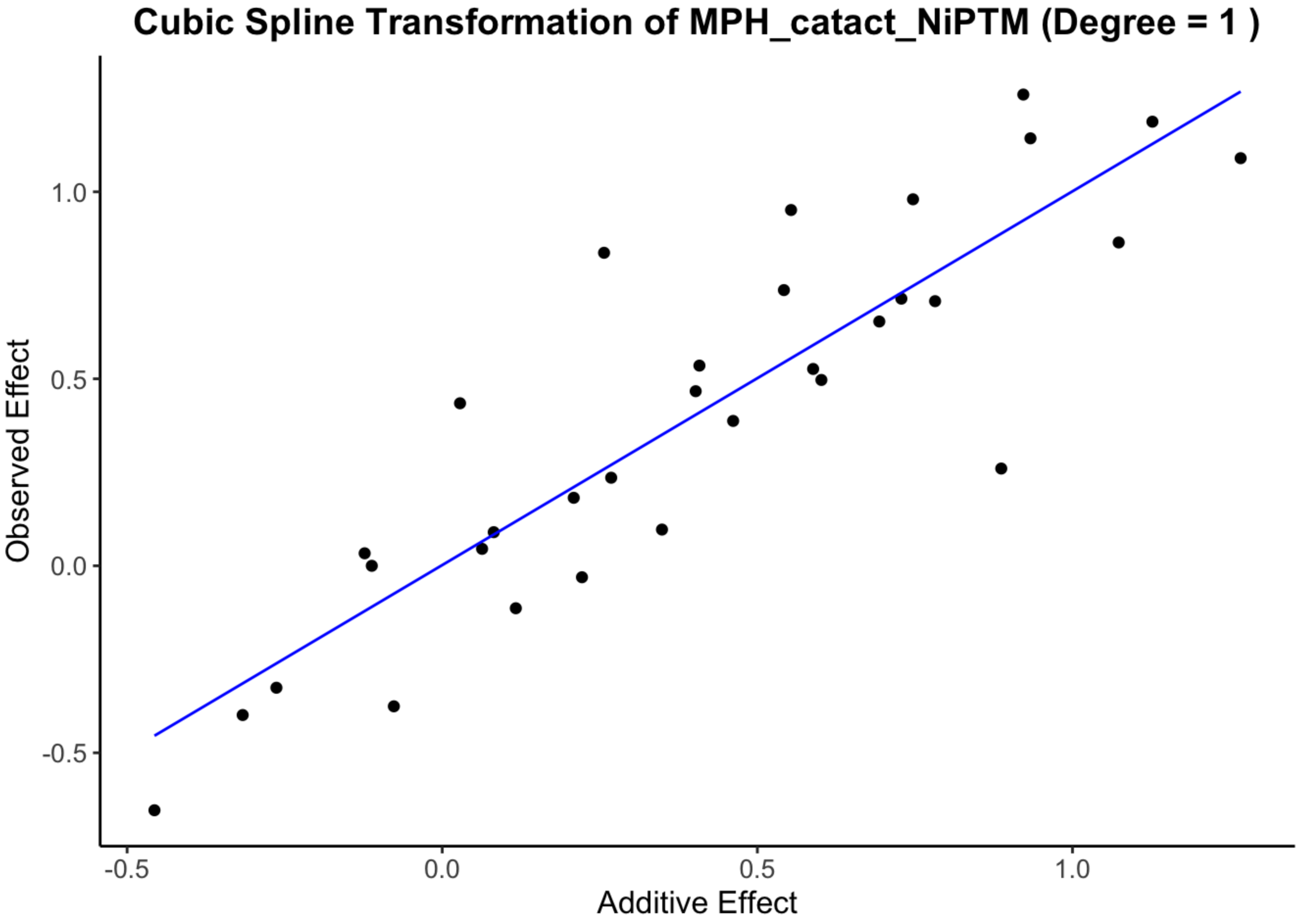
Cubic Spline Transformation of MPH_catact_NiPTM (Degree = 1)
Observed Effect
Additive Effect
1.0
0.5
0.0
-0.5
-0.5
0.0
0.5
1.0

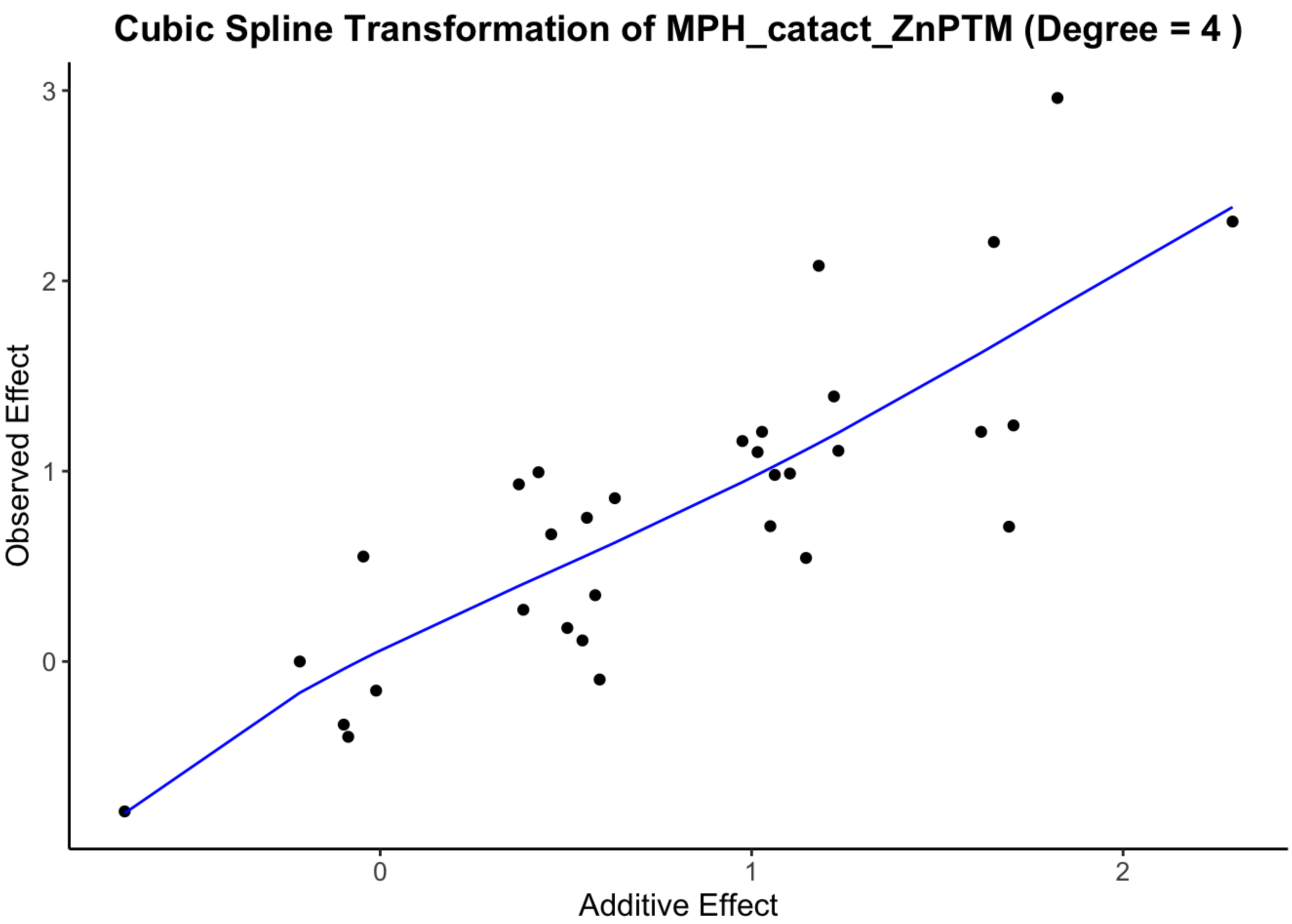
Cubic Spline Transformation of MPH_catact_ZnPTM (Degree = 4)
Observed Effect
Additive Effect
0
1
2
3
0
1
2

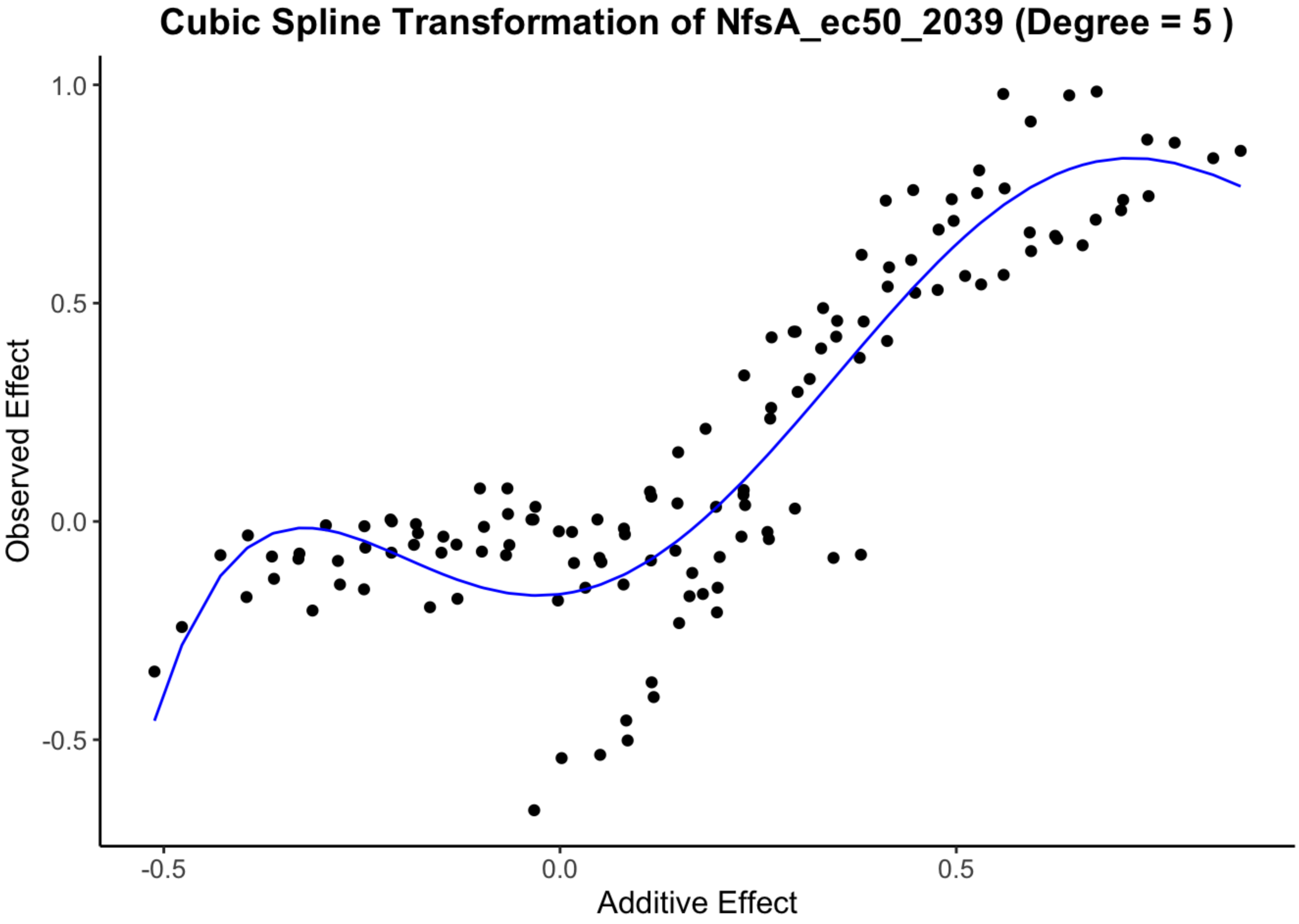
Cubic Spline Transformation of NfsA_ec50_2039 (Degree = 5)
Observed Effect
Additive Effect
1.0
0.5
0.0
-0.5
-0.5
0.0
0.5

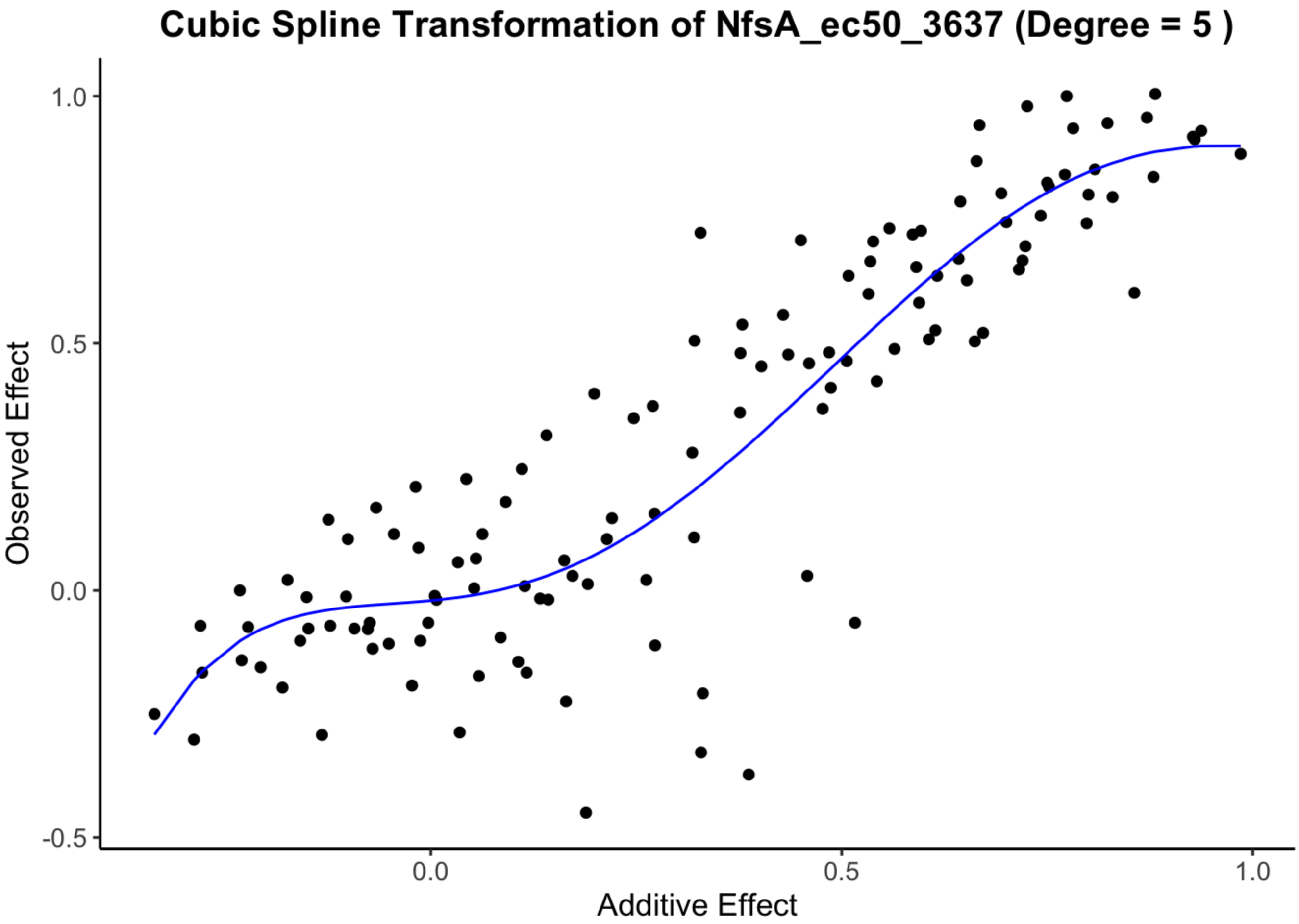
Cubic Spline Transformation of NfsA_ec50_3637 (Degree = 5)
Observed Effect
Additive Effect
1.0
0.5
0.0
-0.5
0.0
0.5
1.0

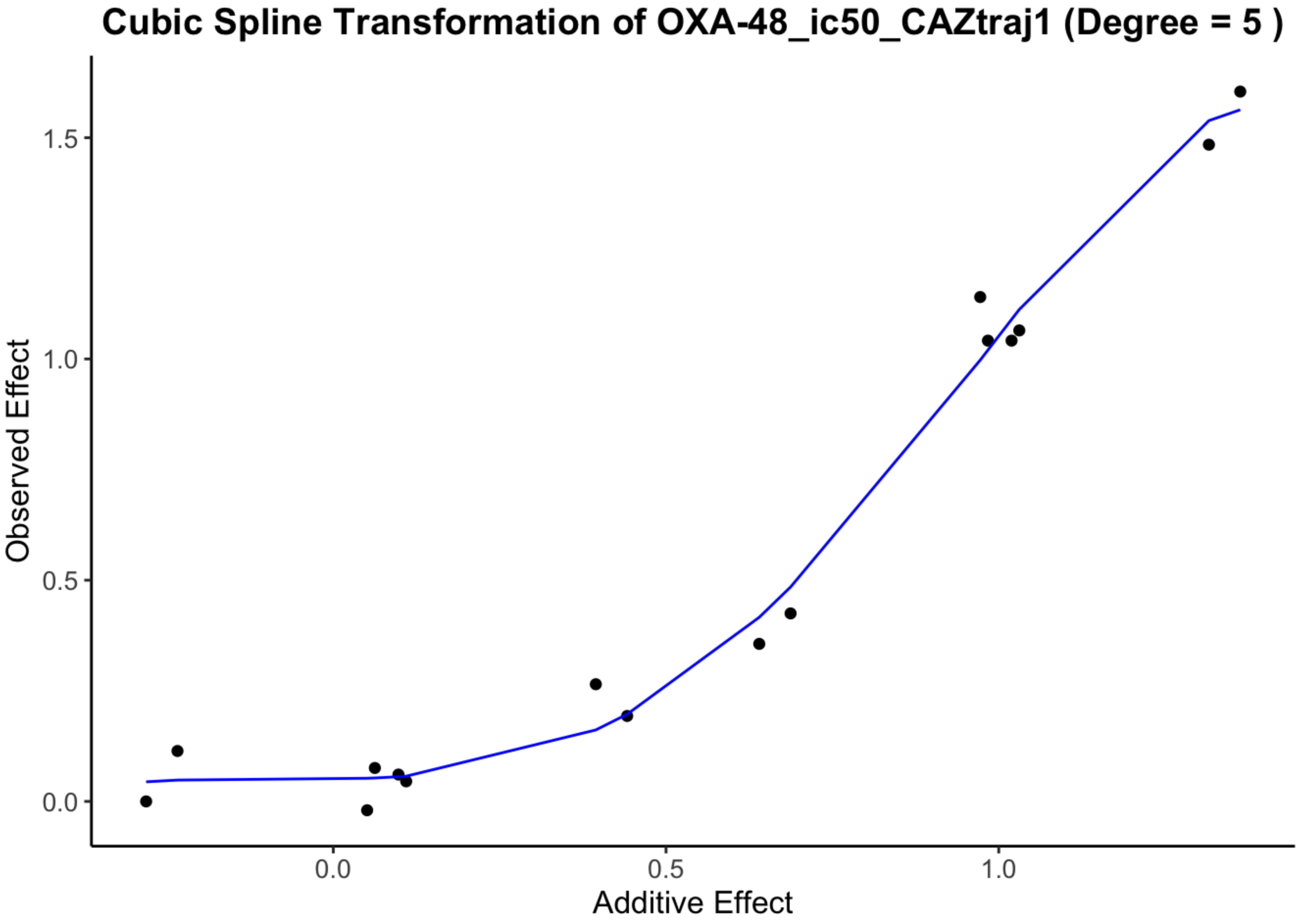
Cubic Spline Transformation of OXA-48_ic50_CAZtraj1 (Degree = 5)
Observed Effect
Additive Effect
1.5
1.0
0.5
0.0
0.0
0.5
1.0

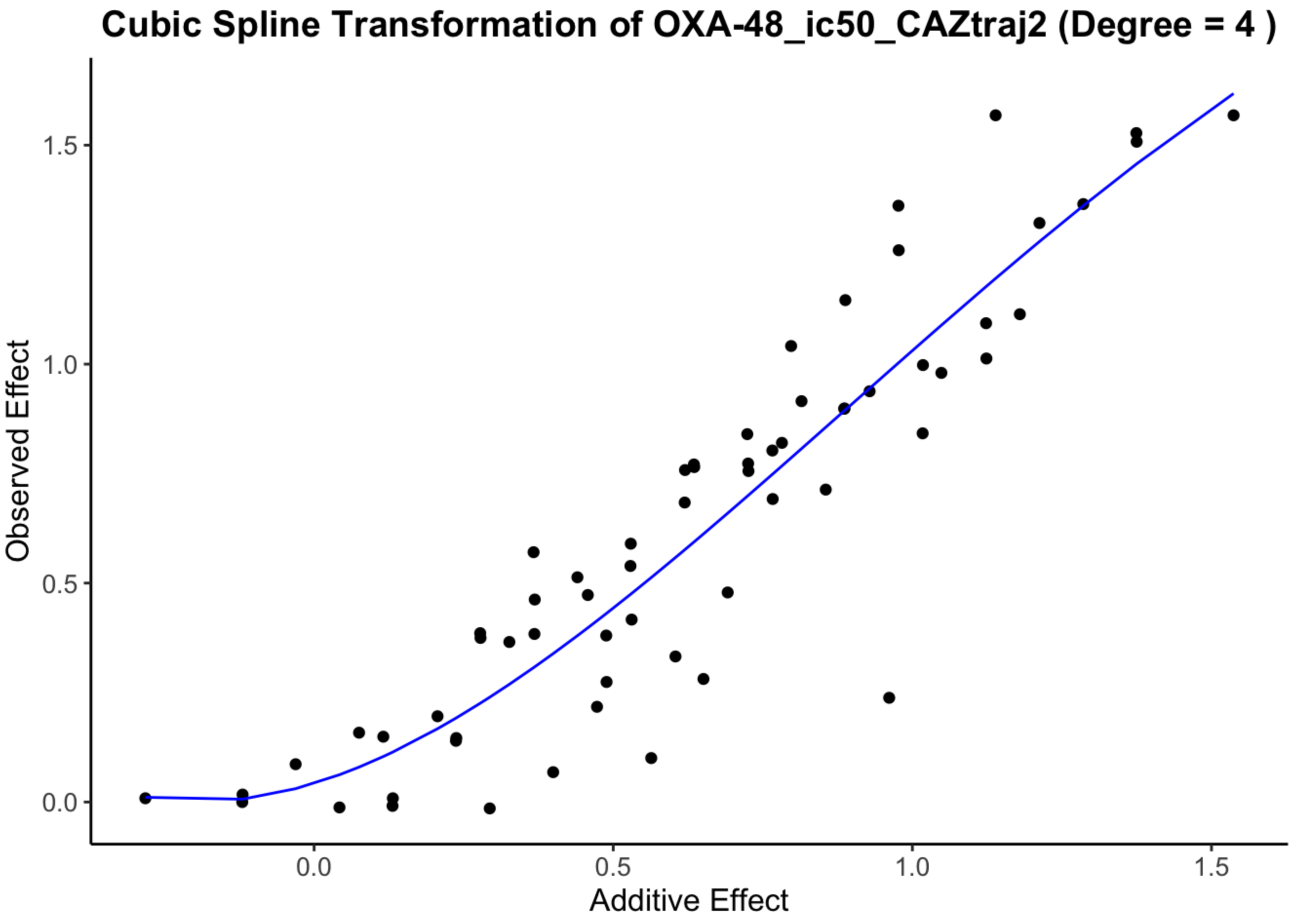

Cubic Spline Transformation of OXA-48_ic50_CAZtraj2 (Degree = 4)
Observed Effect
Additive Effect
0.0
0.5
1.0
1.5

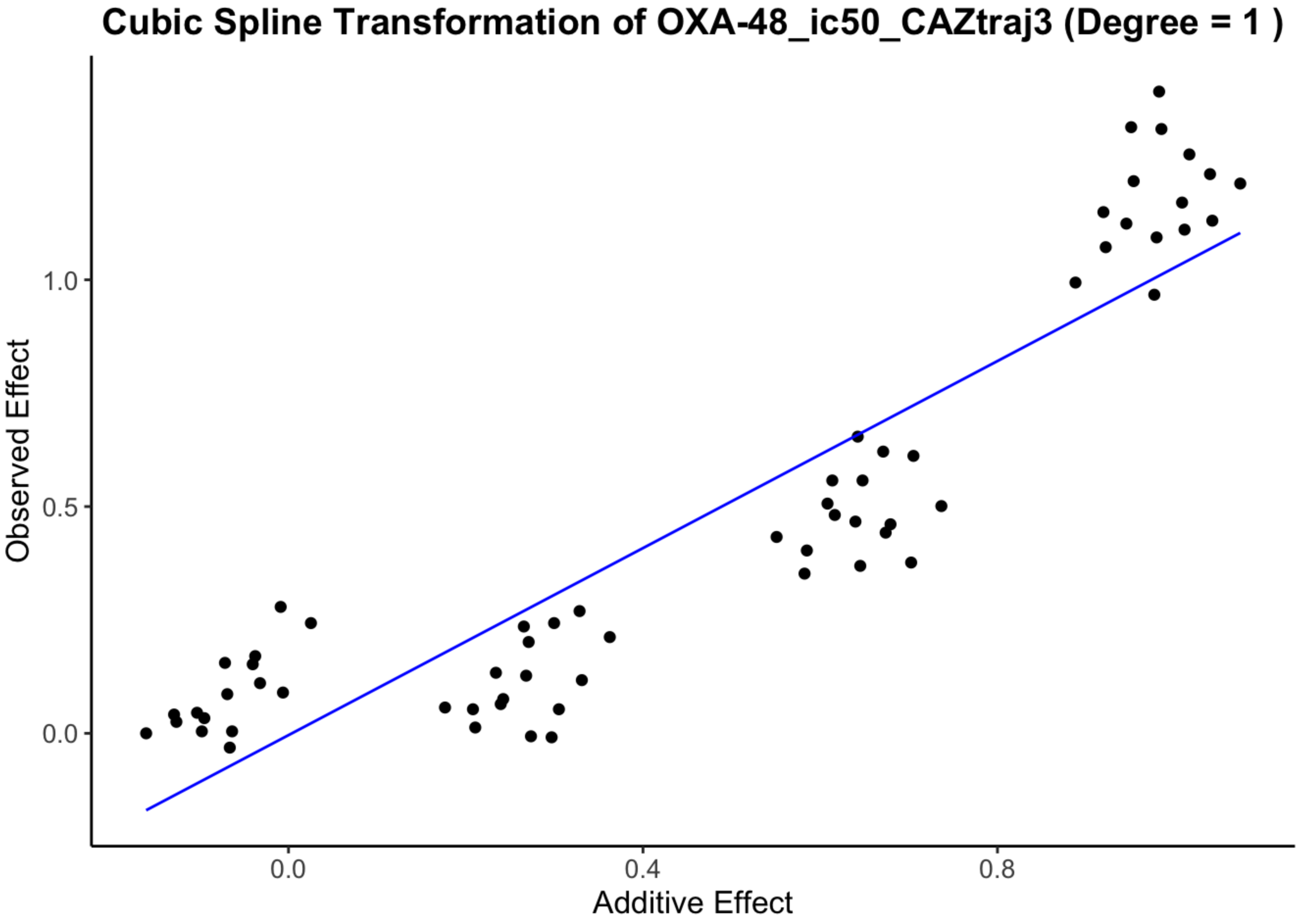

Cubic Spline Transformation of OXA-48_ic50_CAZtraj3 (Degree = 1)
Observed Effect
Additive Effect
0.0
0.5
1.0
0.4
0.8

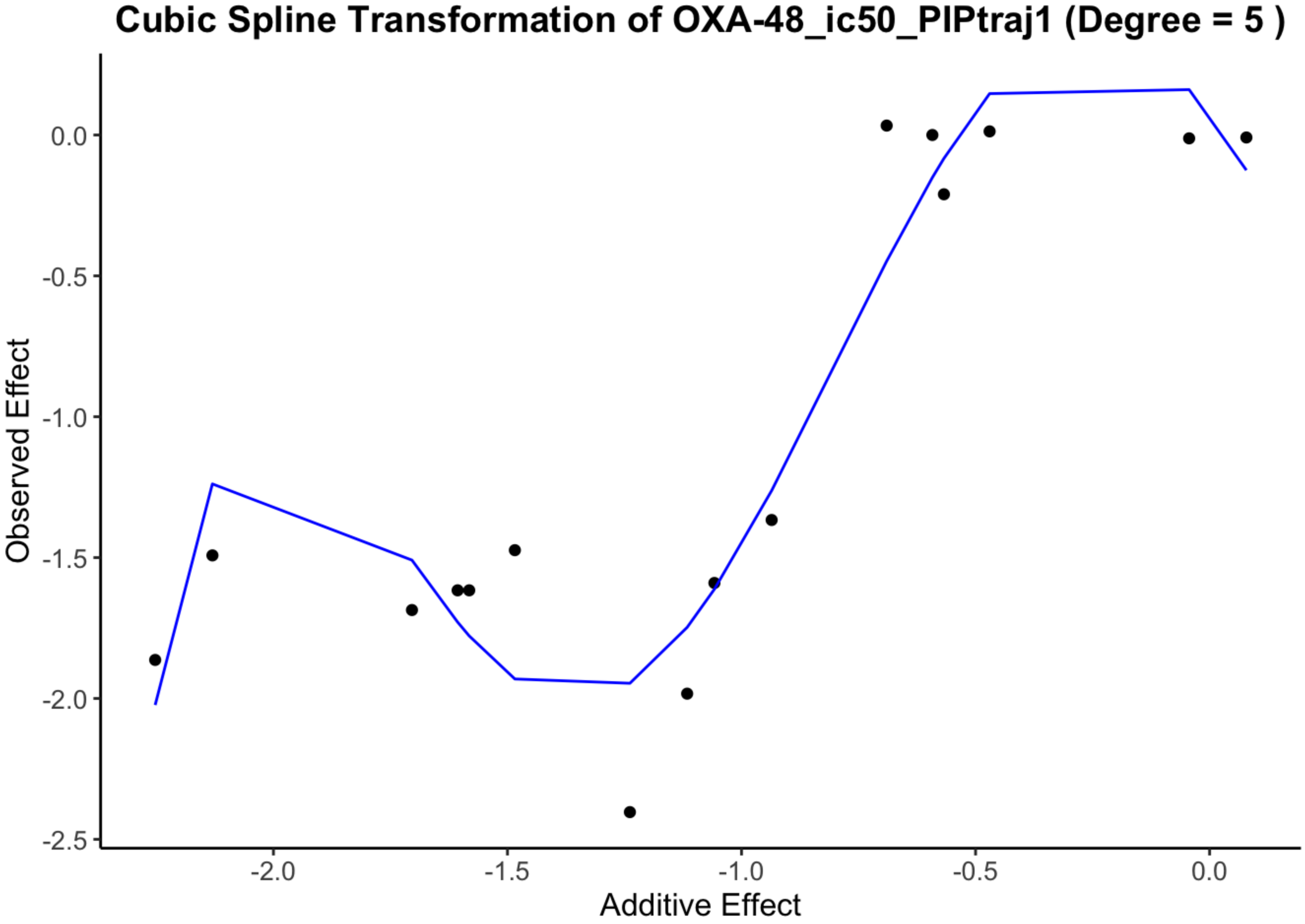
Cubic Spline Transformation of OXA-48_ic50_PIPtraj1 (Degree = 5)
Observed Effect
0.0
-0.5
-1.0
-1.5
-2.0
-2.5
-2.0
-1.5
-1.0
-0.5
0.0
Additive Effect

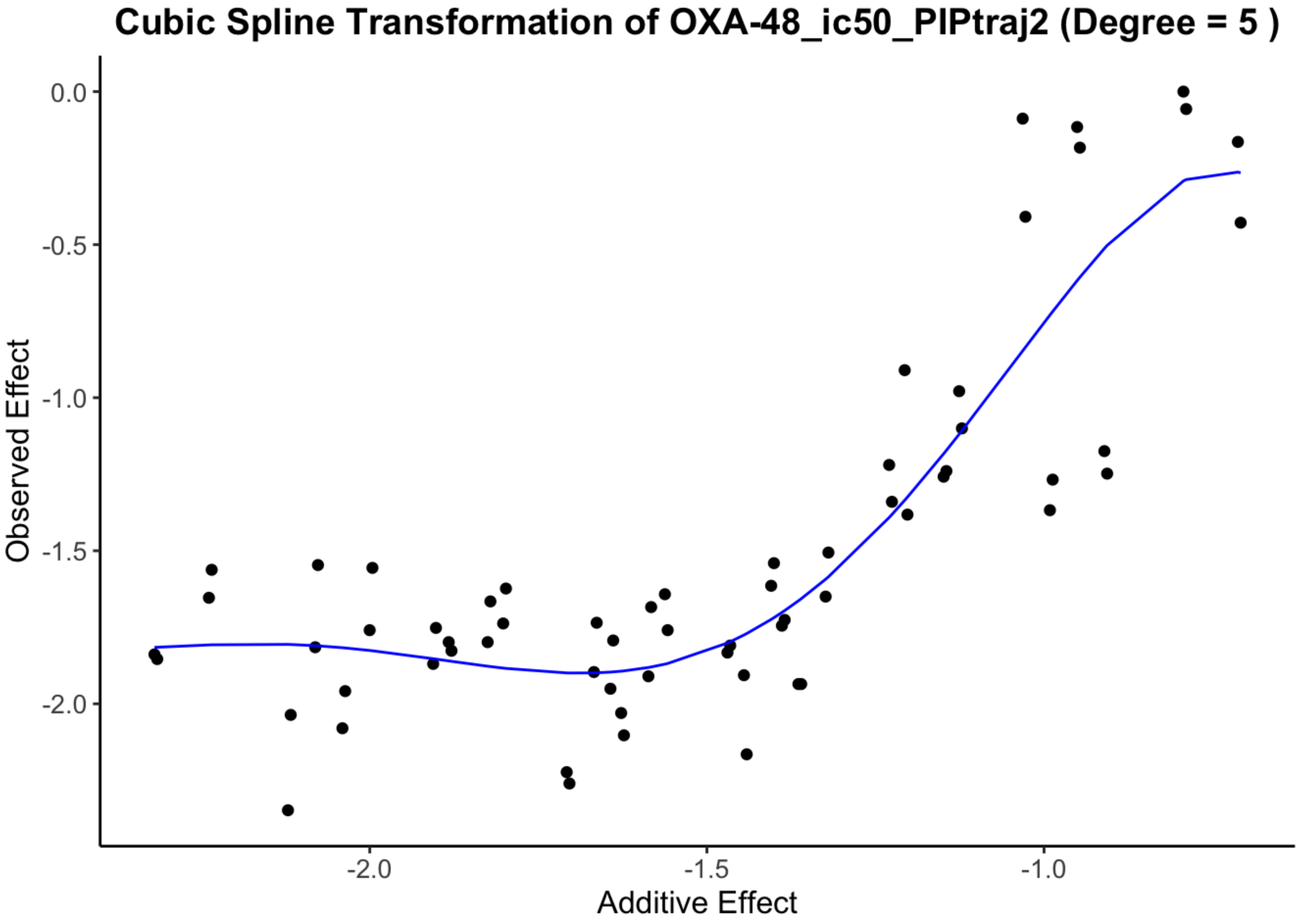
Cubic Spline Transformation of OXA-48_ic50_PIPtraj2 (Degree = 5)
Observed Effect
0.0
-0.5
-1.0
-1.5
-2.0
-2.0
-1.5
-1.0
Additive Effect

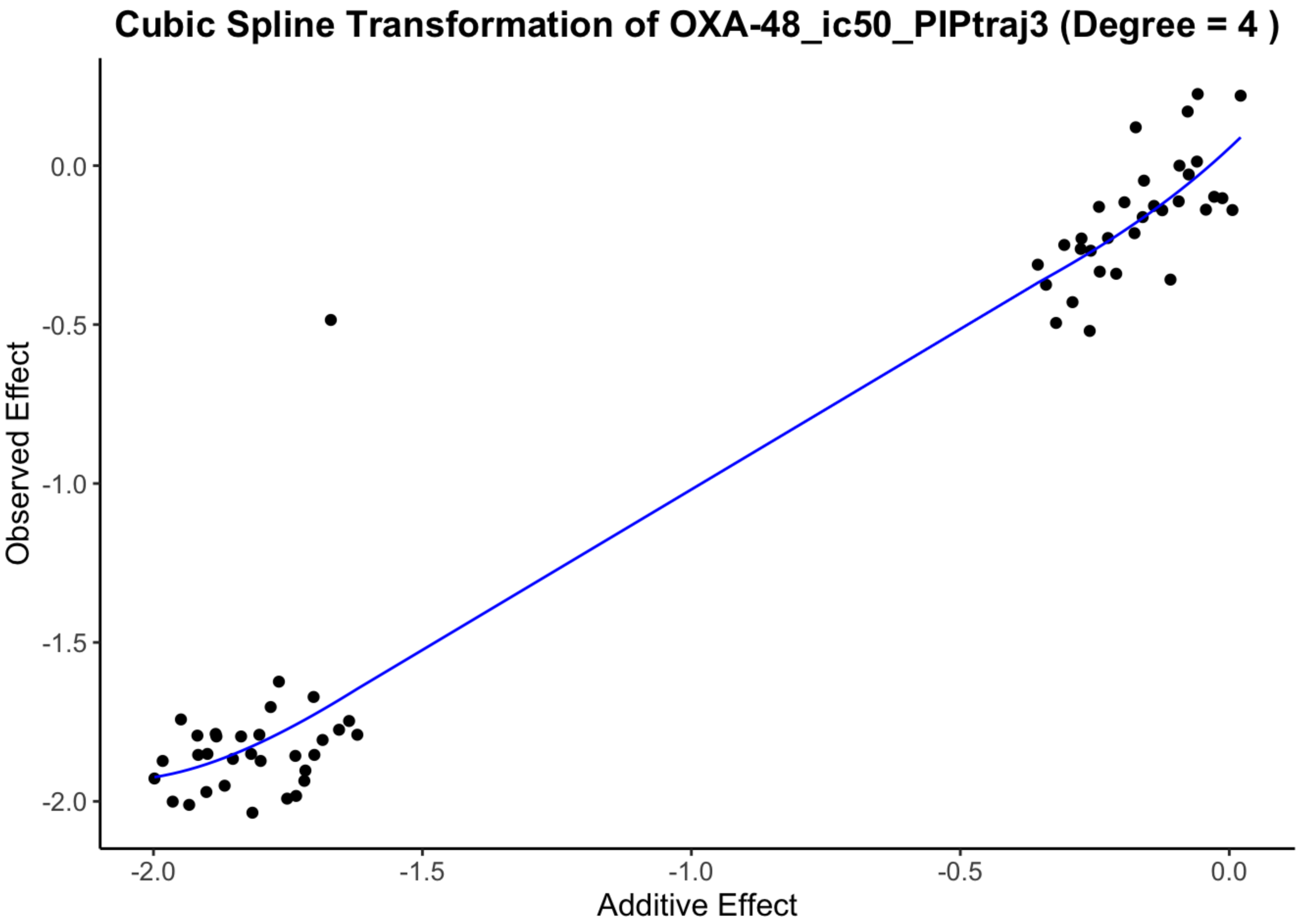
Cubic Spline Transformation of OXA-48_ic50_PIPtraj3 (Degree = 4)
Observed Effect
0.0
-0.5
-1.0
-1.5
-2.0
-2.0
-1.5
-1.0
-0.5
0.0
Additive Effect

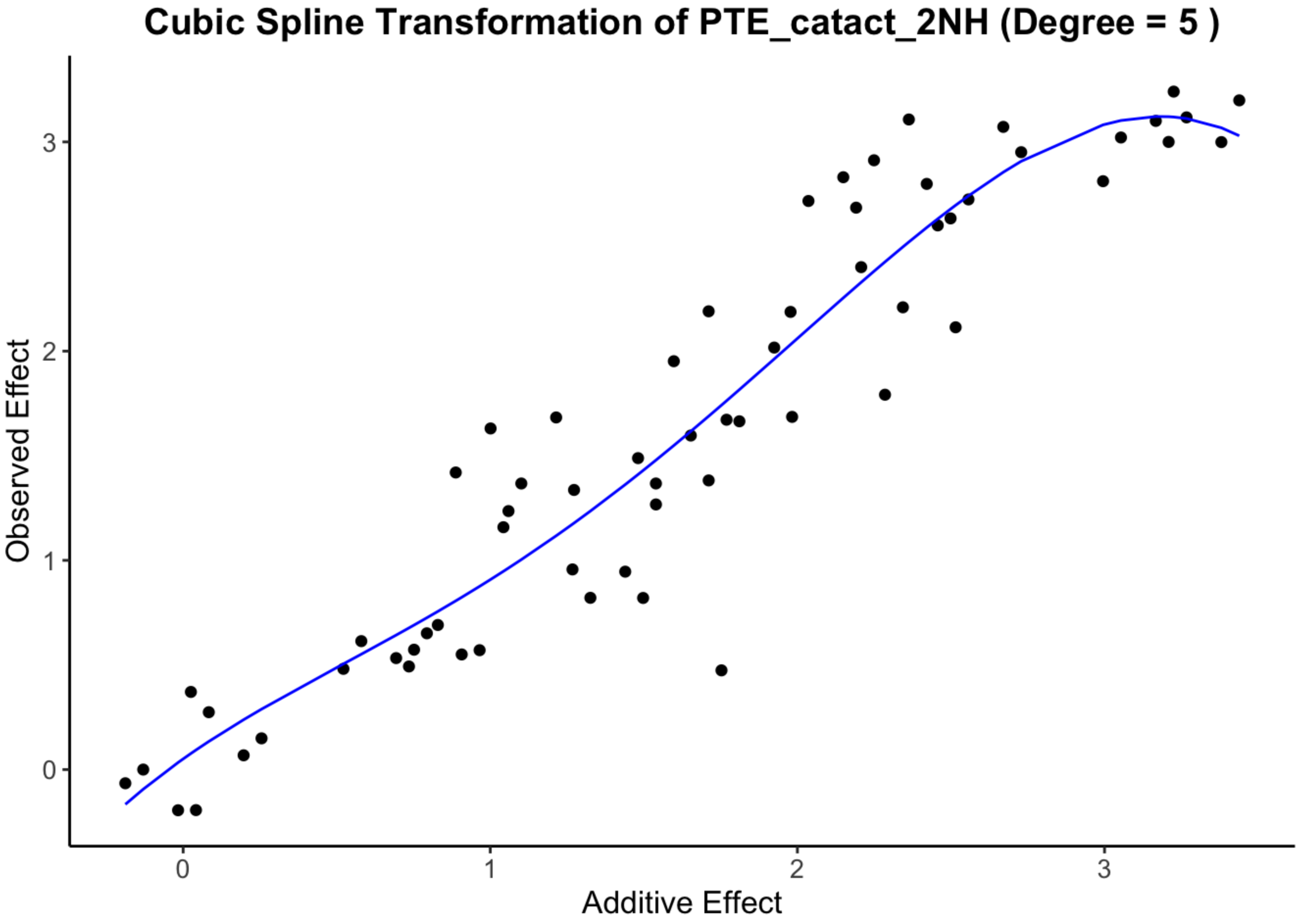
Cubic Spline Transformation of PTE_catact_2NH (Degree = 5)
Observed Effect
3
2
1
0
0
1
2
3
Additive Effect

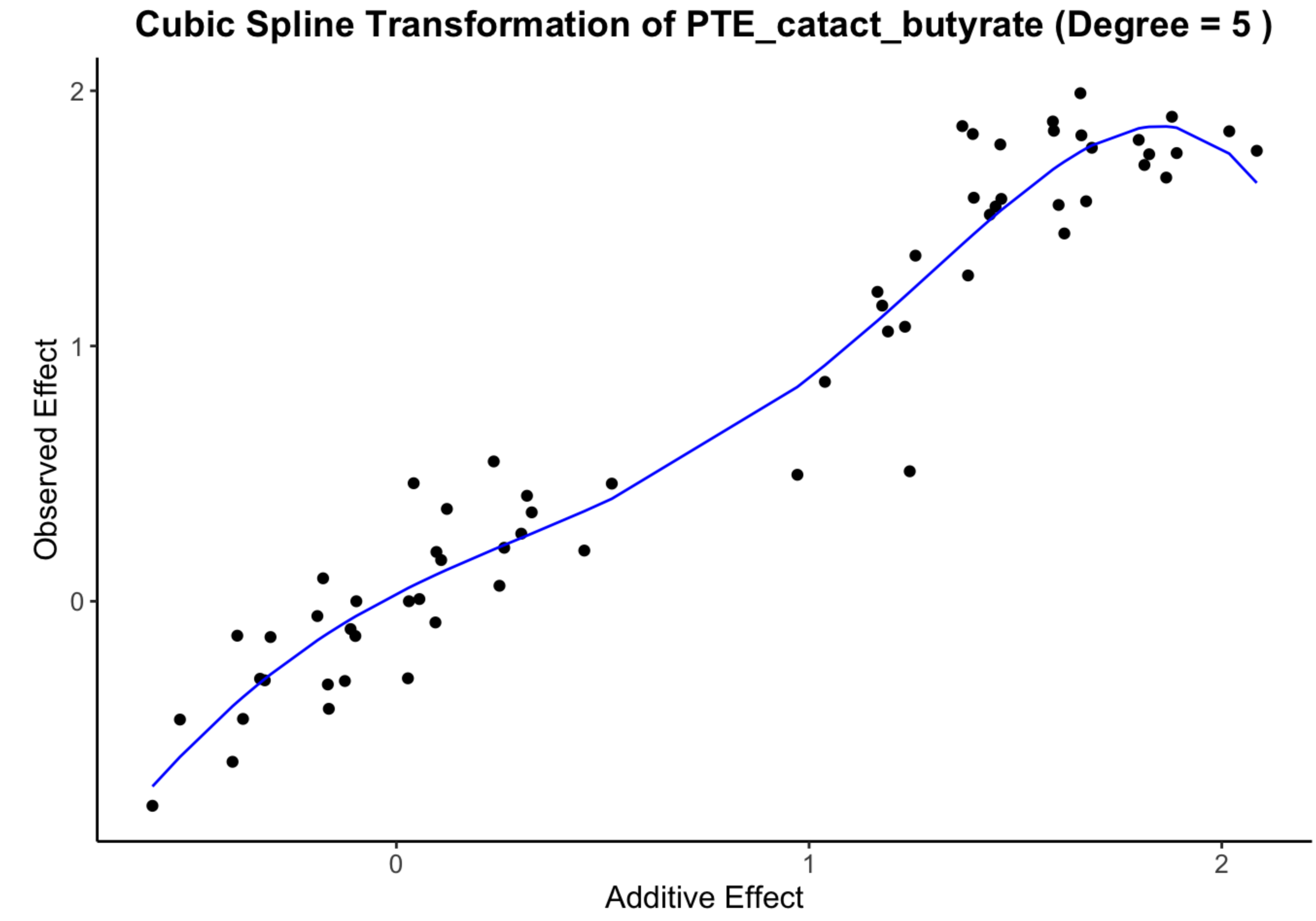
Cubic Spline Transformation of PTE_catact_butyrate (Degree = 5)
Observed Effect
Additive Effect
2
1
0
0
1
2

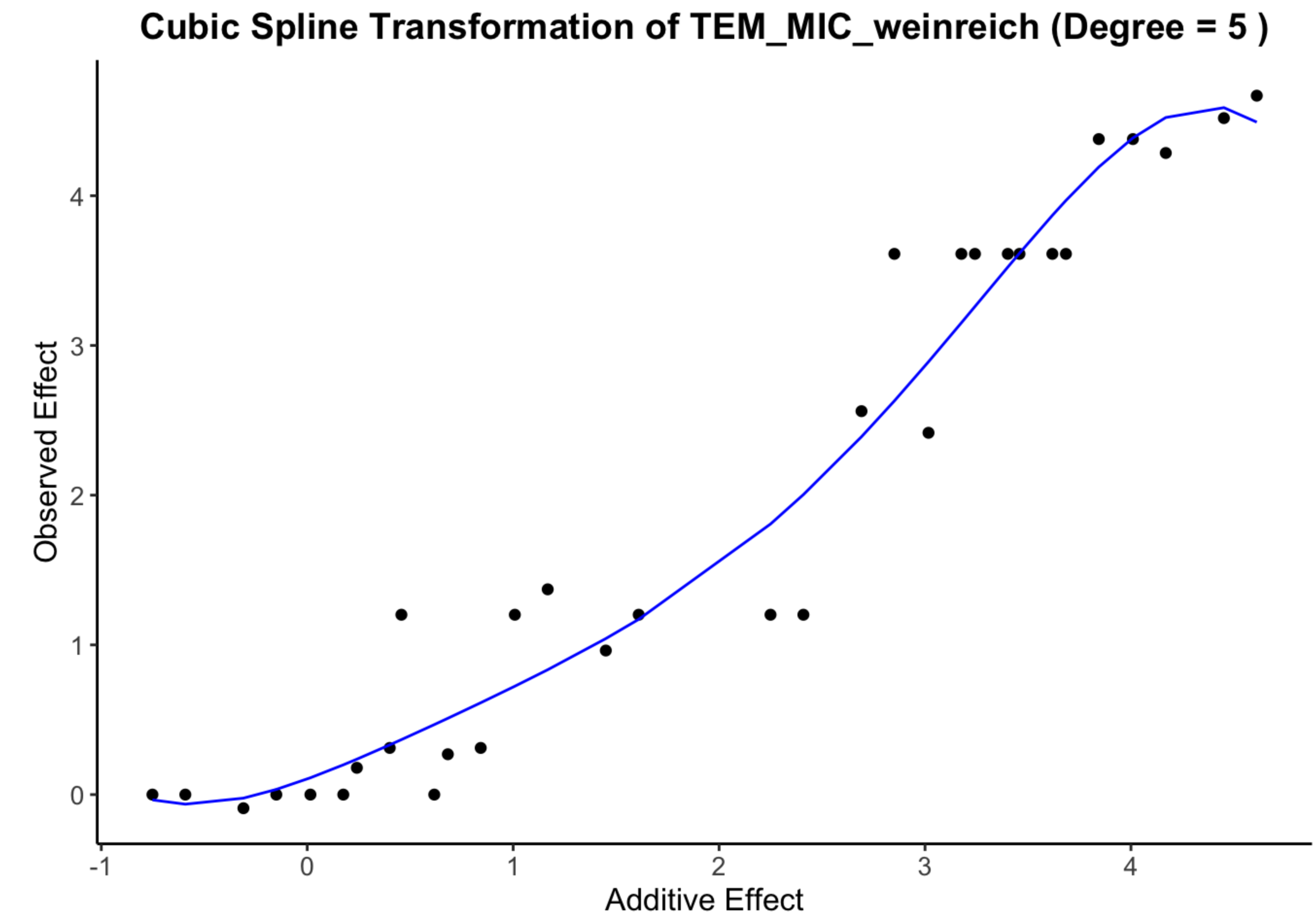
Cubic Spline Transformation of TEM_MIC_weinreich (Degree = 5)
Observed Effect
Additive Effect
4
3
2
1
0
-1
0
1
2
3
4

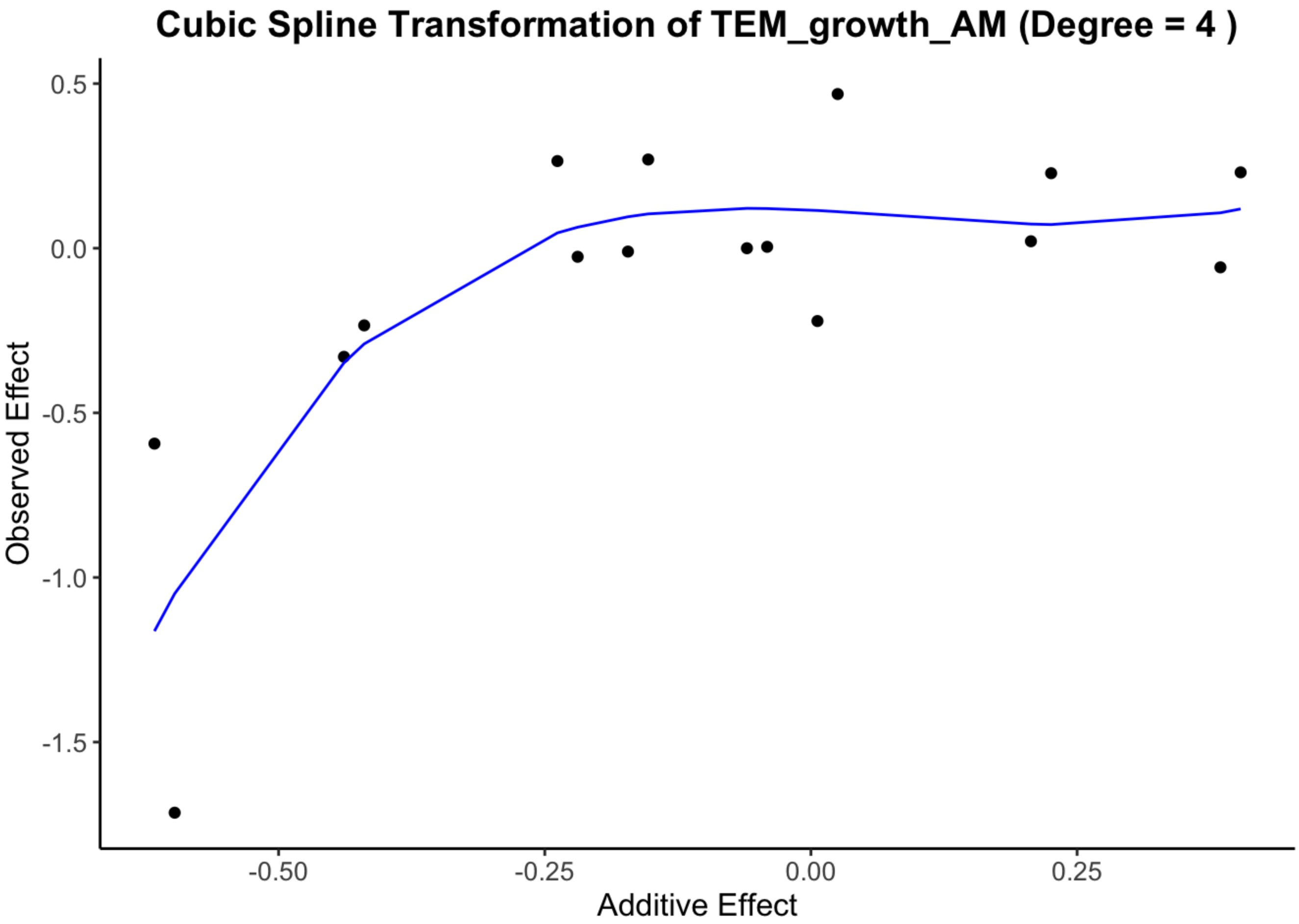
Cubic Spline Transformation of TEM_growth_AM (Degree = 4)
Observed Effect
0.5
0.0
-0.5
-1.0
-1.5
-0.50
-0.25
0.00
0.25
Additive Effect

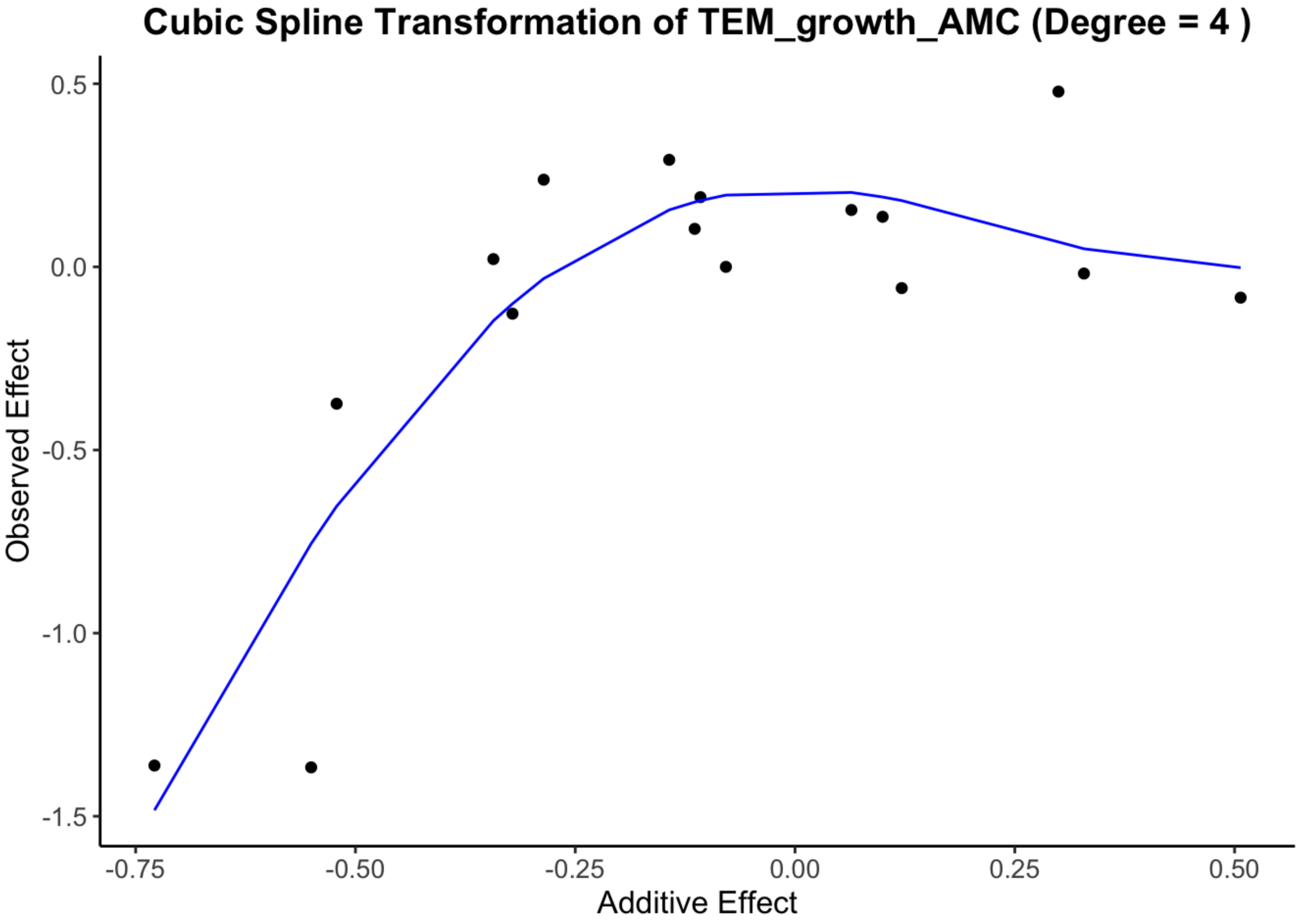
Cubic Spline Transformation of TEM_growth_AMC (Degree = 4)
Observed Effect
0.5
0.0
-0.5
-1.0
-1.5
-0.75
-0.50
-0.25
0.00
0.25
0.50
Additive Effect

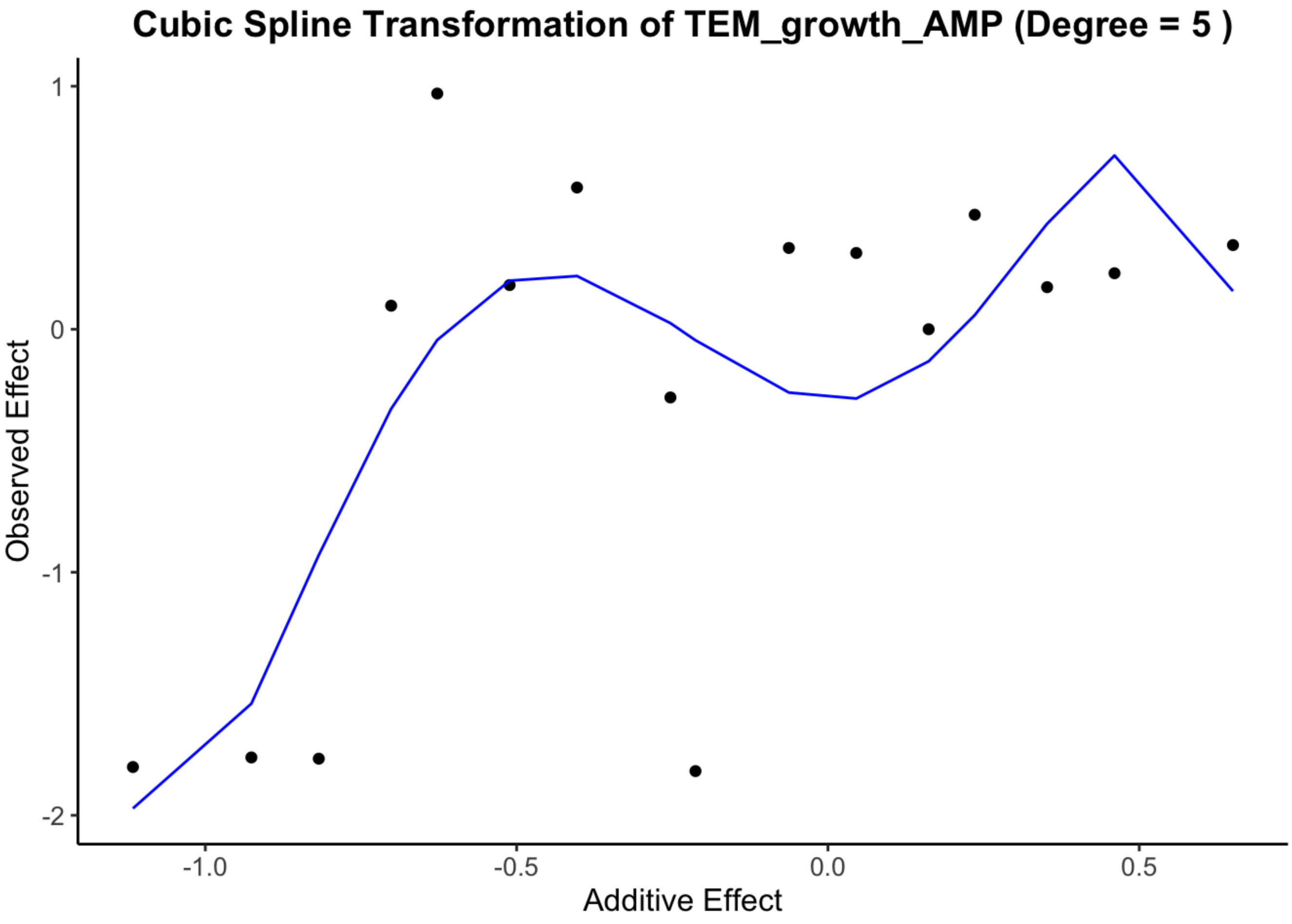

Cubic Spline Transformation of TEM_growth_AMP (Degree = 5)
Observed Effect
Additive Effect
1
0
-1
-2
-1.0
-0.5
0.0
0.5

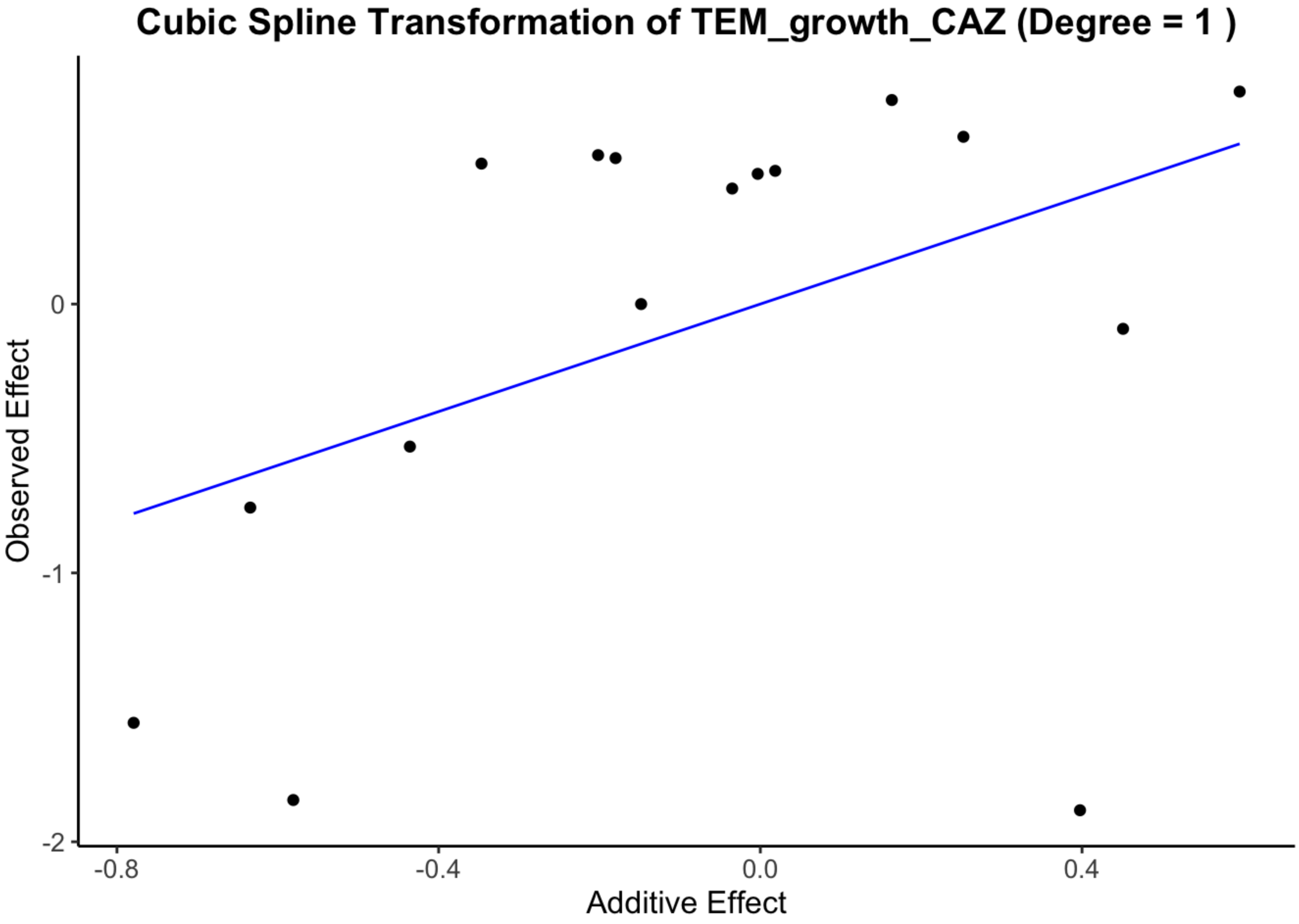

Cubic Spline Transformation of TEM_growth_CAZ (Degree = 1)
Observed Effect
Additive Effect
0
-1
-2
-0.8
-0.4
0.0
0.4

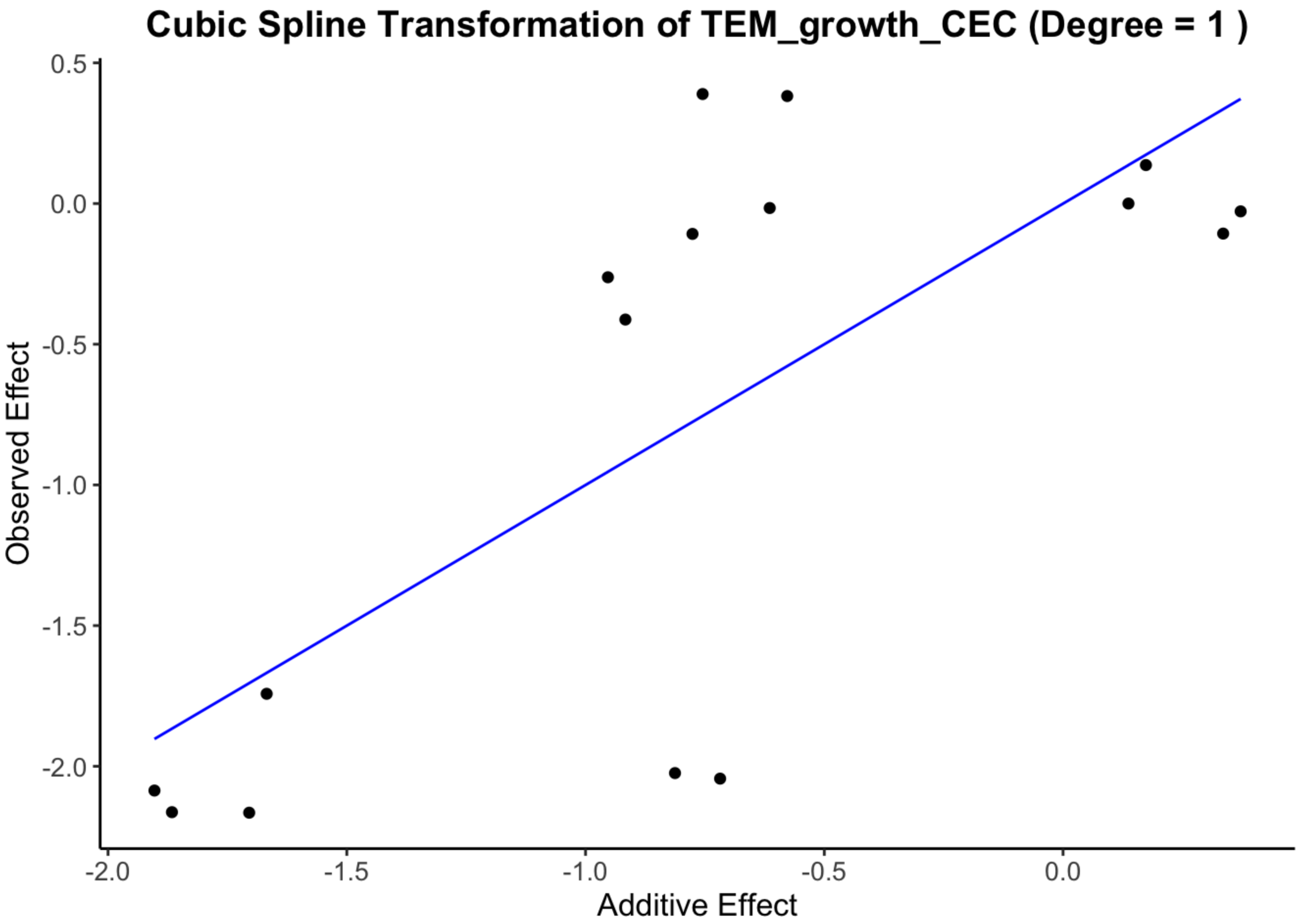
Cubic Spline Transformation of TEM_growth_CEC (Degree = 1)
Observed Effect
0.5
0.0
-0.5
-1.0
-1.5
-2.0
-2.0
-1.5
-1.0
-0.5
0.0
Additive Effect

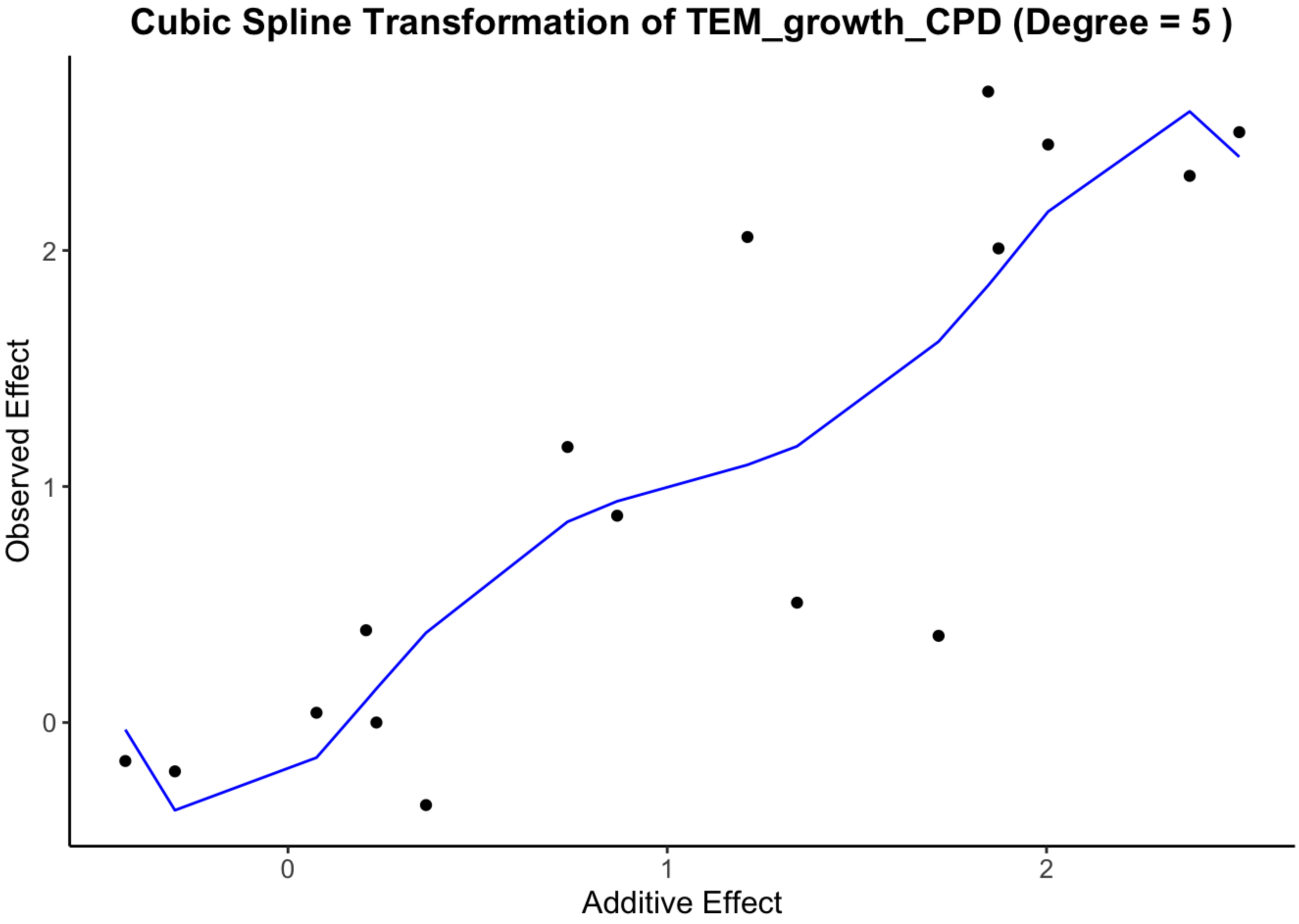
Cubic Spline Transformation of TEM_growth_CPD (Degree = 5)
Observed Effect
2
1
0
0
1
2
Additive Effect

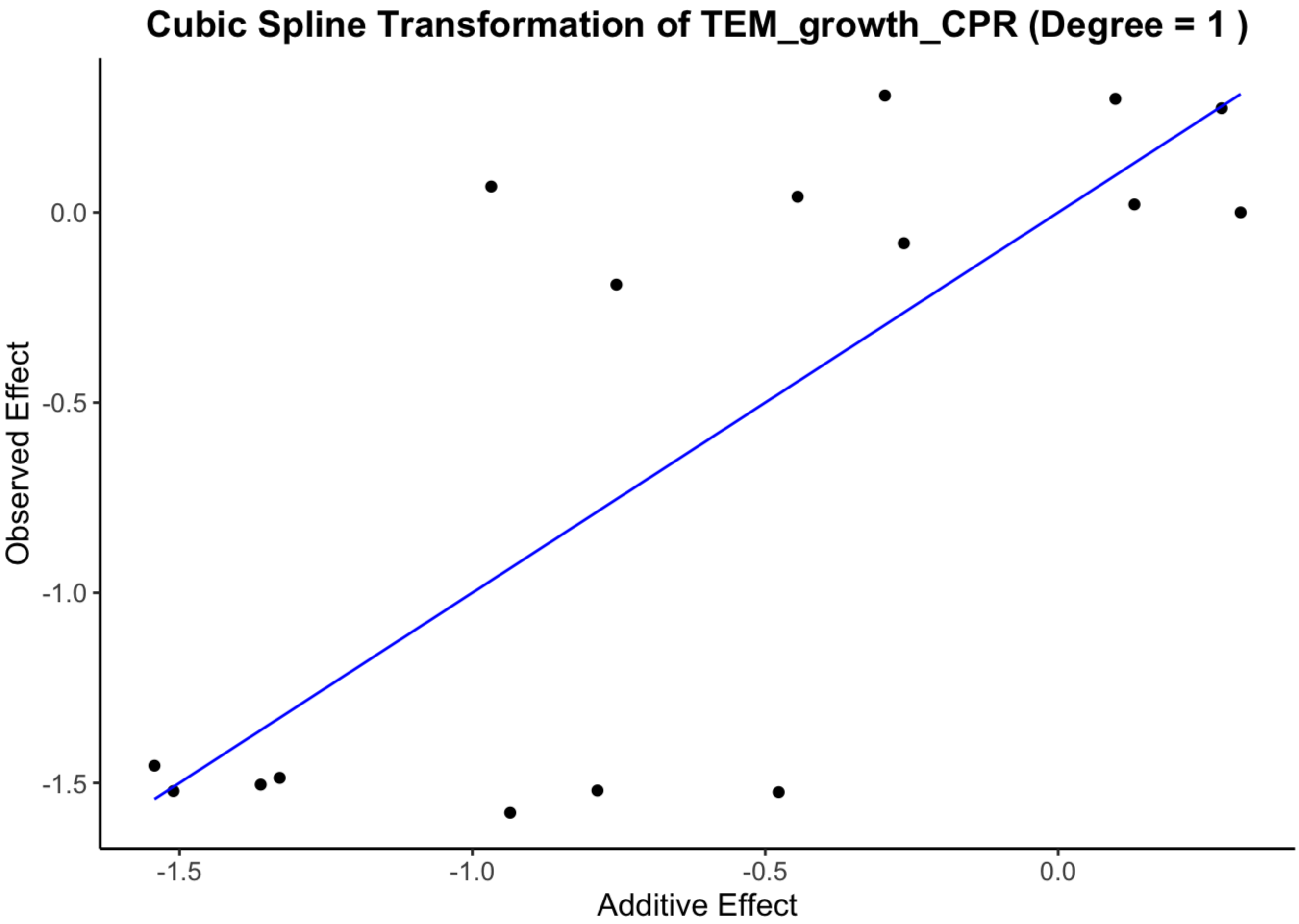

Cubic Spline Transformation of TEM_growth_CPR (Degree = 1)
Observed Effect
0.0
-0.5
-1.0
-1.5
-1.5
-1.0
-0.5
0.0
Additive Effect

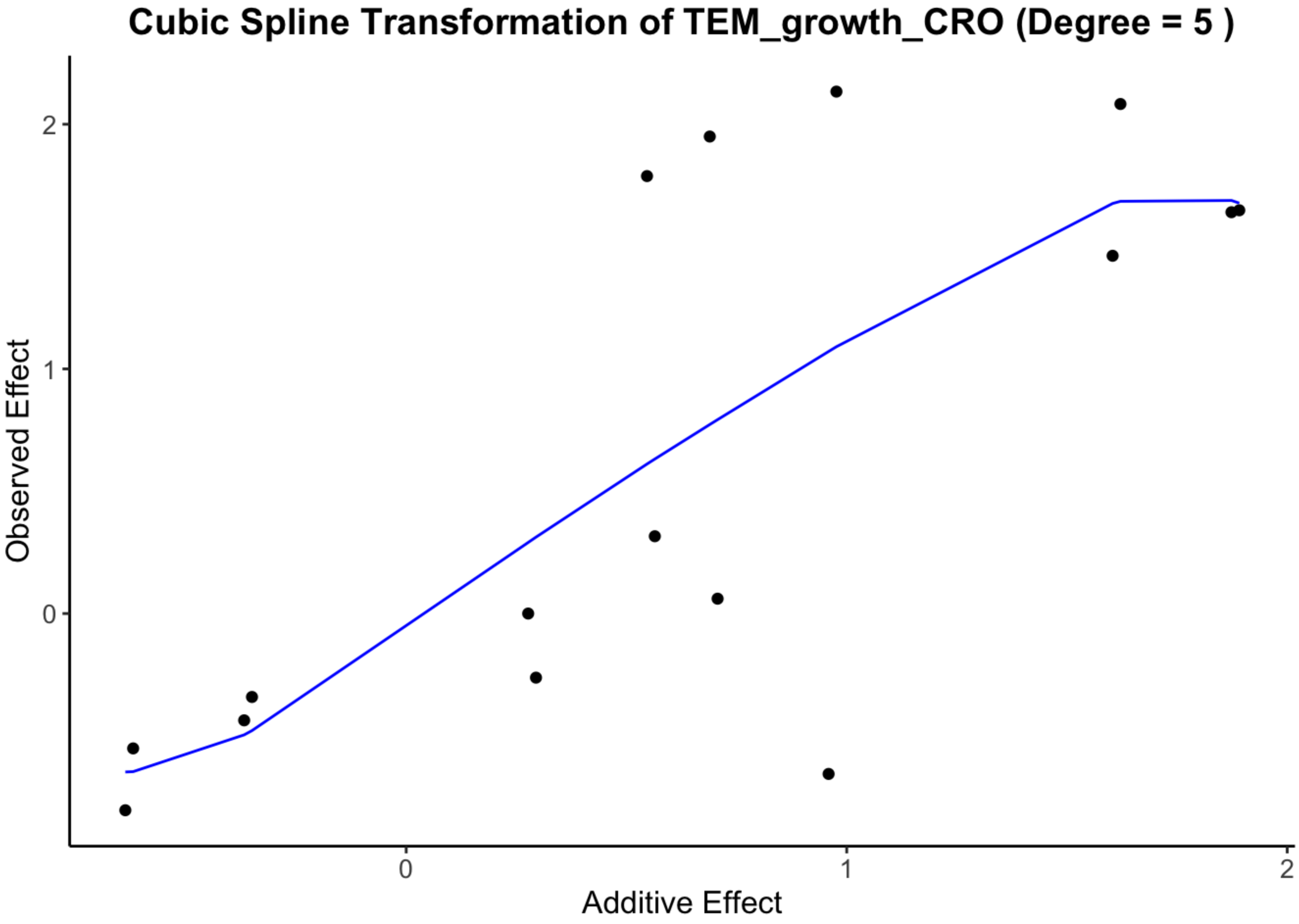

Cubic Spline Transformation of TEM_growth_CRO (Degree = 5)
Observed Effect
2
1
0
0
1
2
Additive Effect

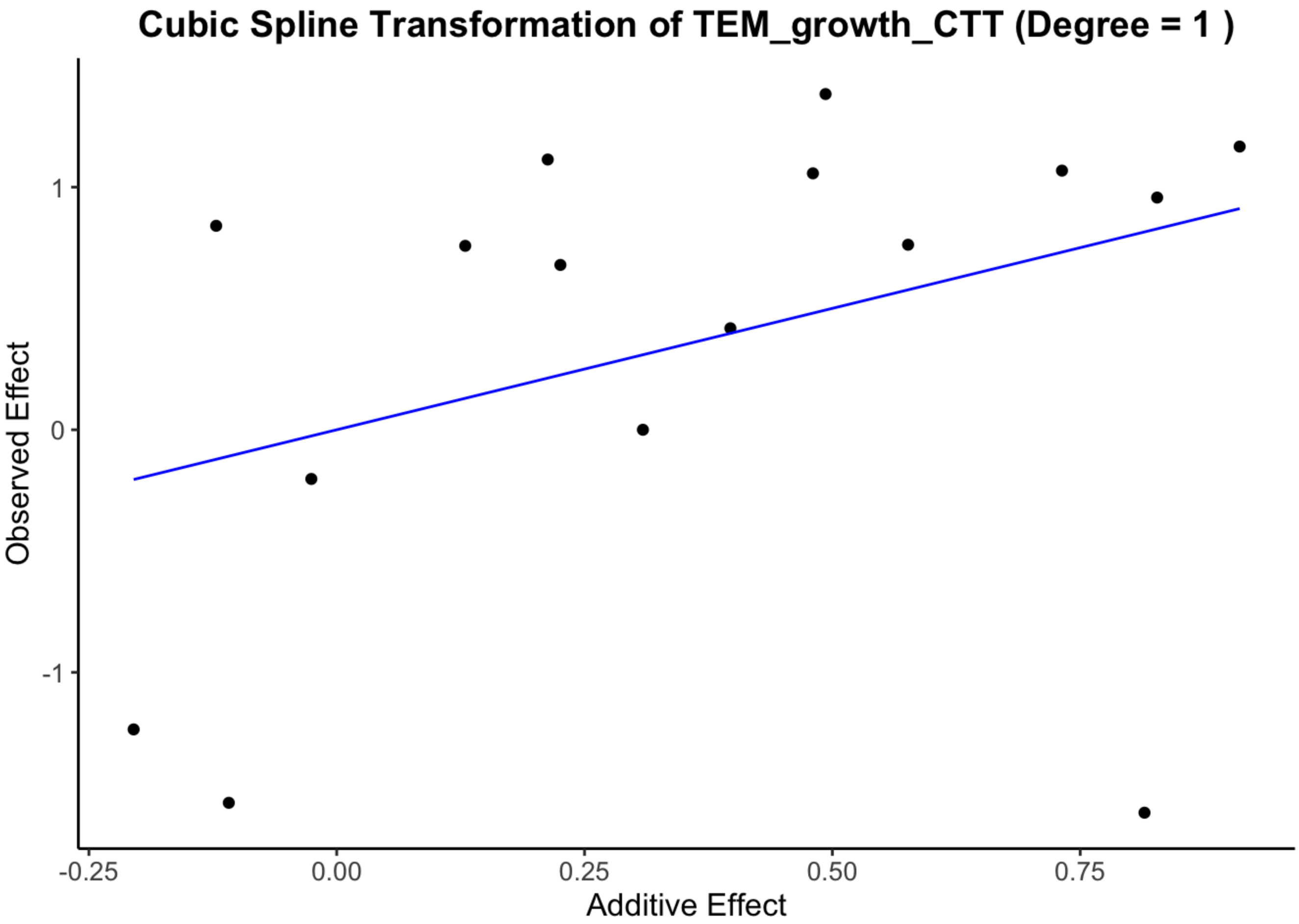
Cubic Spline Transformation of TEM_growth_CTT (Degree = 1)
Observed Effect
Additive Effect
1
0
-1
-0.25
0.00
0.25
0.50
0.75

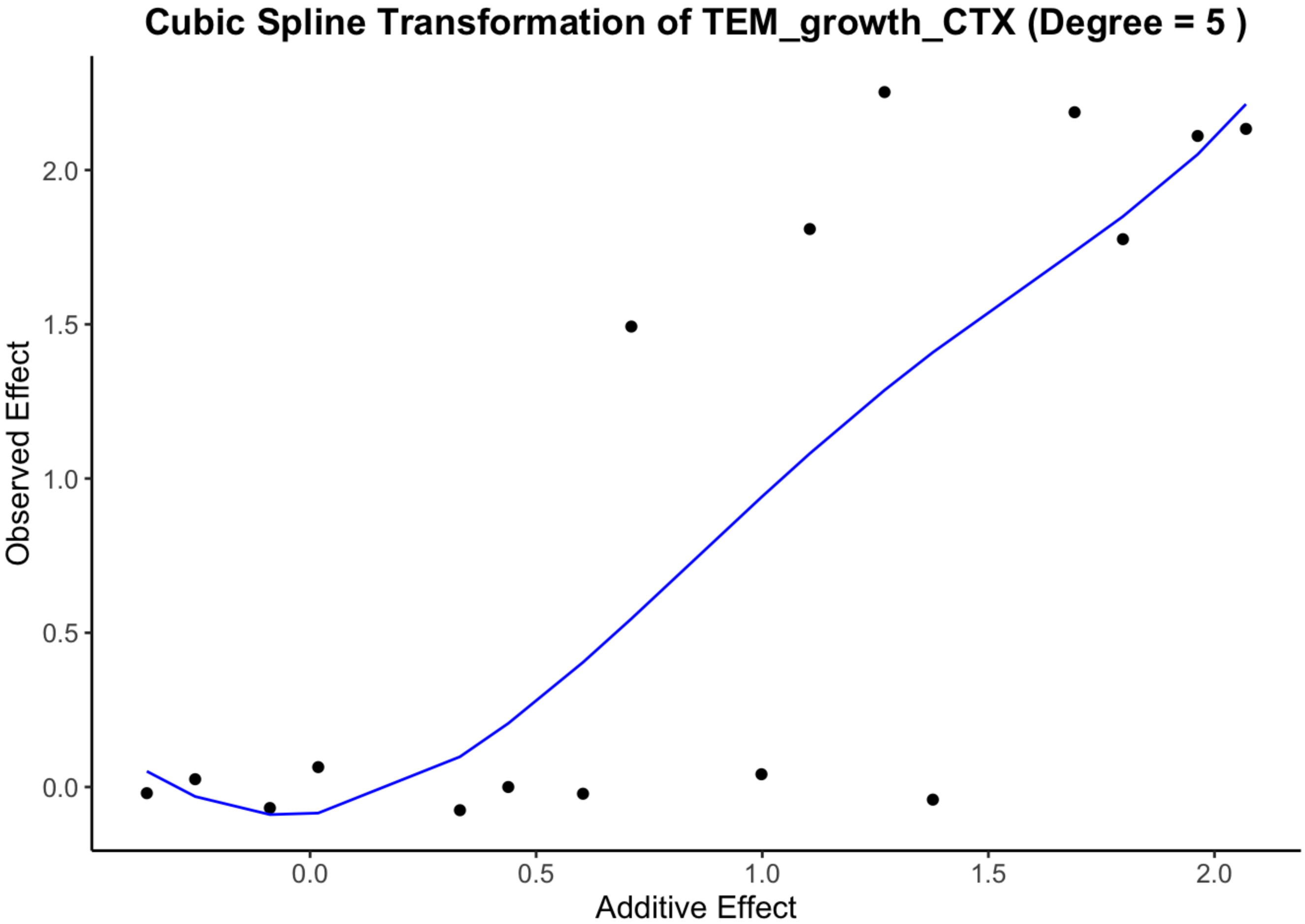
Cubic Spline Transformation of TEM_growth_CTX (Degree = 5)
Observed Effect
Additive Effect
2.0
1.5
1.0
0.5
0.0
0.0
0.5
1.0
1.5
2.0

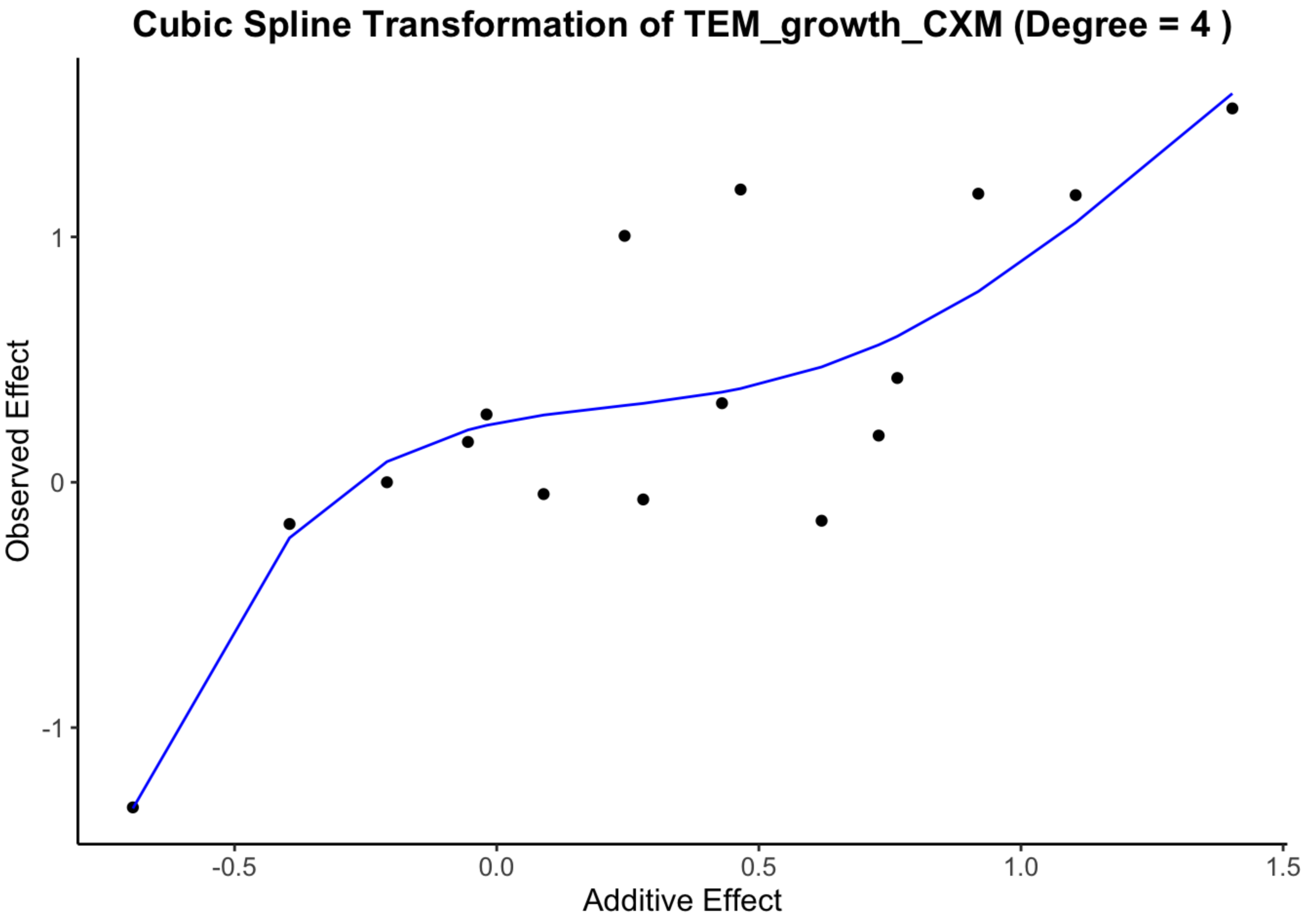
Cubic Spline Transformation of TEM_growth_CXM (Degree = 4)
Observed Effect
Additive Effect
1
0
-1
-0.5
0.0
0.5
1.0
1.5

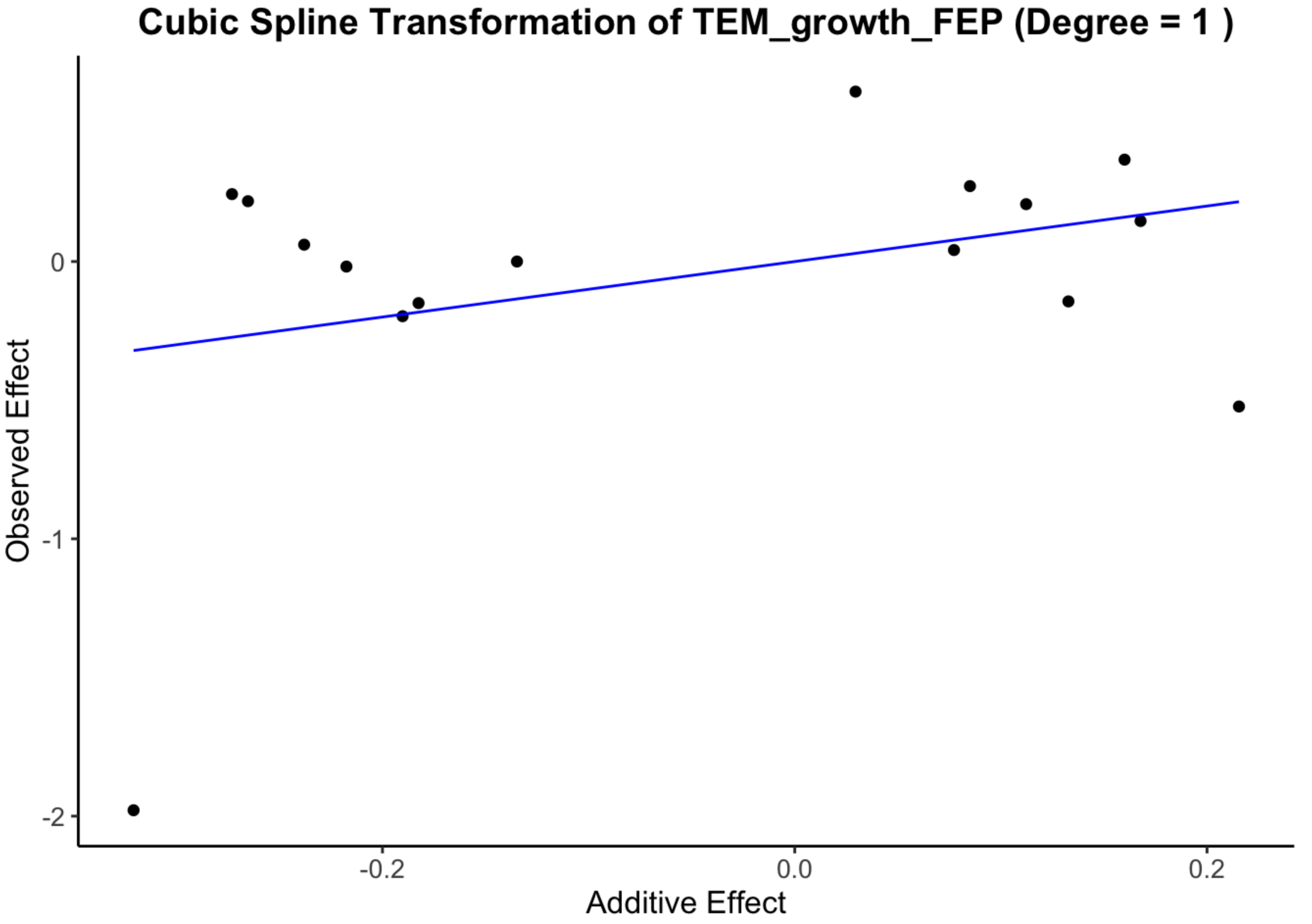
Cubic Spline Transformation of TEM_growth_FEP (Degree = 1)
Observed Effect
Additive Effect
0
-1
-2
-0.2
0.0
0.2

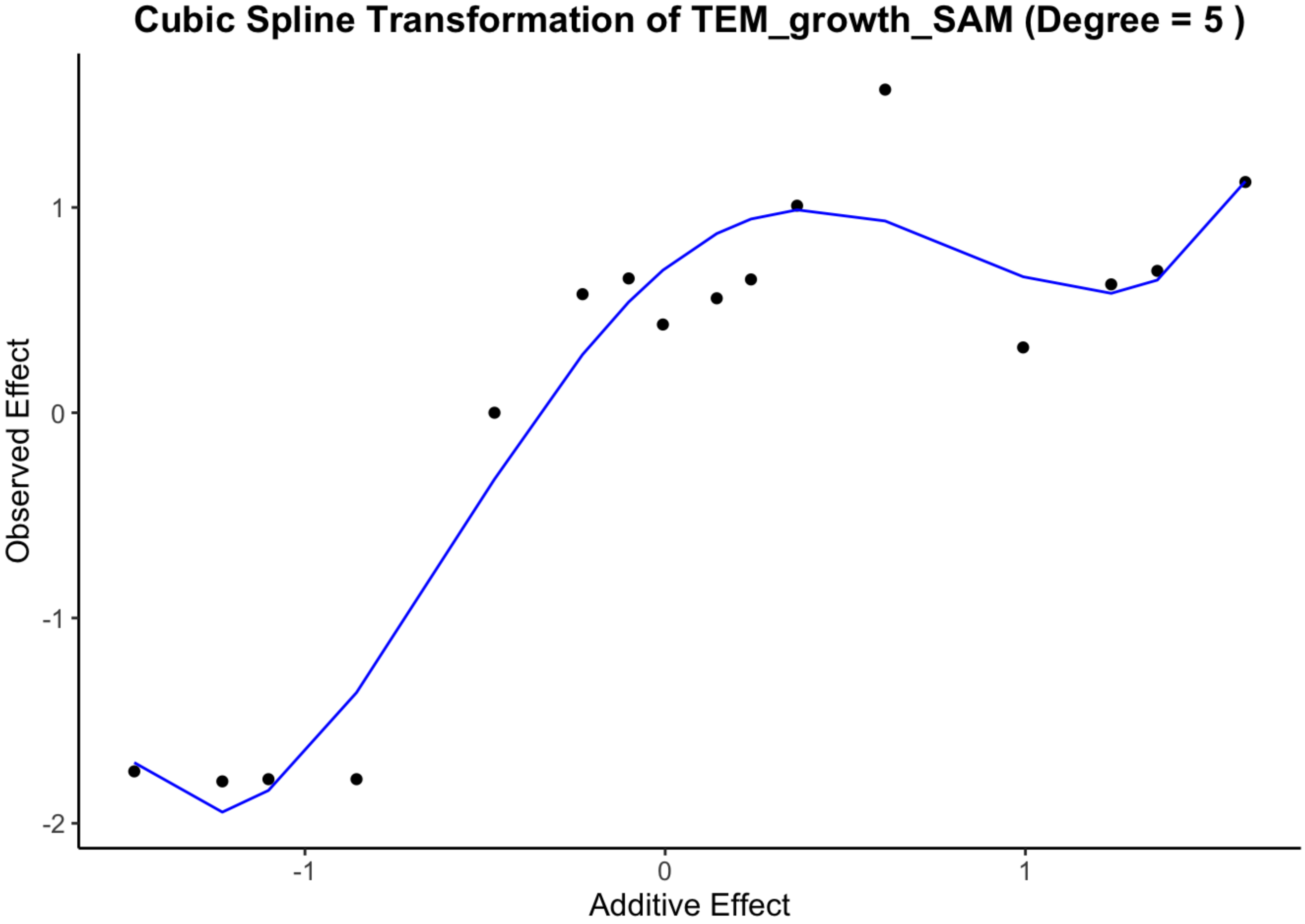

Cubic Spline Transformation of TEM_growth_SAM (Degree = 5)
Observed Effect
Additive Effect
1
0
-1
-2

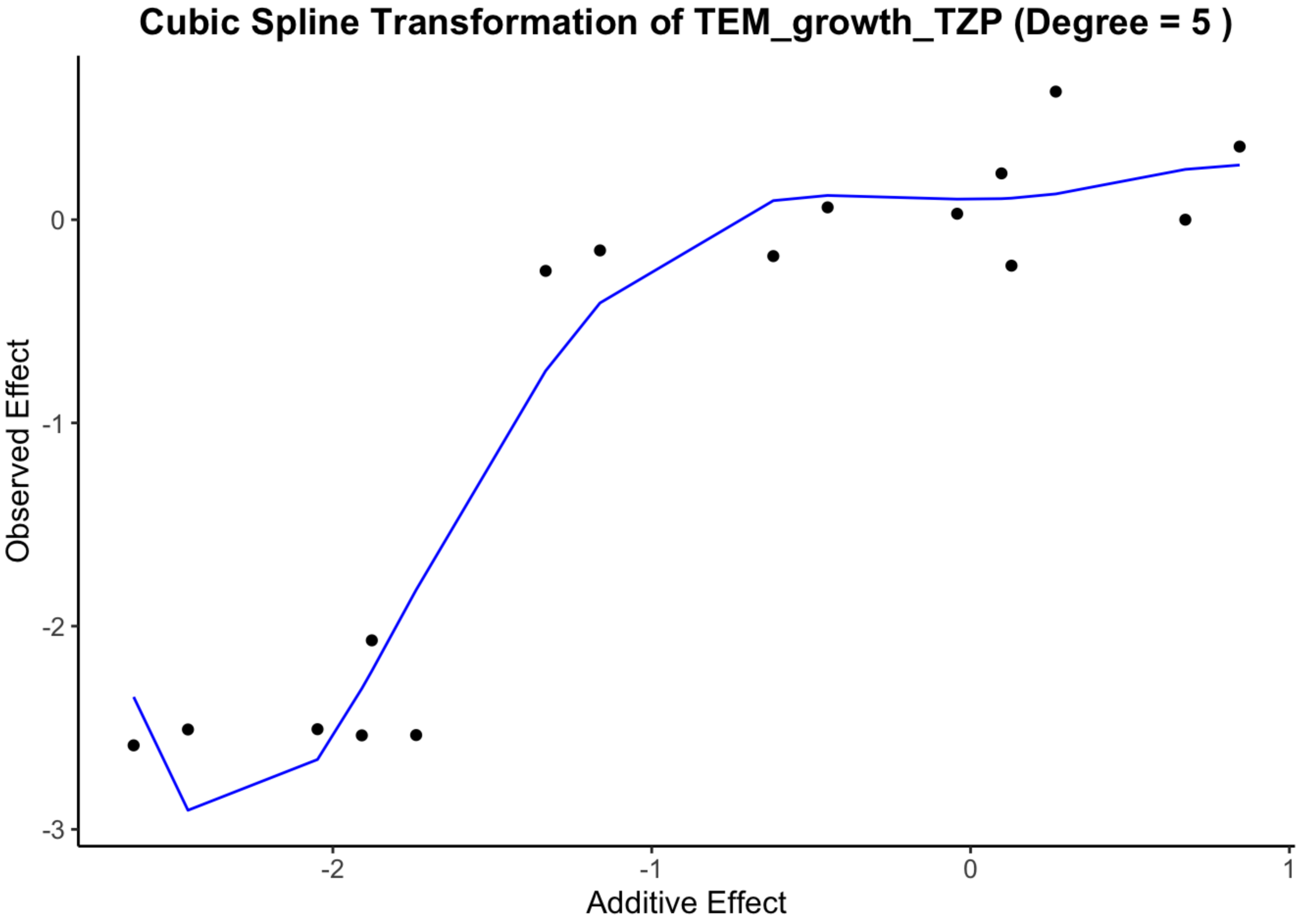

Cubic Spline Transformation of TEM_growth_TZP (Degree = 5)
Observed Effect
Additive Effect
0
-1
-2
-3
1

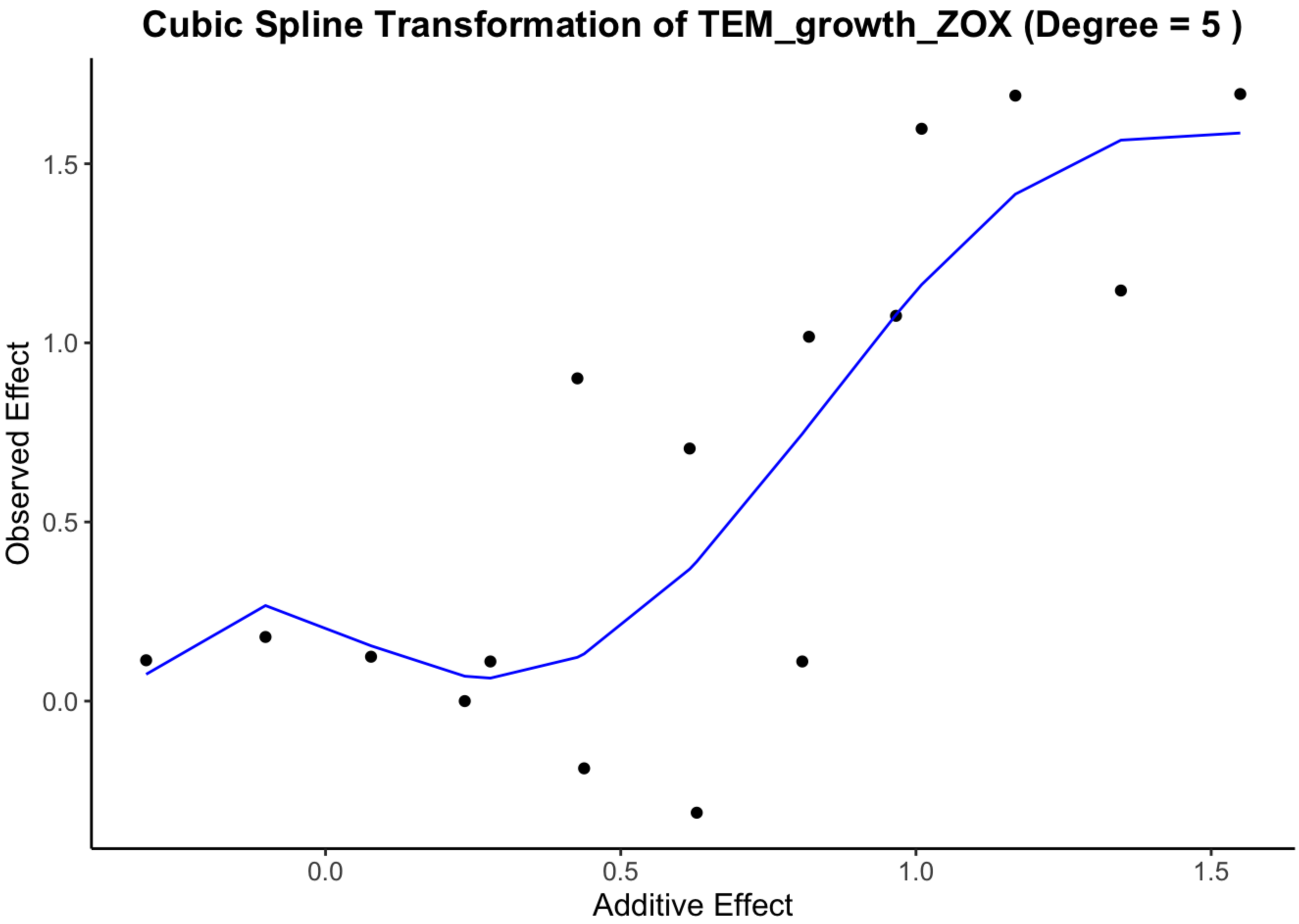

From the fits we notice a certain degree of over fitting. One approach to alleviate this would be to arbitrarily lower the number of degrees, however instead we opted to use a different transformation method which is less prone to over fitting.

Evaluation of four parameter transformations

The drawback of using monotonic splines (or even power transforms), as can be seen from the plots above, is the lack of bounding. Bounding is crucial to avoid greatly transforming phenotype values that lay outside of the bounds of the transform, which itself is constrained by the range of the predicted first-order effects.

In other words, phenotypes with magnitudes that lay outside of the range of predicted values by the first-order model are at risk of being incorrectly transformed. Though this is somewhat true for a four-parameter model, the bounded upper- and lower-thresholds ensure that phenotypes that lay outside of the predicted range are likely restricted to the upper and lower bounds.

Four parameter function transform

We attempted to transform all datasets using the four-parameter function, with fitting performed by non-linear least squares regression using `nlsLM`. The fits are compared to a simple linear model fit using an AIC to determine whether the additional information stemming from the four-parameter transform is parsimonious, otherwise, no transform is applied.

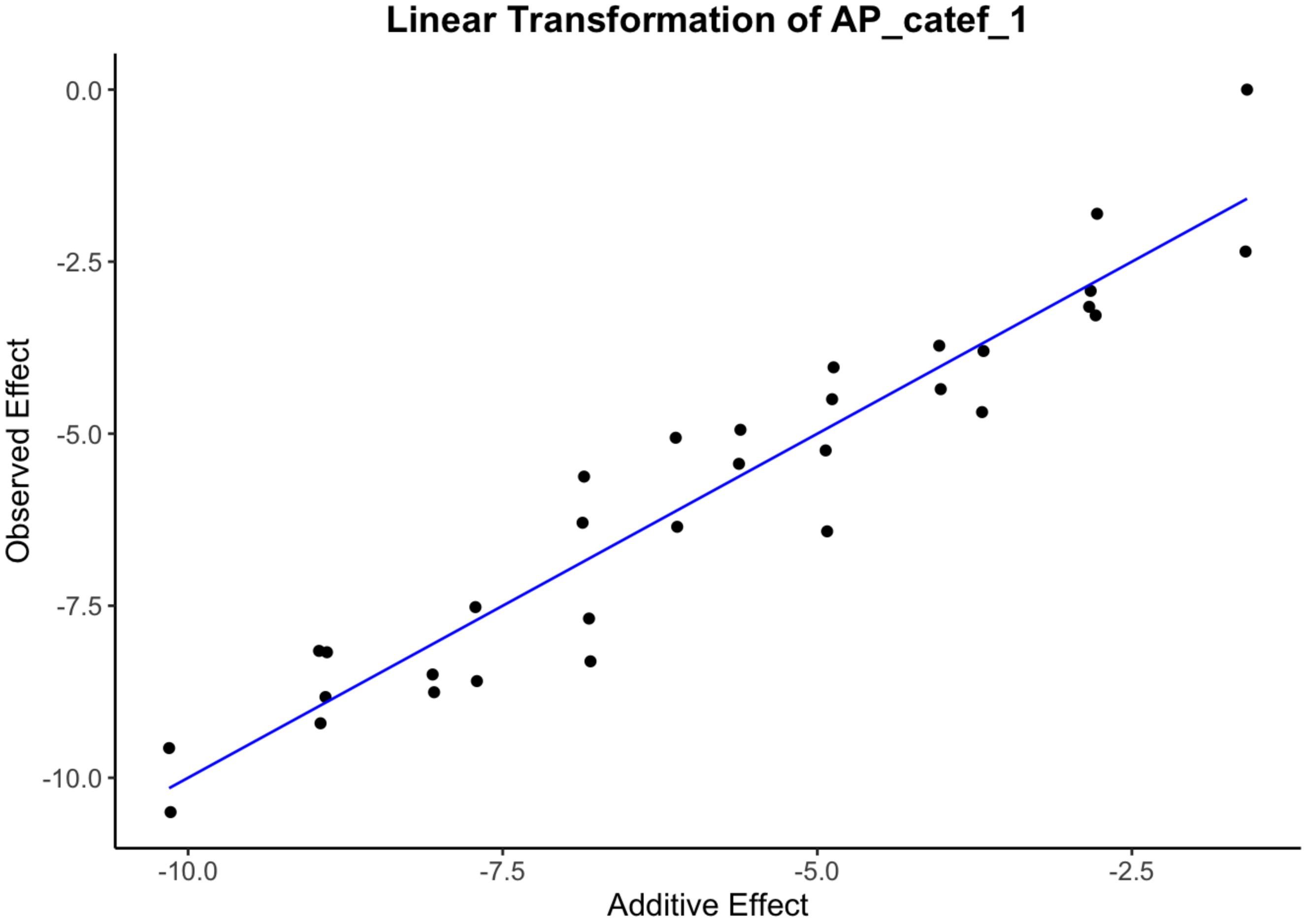

Linear Transformation of AP_catef_1
0.0
-2.5
-5.0
-7.5
-10.0
Observed Effect
-10.0
-7.5
-5.0
-2.5
Additive Effect

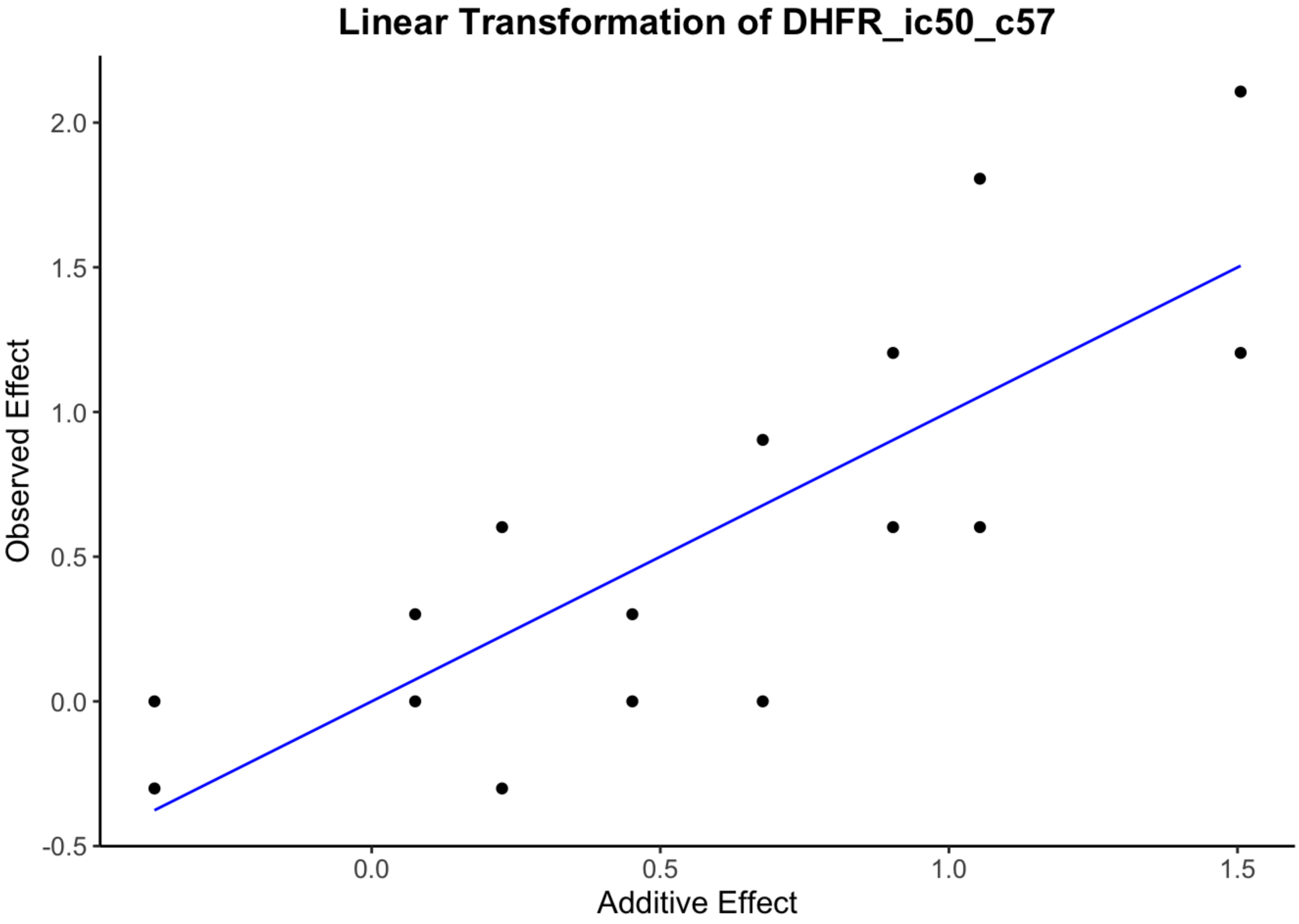

Linear Transformation of DHFR_ic50_c57
2.0
1.5
1.0
0.5
0.0
-0.5
Observed Effect
0.0
0.5
1.0
1.5
Additive Effect

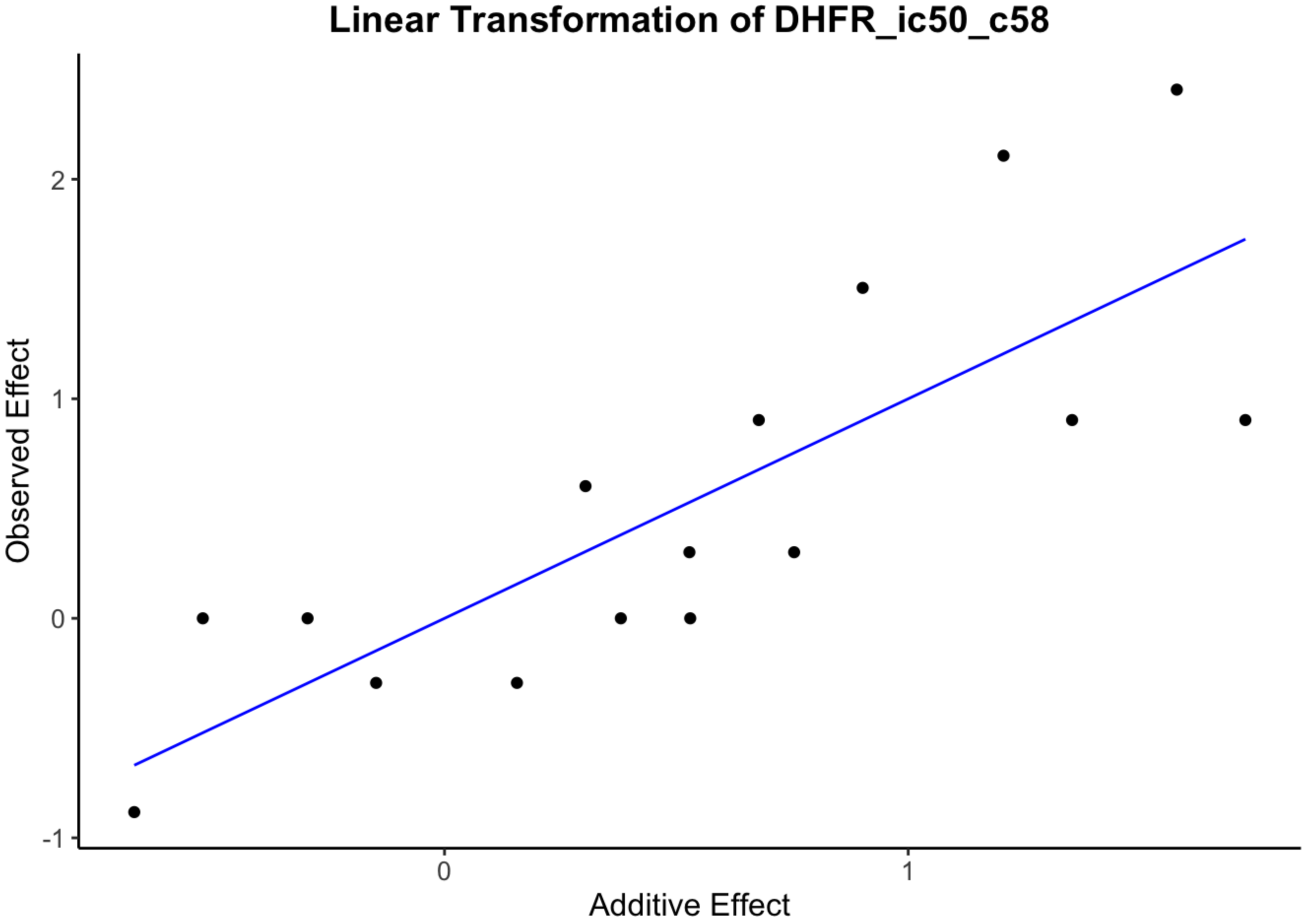
Linear Transformation of DHFR_ic50_c58
Observed Effect
Additive Effect
2
1
0
-1
0
1

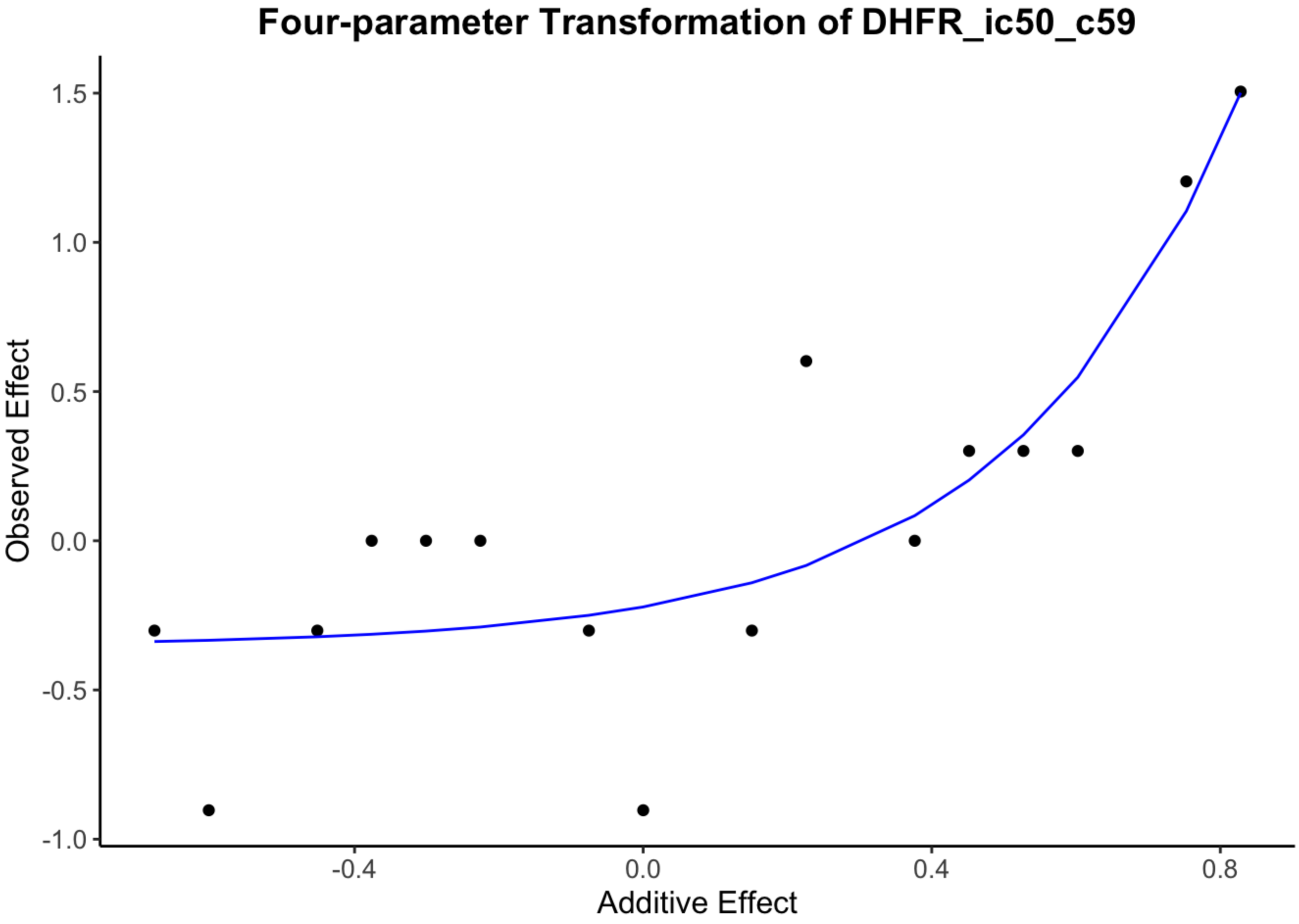
Four-parameter Transformation of DHFR_ic50_c59
Observed Effect
Additive Effect
1.5
1.0
0.5
0.0
-0.5
-1.0
-0.4
0.0
0.4
0.8

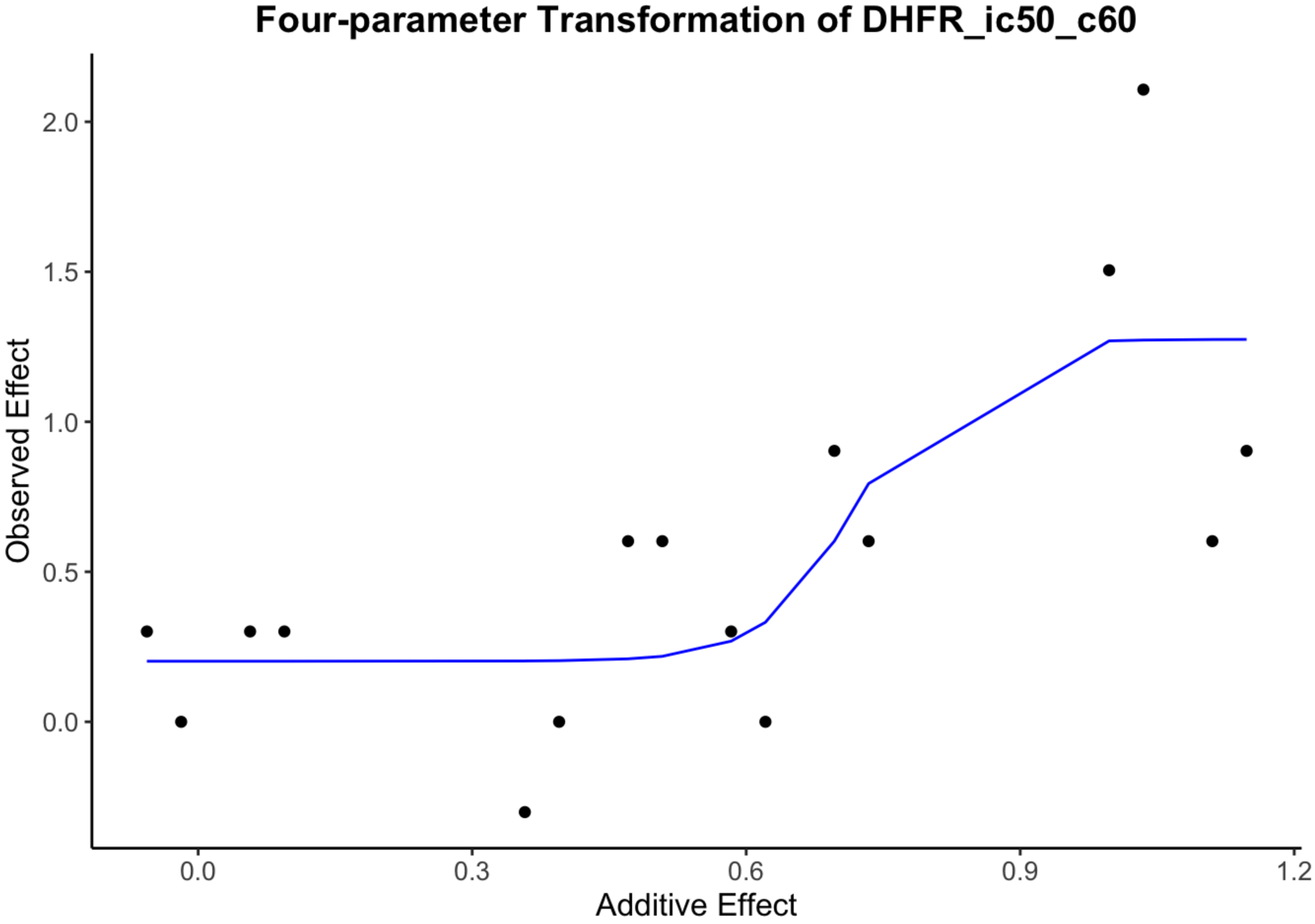


```
## Error in nlsModel(formula, mf, start, wts) :
##   singular gradient matrix at initial parameter estimates
```

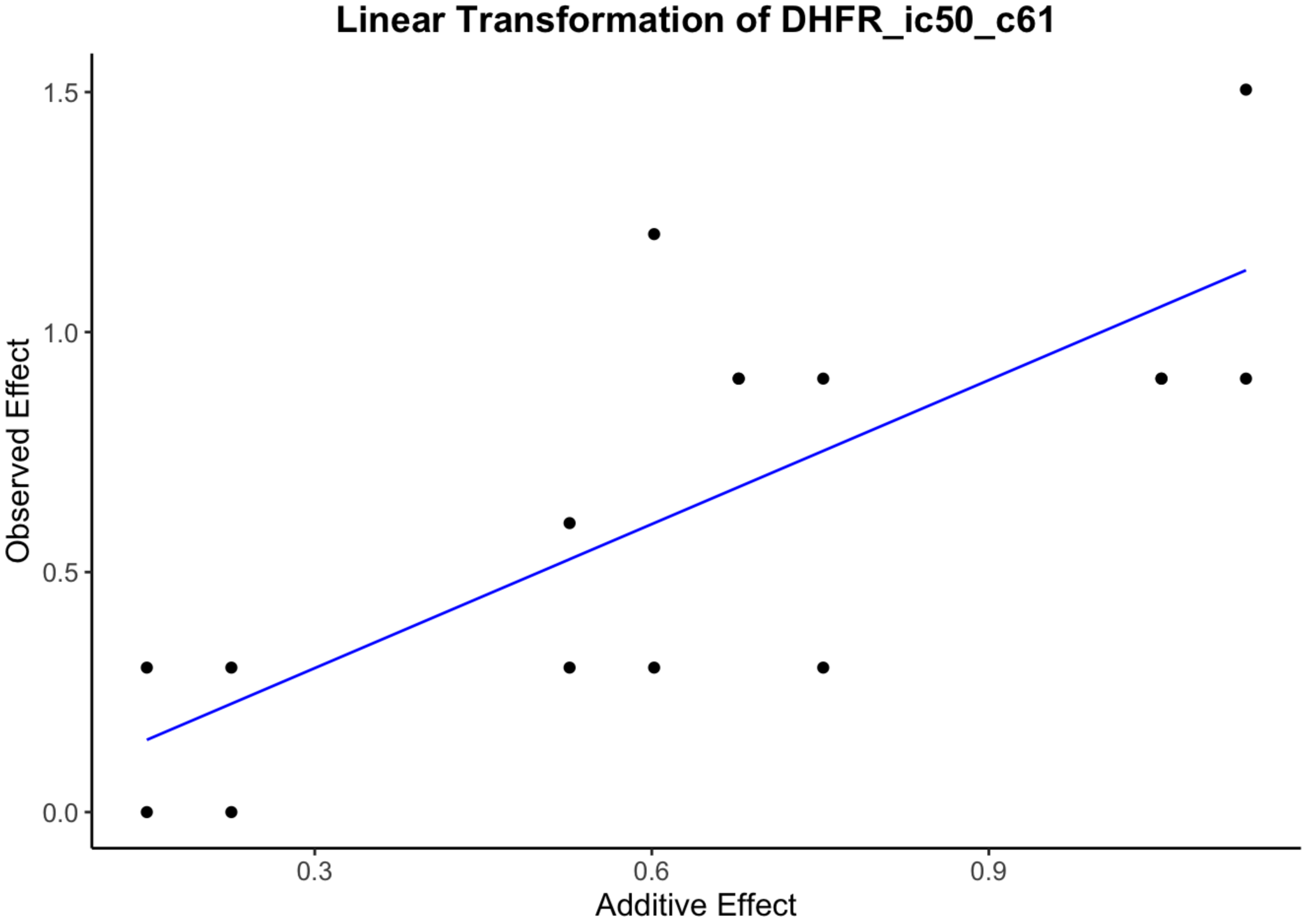
Linear Transformation of DHFR_ic50_c61
Observed Effect
Additive Effect
0.0
0.5
1.0
1.5
0.3
0.6
0.9

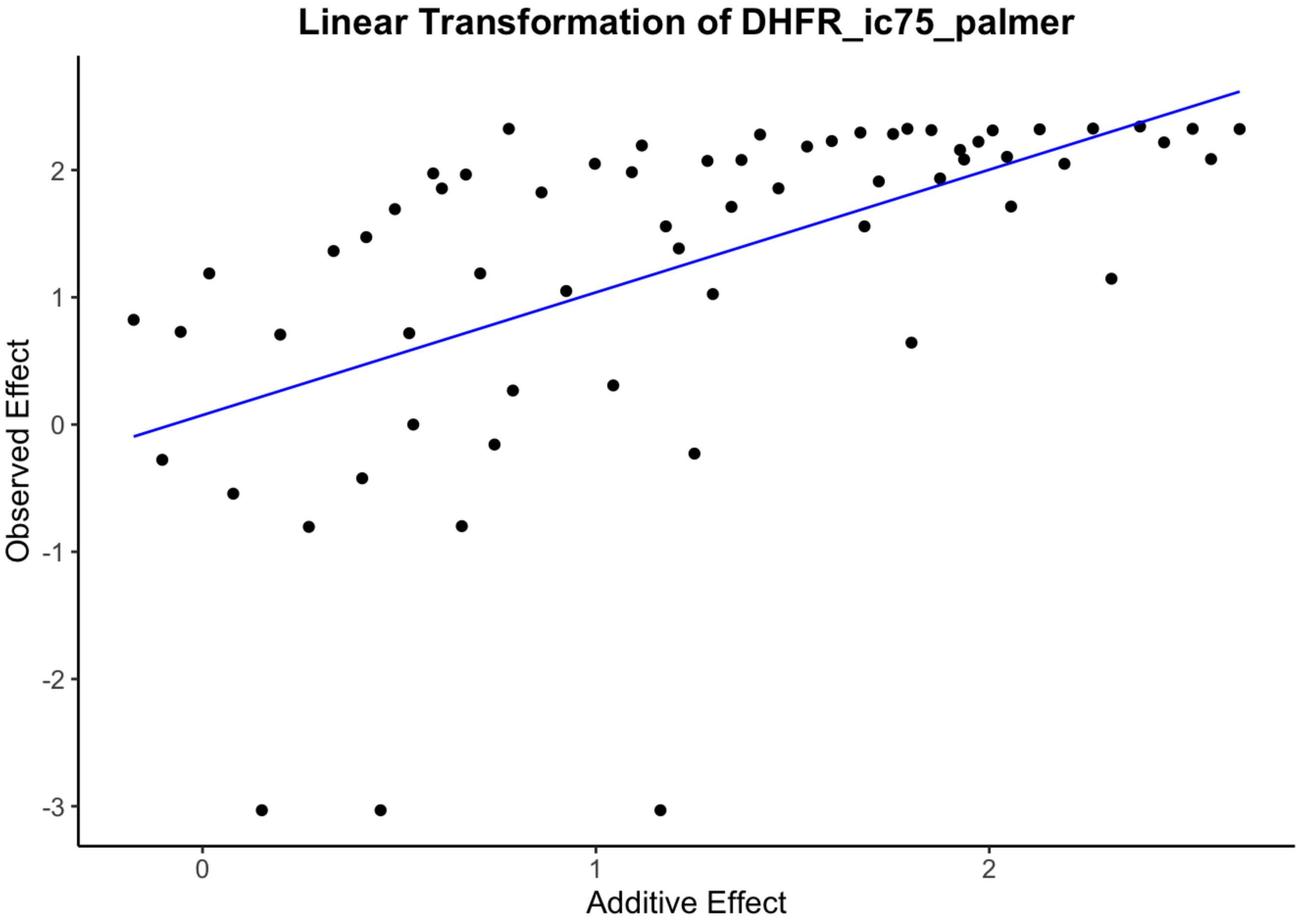
Linear Transformation of DHFR_ic75_palmer
Observed Effect
Additive Effect
-3
-2
-1
0
1
2
0
1
2

Four-parameter Transformation of DHFR_kcat_trajg

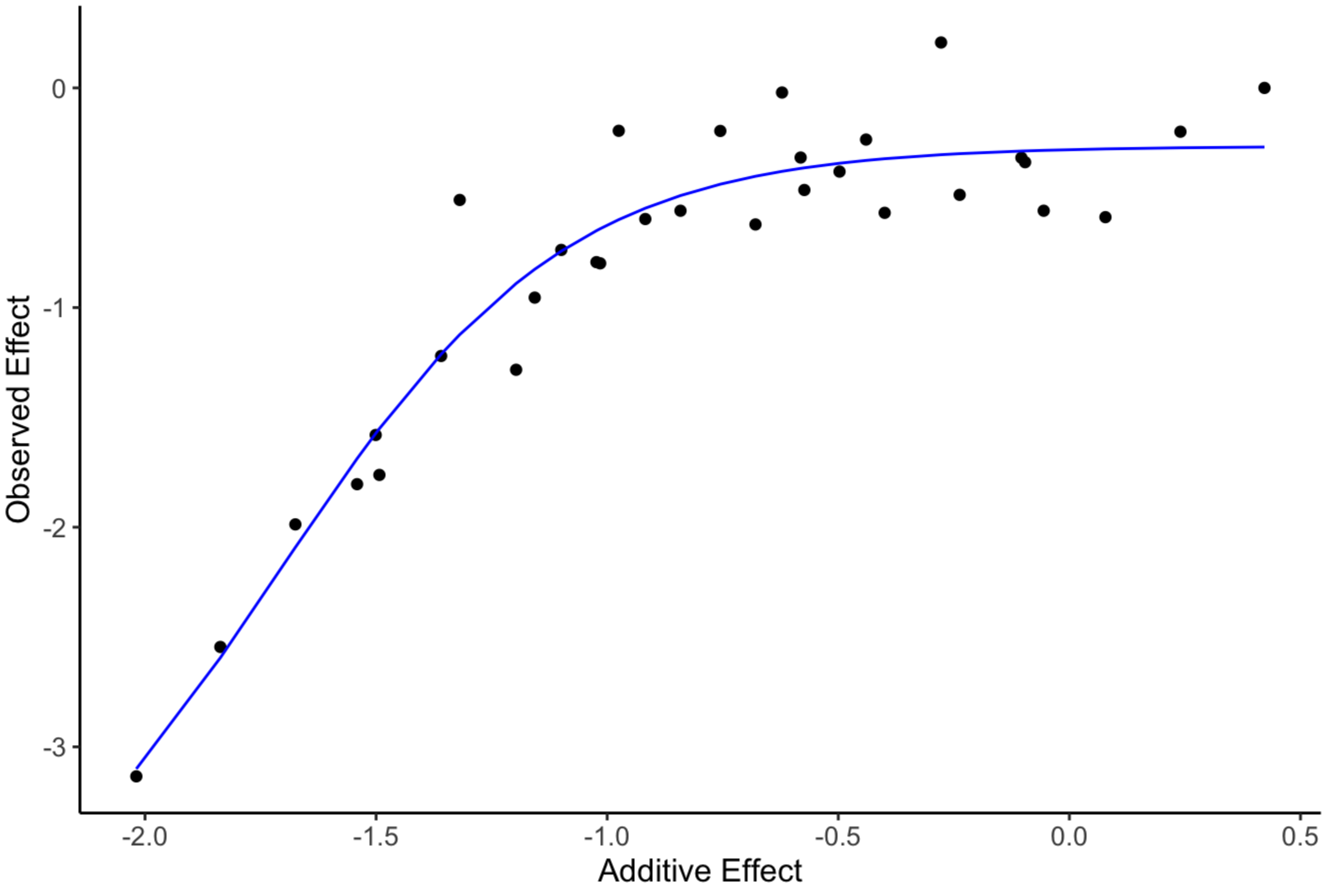

Four-parameter Transformation of DHFR_kcat_trajr

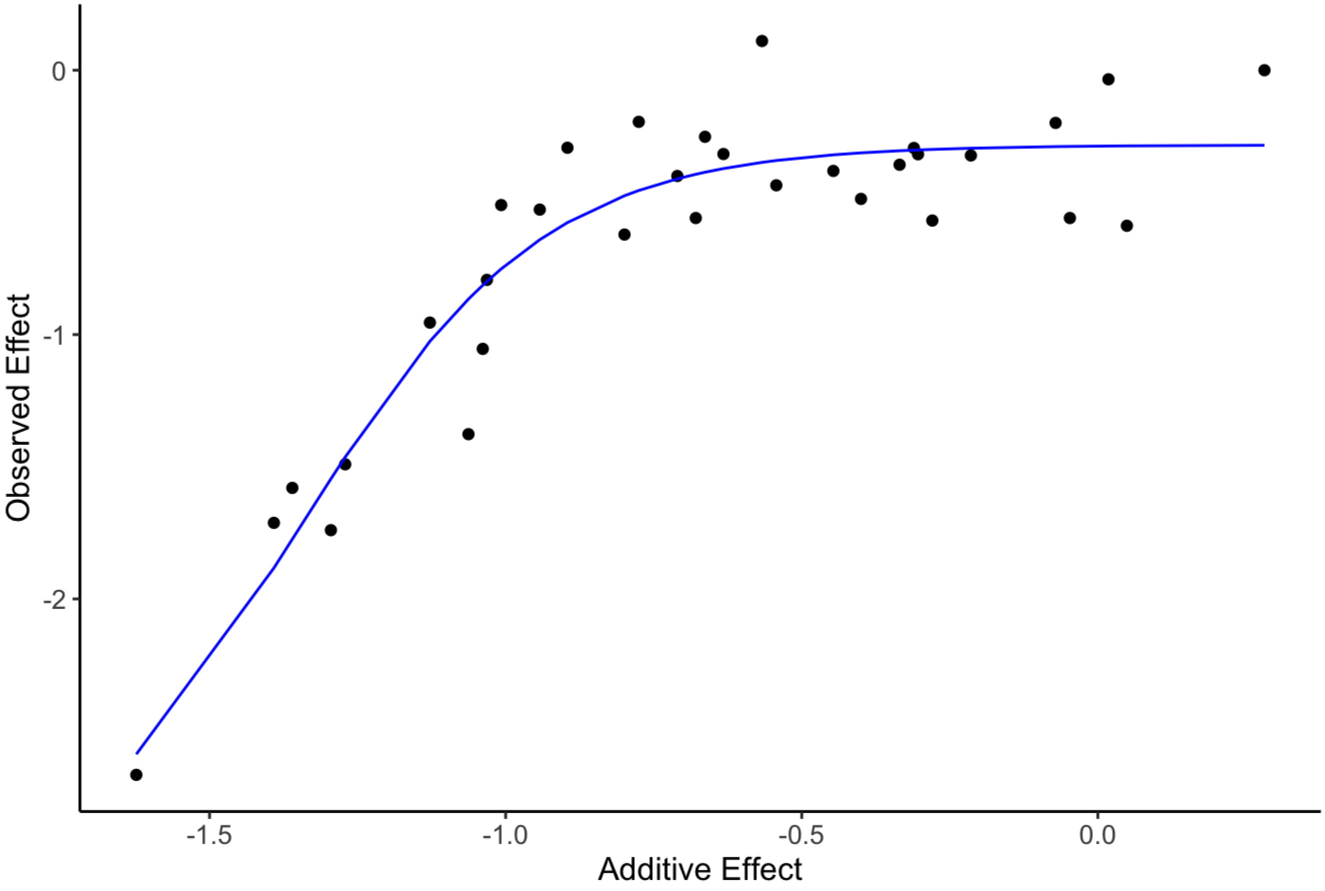

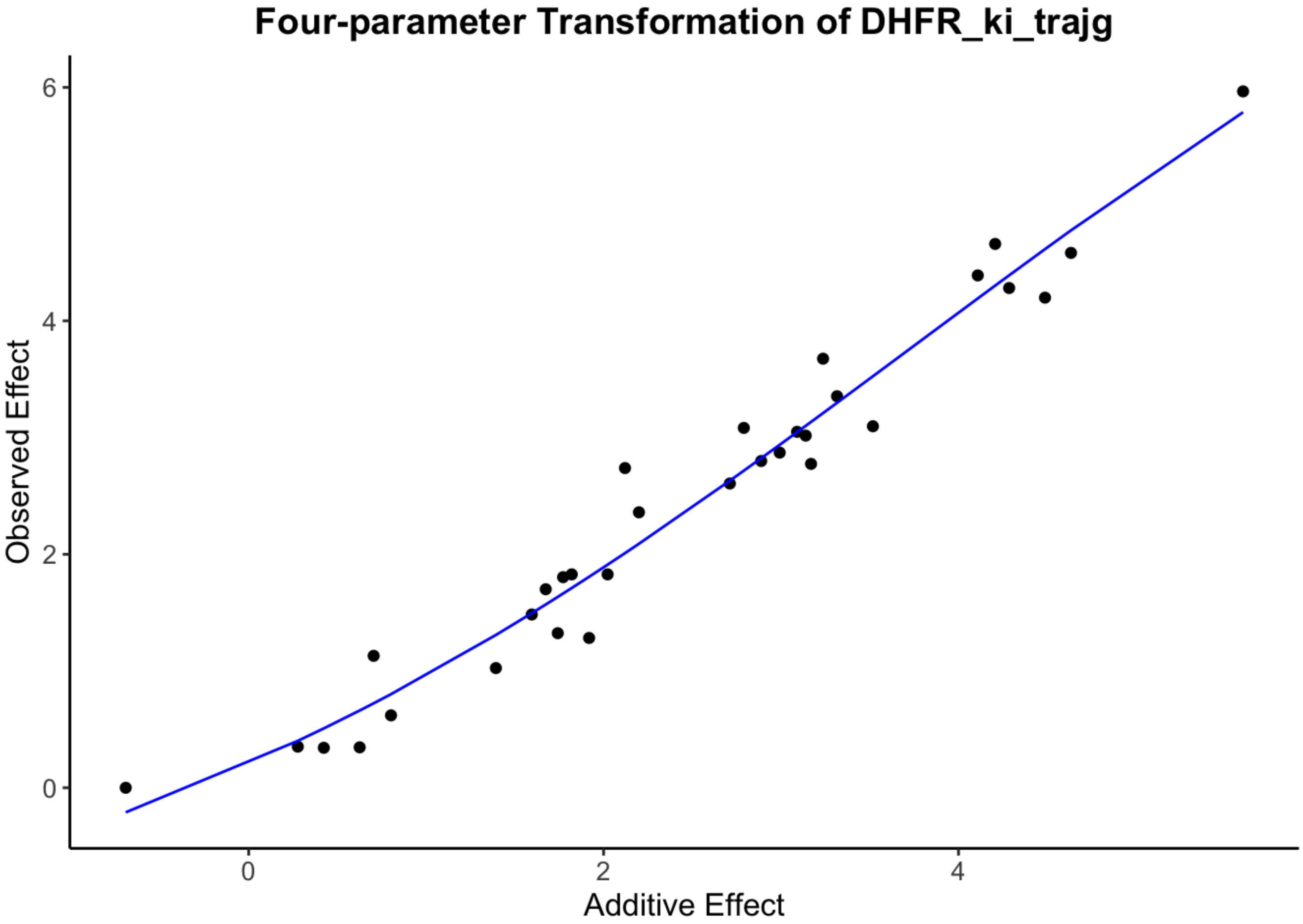
Four-parameter Transformation of DHFR_ki_trajg
Observed Effect
Additive Effect
0
2
4
6
0
2
4

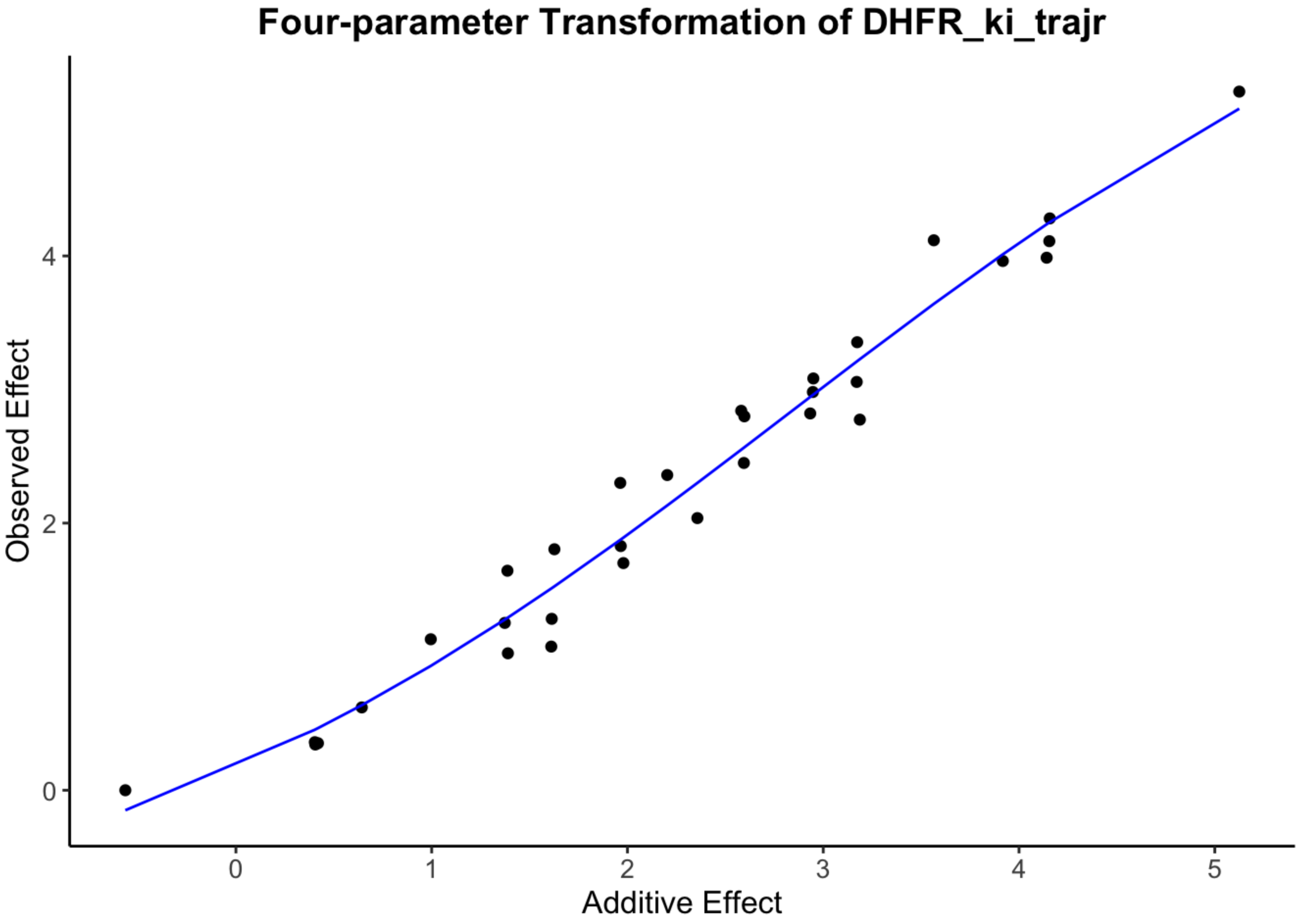
Four-parameter Transformation of DHFR_ki_trajr
Observed Effect
Additive Effect
0
2
4
0
1
2
3
4
5

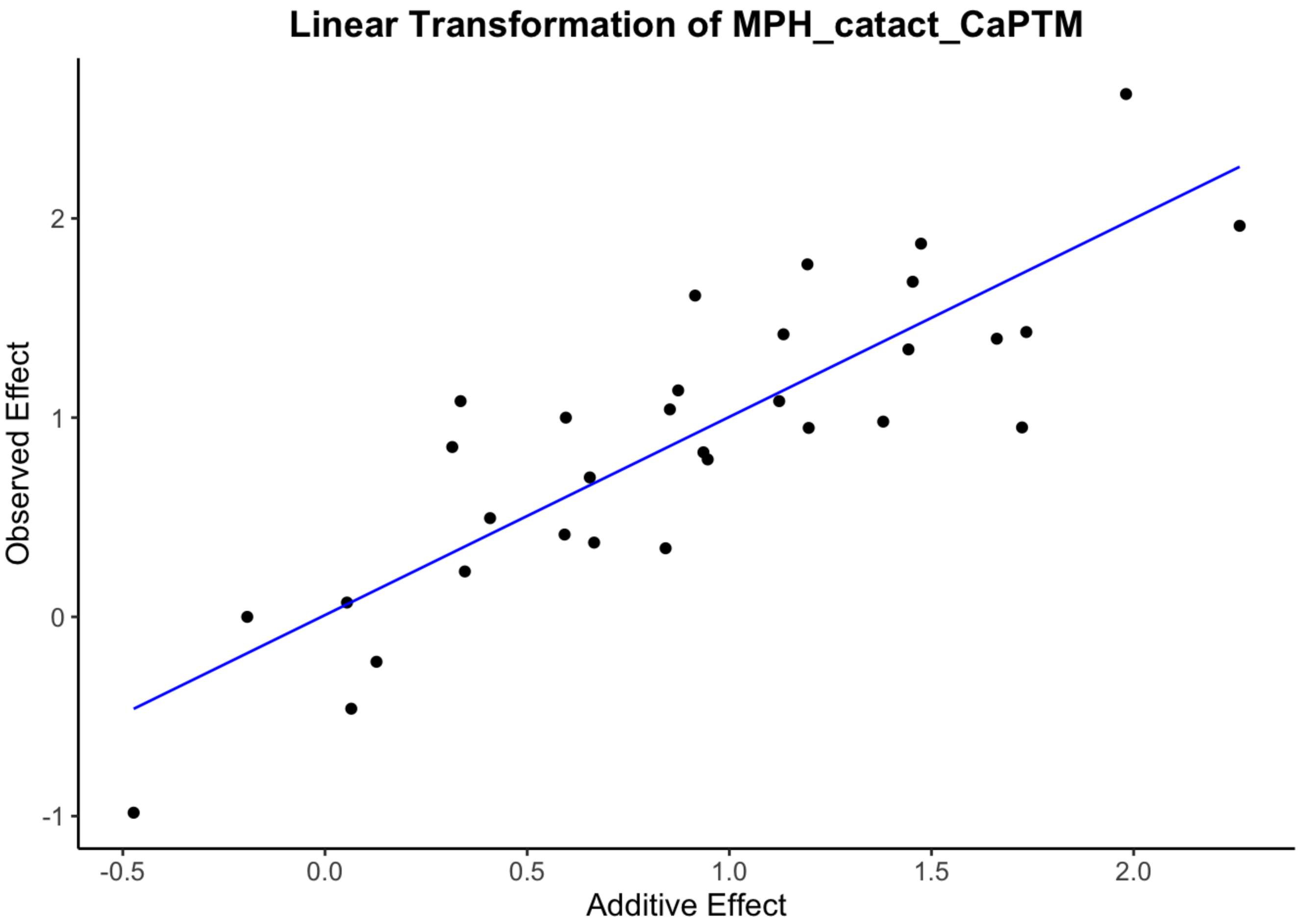
Linear Transformation of MPH_catact_CaPTM
Observed Effect
Additive Effect
-1
0
1
2
-0.5
0.0
0.5
1.0
1.5
2.0

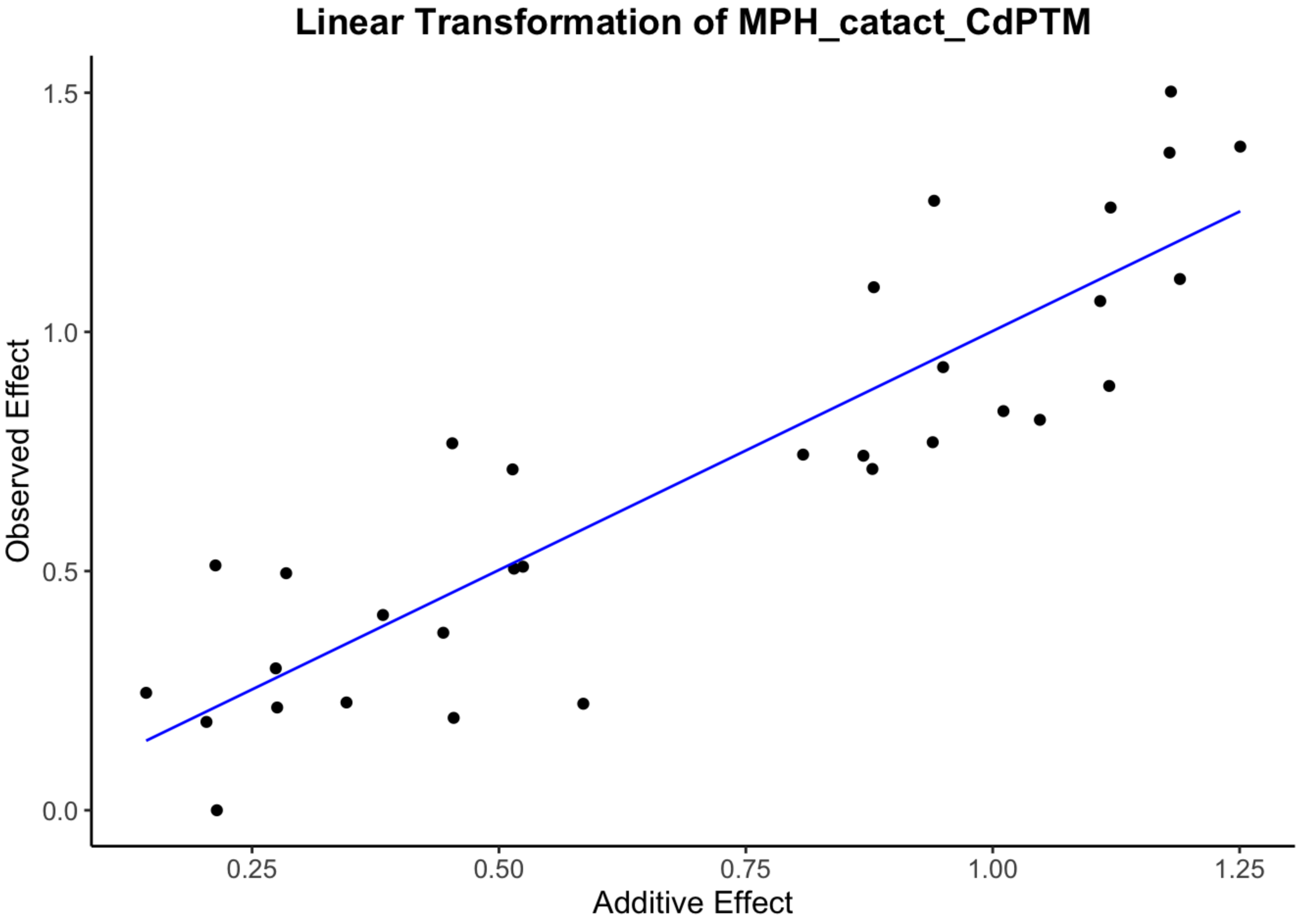
Linear Transformation of MPH_catact_CdPTM
Observed Effect
Additive Effect
0.0
0.5
1.0
1.5
0.25
0.50
0.75
1.00
1.25

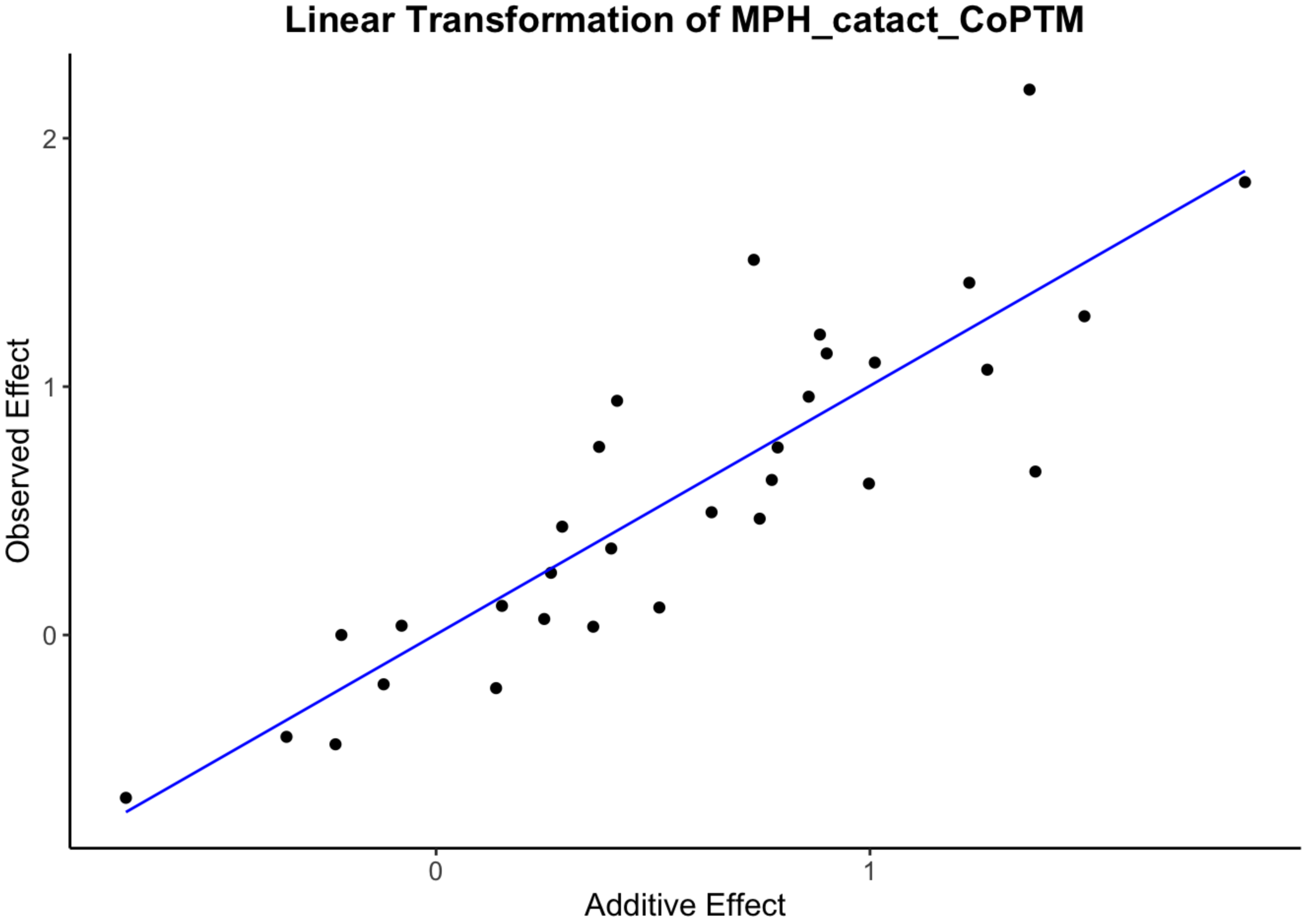
Linear Transformation of MPH_catact_CoPTM
Observed Effect
Additive Effect
2
1
0
0
1

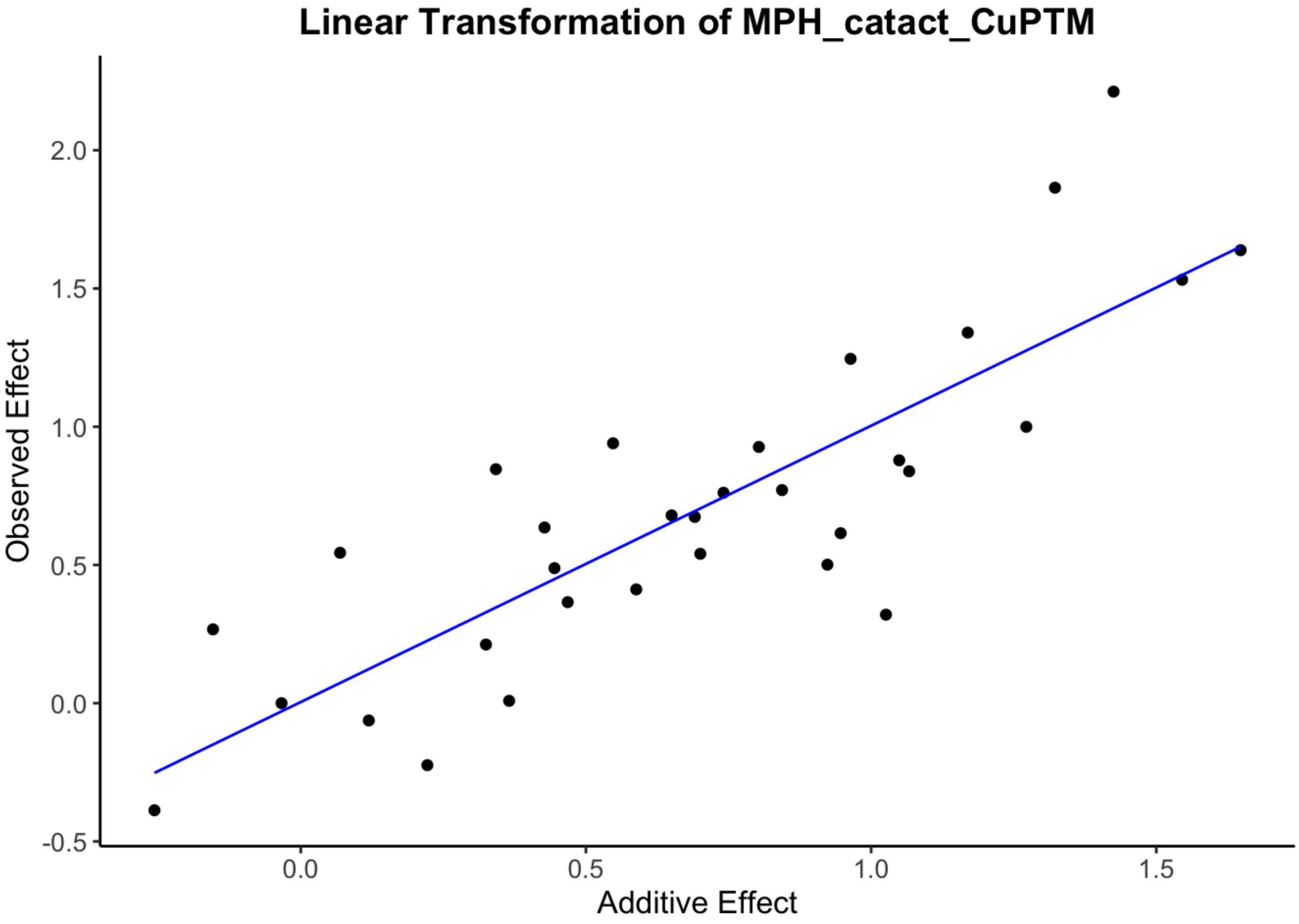
Linear Transformation of MPH_catact_CuPTM
Observed Effect
Additive Effect
2.0
1.5
1.0
0.5
0.0
-0.5
0.0
0.5
1.0
1.5

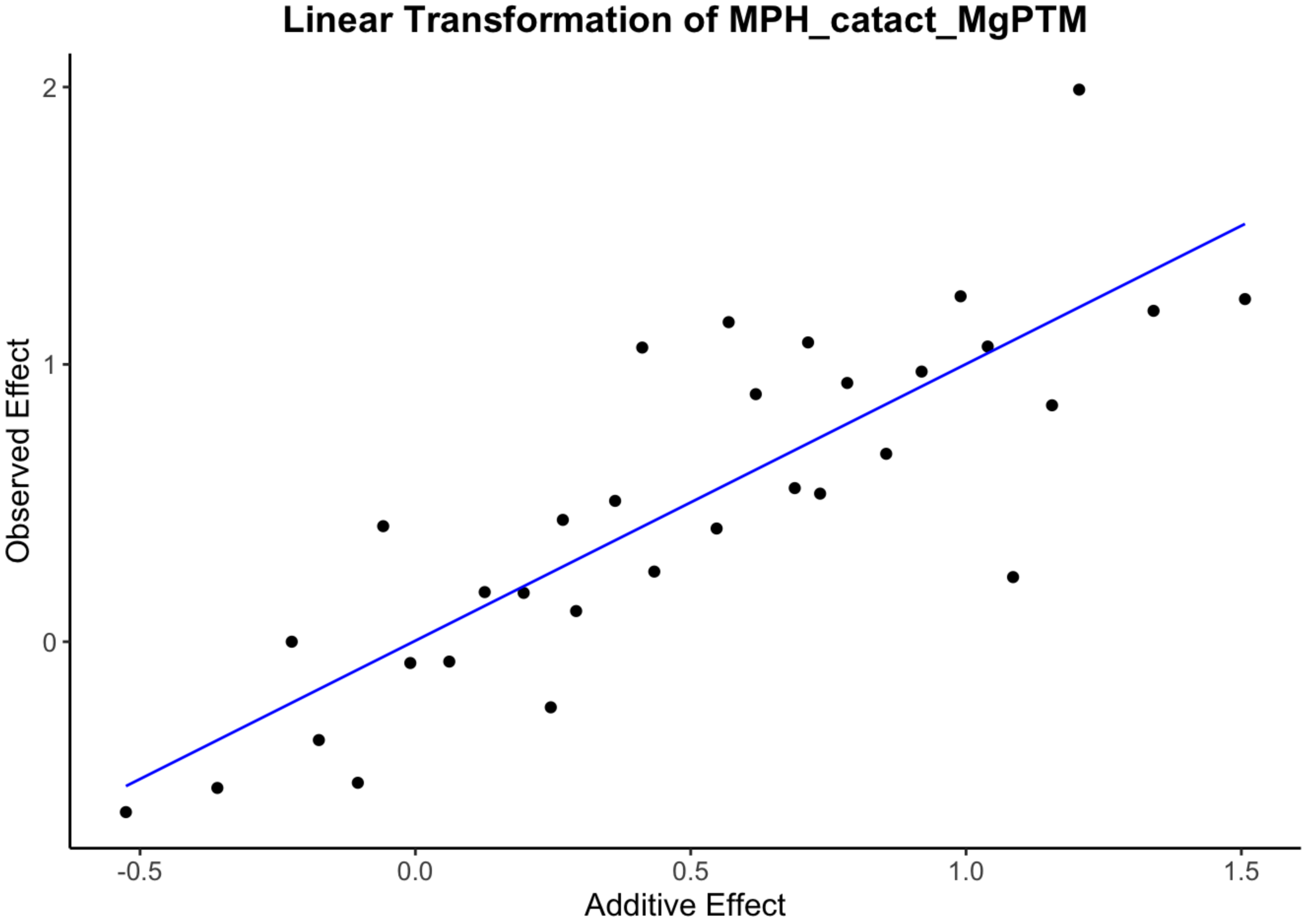
Linear Transformation of MPH_catact_MgPTM
Observed Effect
Additive Effect
2
1
0
-0.5
0.0
0.5
1.0
1.5

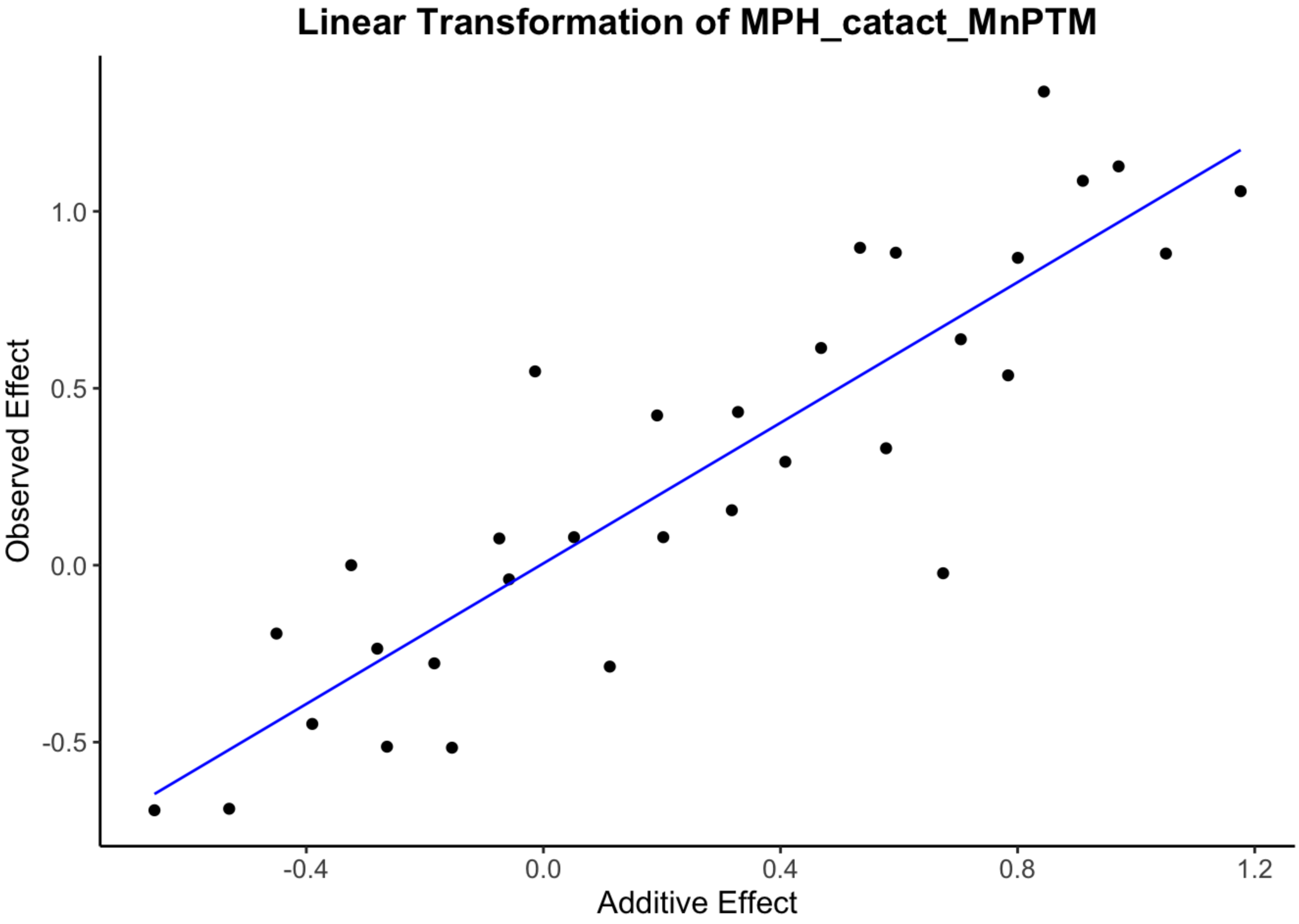
Linear Transformation of MPH_catact_MnPTM
Observed Effect
Additive Effect
1.0
0.5
0.0
-0.5
-0.4
0.0
0.4
0.8
1.2

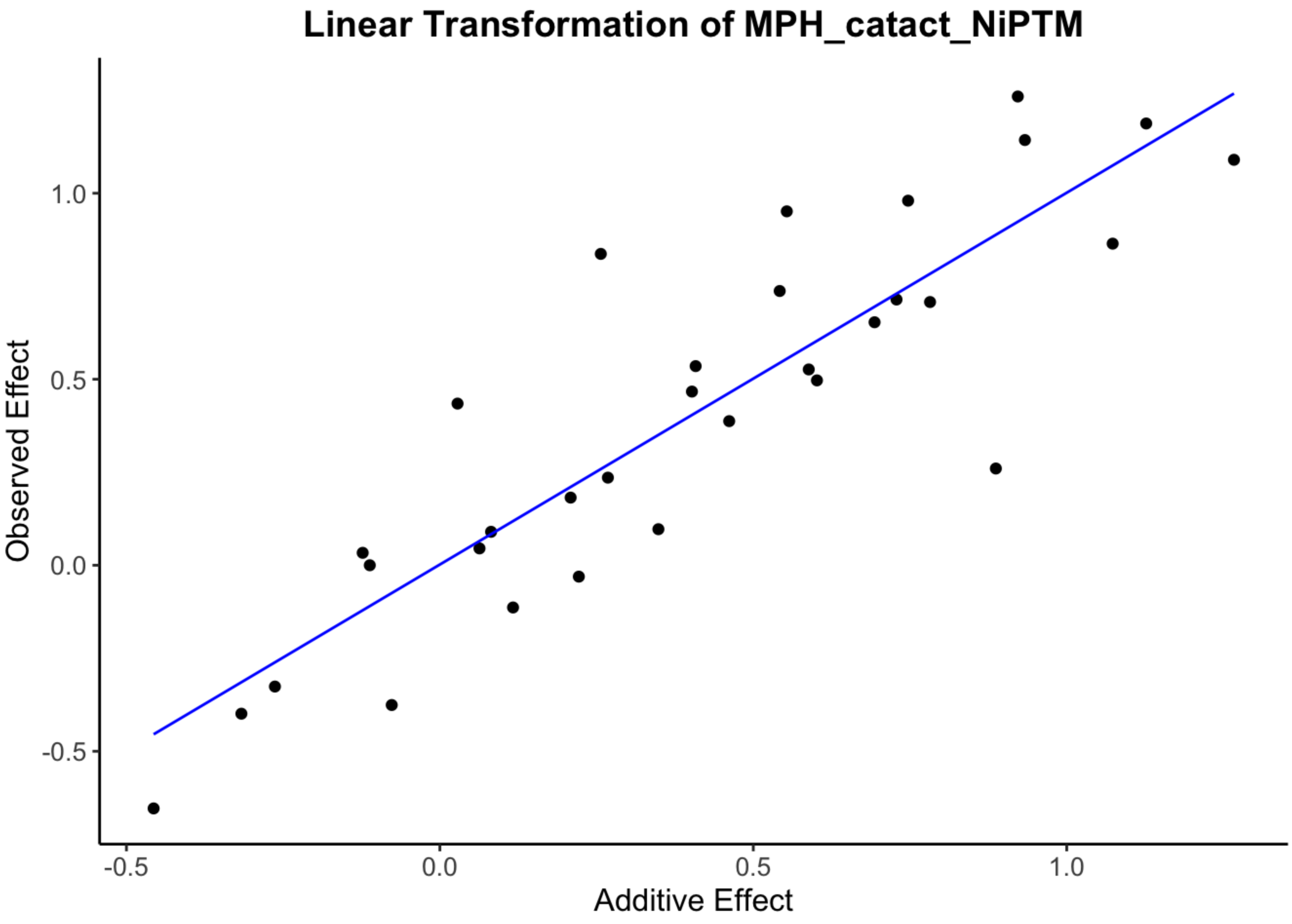
Linear Transformation of MPH_catact_NiPTM
Observed Effect
1.0
0.5
0.0
-0.5
-0.5
0.0
0.5
1.0
Additive Effect

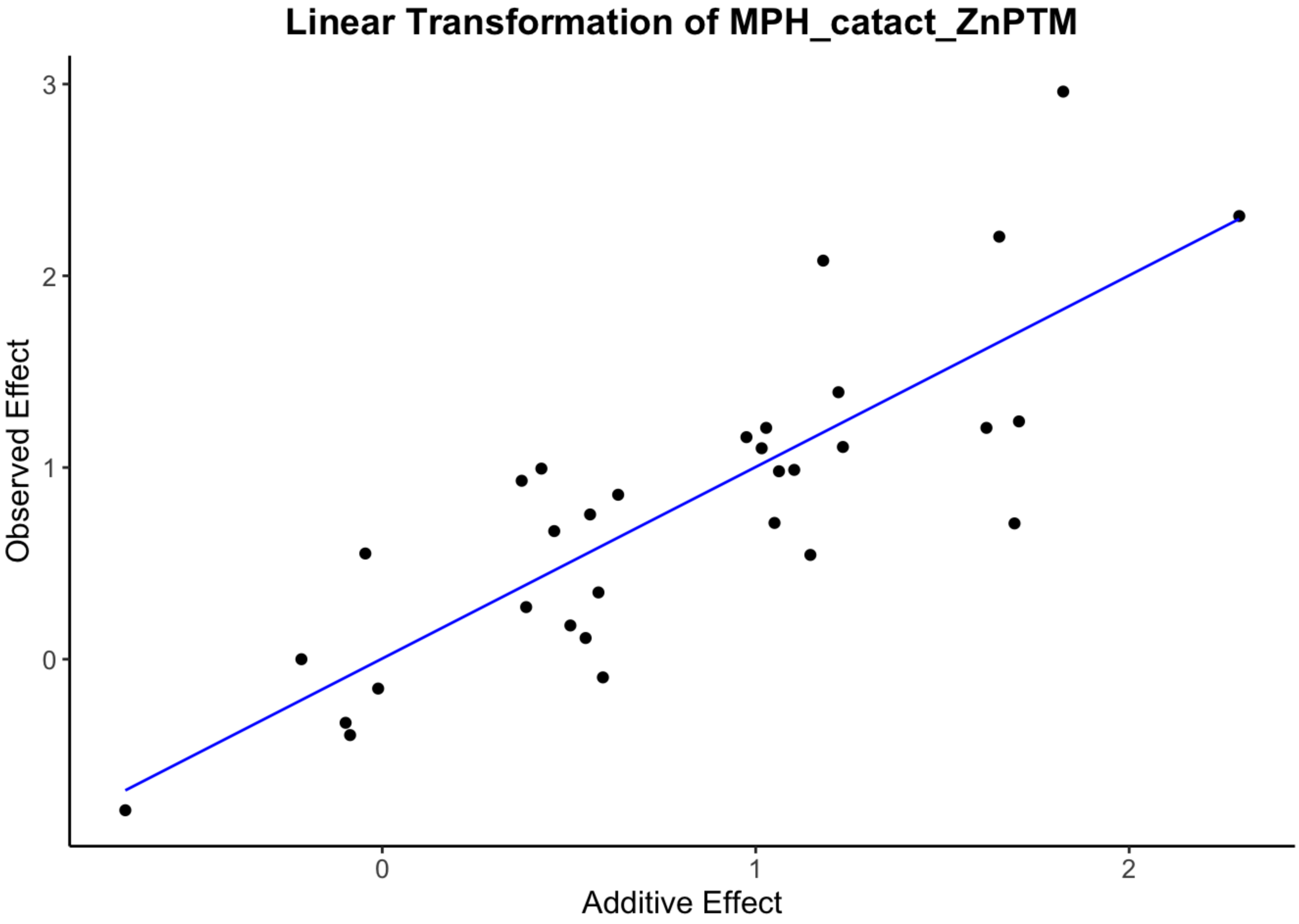
Linear Transformation of MPH_catact_ZnPTM
Observed Effect
3
2
1
0
0
1
2
Additive Effect

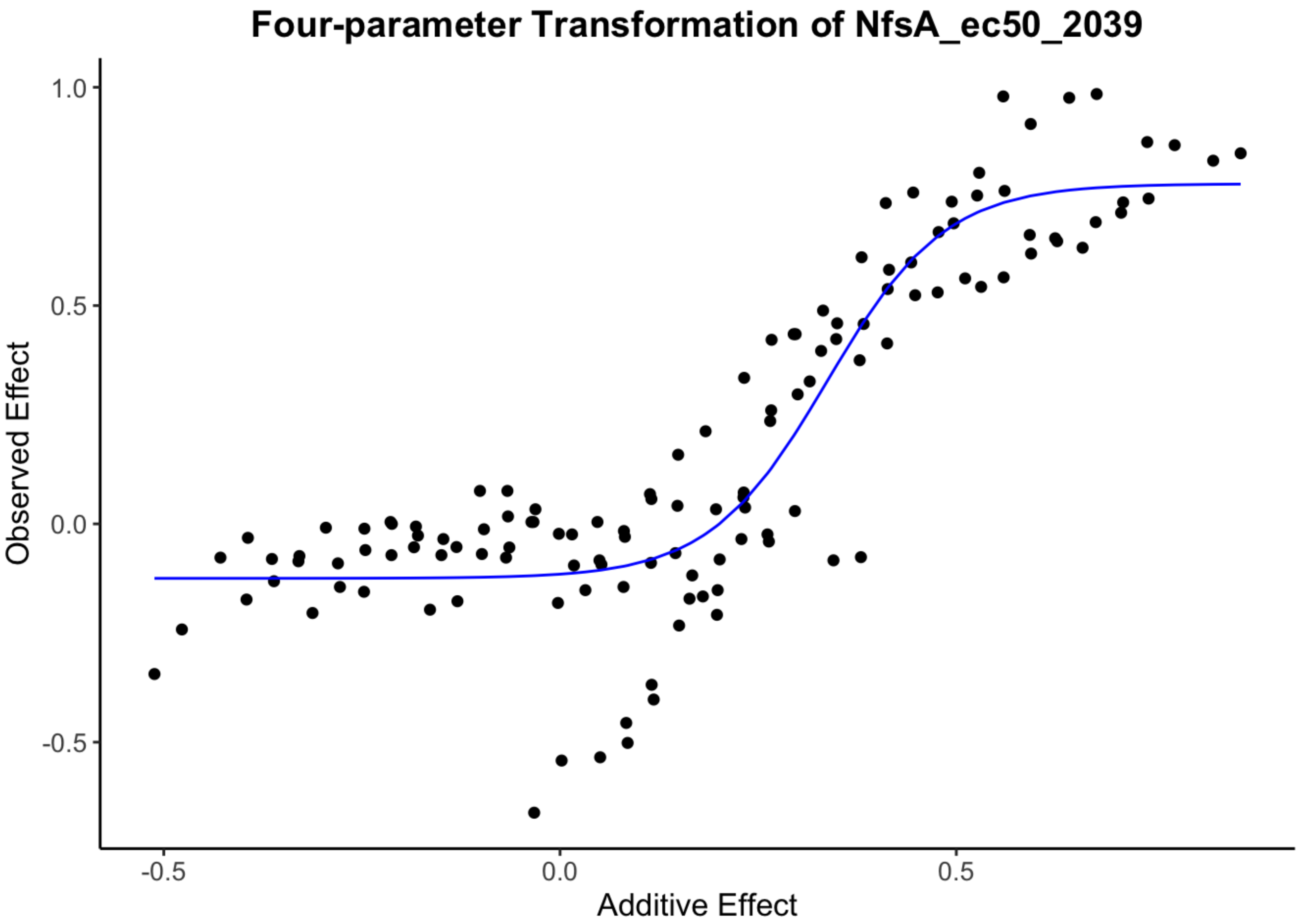
Four-parameter Transformation of NfsA_ec50_2039
Observed Effect
Additive Effect
1.0
0.5
0.0
-0.5
-0.5
0.0
0.5

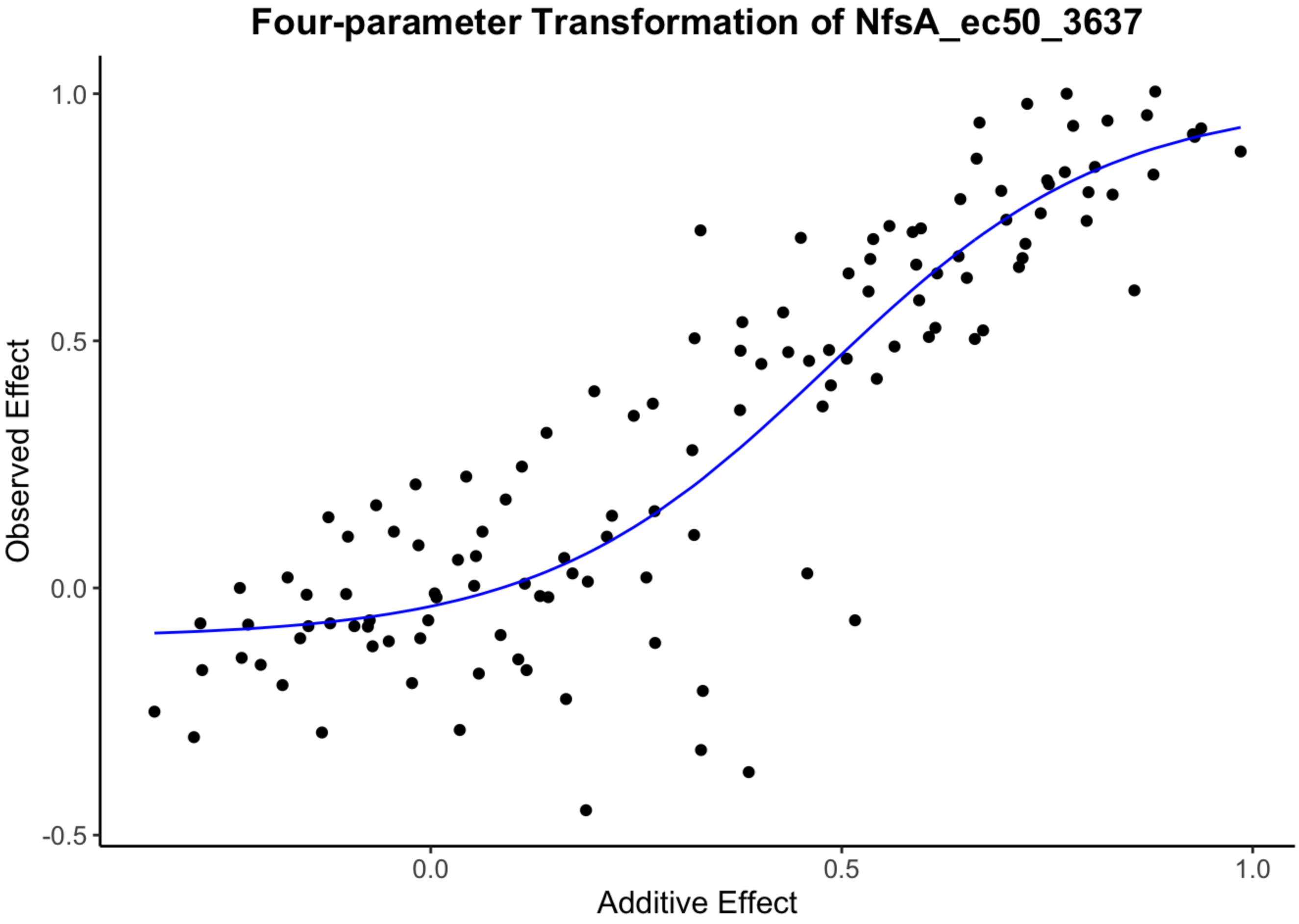
Four-parameter Transformation of NfsA_ec50_3637
Observed Effect
Additive Effect
1.0
0.5
0.0
-0.5
0.0
0.5
1.0

Four-parameter Transformation of OXA-48_ic50_CAZtraj1

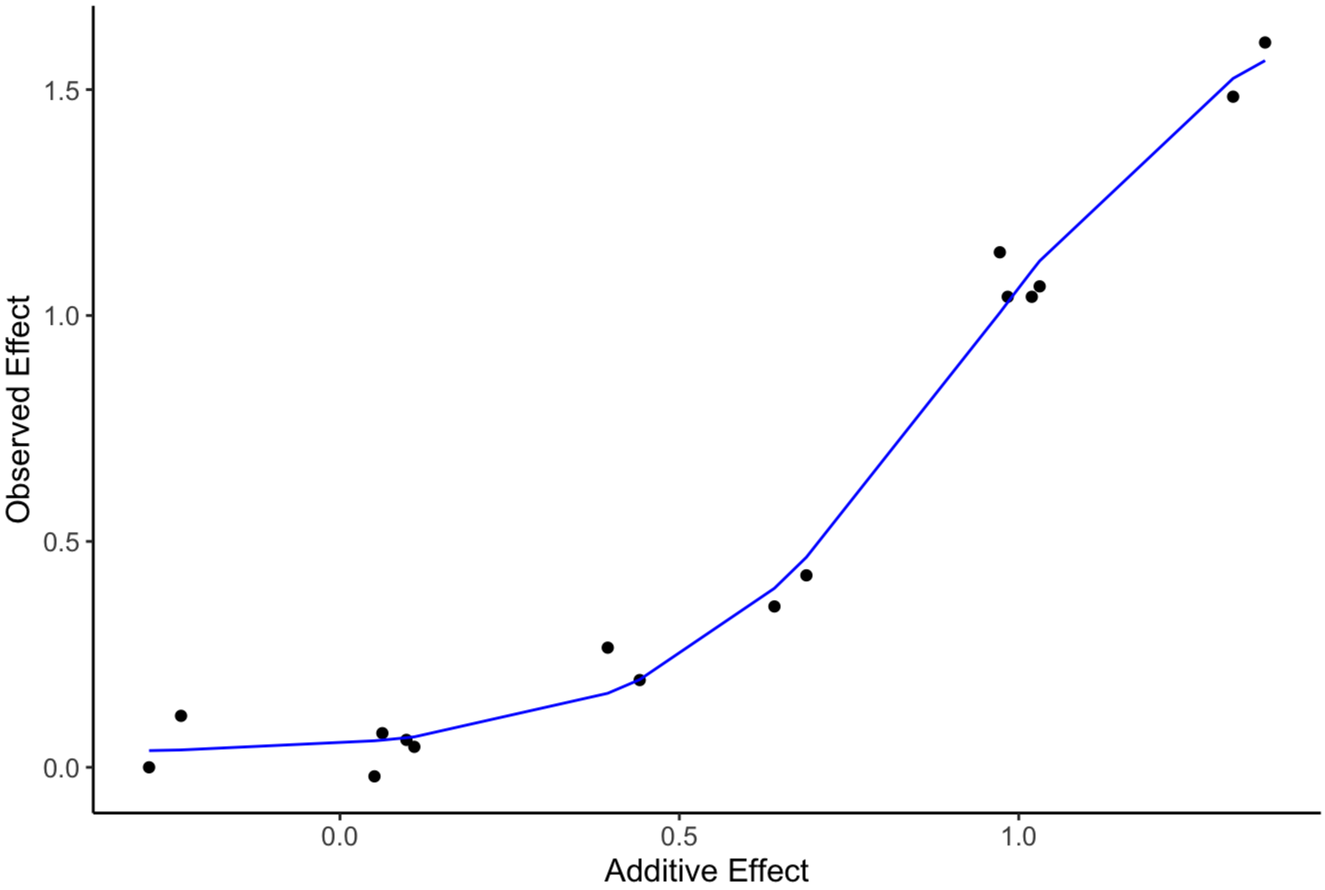

Four-parameter Transformation of OXA-48_ic50_CAZtraj2

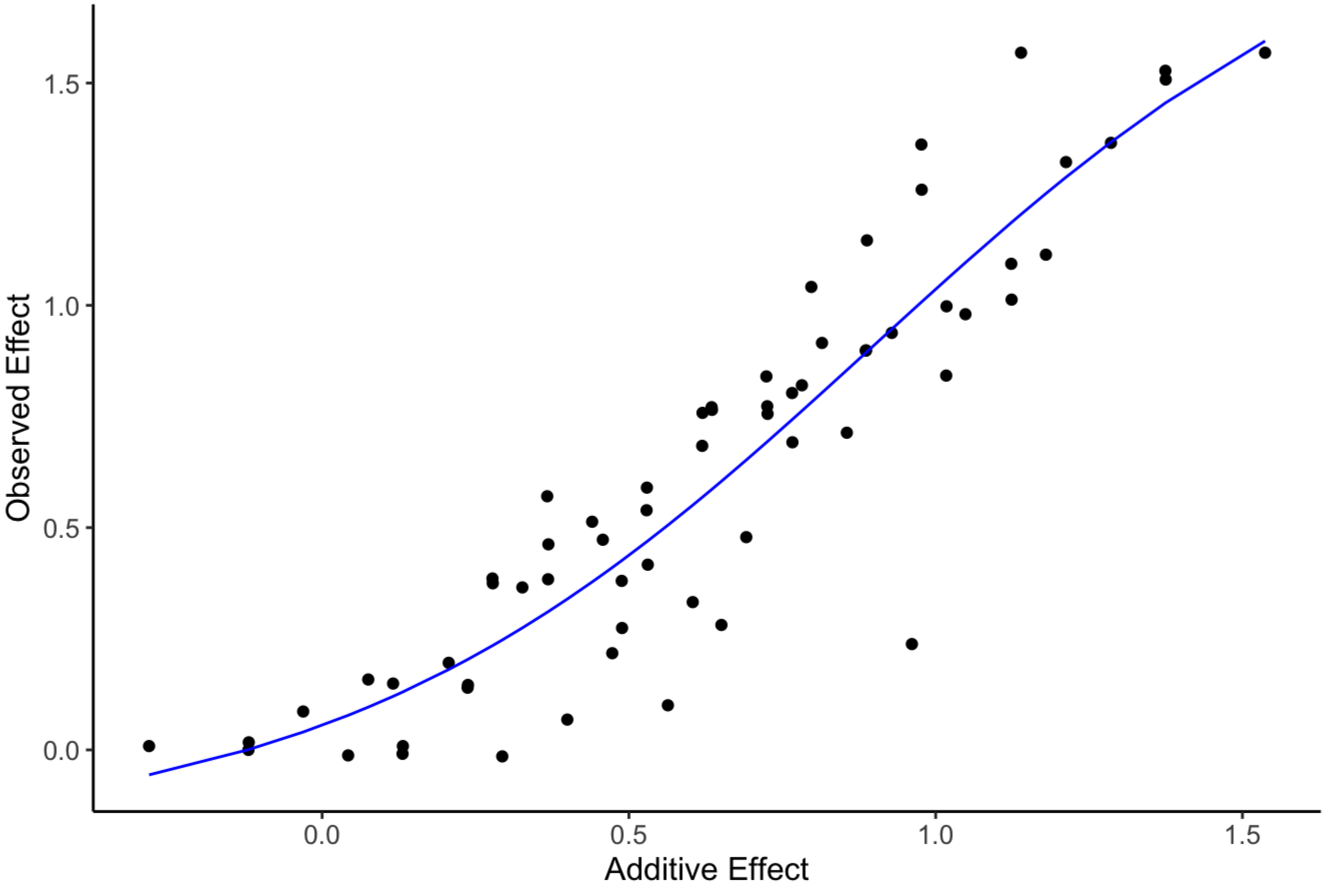

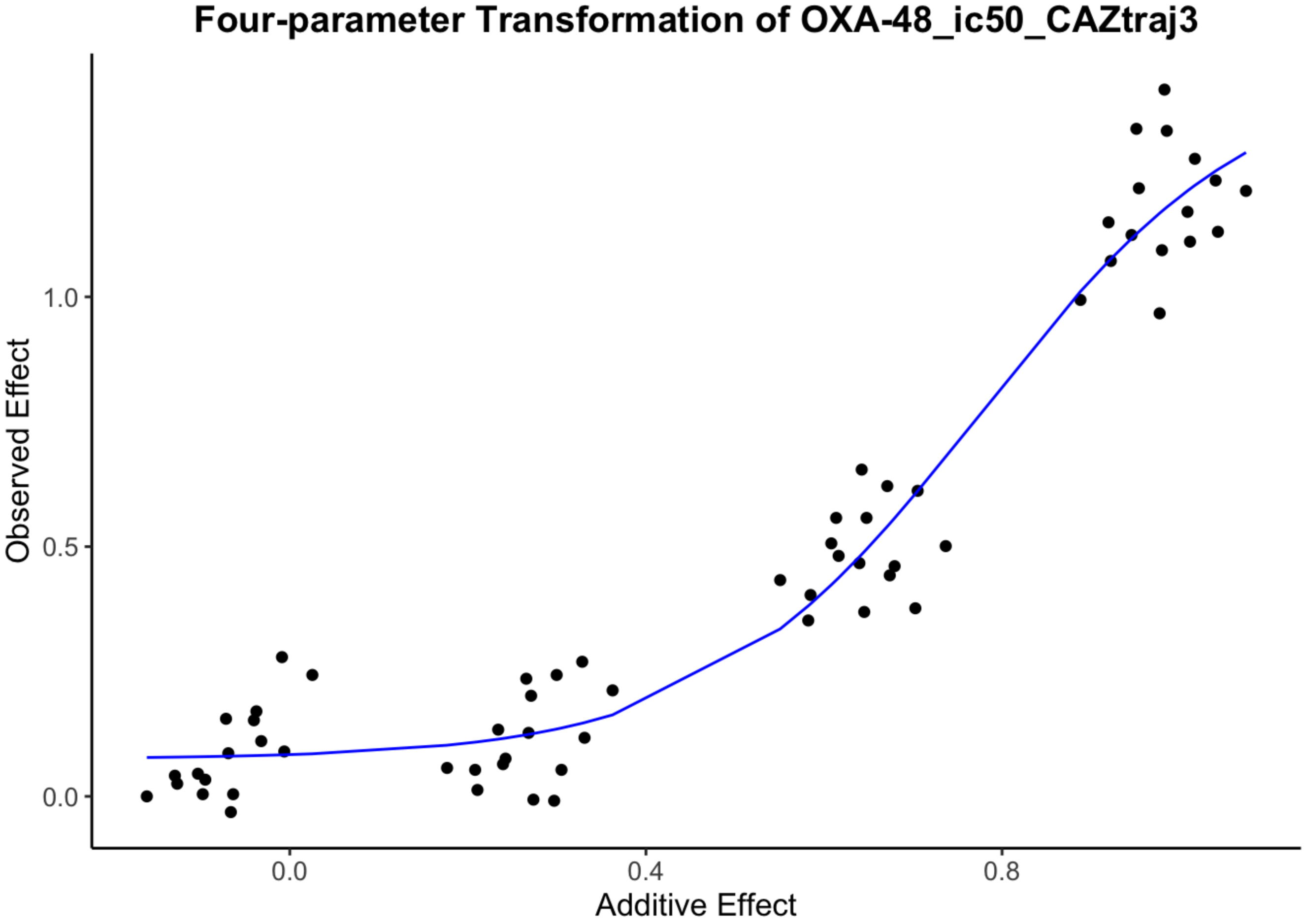
Four-parameter Transformation of OXA-48_ic50_CAZtraj3
Observed Effect
Additive Effect
0.0
0.5
1.0
0.0
0.4
0.8

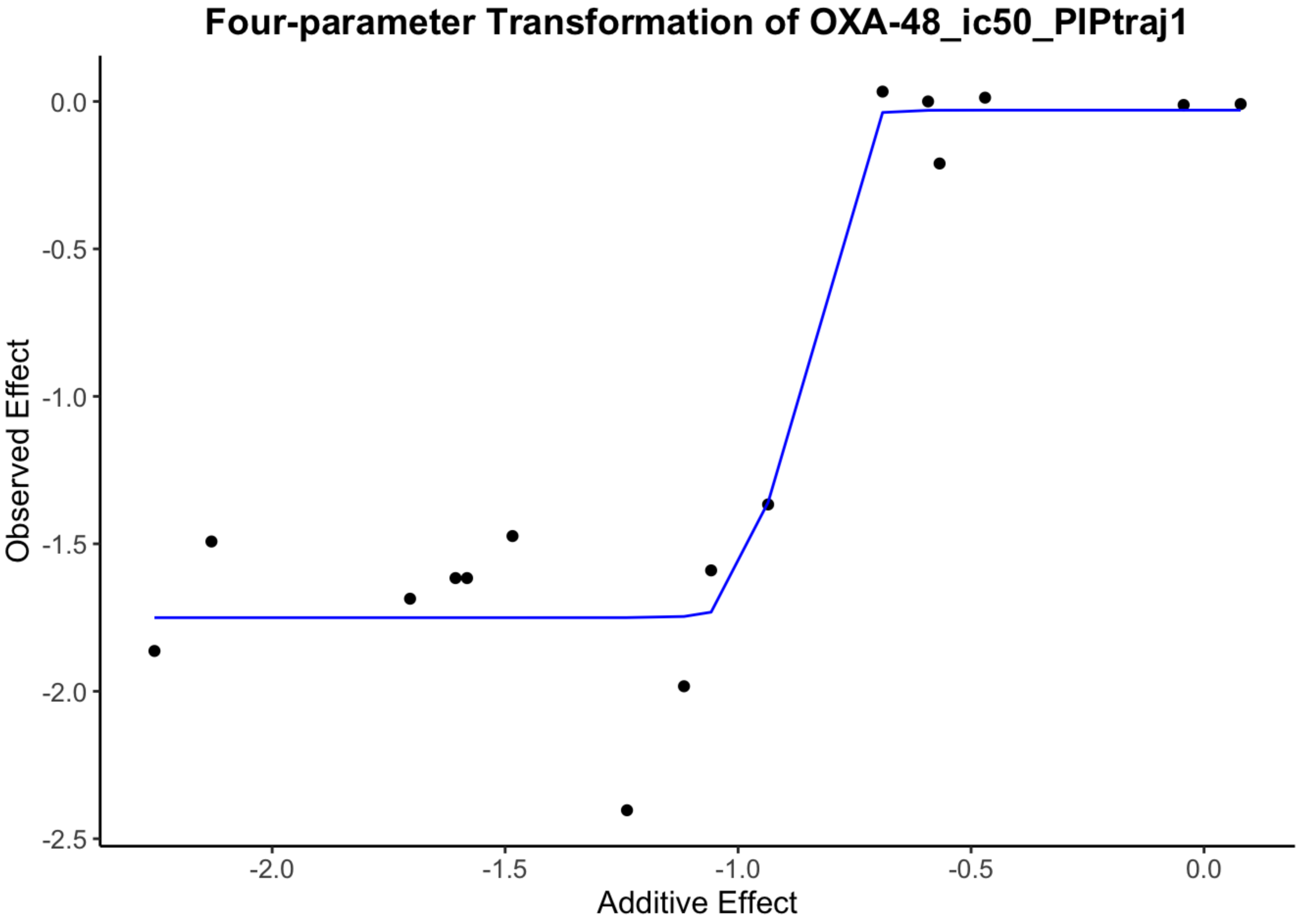
Four-parameter Transformation of OXA-48_ic50_PIPtraj1
Observed Effect
Additive Effect
0.0
-0.5
-1.0
-1.5
-2.0
-2.5
-2.0
-1.5
-1.0
-0.5
0.0

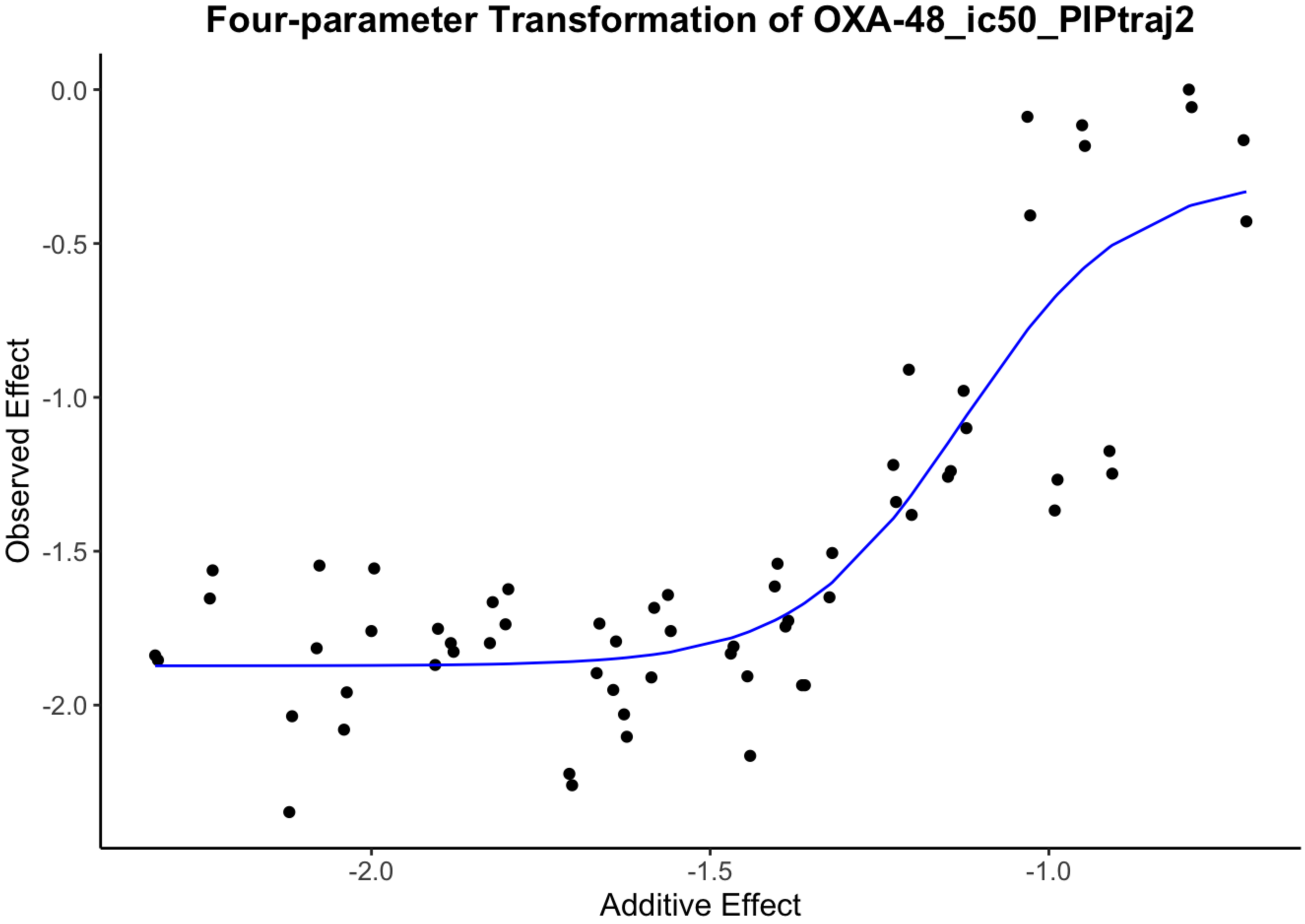
Four-parameter Transformation of OXA-48_ic50_PIPtraj2
Observed Effect
Additive Effect
0.0
-0.5
-1.0
-1.5
-2.0
-2.0
-1.5
-1.0

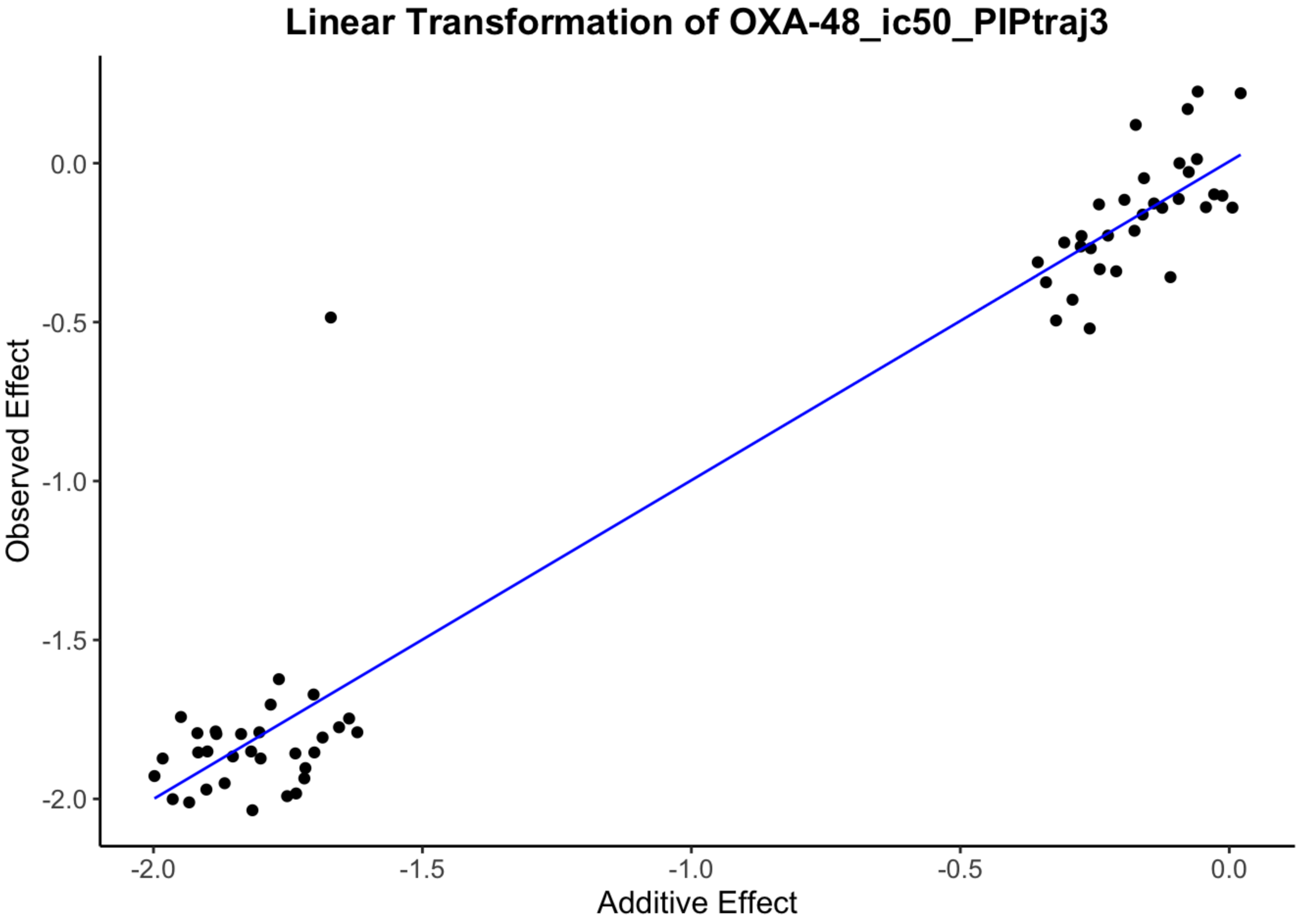
Linear Transformation of OXA-48_ic50_PIPtraj3
Observed Effect
Additive Effect
0.0
-0.5
-1.0
-1.5
-2.0
-2.0
-1.5
-1.0
-0.5
0.0

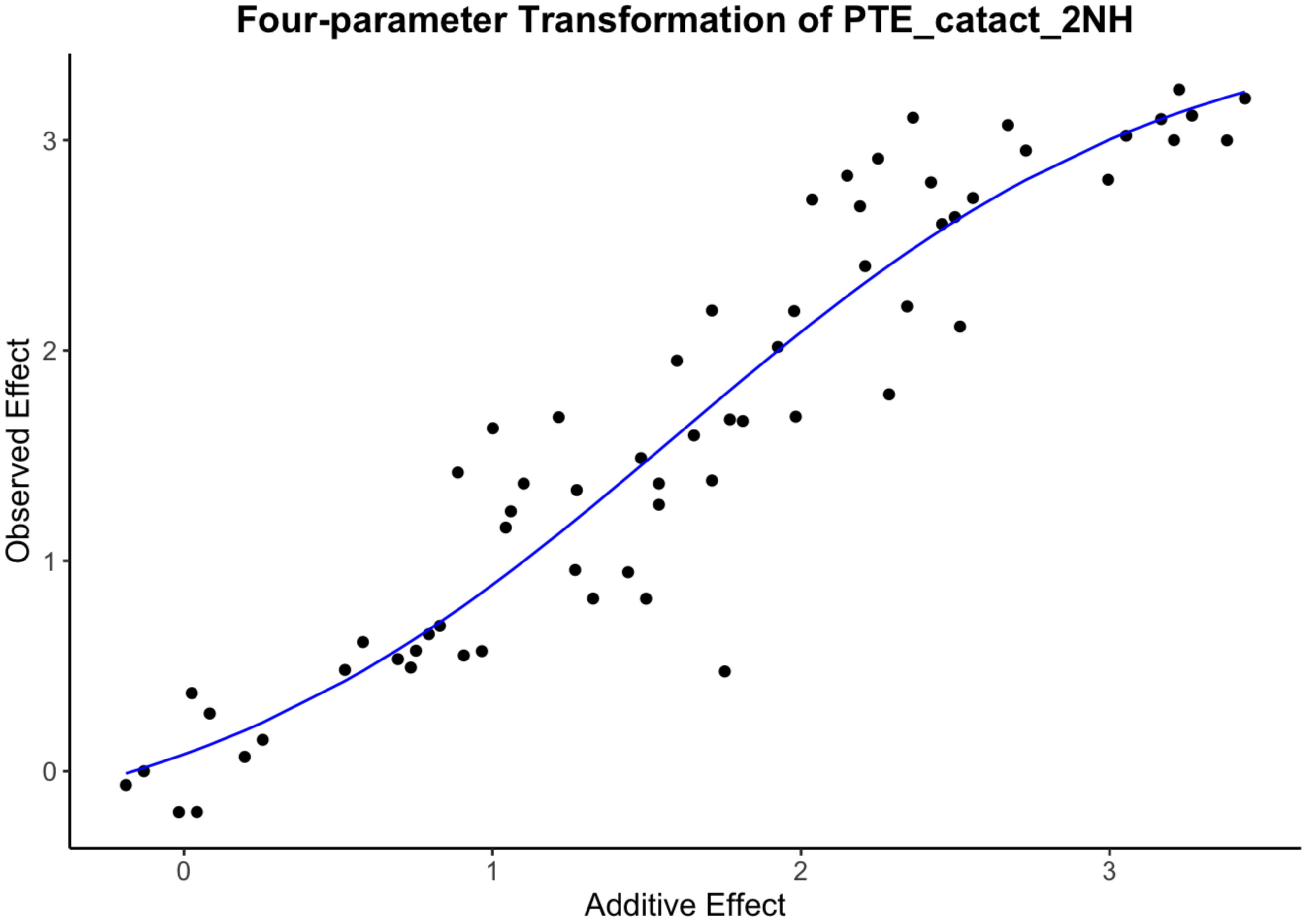

Four-parameter Transformation of PTE_catact_2NH
Observed Effect
Additive Effect
0
1
2
3

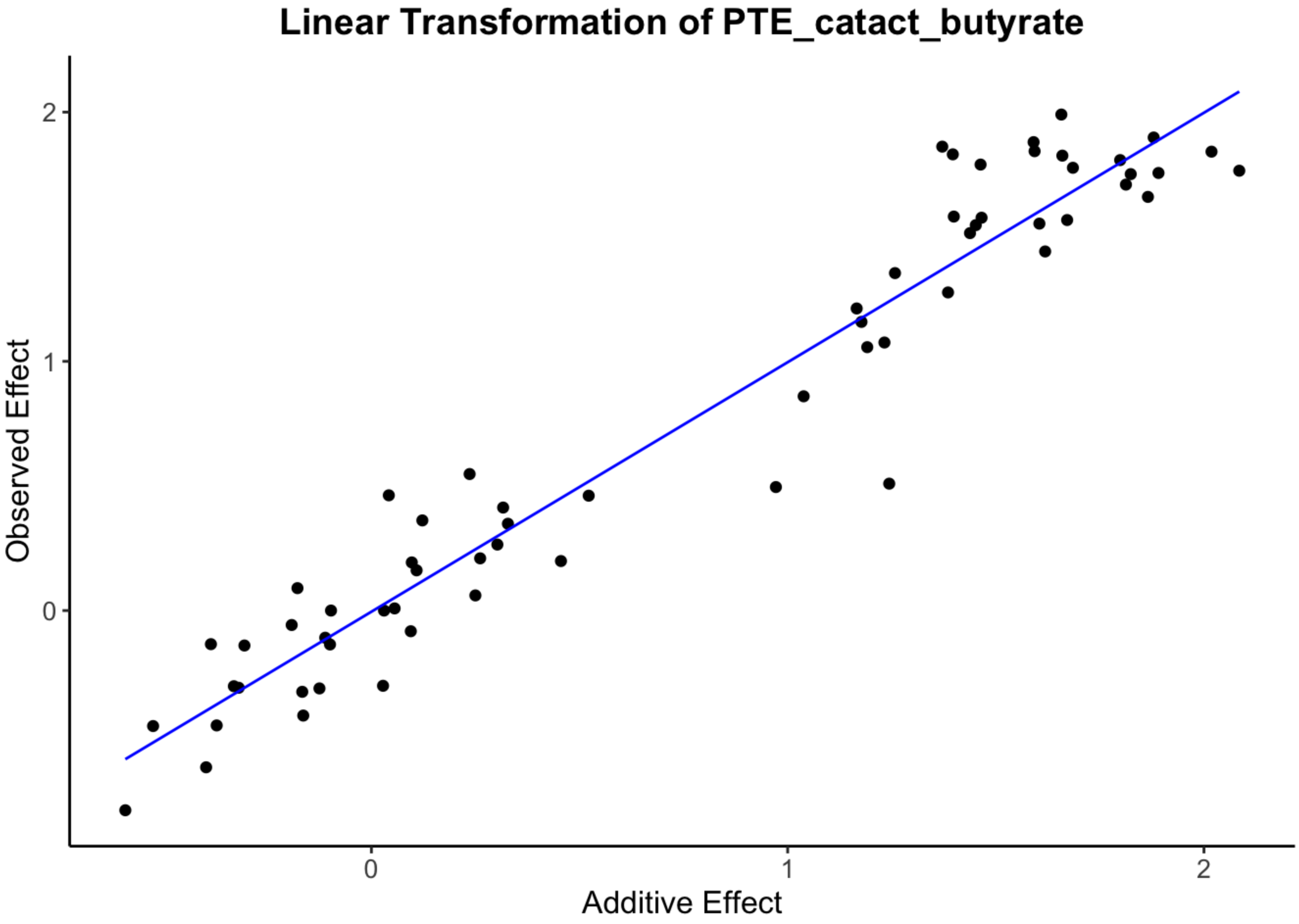

Linear Transformation of PTE_catact_butyrate
Observed Effect
Additive Effect
0
1
2

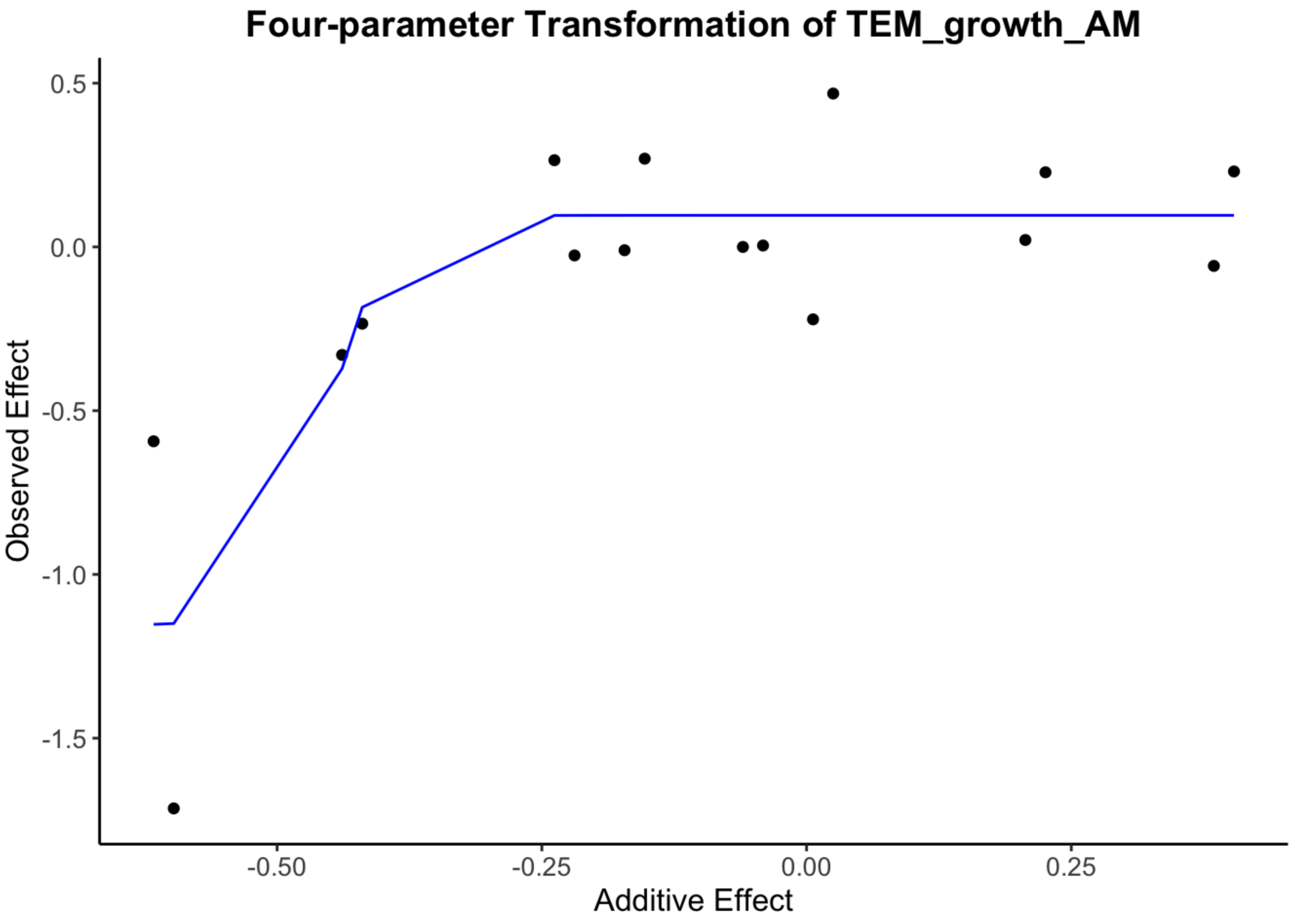

Four-parameter Transformation of TEM_growth_AM
Observed Effect
0.5
0.0
-0.5
-1.0
-1.5
-0.50
-0.25
0.00
0.25
Additive Effect

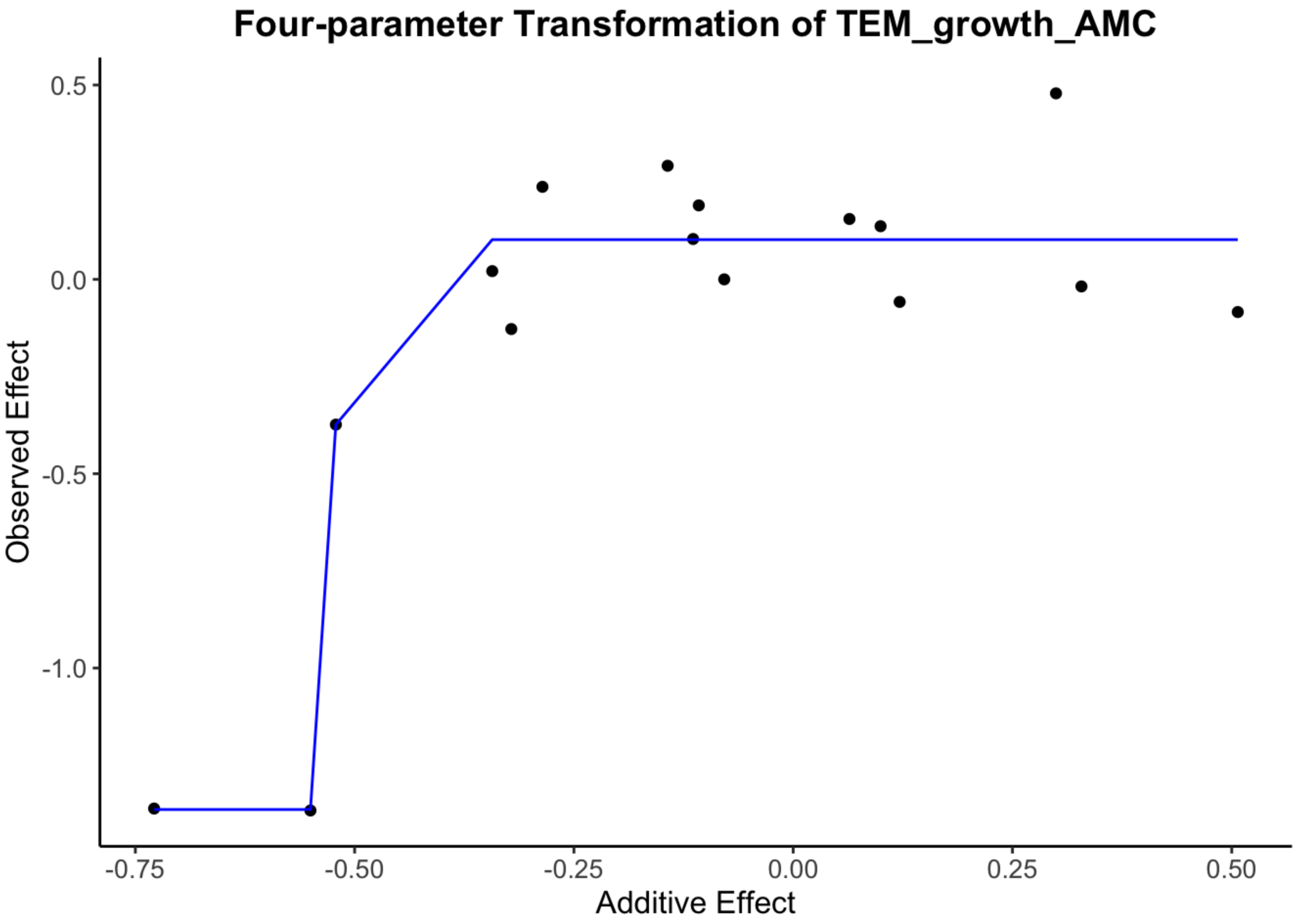

Four-parameter Transformation of TEM_growth_AMC
Observed Effect
0.5
0.0
-0.5
-1.0
-0.75
-0.50
-0.25
0.00
0.25
0.50
Additive Effect

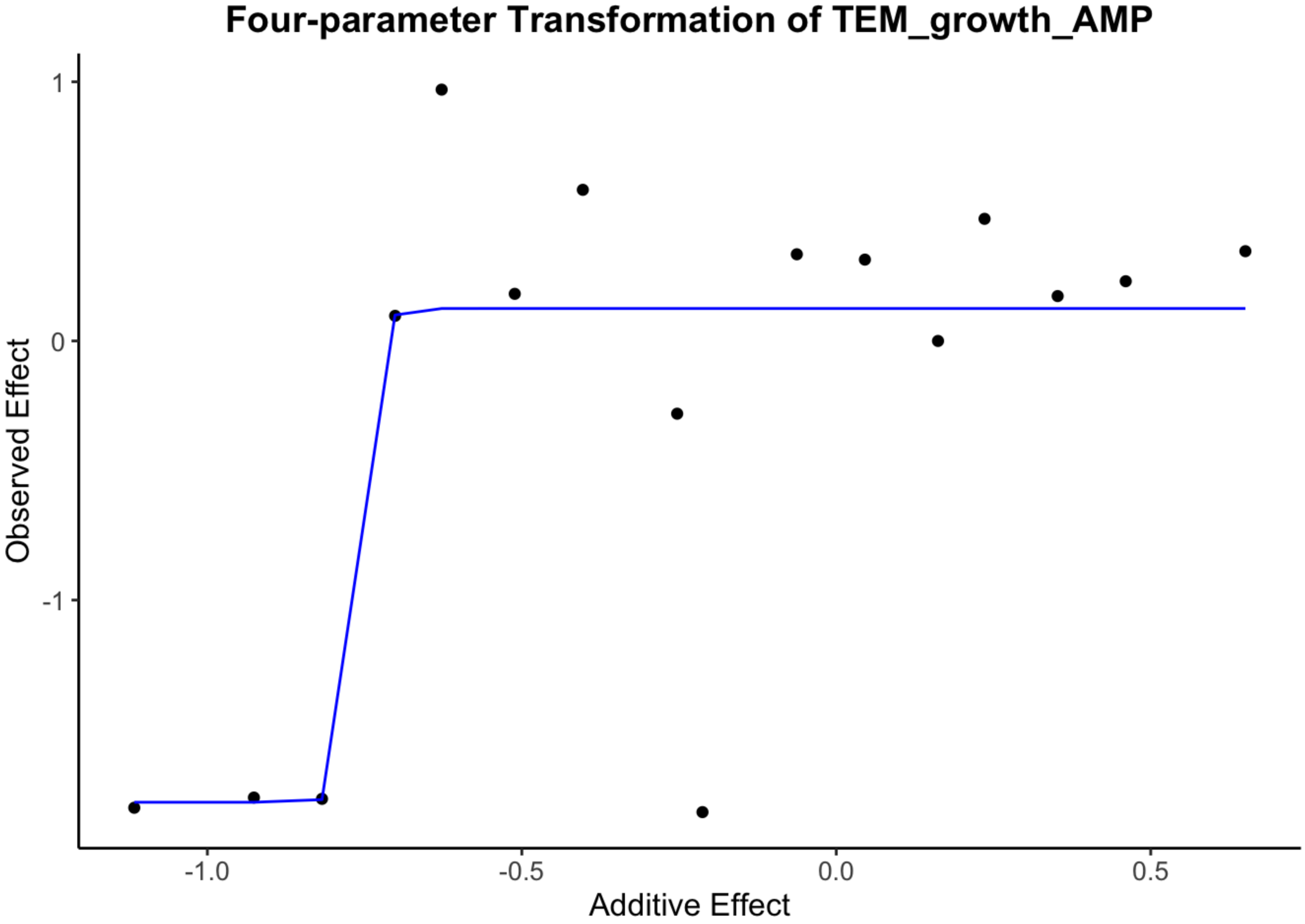
Four-parameter Transformation of TEM_growth_AMP
Observed Effect
Additive Effect
1
0
-1
-1.0
-0.5
0.0
0.5

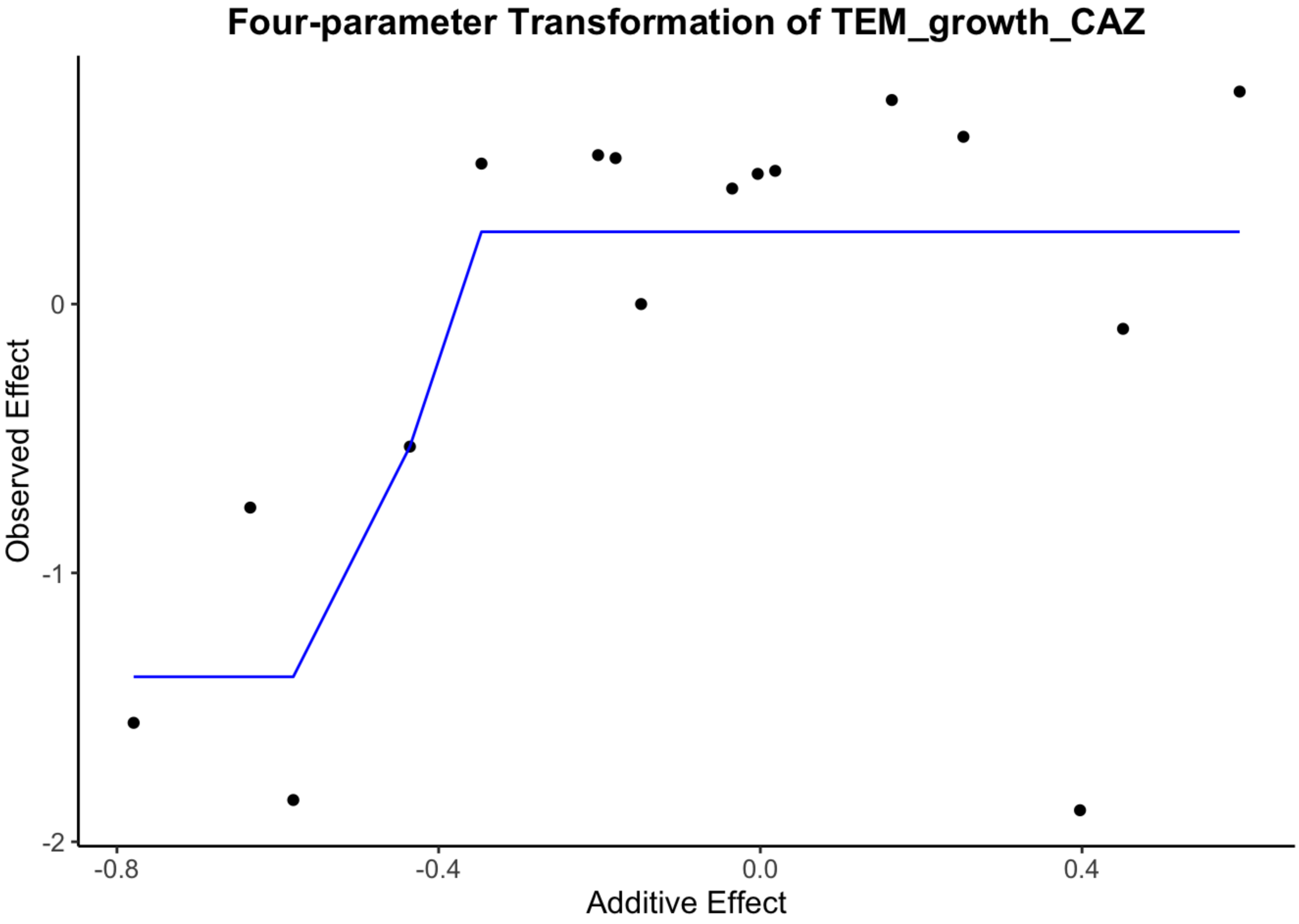
Four-parameter Transformation of TEM_growth_CAZ
Observed Effect
Additive Effect
0
-1
-2
-0.8
-0.4
0.0
0.4

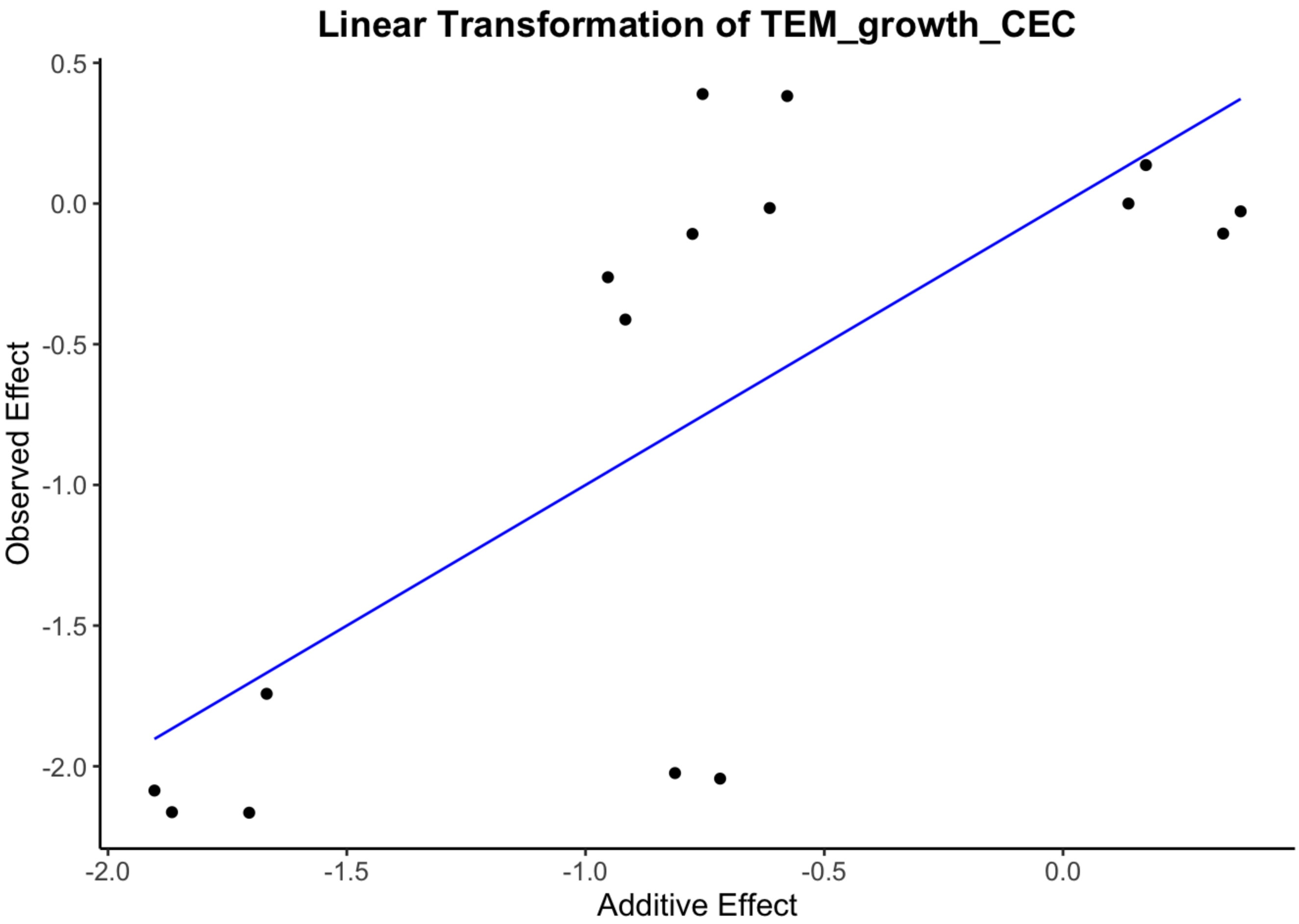
Linear Transformation of TEM_growth_CEC
Observed Effect
0.5
0.0
-0.5
-1.0
-1.5
-2.0
-2.0
-1.5
-1.0
-0.5
0.0
Additive Effect

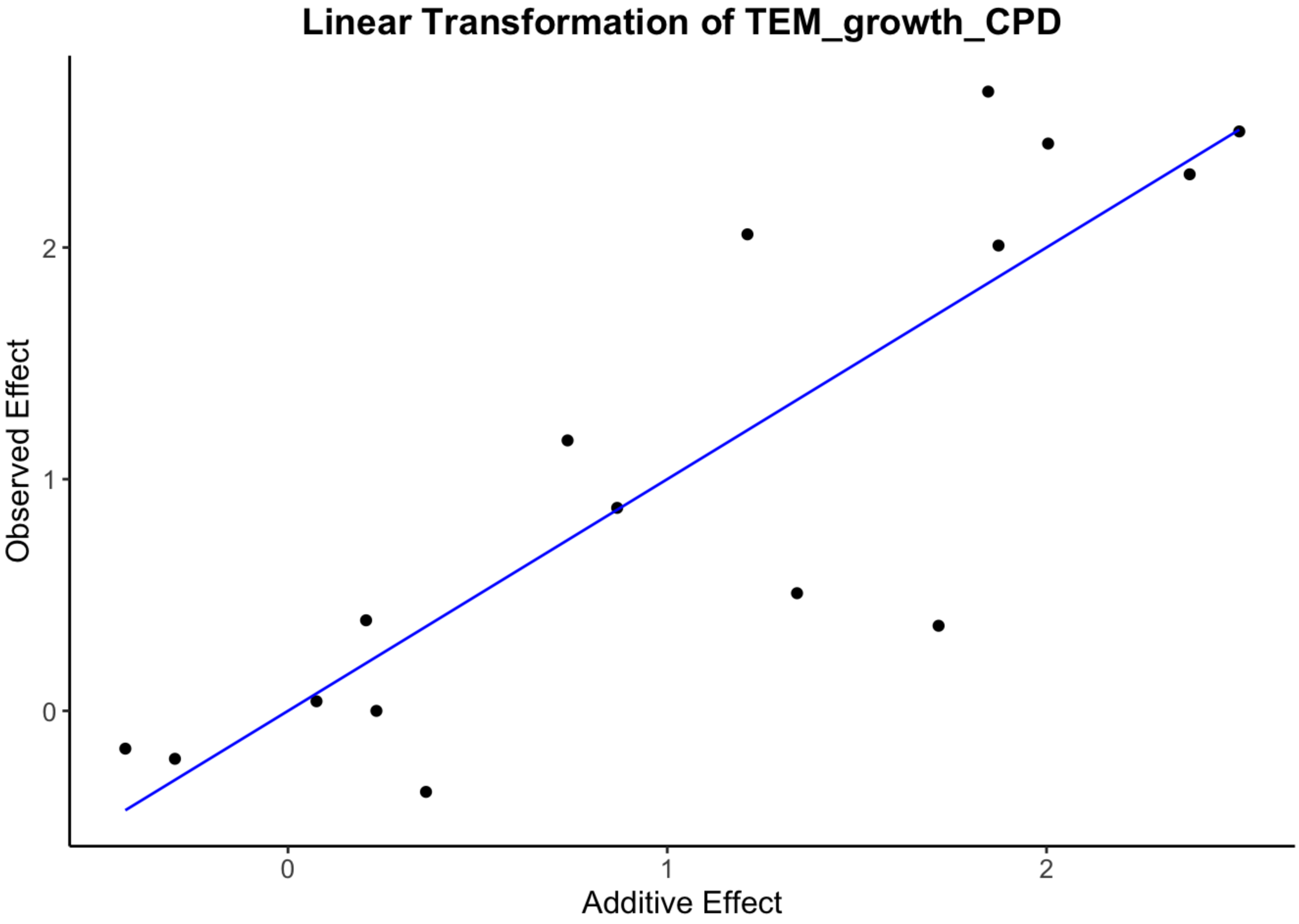
Linear Transformation of TEM_growth_CPD
Observed Effect
2
1
0
0
1
2
Additive Effect

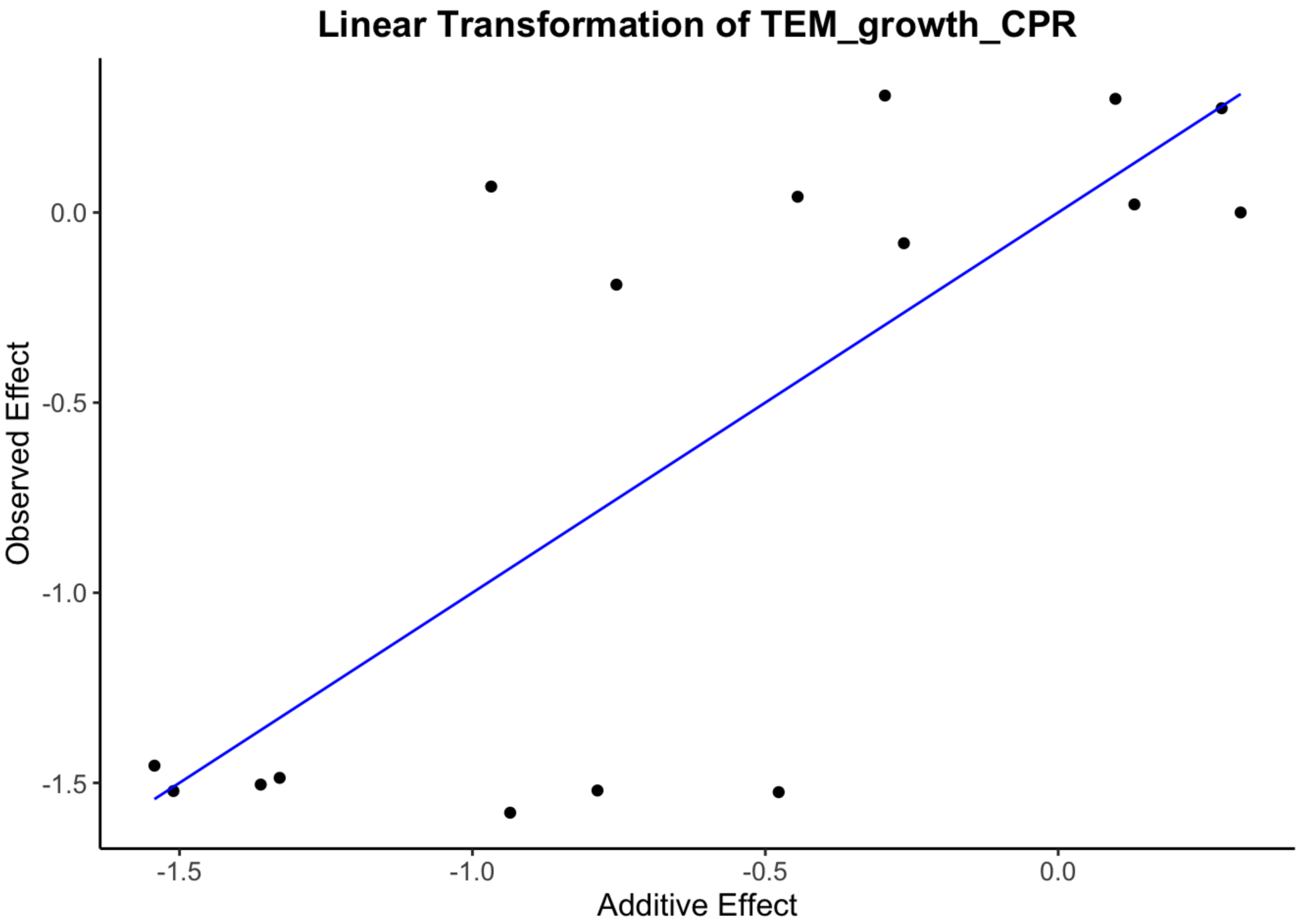
Linear Transformation of TEM_growth_CPR
Observed Effect
0.0
-0.5
-1.0
-1.5
-1.5
-1.0
-0.5
0.0
Additive Effect

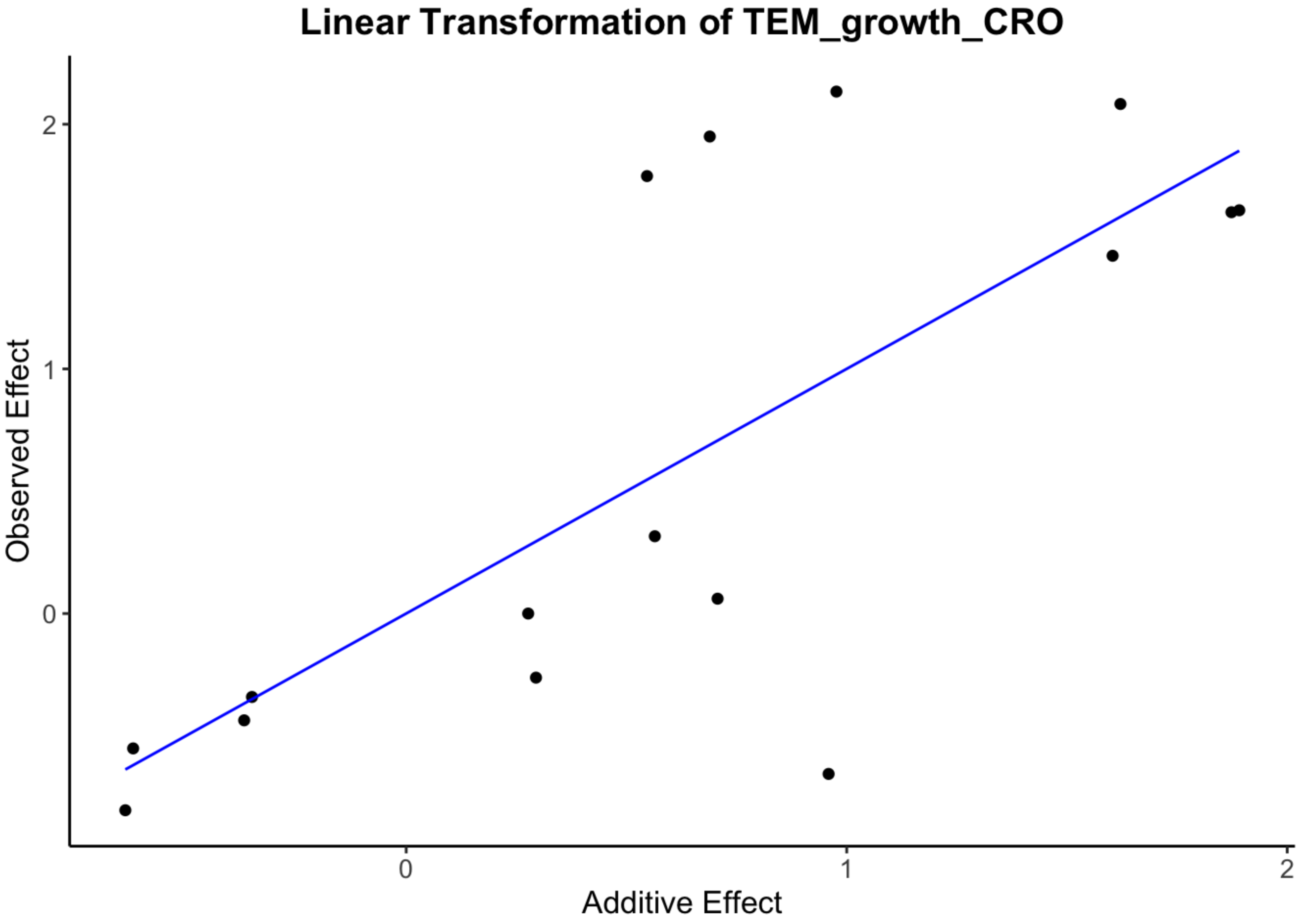
Linear Transformation of TEM_growth_CRO
Observed Effect
2
1
0
0
1
2
Additive Effect

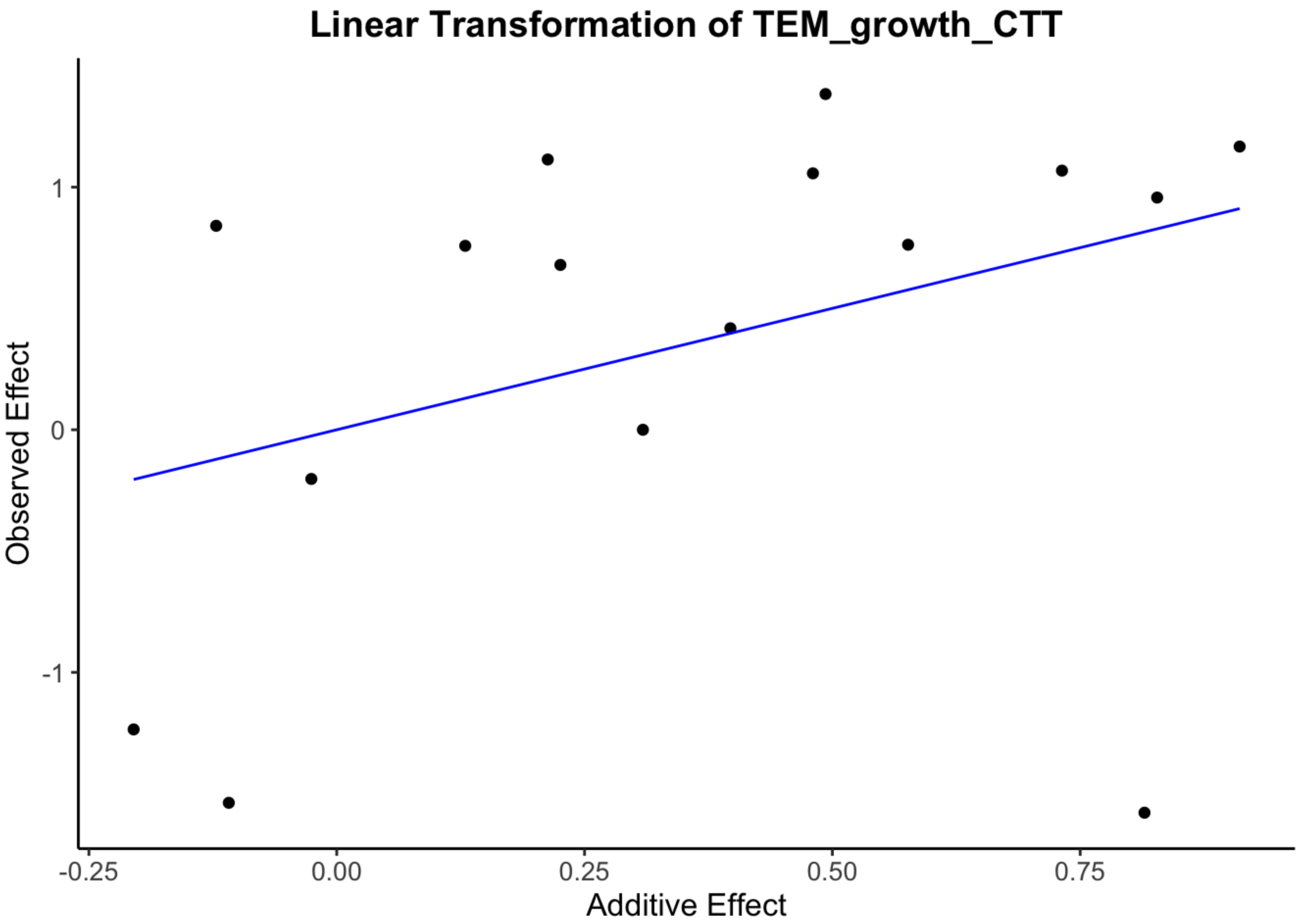
Linear Transformation of TEM_growth_CTT
Observed Effect
Additive Effect
1
0
-1
-0.25
0.00
0.25
0.50
0.75

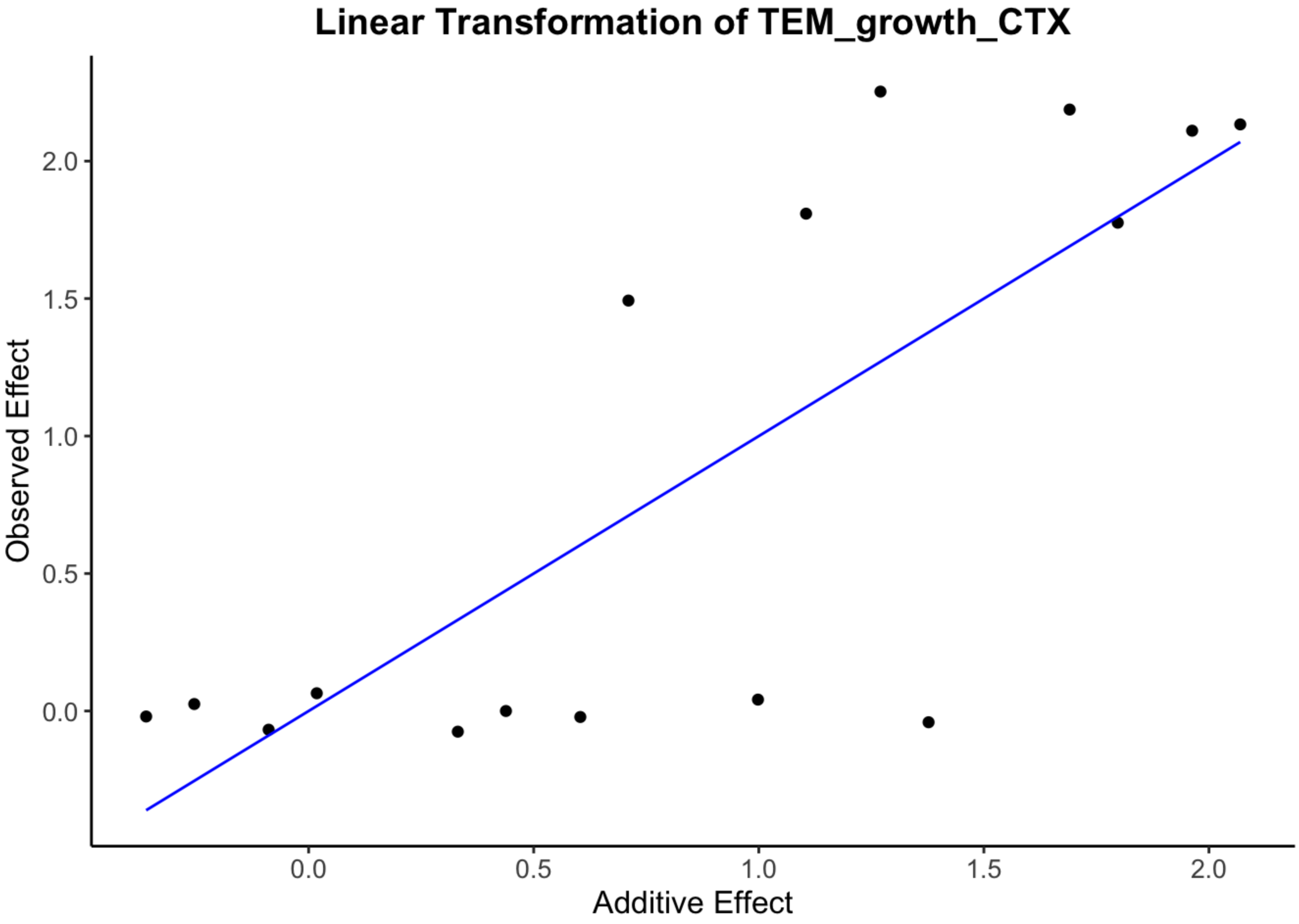
Linear Transformation of TEM_growth_CTX
Observed Effect
Additive Effect
2.0
1.5
1.0
0.5
0.0
0.0
0.5
1.0
1.5
2.0

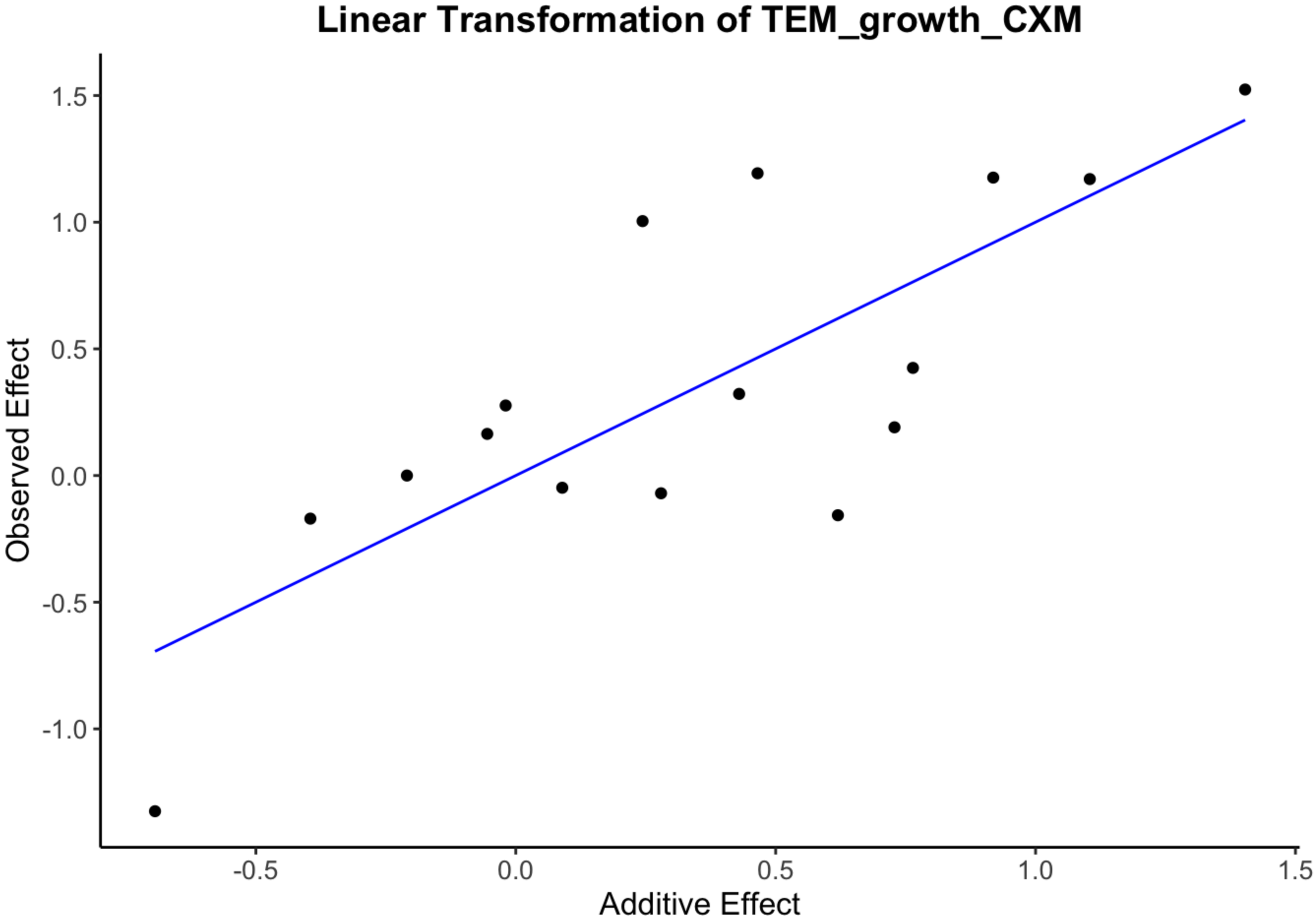


```
## Error in nlsModel(formula, mf, start, wts) :
##   singular gradient matrix at initial parameter estimates
```

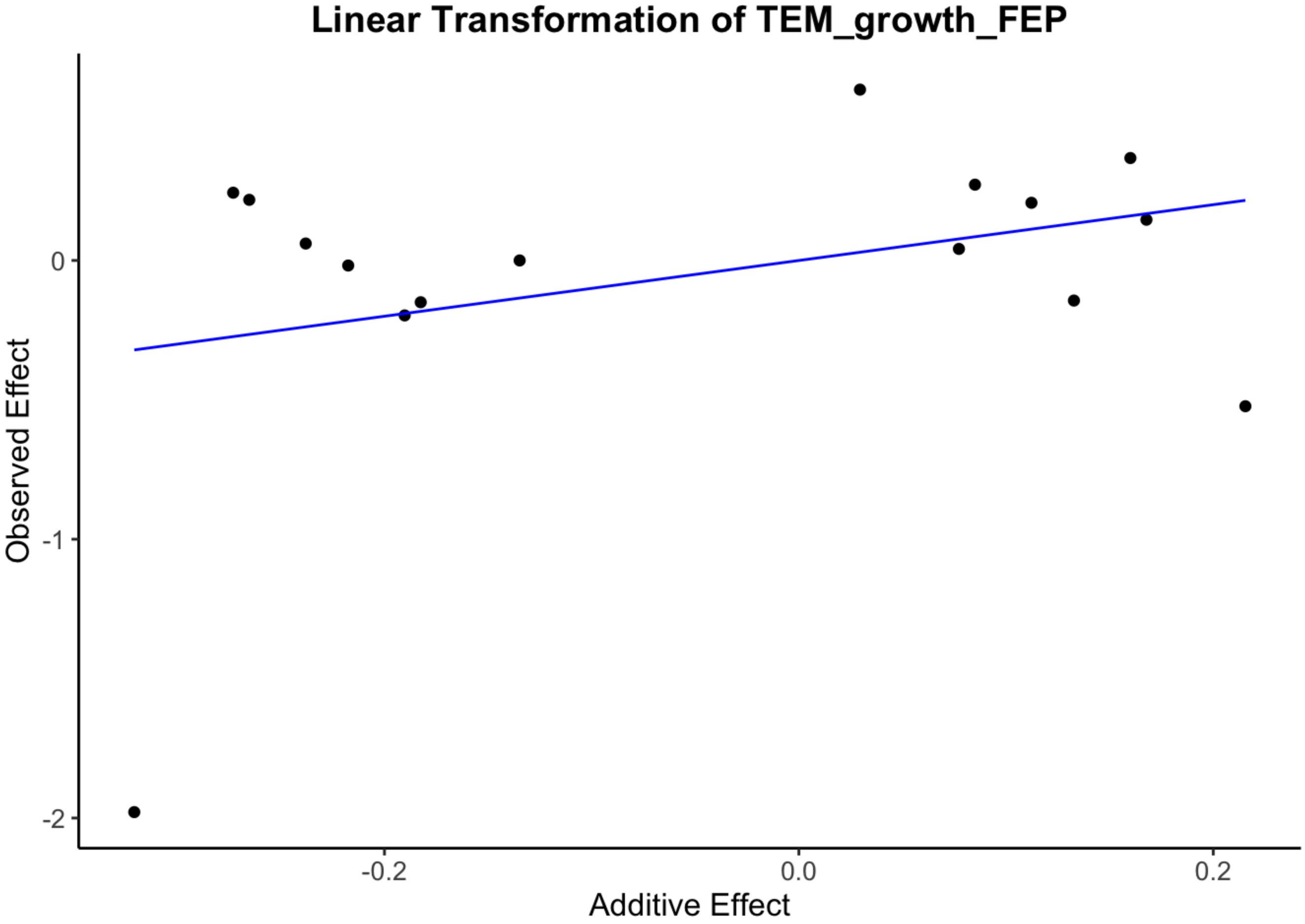
Linear Transformation of TEM_growth_FEP
Observed Effect
0
-1
-2
-0.2
0.0
0.2
Additive Effect

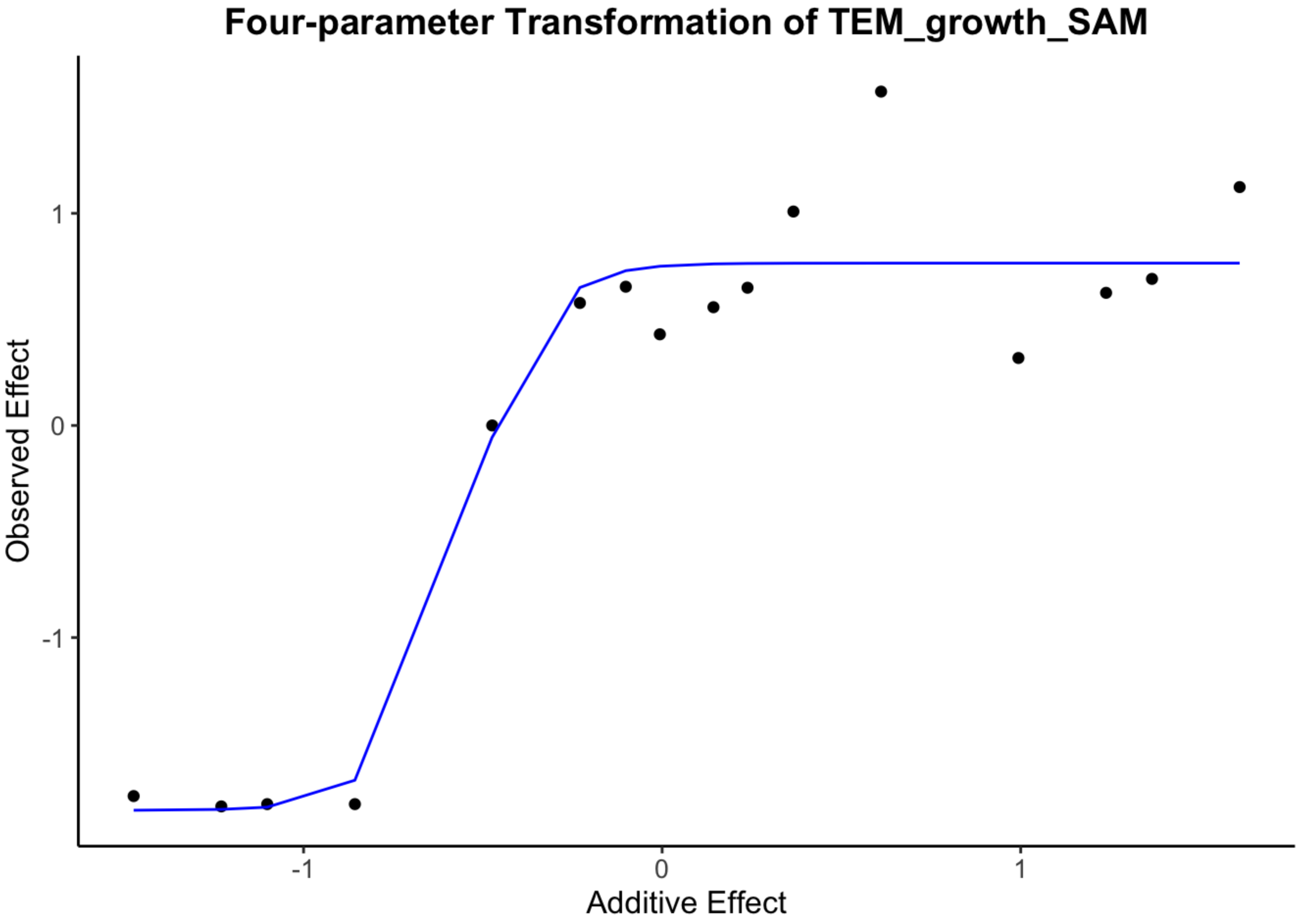
Four-parameter Transformation of TEM_growth_SAM
Observed Effect
1
0
-1
-1
0
1
Additive Effect

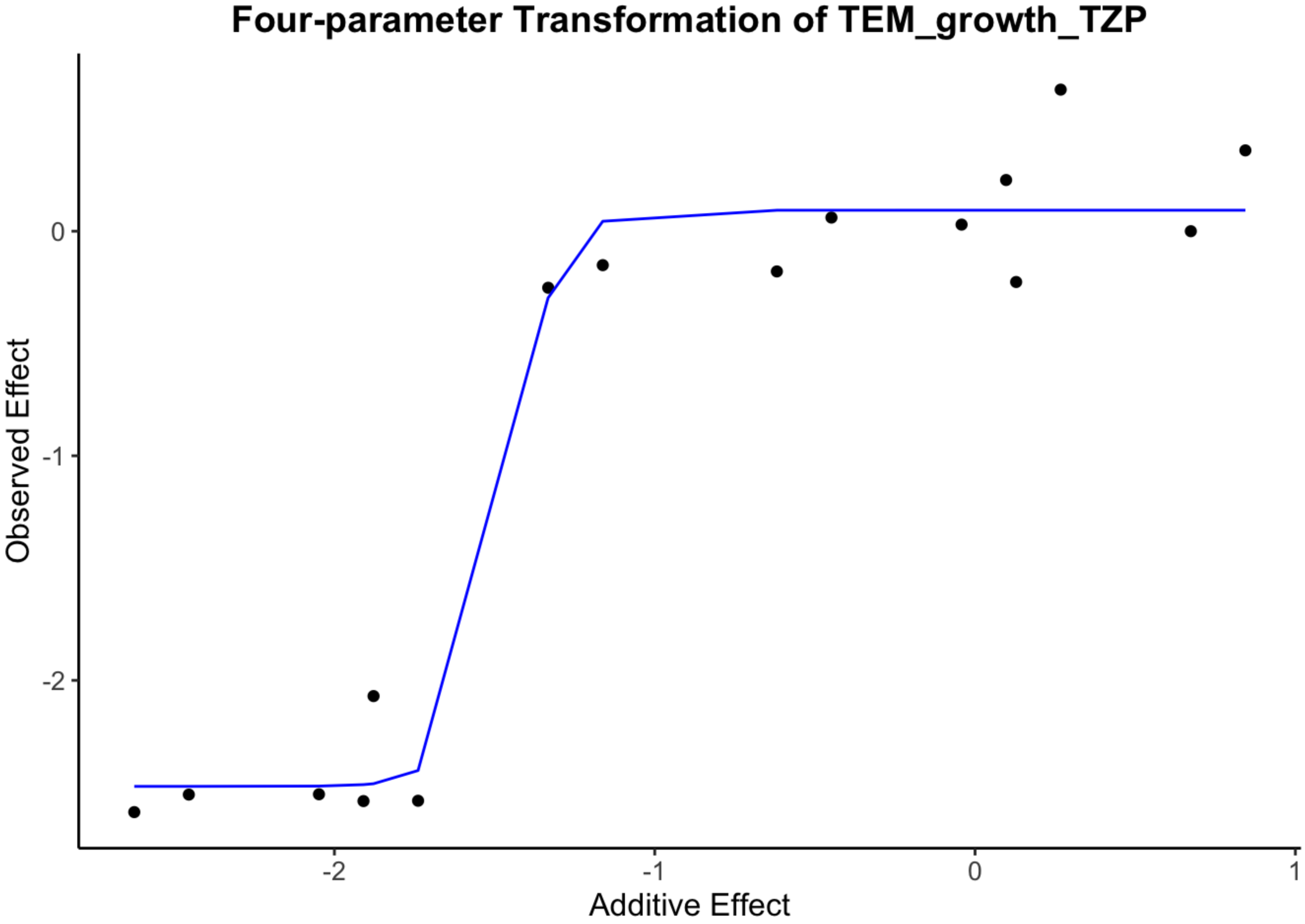

Four-parameter Transformation of TEM_growth_TZP
Observed Effect
0
-1
-2
-2
-1
0
1
Additive Effect

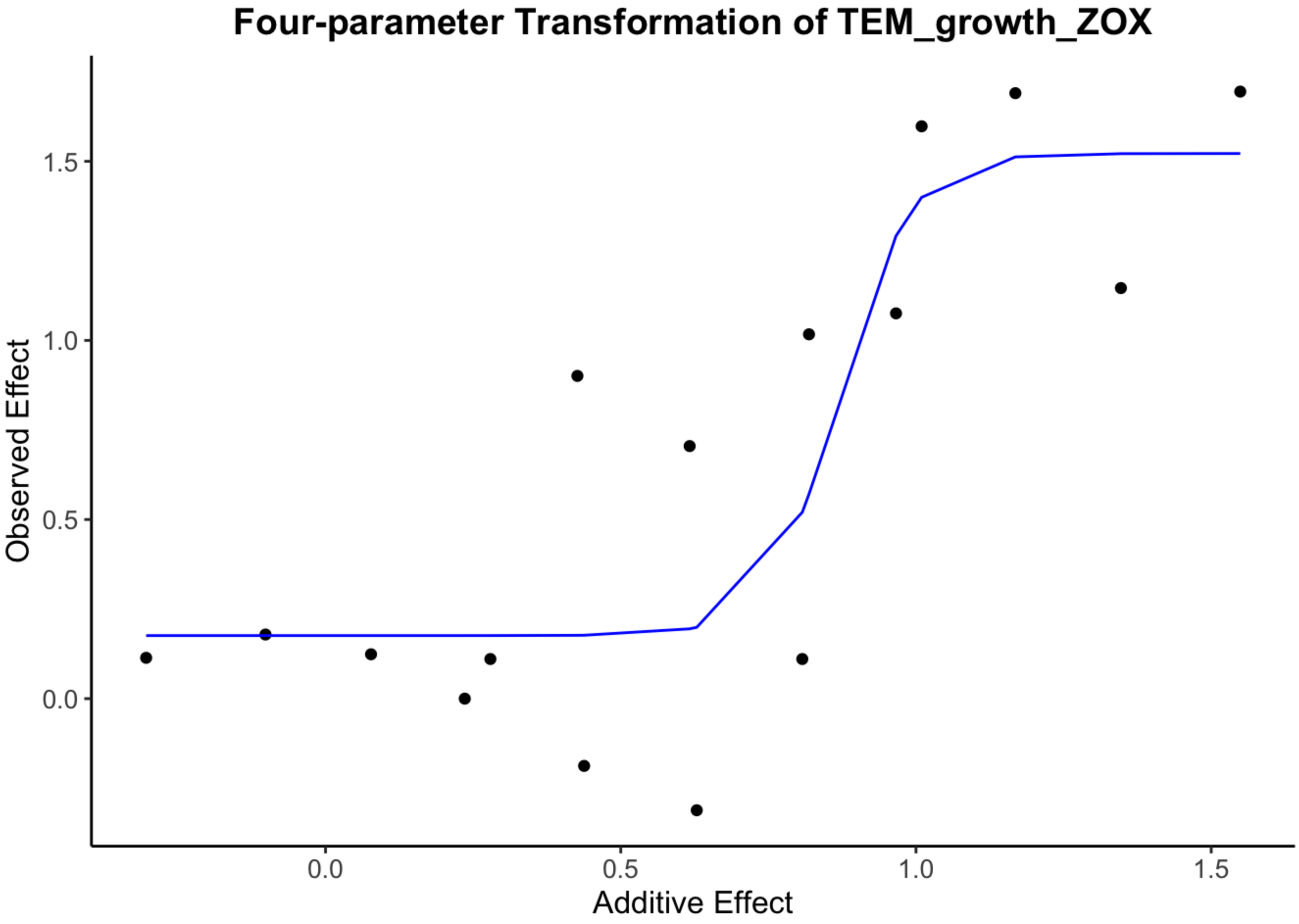

Four-parameter Transformation of TEM_growth_ZOX
Observed Effect
1.5
1.0
0.5
0.0
0.0
0.5
1.0
1.5
Additive Effect

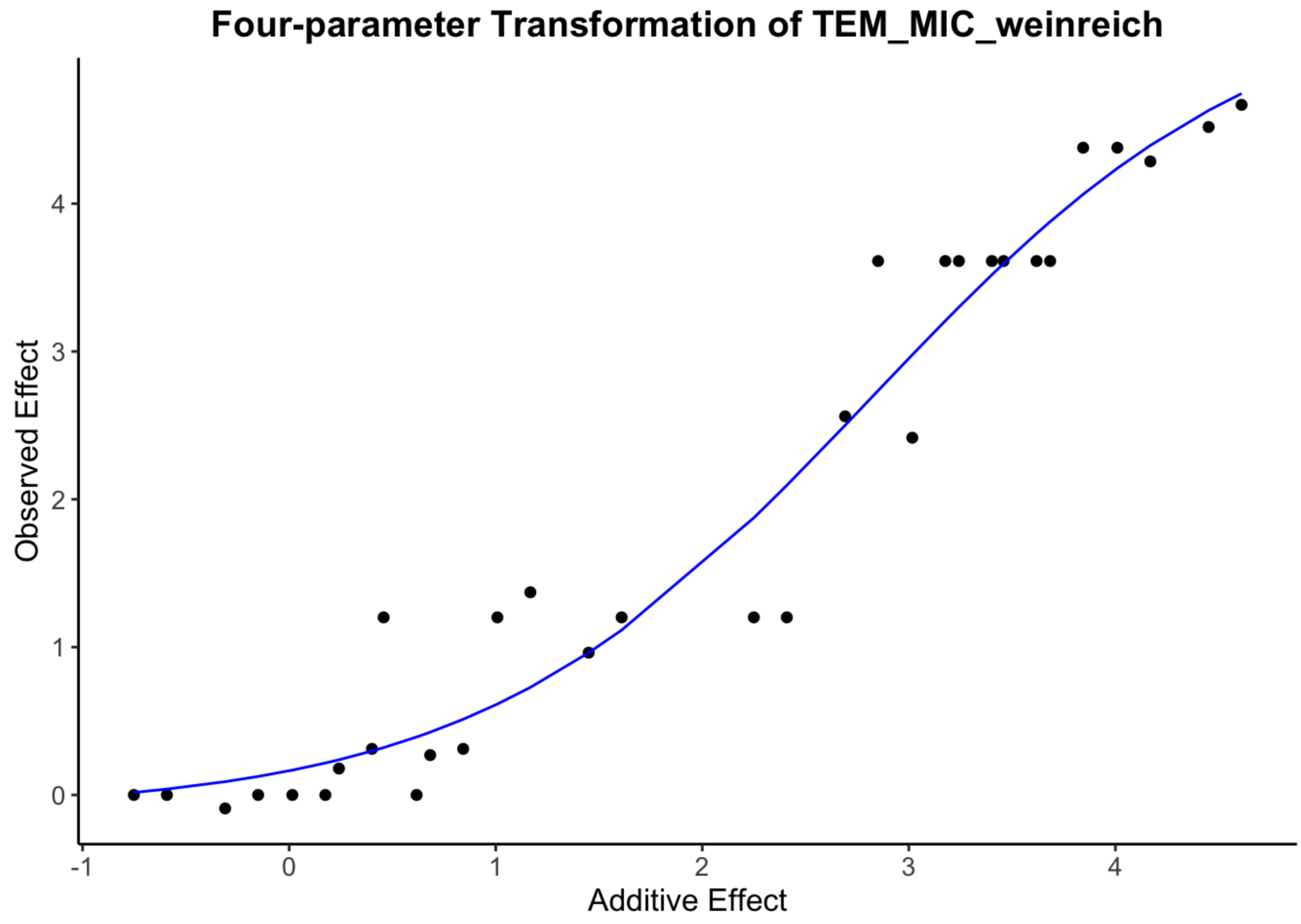

Indeed, these fits appear much better visually than the spline fits, as they avoid over fitting to the datasets. We used these transforms in the subsequent analyses. Note: we removed landscapes TEM_growth_AMP, TEM_growth_AMC, TEM_growth_CAZ, and TEM_growth_TZP, as the four-parameter model was more parsimonious than the linear model, however the fits reduced all values in the landscape to binary values that represented the upper or lower bounds of the four-parameter model.
