# Supplementary material for: Pervasive epistasis exposes intramolecular networks in adaptive enzyme evolution
Source: Nat Commun. 2023 Dec 21;14:8508. doi: 10.1038/s41467-023-44333-5 (PMC10739712; doi:10.1038/s41467-023-44333-5)
Supplement: Supplementary file 8 — Supplementary Data 5 [file 41467_2023_44333_MOESM8_ESM.pdf]

Supplementary Data 5

Representations of 41 single mutation effects (referred to as functional contribution or ΔF in this supplementary file) and 41 epistatic (ε) box plots for each fitness landscape used in the study. Red bars represent mean ΔF or ε for a position or combination, respectively. Black line represents 0 and dashed lines represent positive and negative boundaries for our 1.5-fold significance threshold. ΔF and ε are both on a $\log_{10}$ scale except for all Mira *et al.* TEM boxplots due to their growth rate (additive *vs*. multiplicative) measurements. Box plot indicates the median (black bar), the hinges represent the 25th and 75th percentiles, and the whiskers represent the minimum and maximum value. The n for each position and combination is equal to 2^{p-o} where p is the number of positions for the enzyme and o is the order (1 for functional contribution and 2+ for epistatic effects).

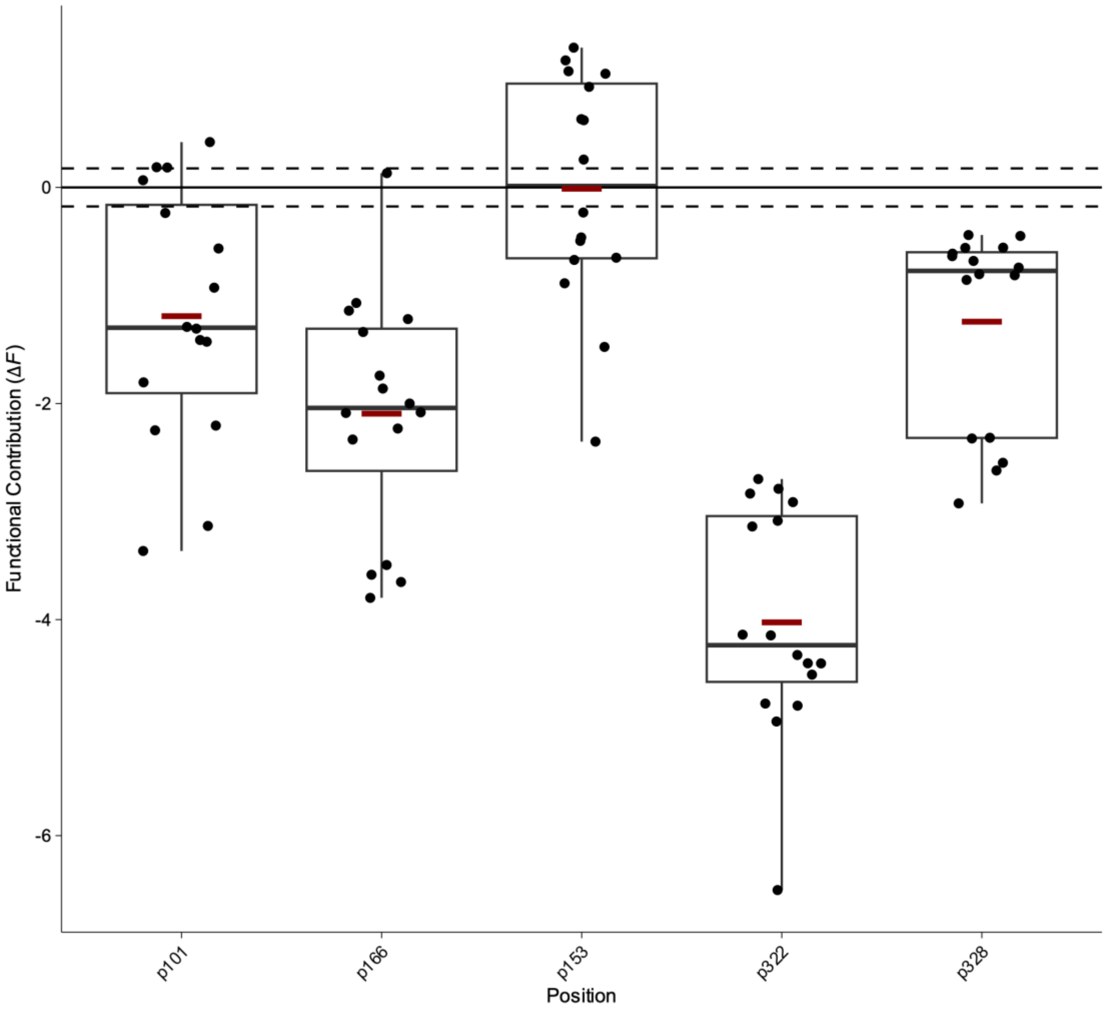

Box Plot 1 | Functional contribution of positional mutations in Alkaline Phosphatase (AP) from Sunden *et al.*

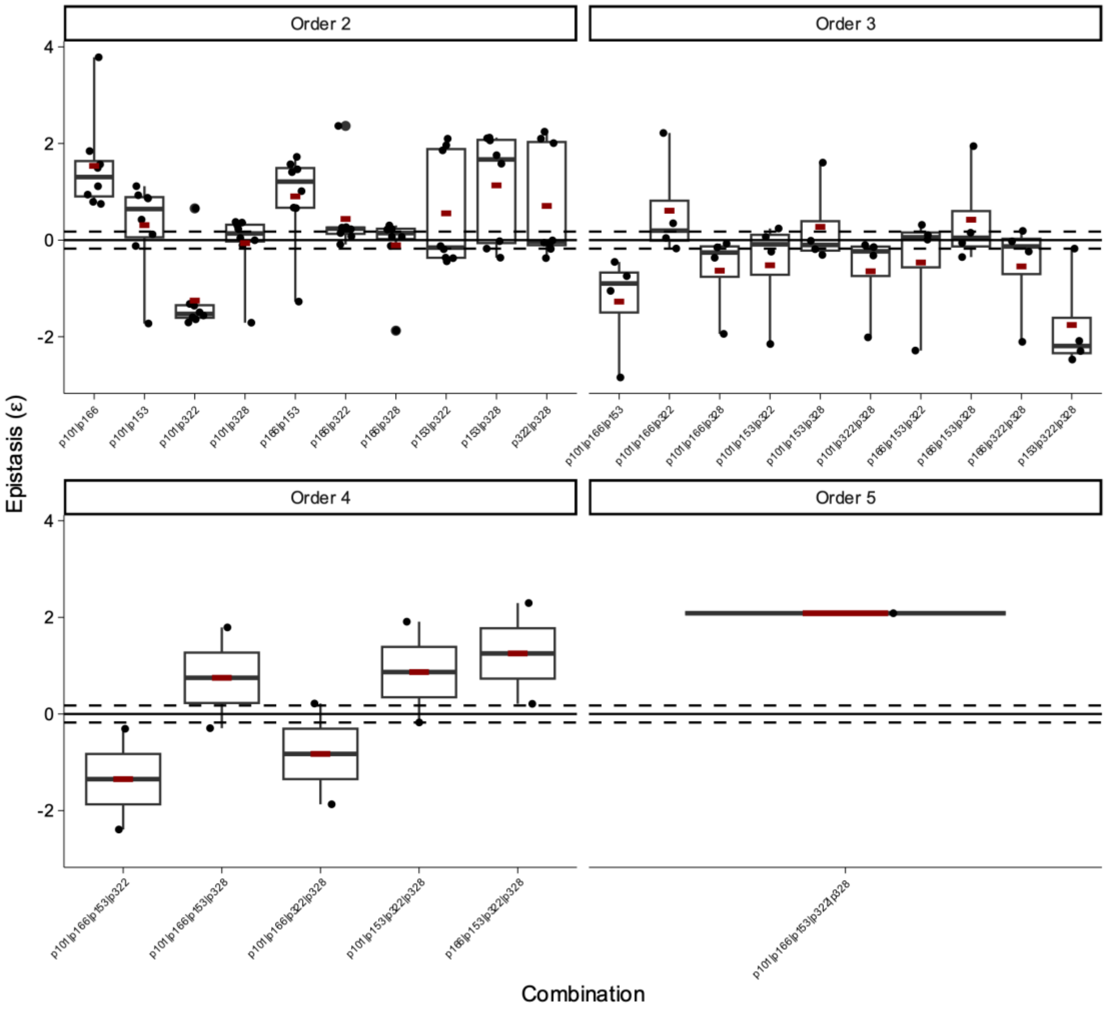

Box Plot 2 | Epistasis of combinatorial mutations in Alkaline Phosphatase (AP) from Sunden *et al.*

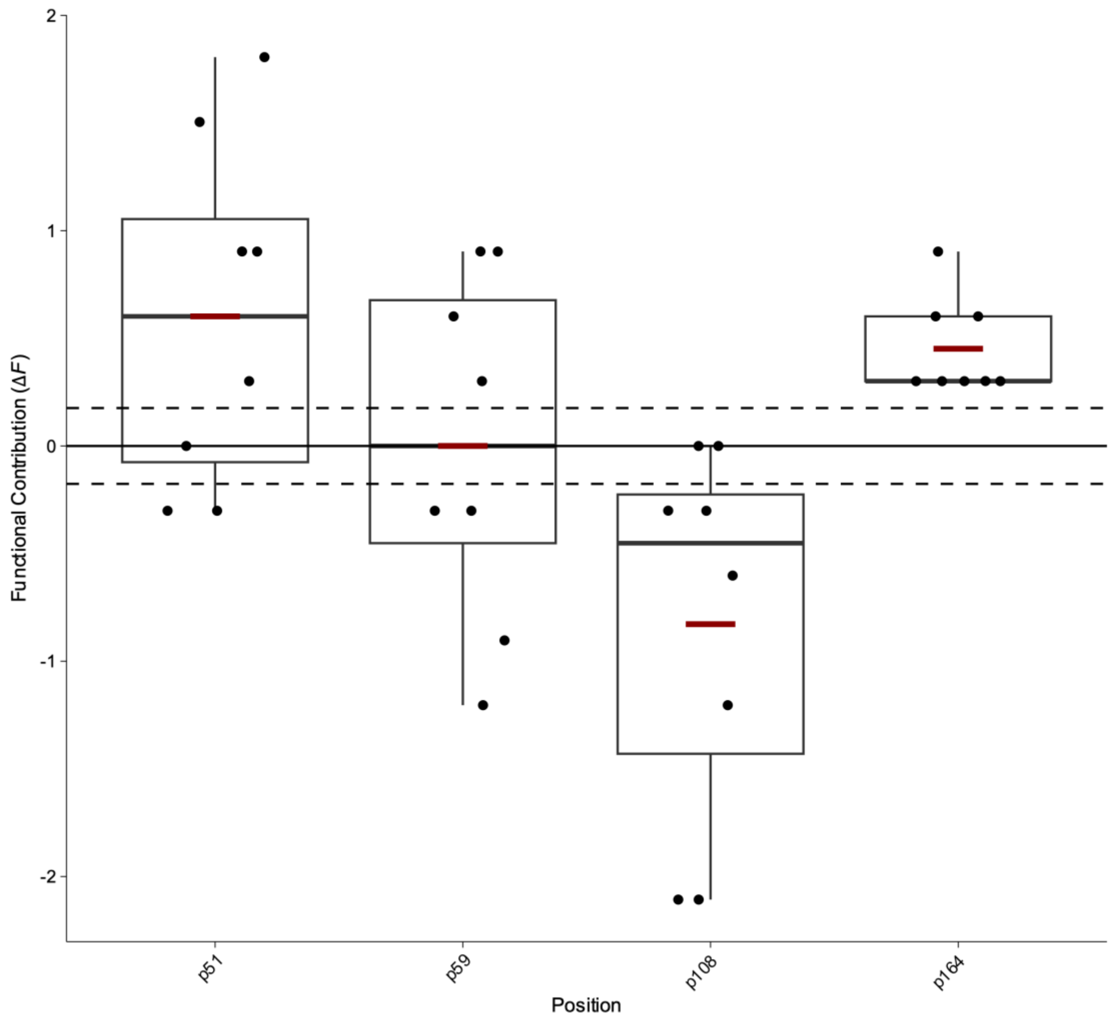

Box Plot 3 | Functional contribution of positional mutations in Dihydrofolate Reductase (DHFR) for inhibitor c57 from Lozovsky et al.

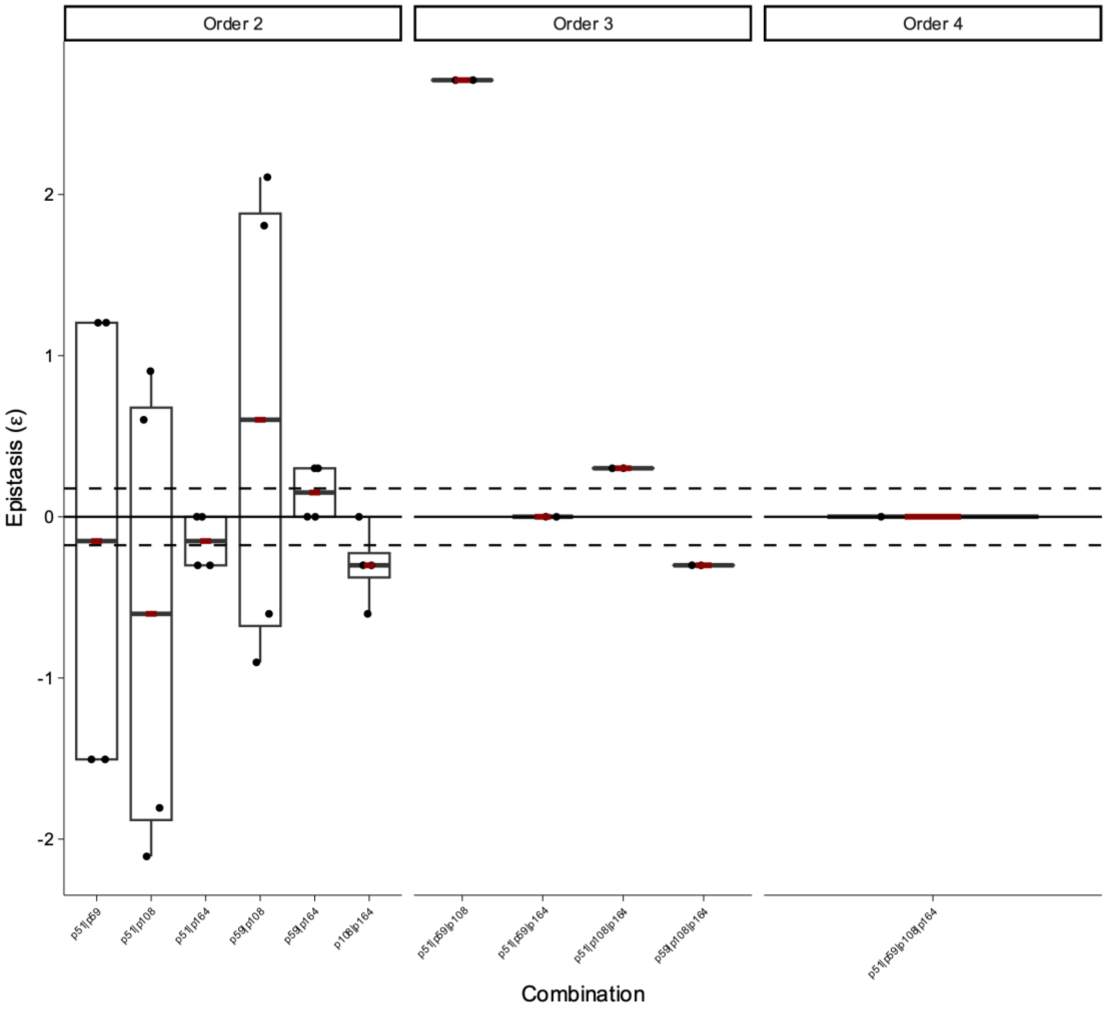

Box Plot 4 | Epistasis of combinatorial mutations in Dihydrofolate Reductase (DHFR) for inhibitor c57 from Lozovsky et al.

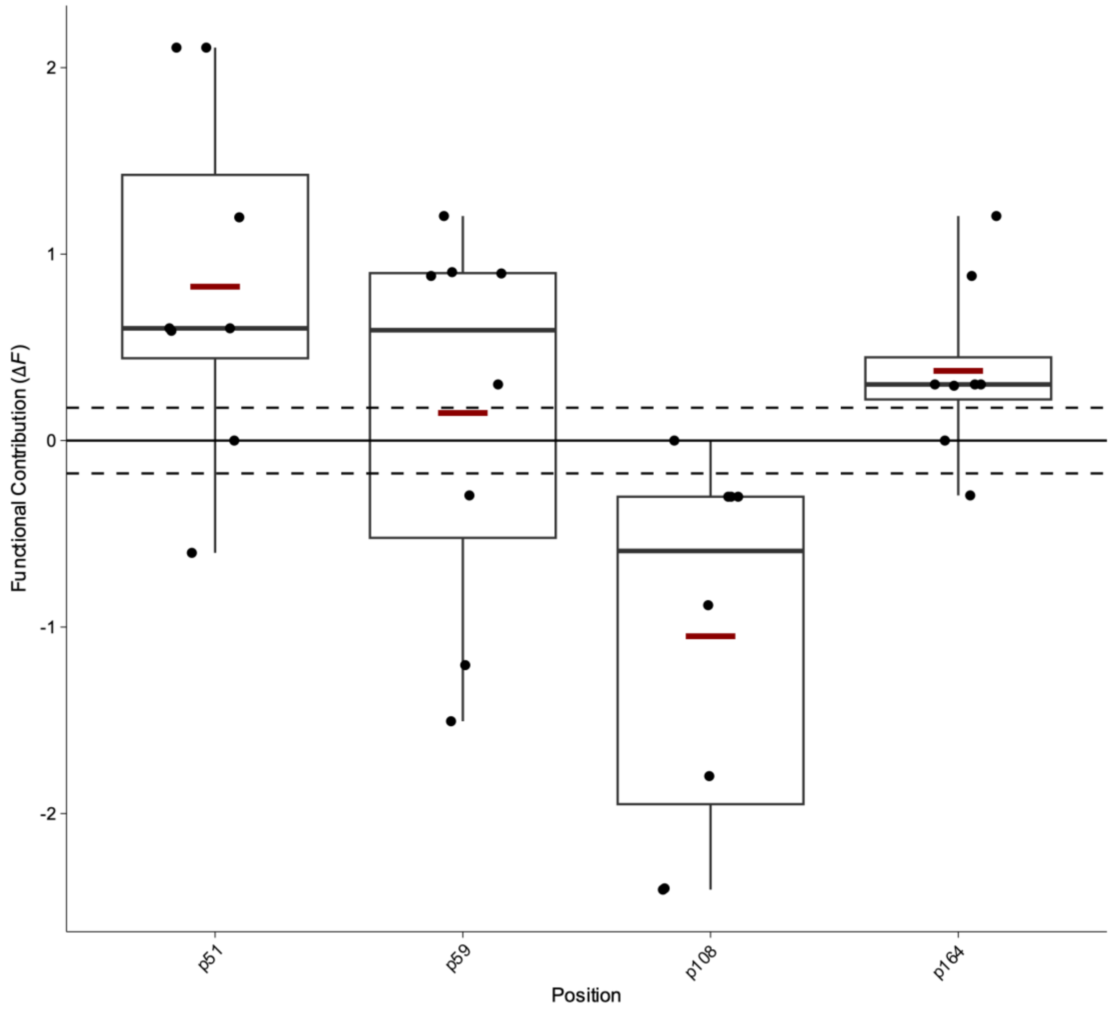

Box Plot 5 | Functional contribution of positional mutations in Dihydrofolate Reductase (DHFR) for inhibitor c58 from Lozovsky et al.

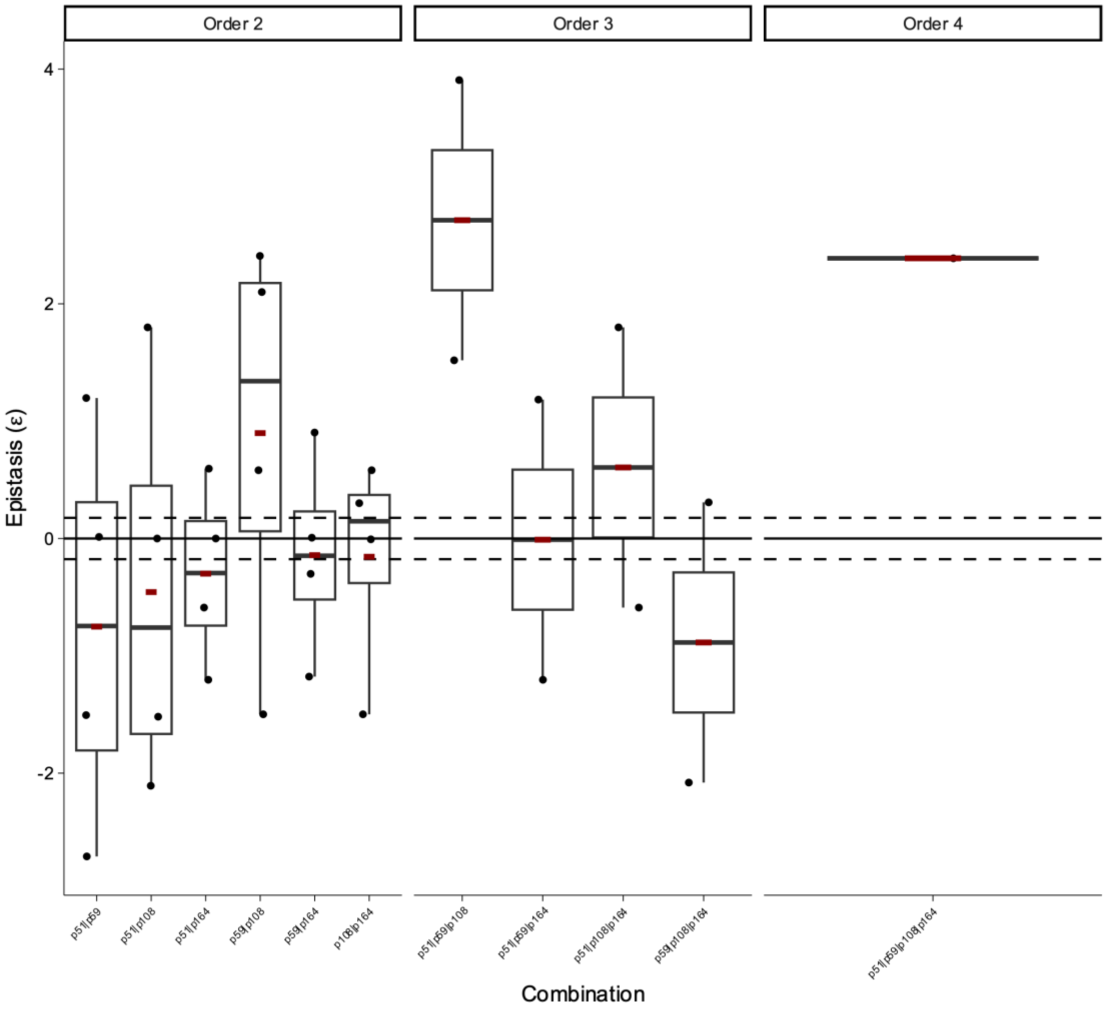

Box Plot 6 | Epistasis of combinatorial mutations in Dihydrofolate Reductase (DHFR) for inhibitor c58 from Lozovsky et al.

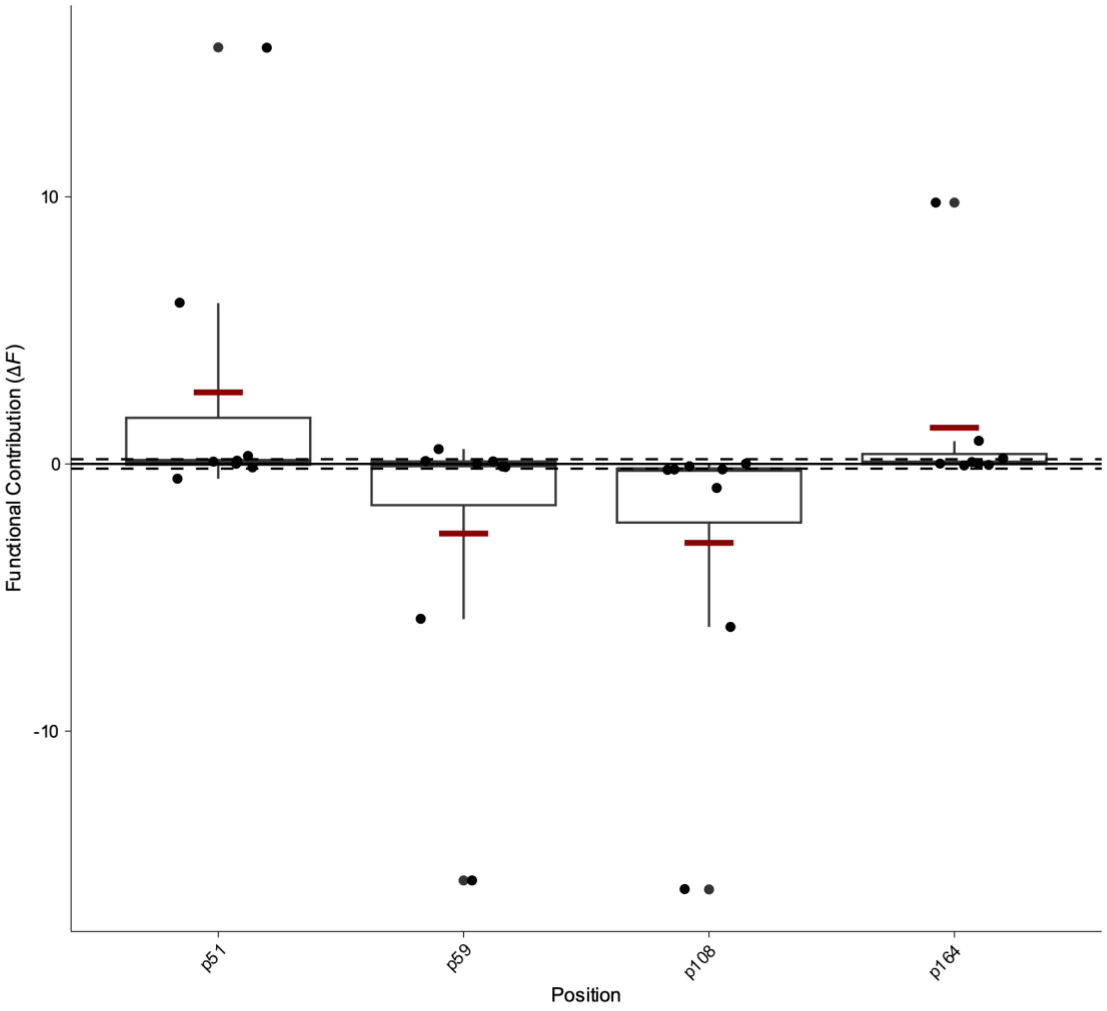

Box Plot 7 | Functional contribution of positional mutations in Dihydrofolate Reductase (DHFR) for inhibitor c59 from Lozovsky et al.

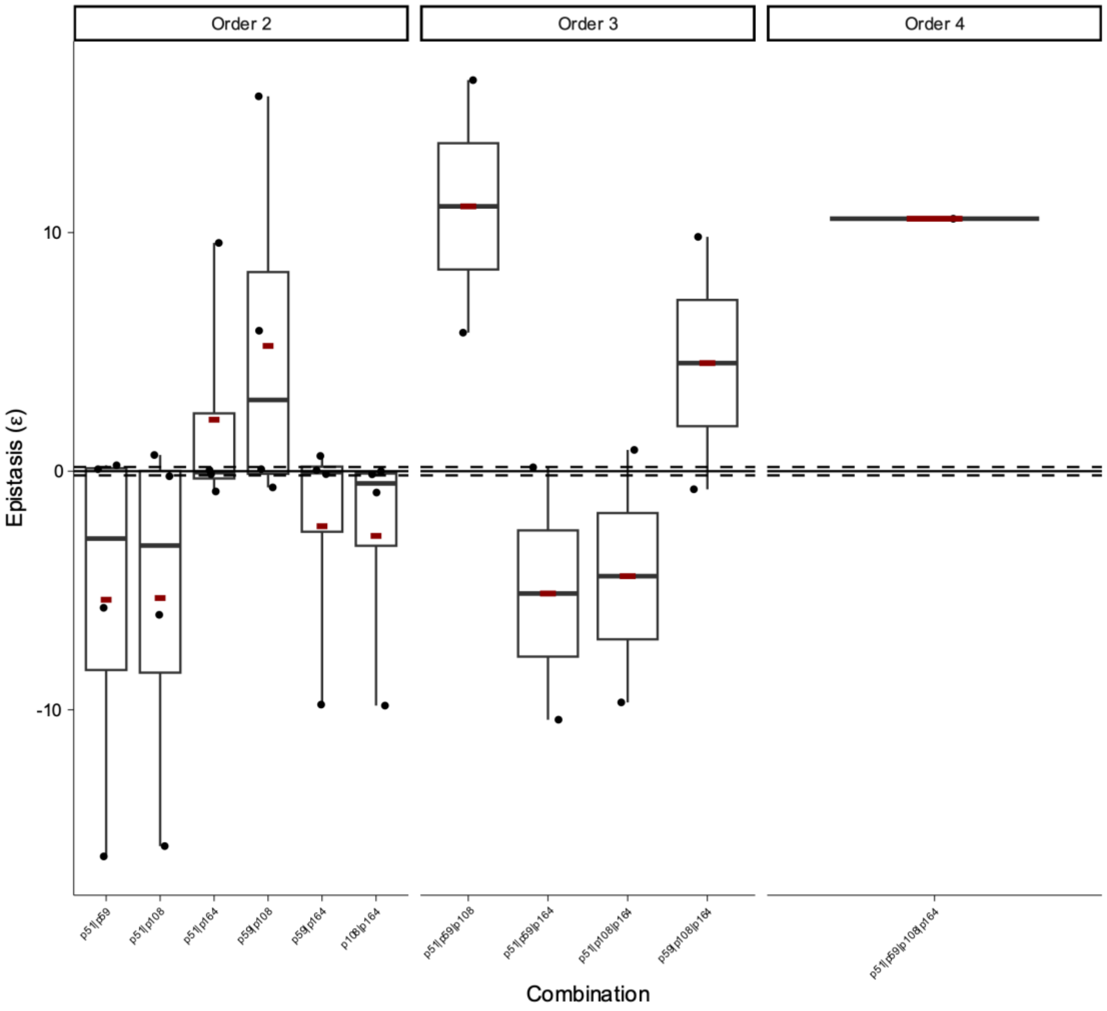

Box Plot 8 | Epistasis of combinatorial mutations in Dihydrofolate Reductase (DHFR) for inhibitor c59 from Lozovsky et al.

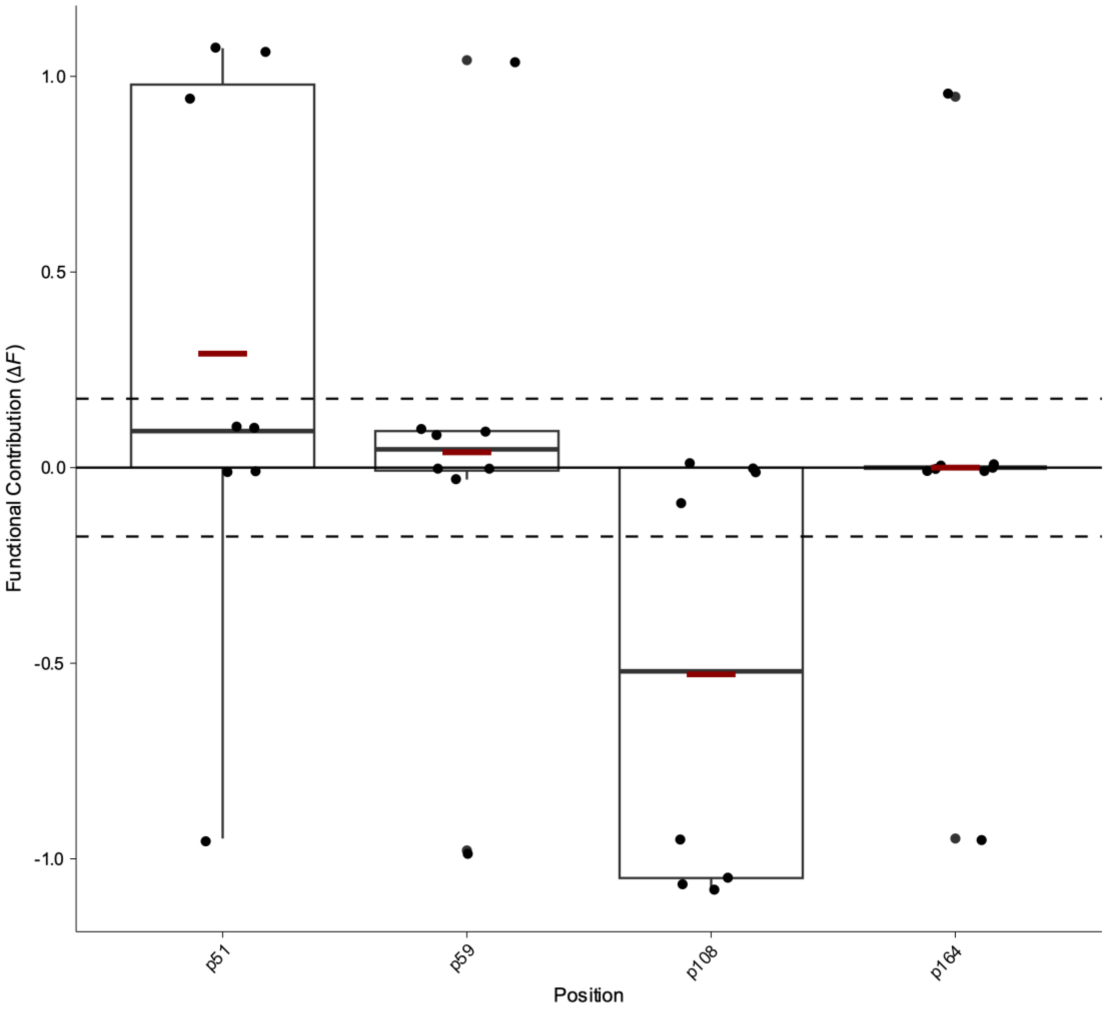

Box Plot 9 | Functional contribution of positional mutations in Dihydrofolate Reductase (DHFR) for inhibitor c60 from Lozovsky et al.

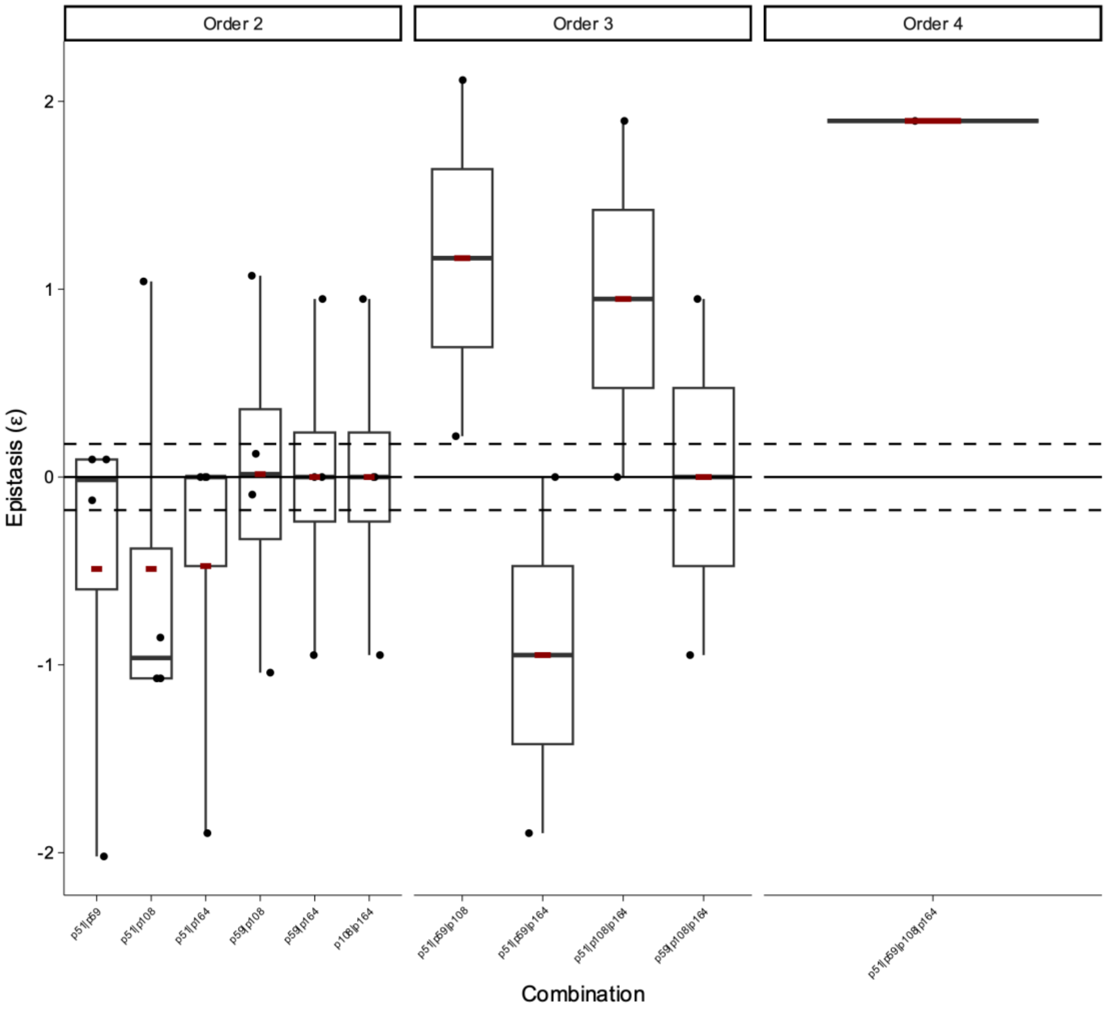

Box Plot 10 | Epistasis of combinatorial mutations in Dihydrofolate Reductase (DHFR) for inhibitor c60 from Lozovsky et al.

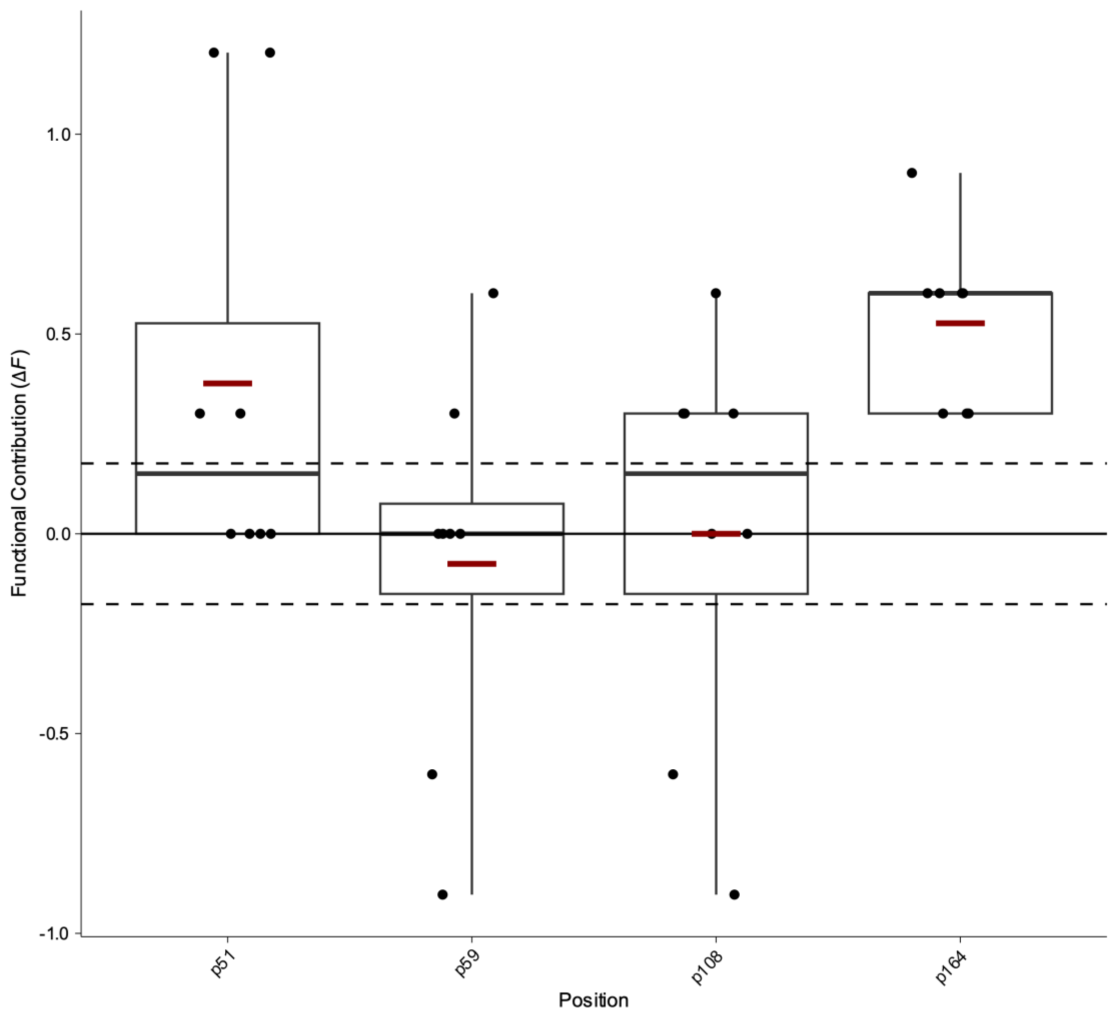

Box Plot 11 | Functional contribution of positional mutations in Dihydrofolate Reductase (DHFR) for inhibitor c61 from Lozovsky et al.

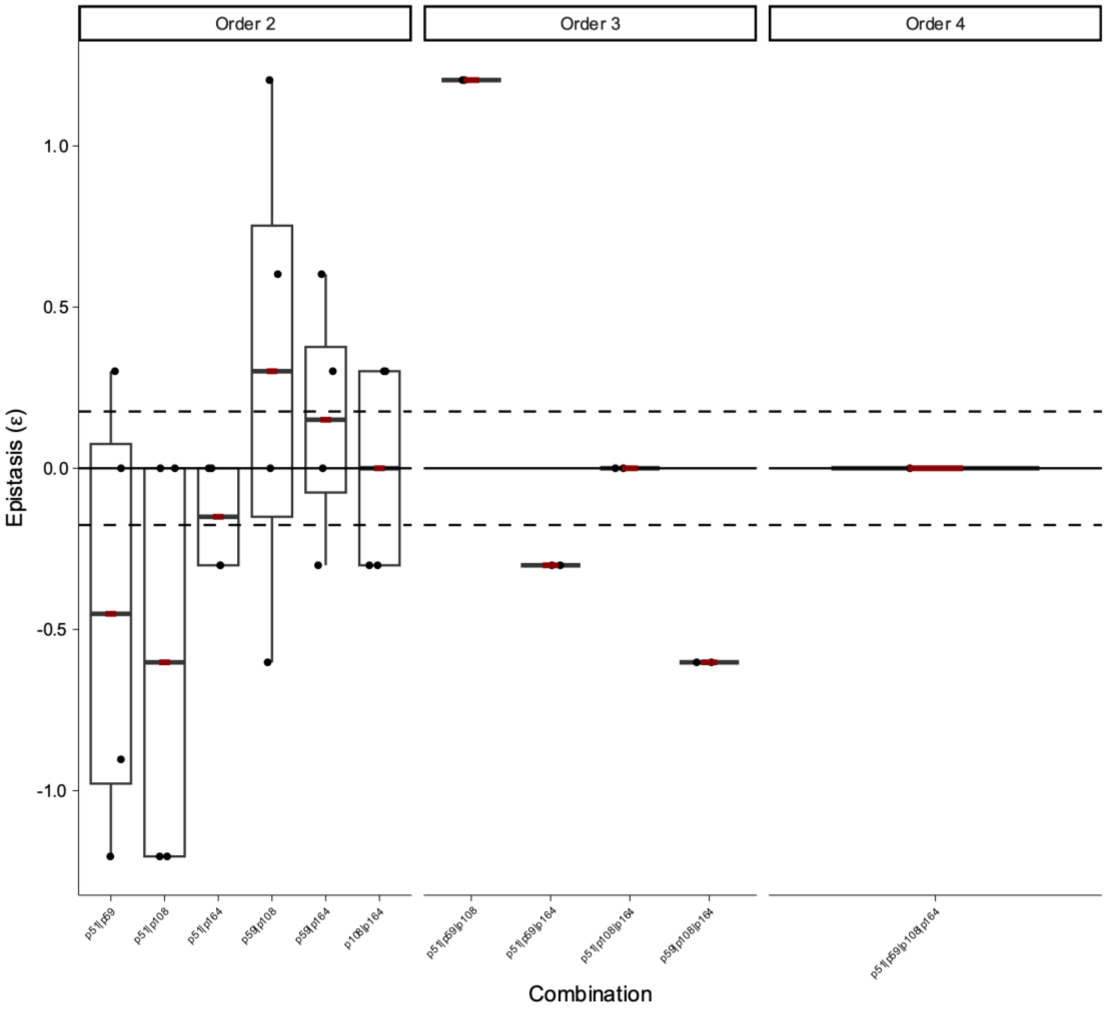

Box Plot 12 | Epistasis of combinatorial mutations in Dihydrofolate Reductase (DHFR) for inhibitor c61 from Lozovsky et al.

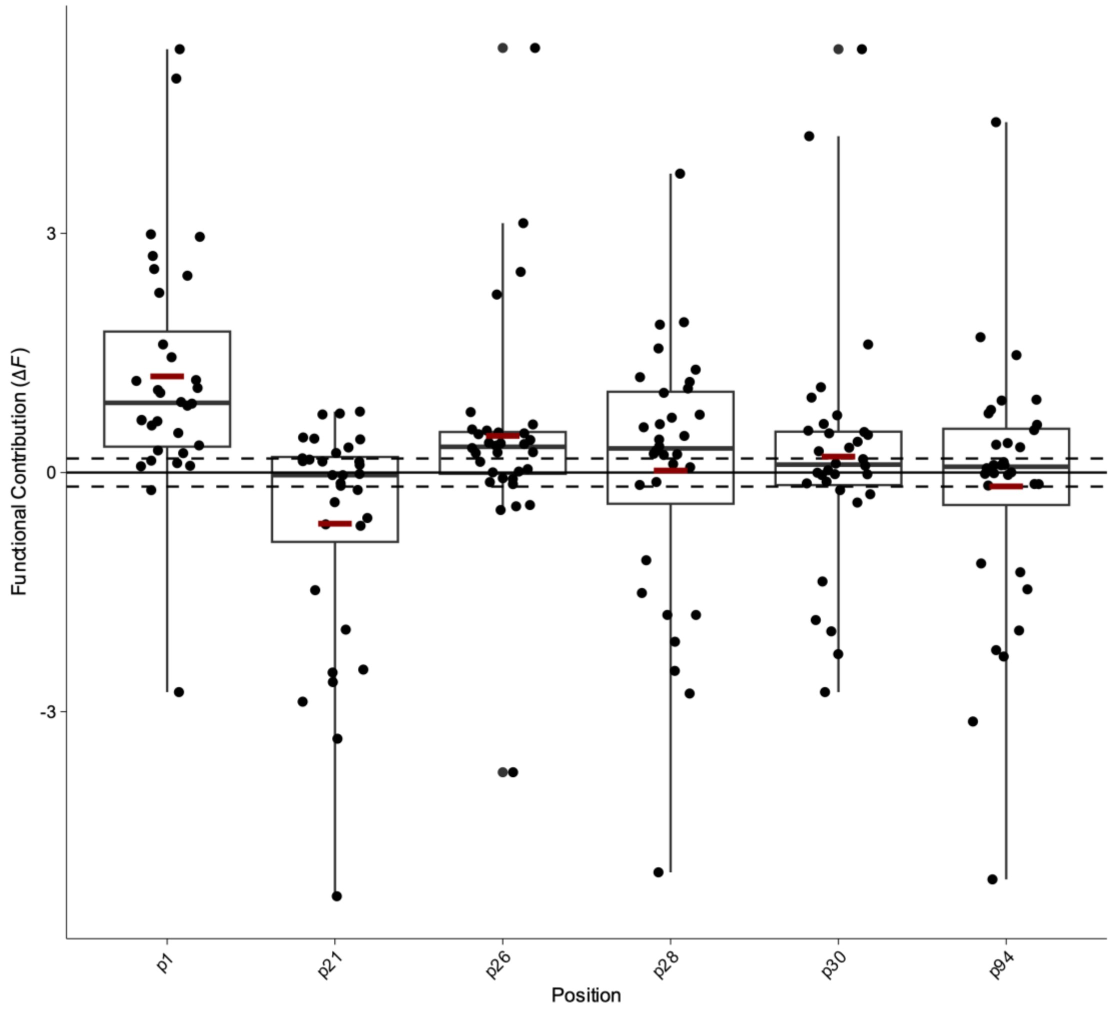

Box Plot 13 | Functional contribution of positional mutations in Dihydrofolate Reductase (DHFR) from Palmer et al.

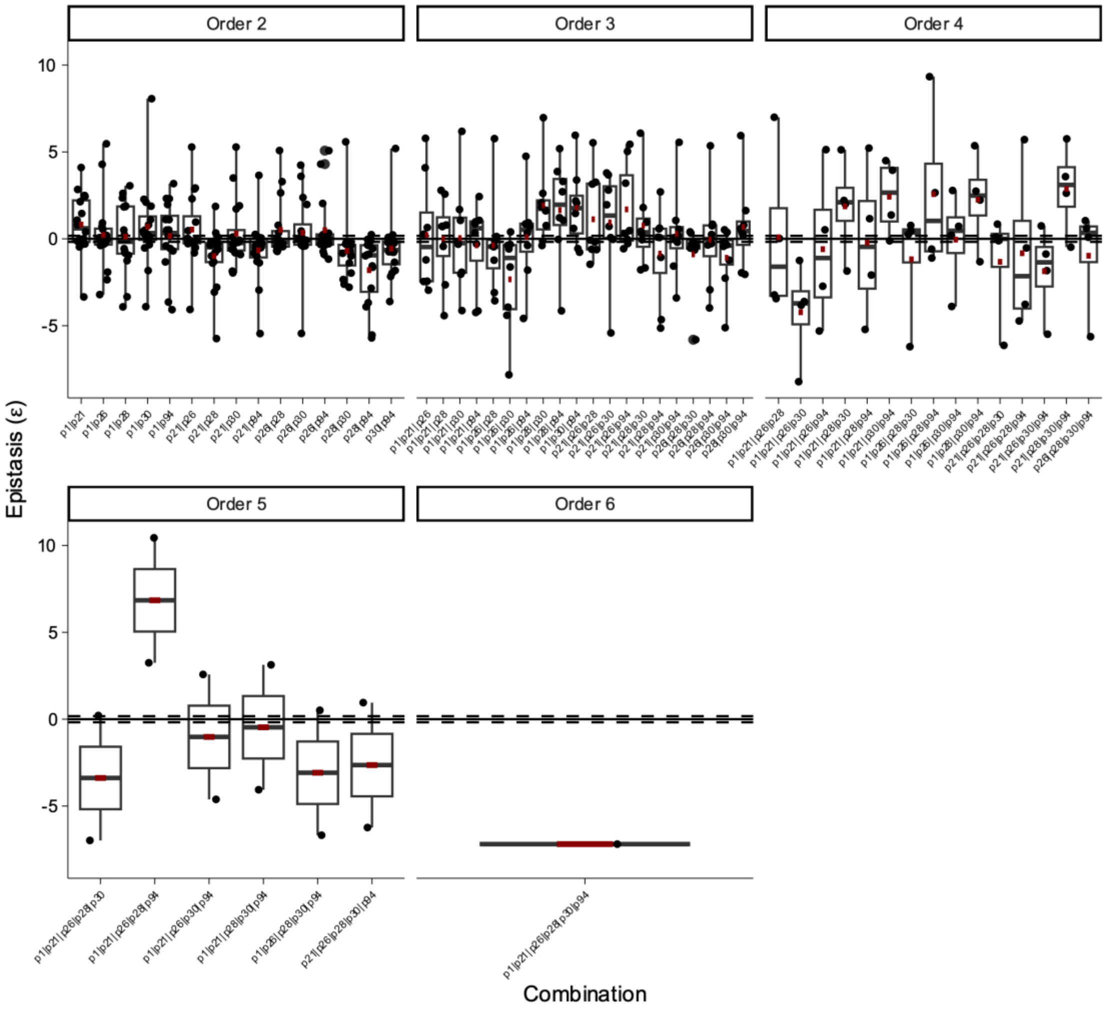

Box Plot 14 | Epistasis of combinatorial mutations in Dihydrofolate Reductase (DHFR) from Palmer et al.

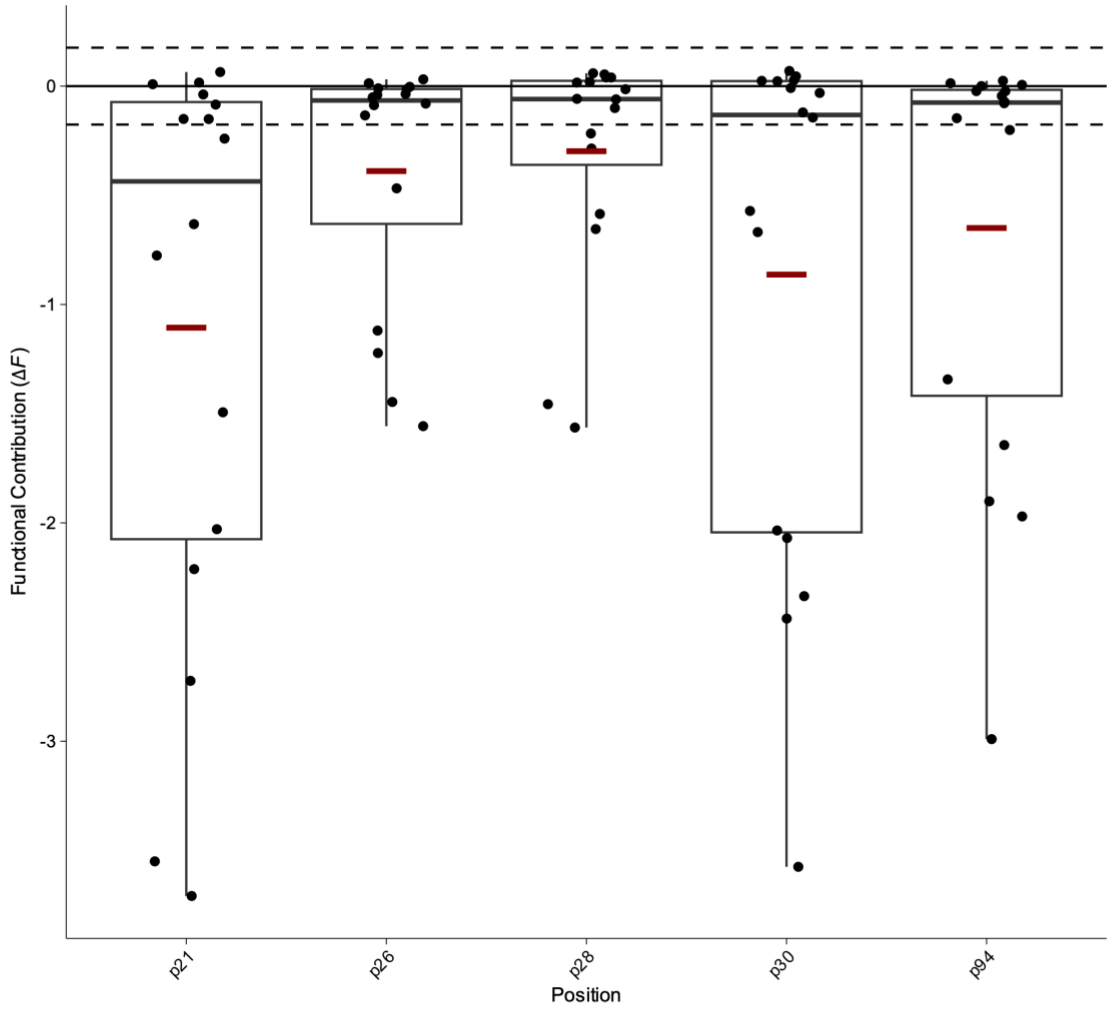

Box Plot 15 | Functional contribution of positional mutations of Dihydrofolate Reductase (DHFR) of k_{cat} in the glycine trajectory from Tamer *et al.*

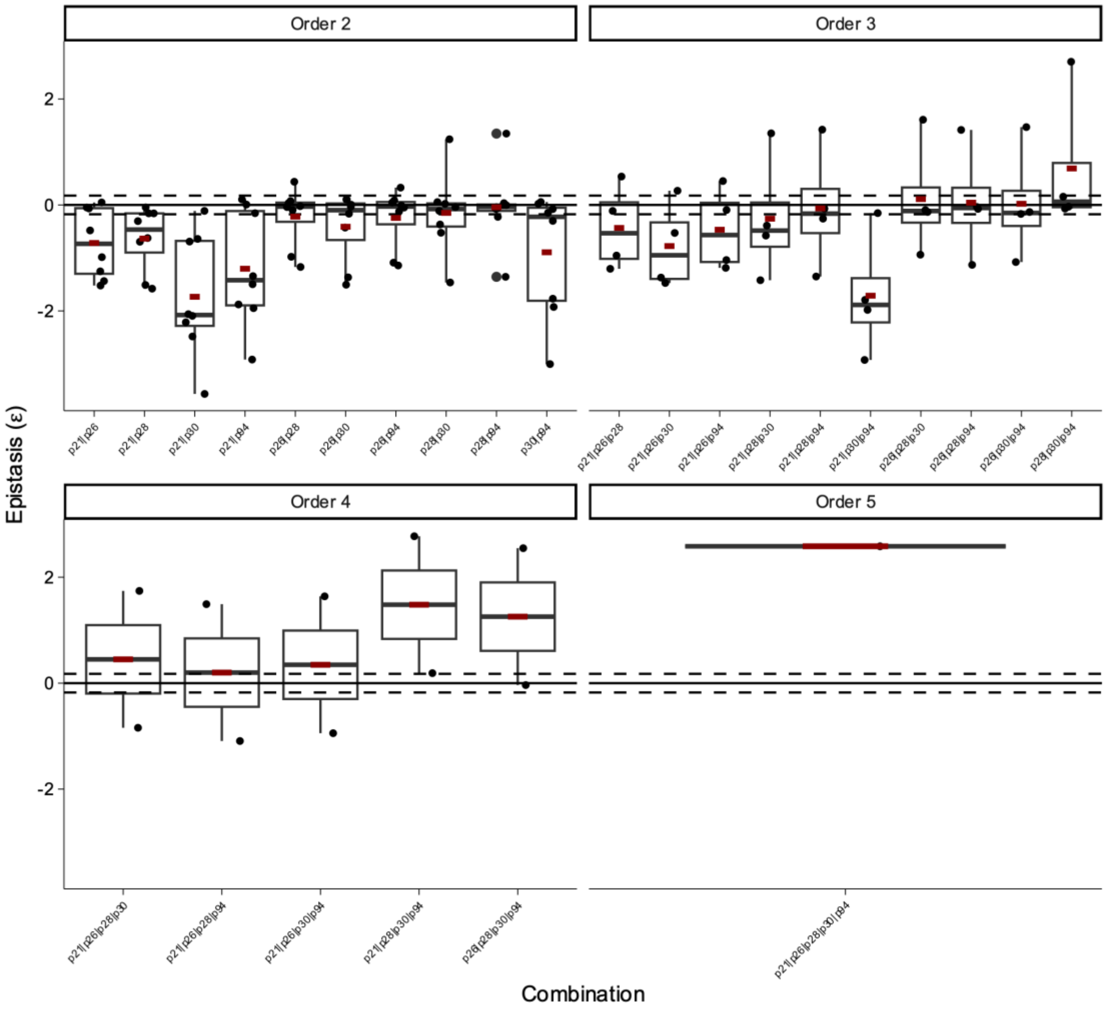

Box Plot 16 | Epistasis of combinatorial mutations of Dihydrofolate Reductase (DHFR) of k_{cat} in the glycine trajectory from Tamer *et al.*

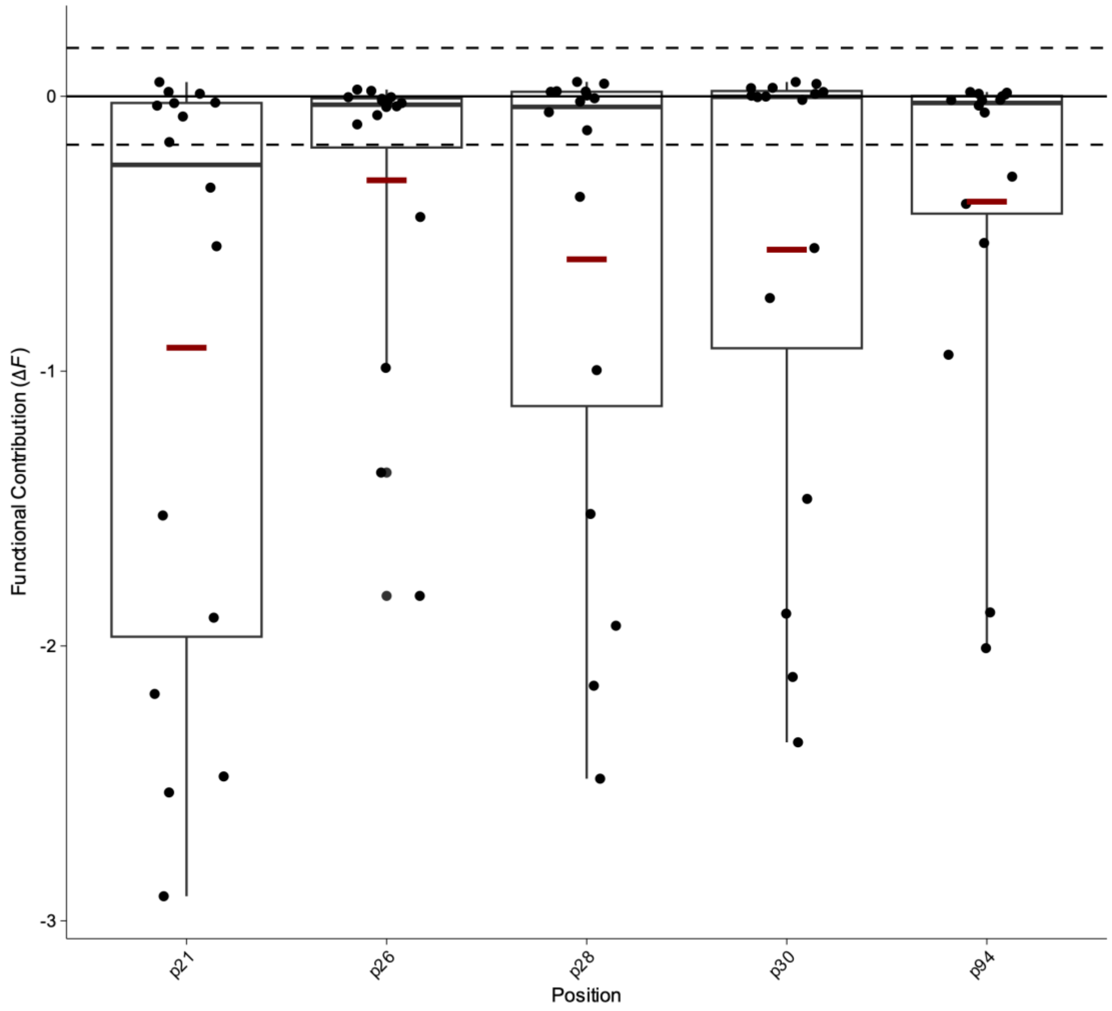

Box Plot 17 | Functional contribution of positional mutations of Dihydrofolate Reductase (DHFR) of k_{cat} in the arginine trajectory from Tamer *et al.*

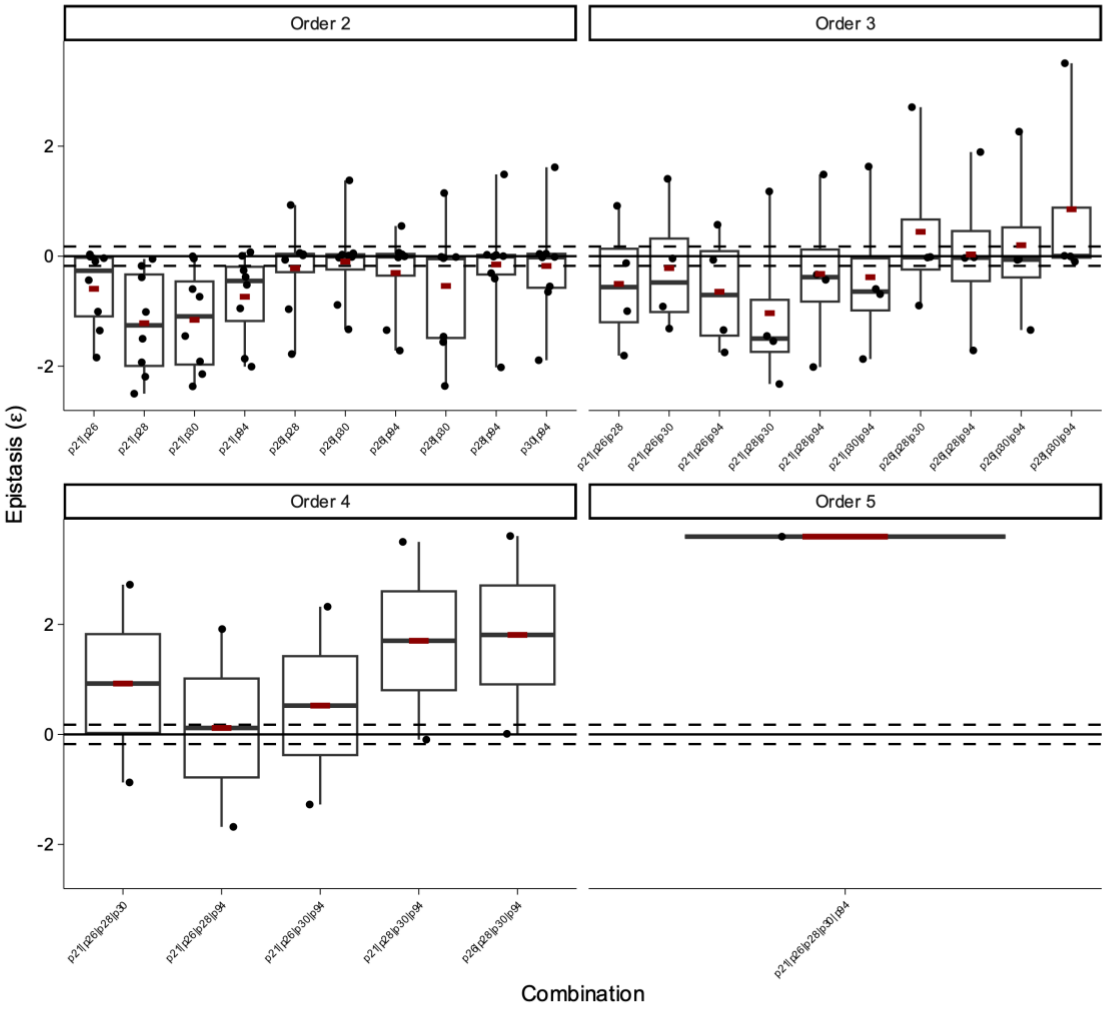

Box Plot 18 | Epistasis of combinatorial mutations of Dihydrofolate Reductase (DHFR) of k_{cat} in the arginine trajectory from Tamer *et al.*

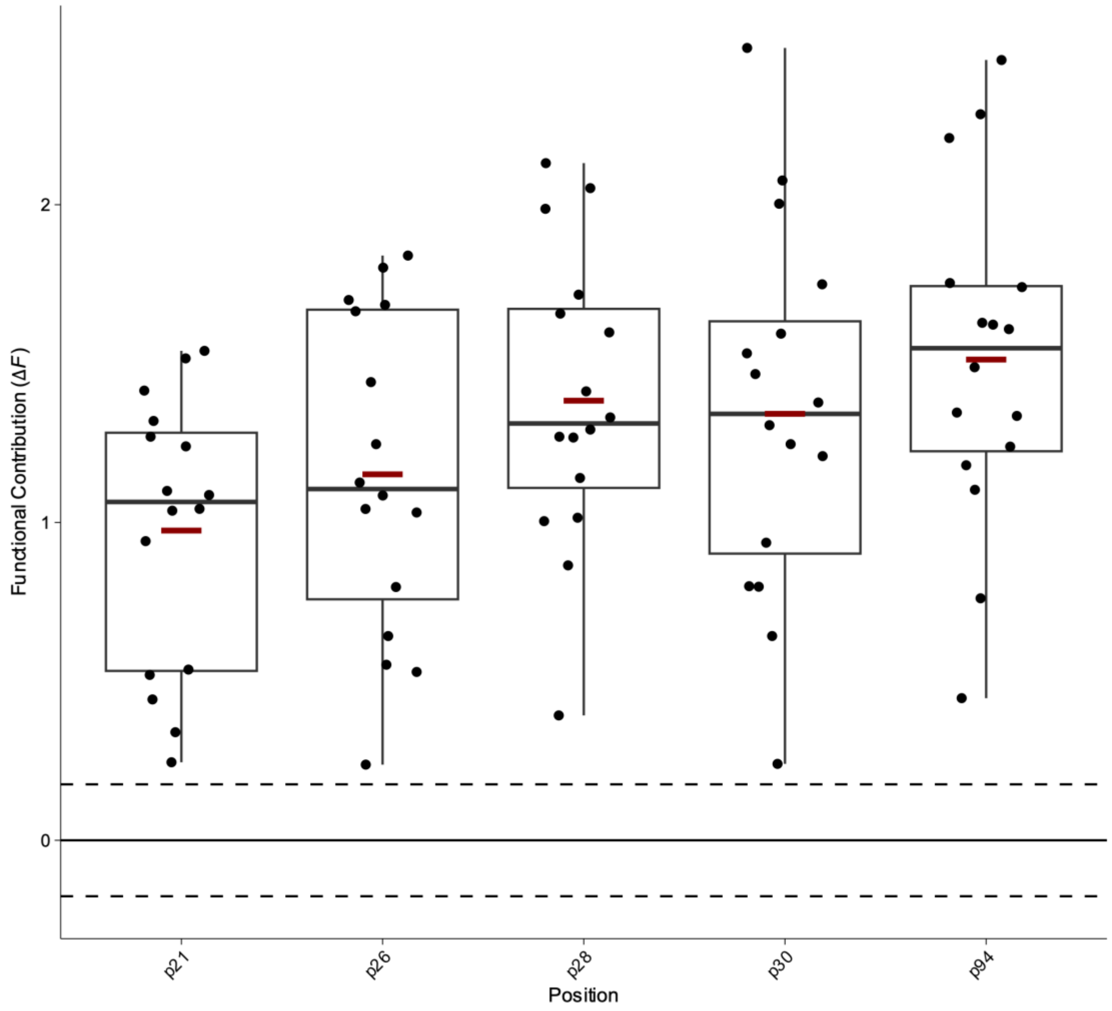

Box Plot 19 | Functional contribution of positional mutations of Dihydrofolate Reductase (DHFR) of K_I in the glycine trajectory from Tamer *et al.*

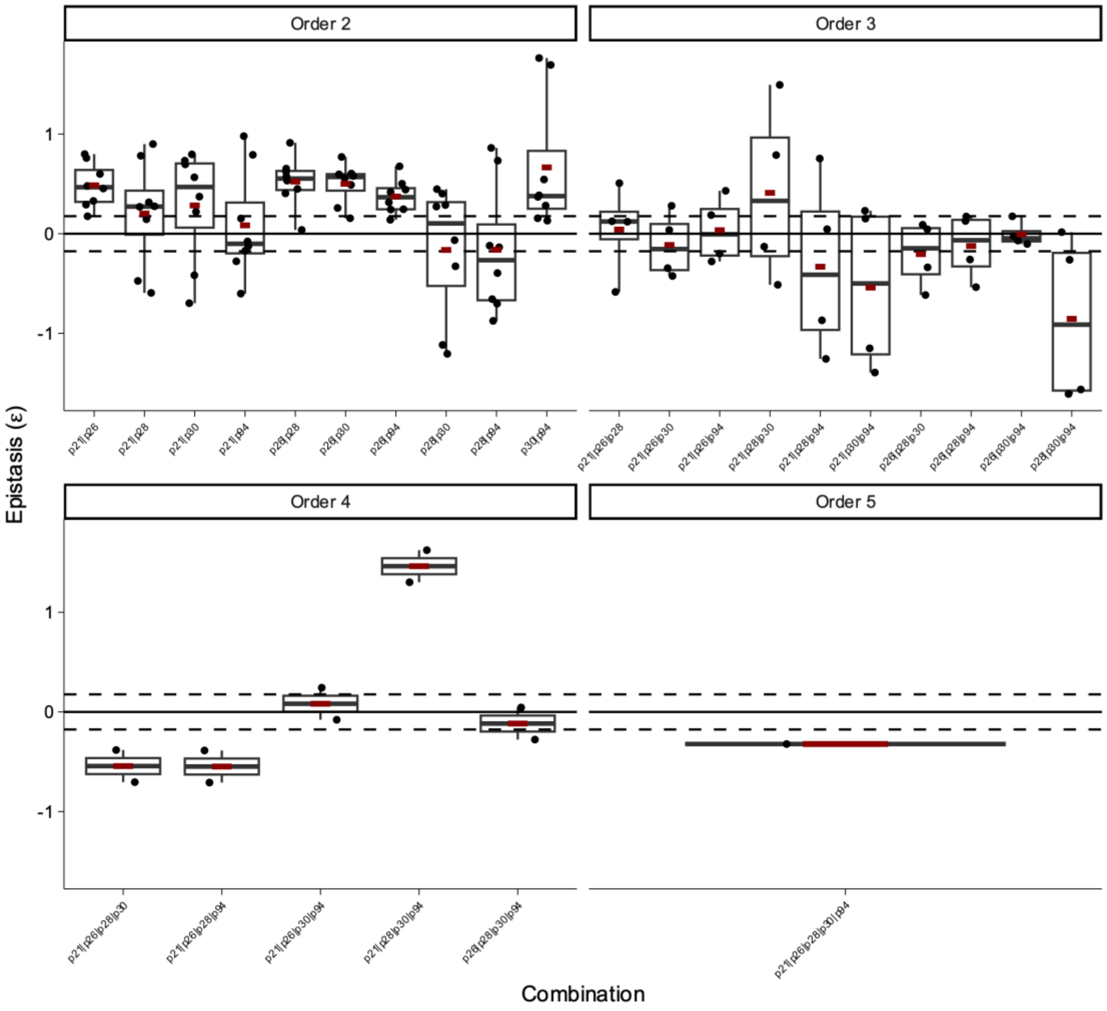

Box Plot 20 | Epistasis of combinatorial mutations of Dihydrofolate Reductase (DHFR) of K_I in the glycine trajectory from Tamer *et al.*

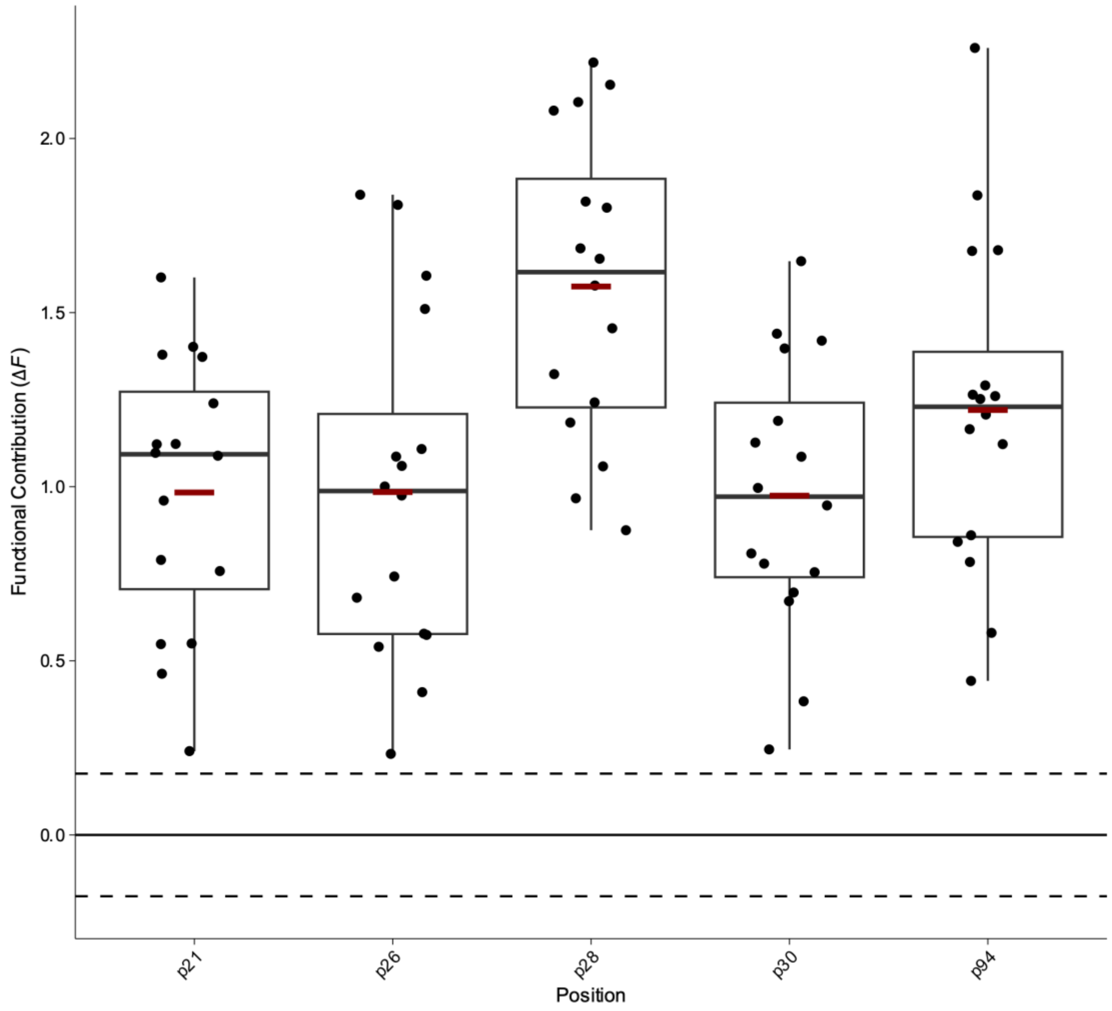

Box Plot 21 | Functional contribution of positional mutations of Dihydrofolate Reductase (DHFR) of K_I in the arginine trajectory from Tamer *et al.*

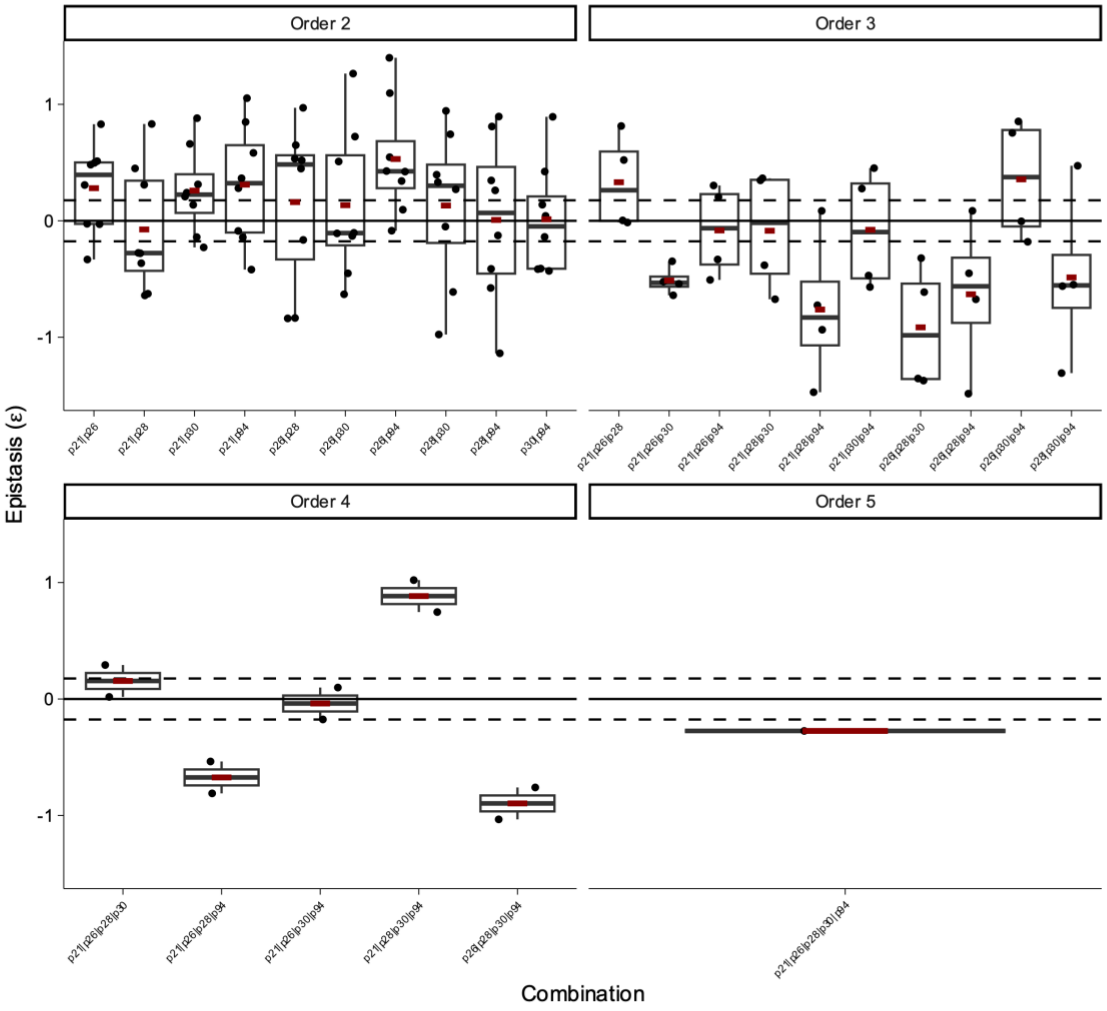

Box Plot 22 | Epistasis of combinatorial mutations of Dihydrofolate Reductase (DHFR) of K_I in the arginine trajectory from Tamer *et al.*

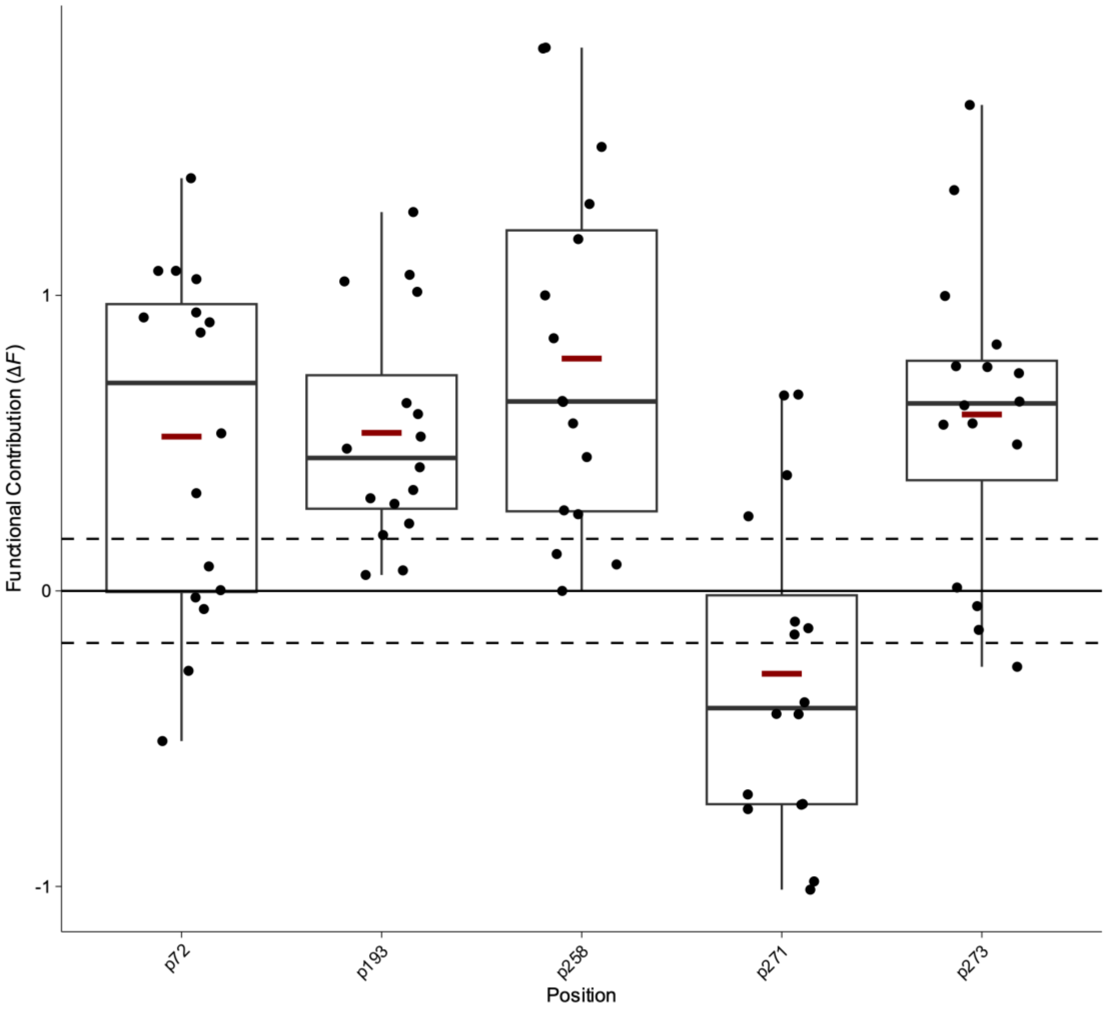

Box Plot 23 | Functional contribution of positional mutations in Methyl Parathion Hydrolase (MPH) for methyl parathion in calcium metal conditions from Anderson *et al.*

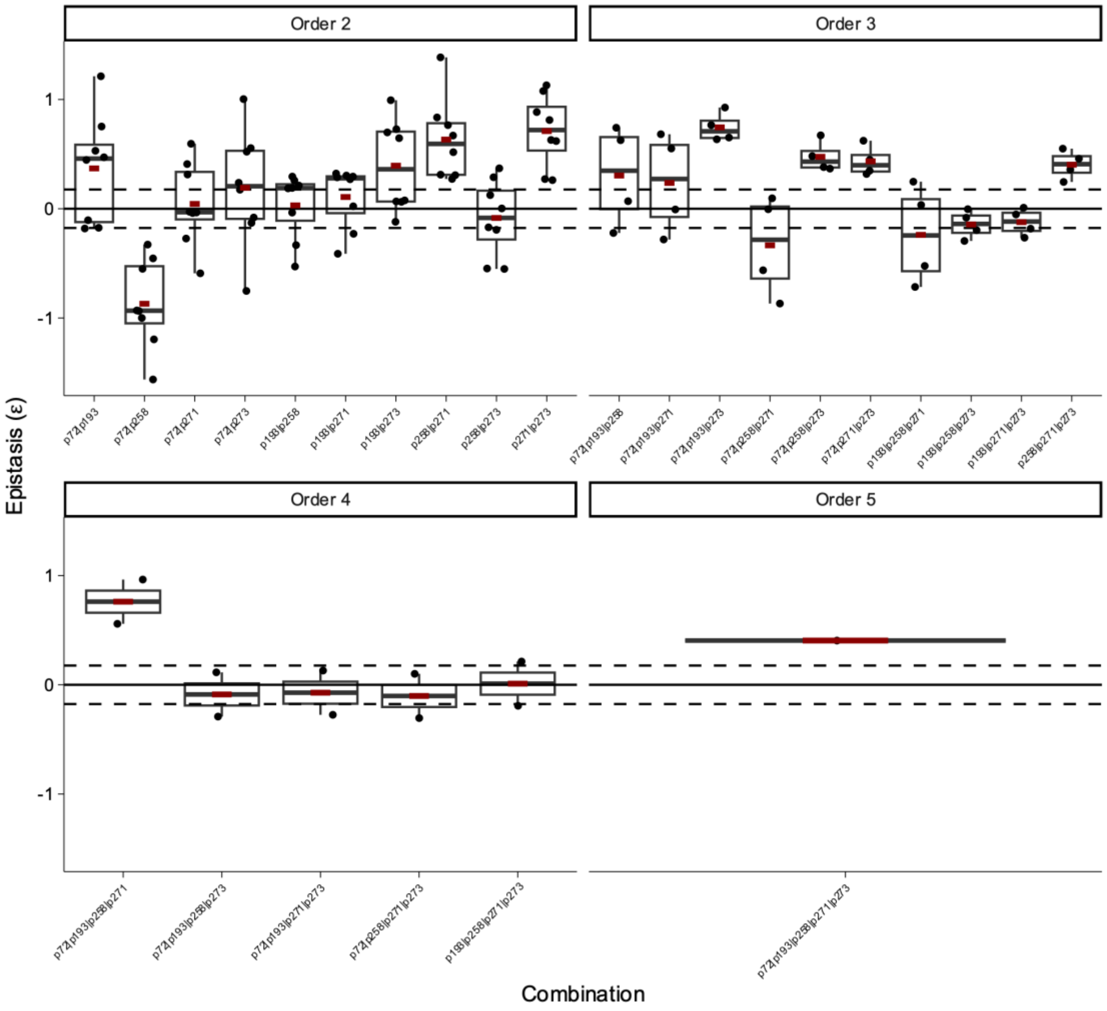

Box Plot 24 | Epistasis of combinatorial mutations in Methyl Parathion Hydrolase (MPH) for methyl parathion in calcium metal conditions from Anderson *et al.*

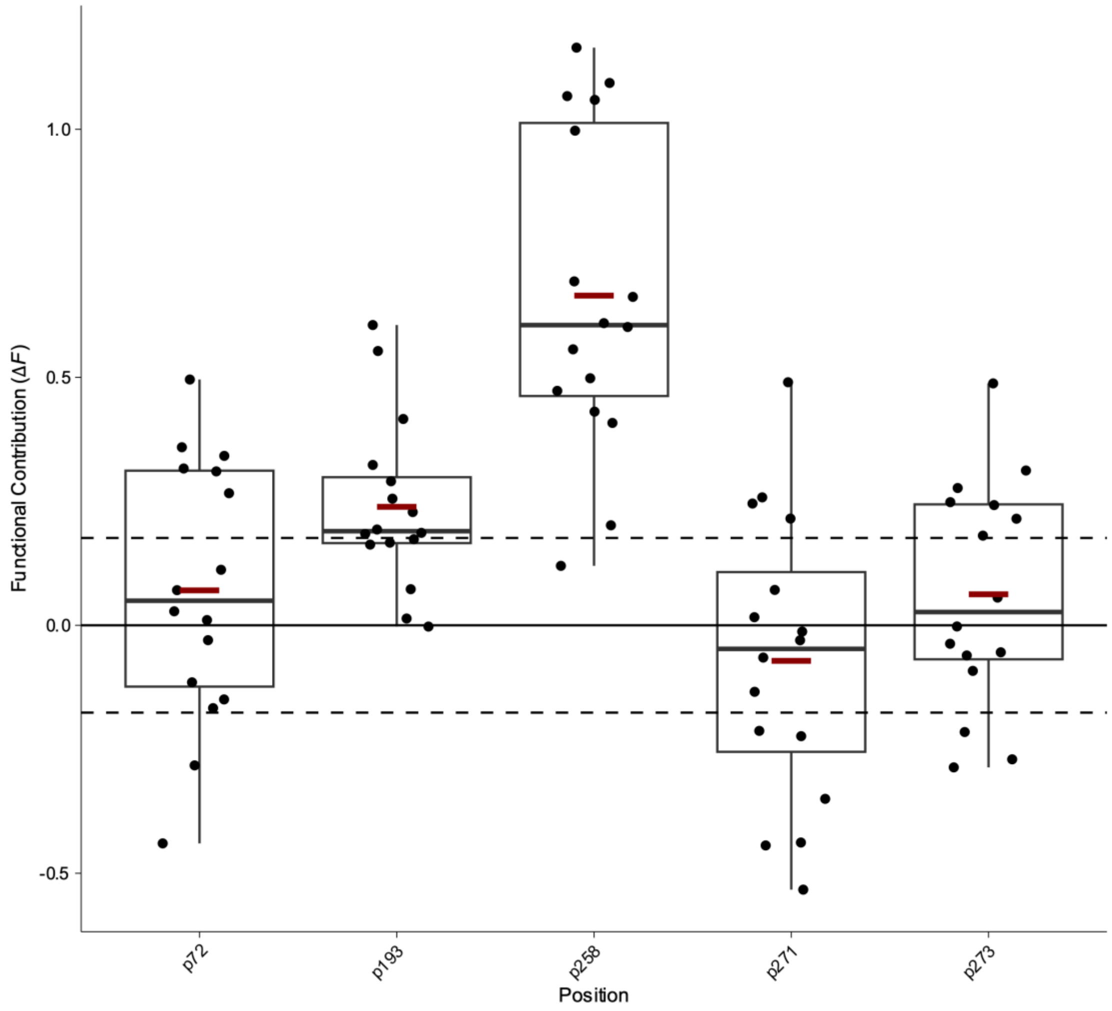

Box Plot 25 | Functional contribution of positional mutations in Methyl Parathion Hydrolase (MPH) for methyl parathion in cadmium metal conditions from Anderson *et al.*

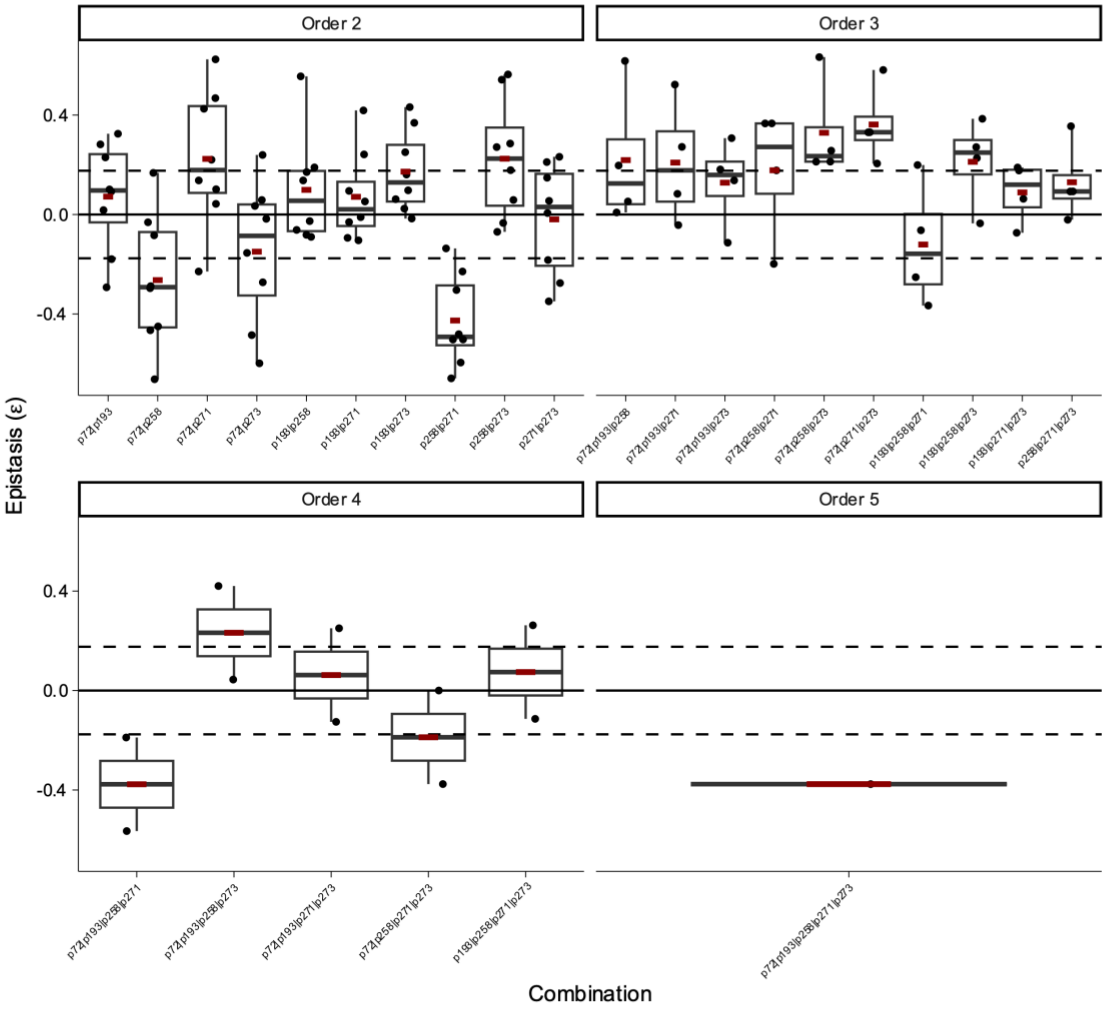

Box Plot 26 | Epistasis of combinatorial mutations in Methyl Parathion Hydrolase (MPH) for methyl parathion in cadmium metal conditions from Anderson *et al.*

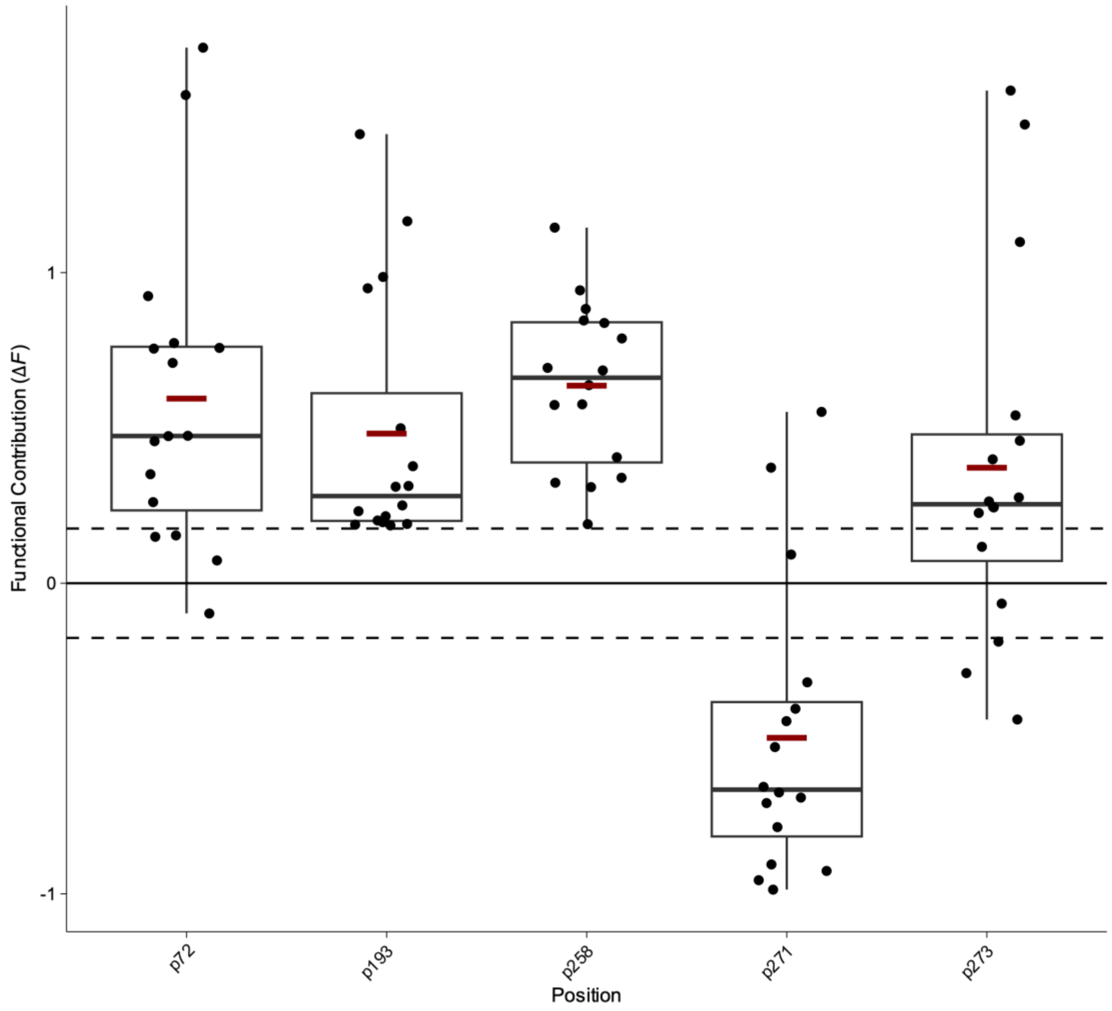

Box Plot 27 | Functional contribution of positional mutations in Methyl Parathion Hydrolase (MPH) for methyl parathion in cobalt metal conditions from Anderson *et al.*

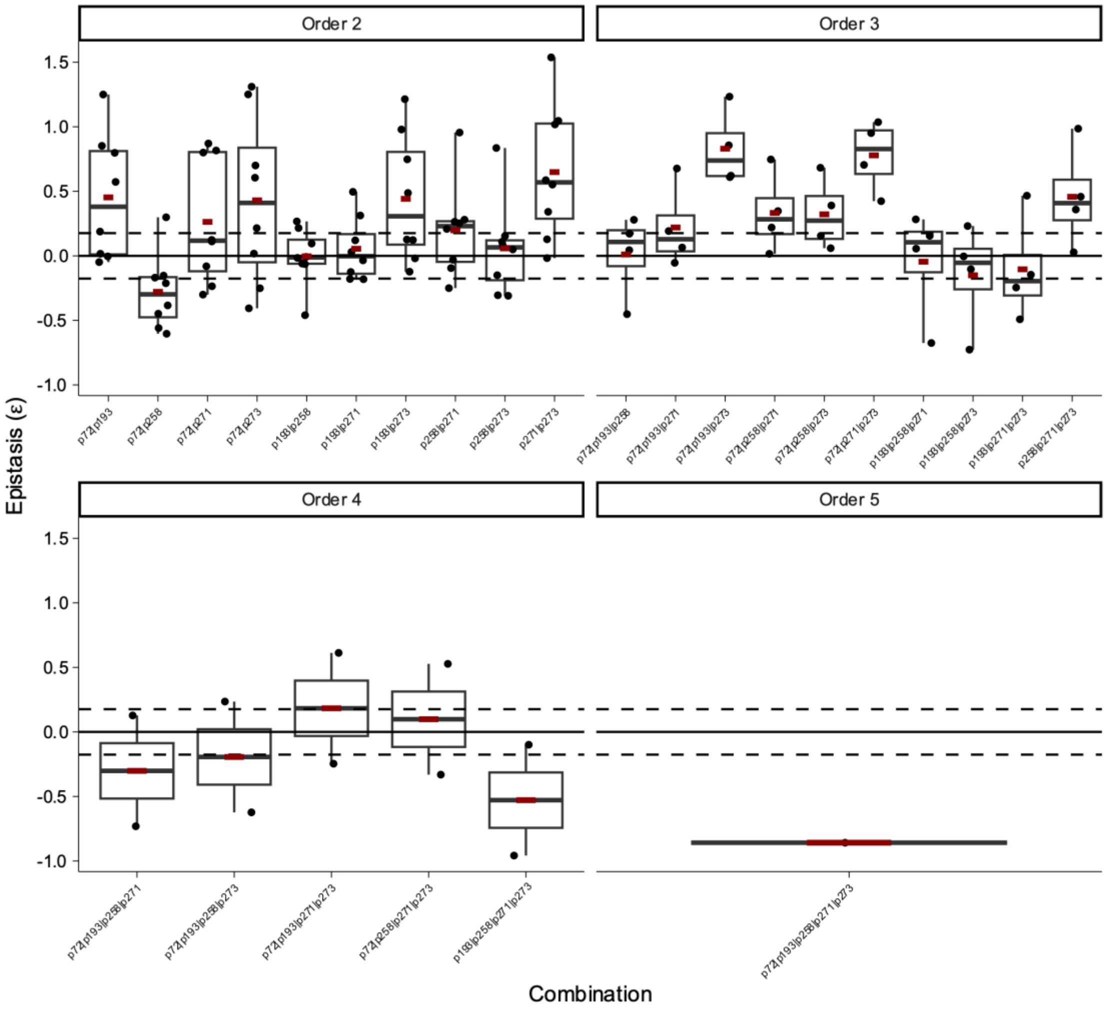

Box Plot 28 | Epistasis of combinatorial mutations in Methyl Parathion Hydrolase (MPH) for methyl parathion in cobalt metal conditions from Anderson *et al.*

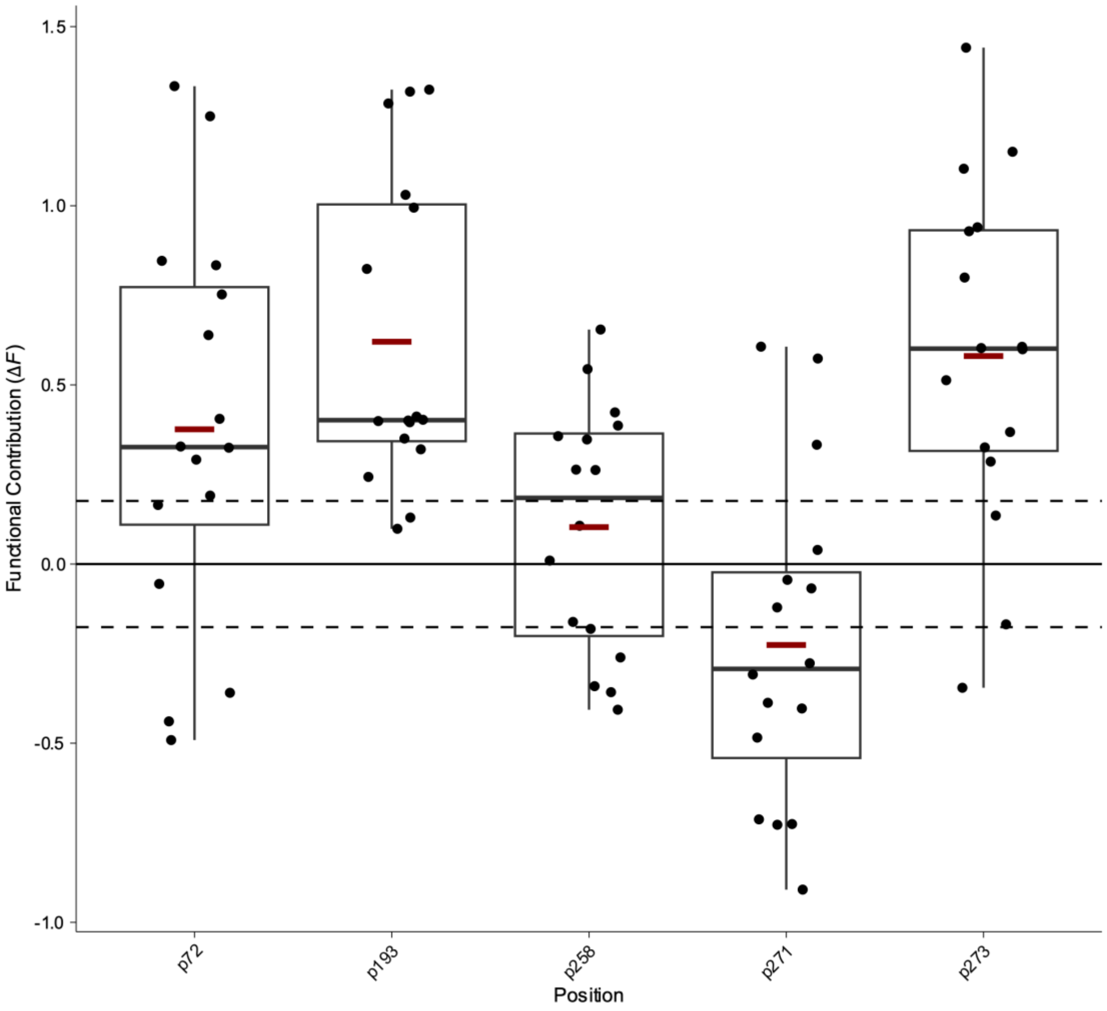

Box Plot 29 | Functional contribution of positional mutations in Methyl Parathion Hydrolase (MPH) for methyl parathion in copper metal conditions from Anderson *et al.*

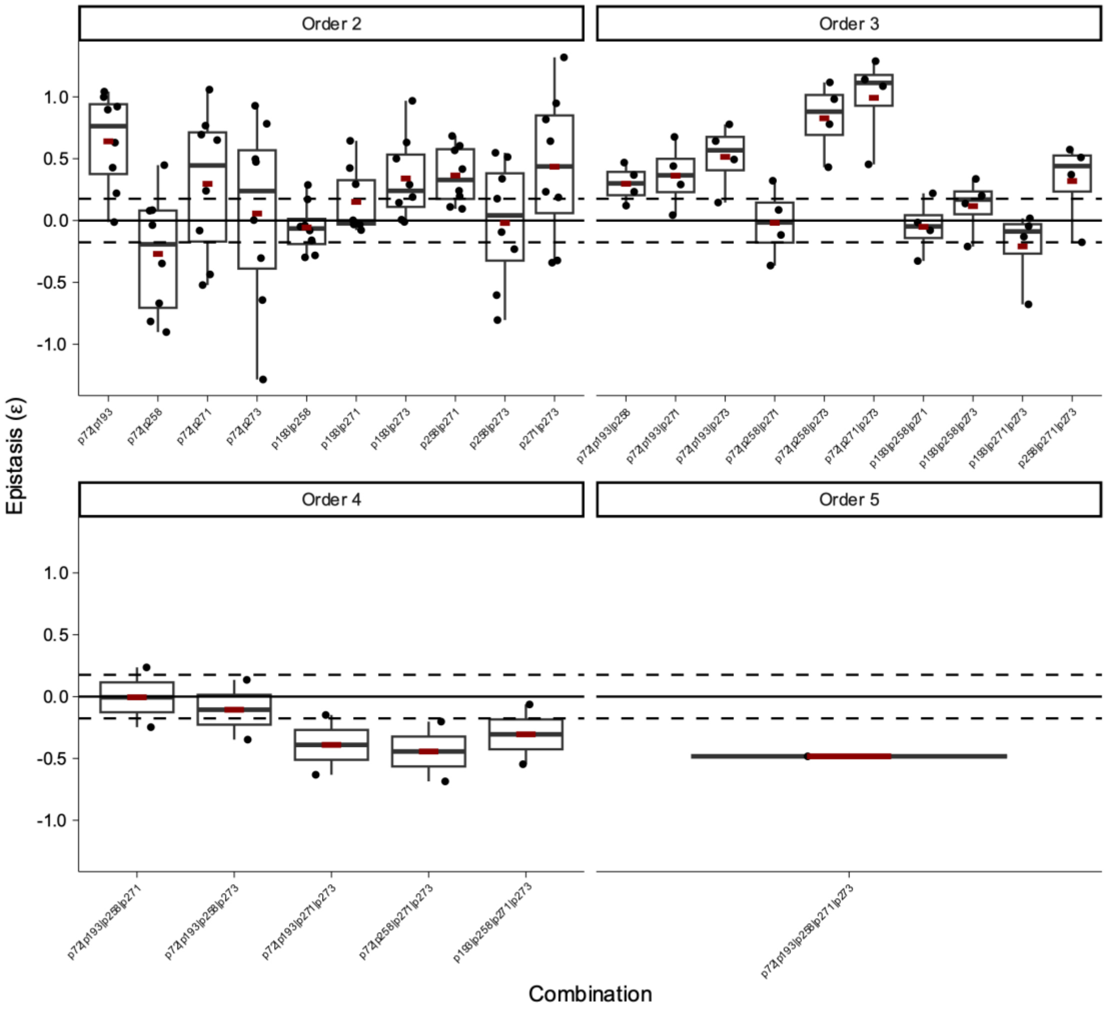

Box Plot 30 | Epistasis of combinatorial mutations in Methyl Parathion Hydrolase (MPH) for methyl parathion in copper metal conditions from Anderson *et al.*

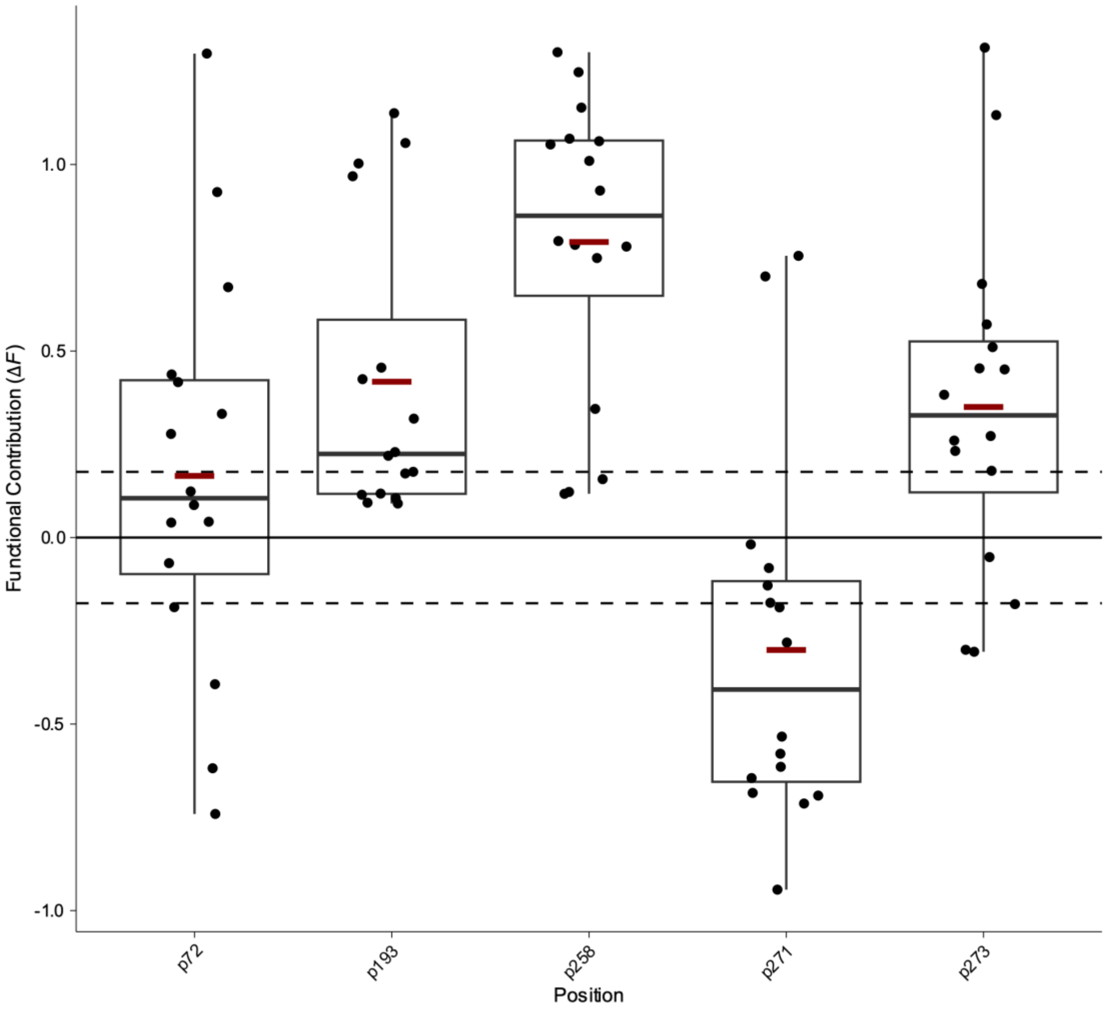

Box Plot 31 | Functional contribution of positional mutations in Methyl Parathion Hydrolase (MPH) for methyl parathion in magnesium metal conditions from Anderson *et al.*

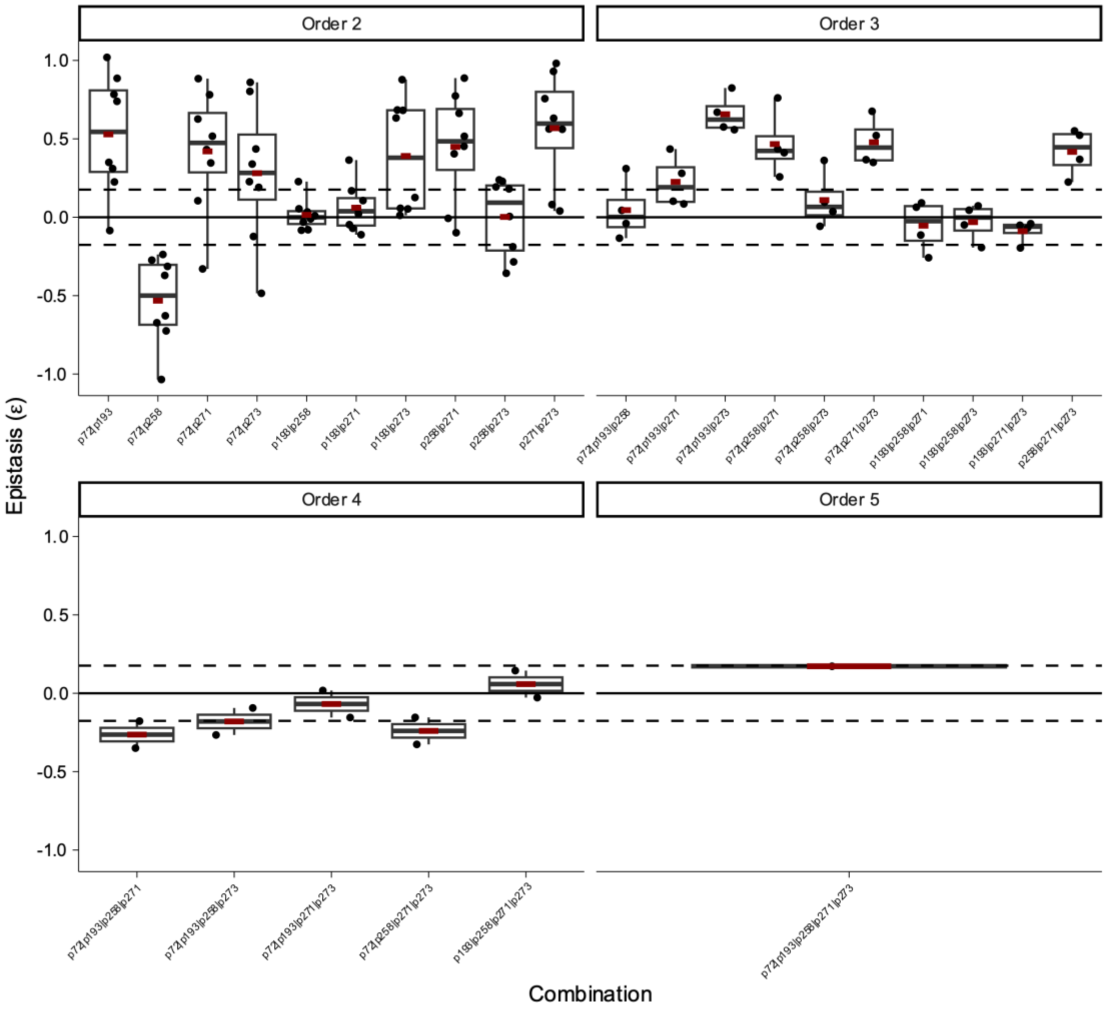

Box Plot 32 | Epistasis of combinatorial mutations in Methyl Parathion Hydrolase (MPH) for methyl parathion in magnesium metal conditions from Anderson *et al.*

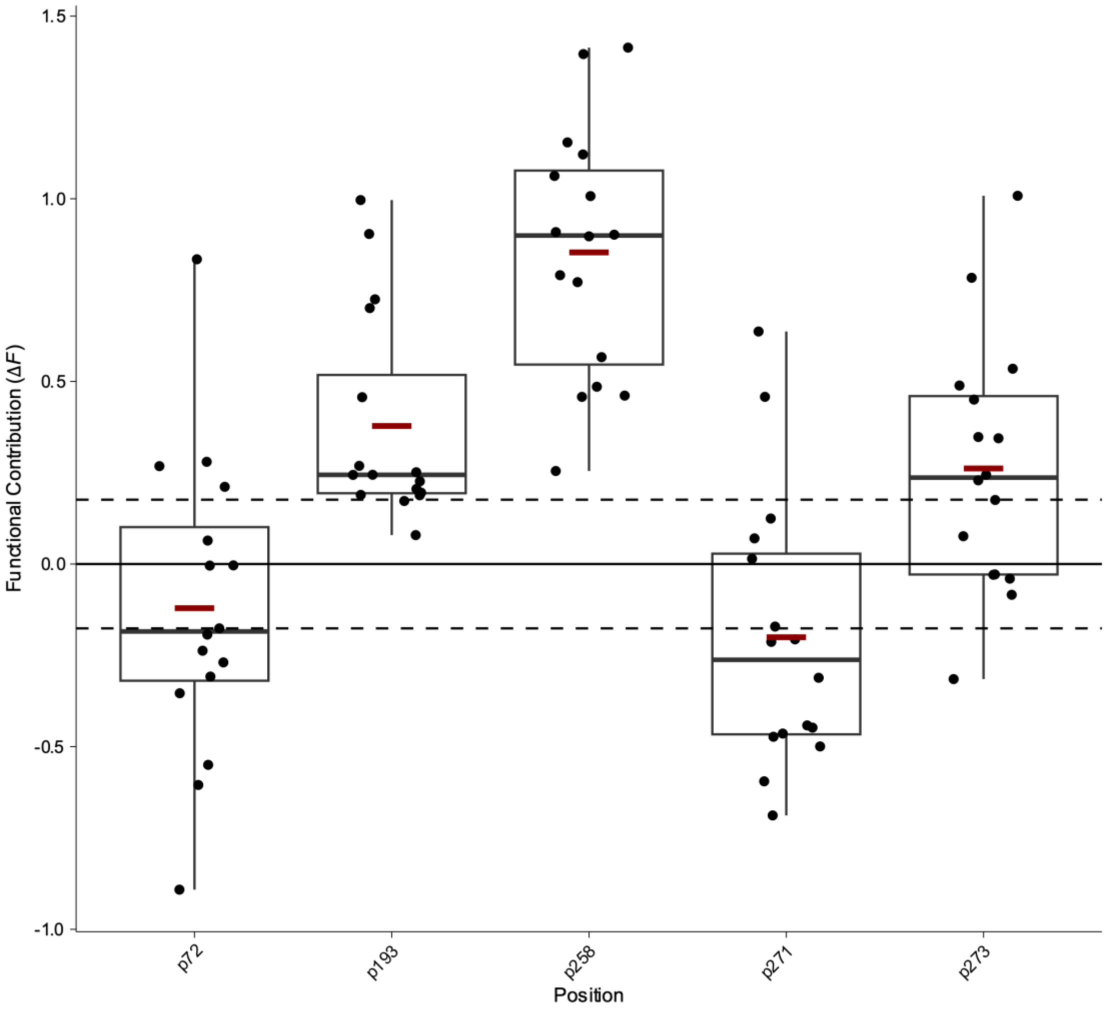

Box Plot 33 | Functional contribution of positional mutations in Methyl Parathion Hydrolase (MPH) for methyl parathion in manganese metal conditions from Anderson *et al.*

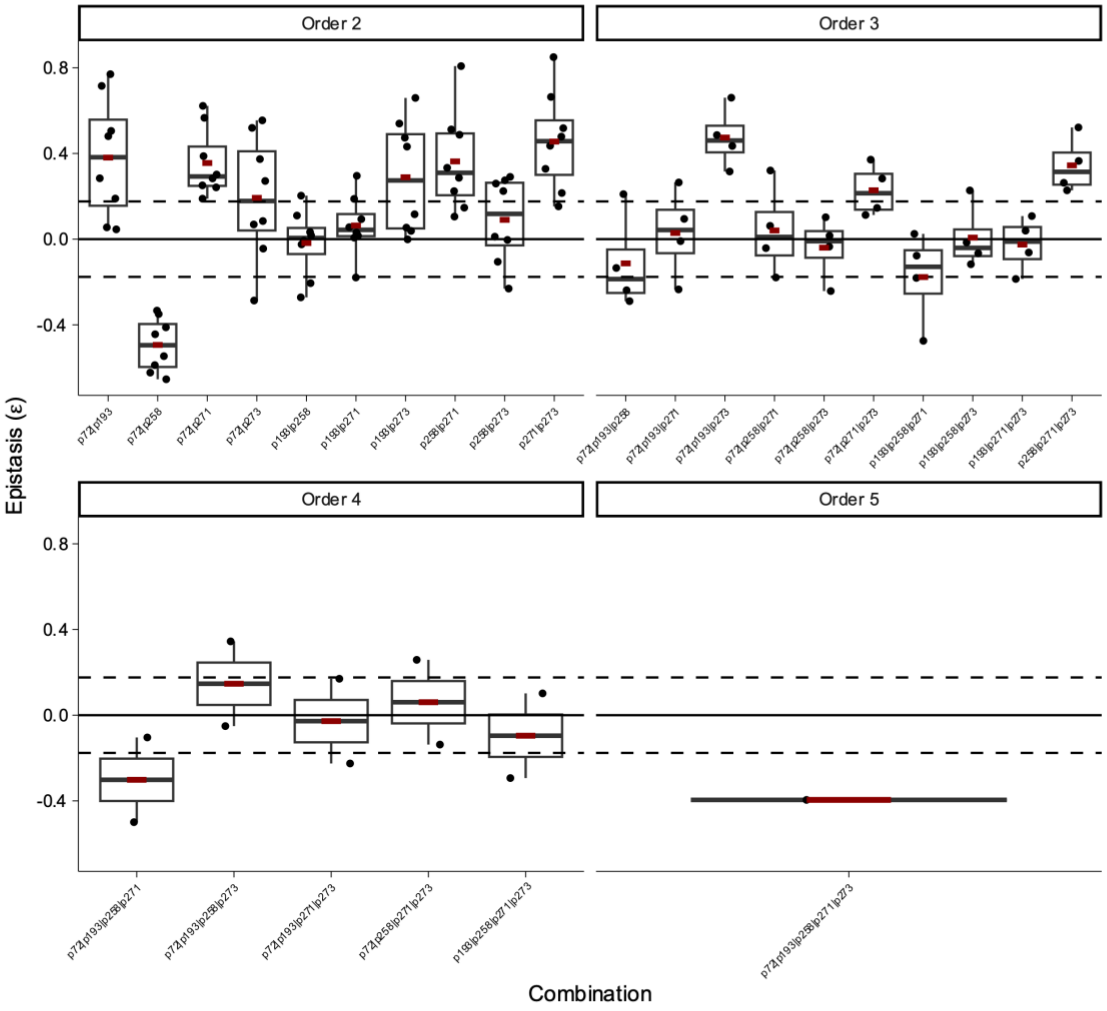

Box Plot 34 | Epistasis of combinatorial mutations in Methyl Parathion Hydrolase (MPH) for methyl parathion in manganese metal conditions from Anderson *et al.*

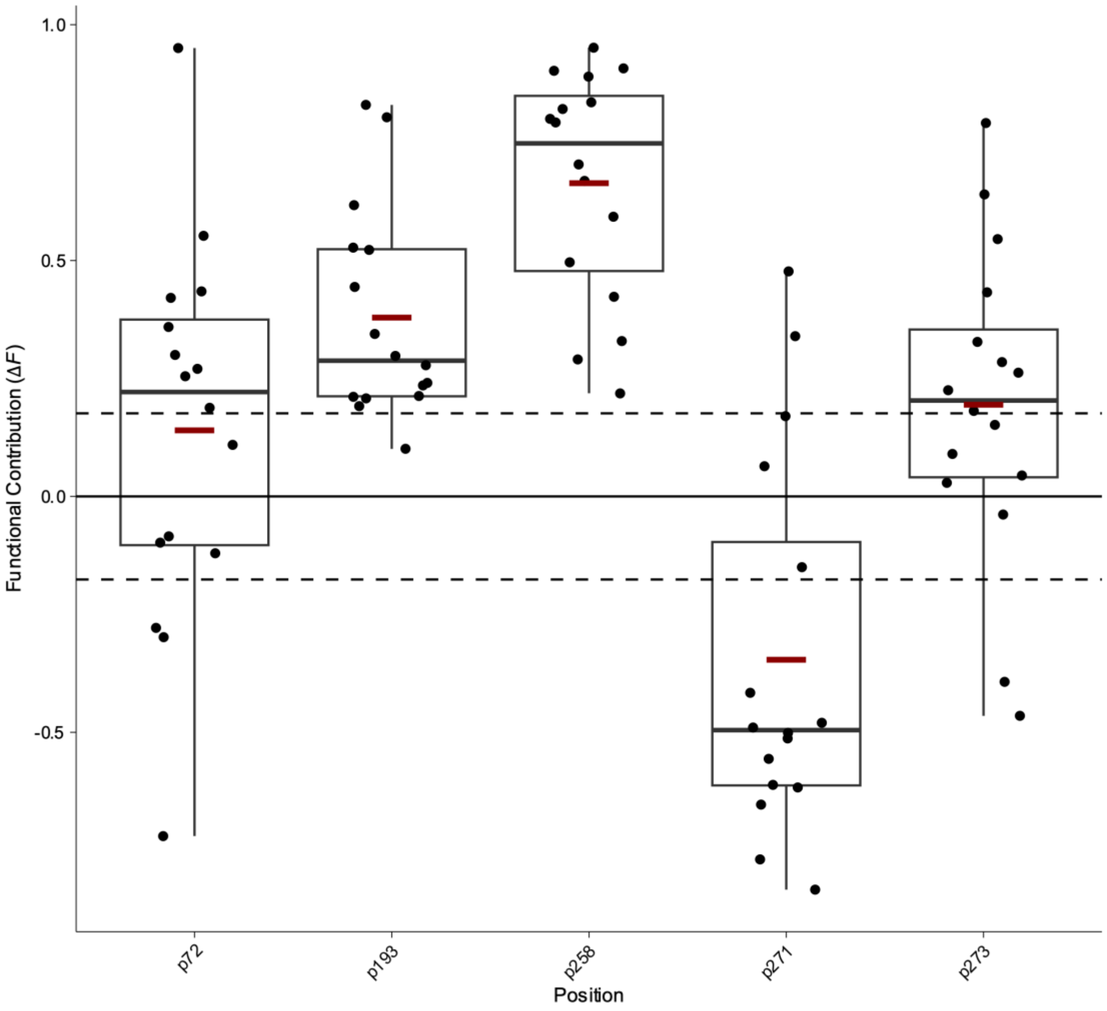

Box Plot 35 | Functional contribution of positional mutations in Methyl Parathion Hydrolase (MPH) for methyl parathion in nickel metal conditions from Anderson *et al.*

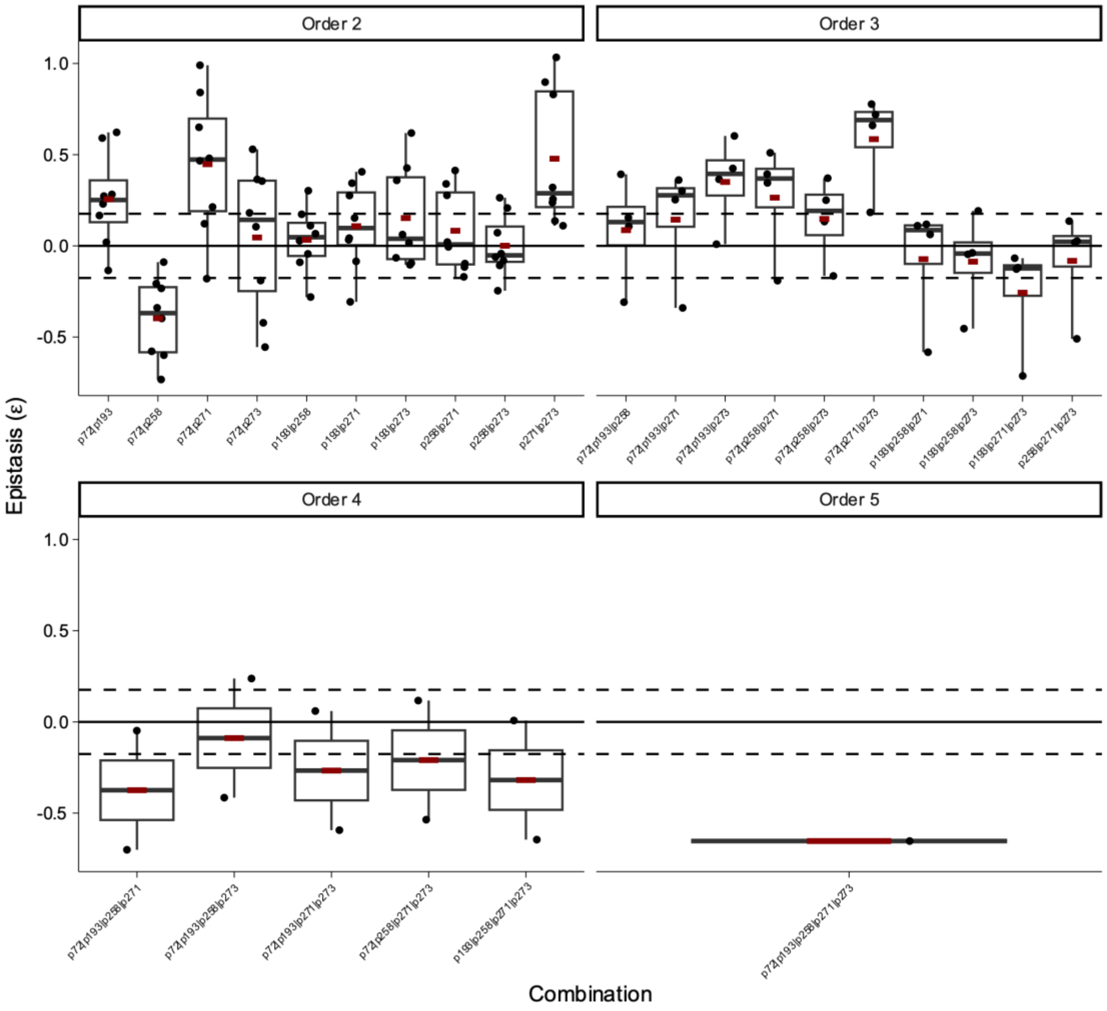

Box Plot 36 | Epistasis of combinatorial mutations in Methyl Parathion Hydrolase (MPH) for methyl parathion in nickel metal conditions from Anderson *et al.*

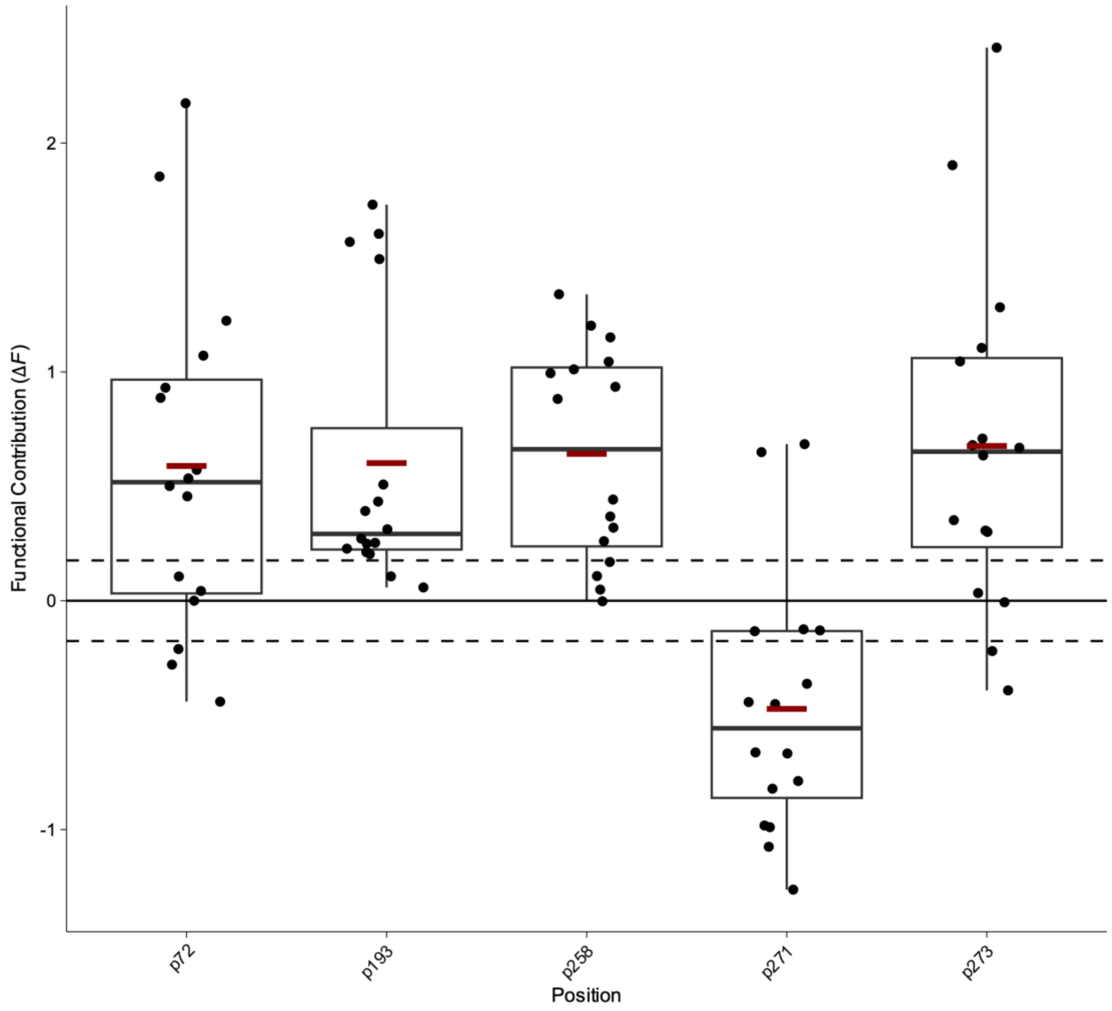

Box Plot 37 | Functional contribution of positional mutations in Methyl Parathion Hydrolase (MPH) for methyl parathion in zinc metal conditions from Anderson *et al.*

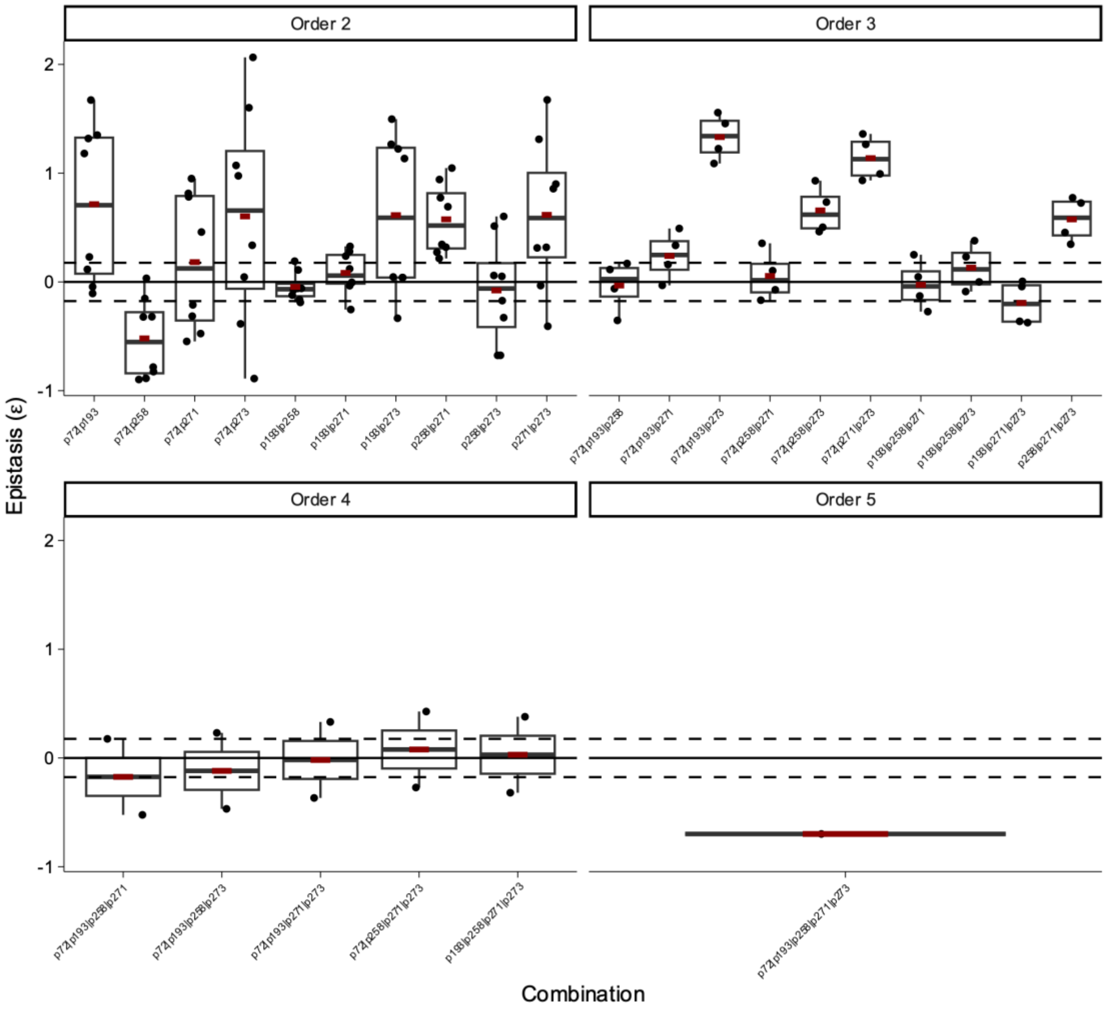

Box Plot 38 | Epistasis of combinatorial mutations in Methyl Parathion Hydrolase (MPH) for methyl parathion in zinc metal conditions from Anderson *et al.*

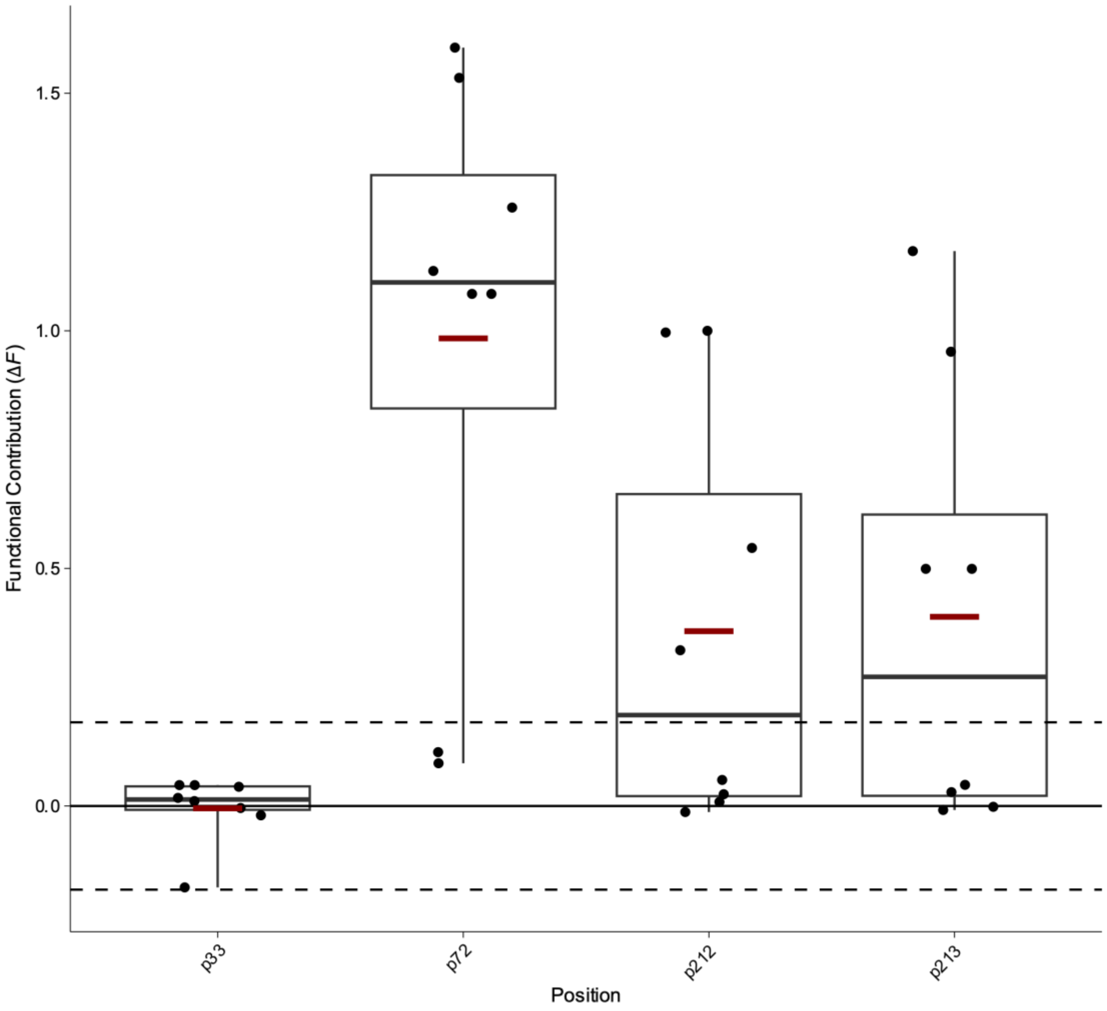

Box Plot 39 | Functional contribution of positional mutations in beta lactamase OXA-48 for ceftazidime hydrolysis in trajectory 1 from Fröhlich et al.

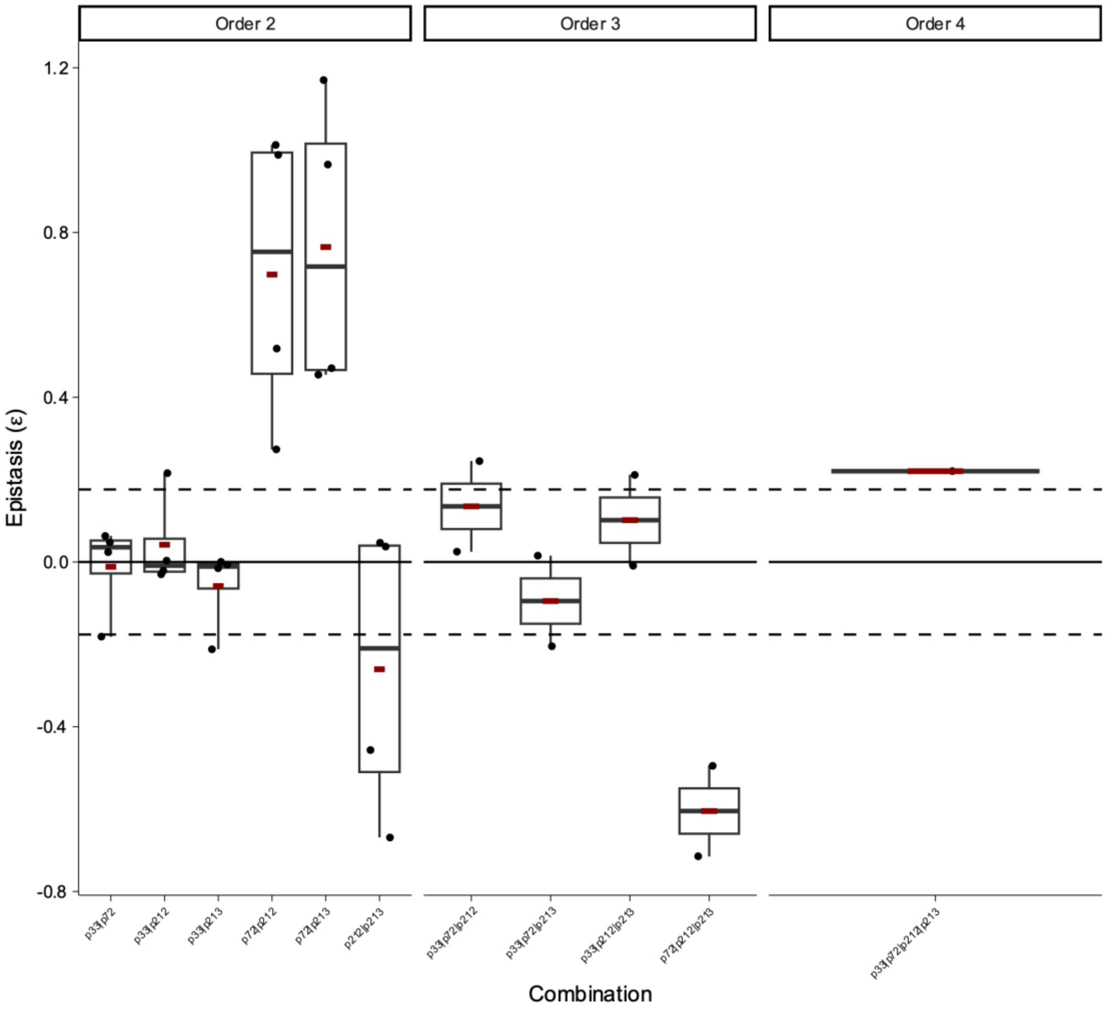

Box Plot 40 | Epistasis of combinatorial mutations in beta lactamase OXA-48 for ceftazidime hydrolysis in trajectory 1 from Fröhlich et al.

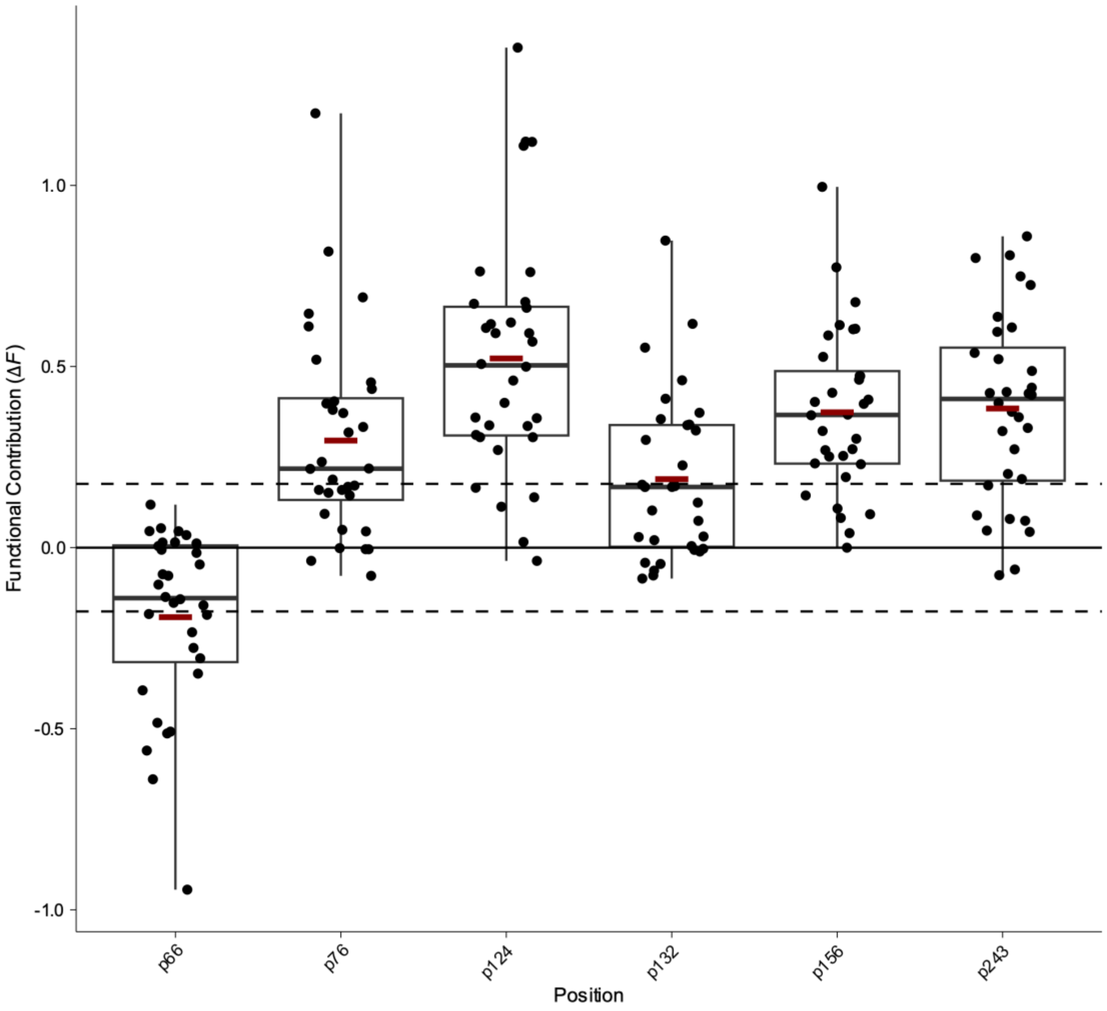

Box Plot 41 | Functional contribution of positional mutations in beta lactamase OXA-48 for ceftazidime hydrolysis in trajectory 2 from Fröhlich et al.

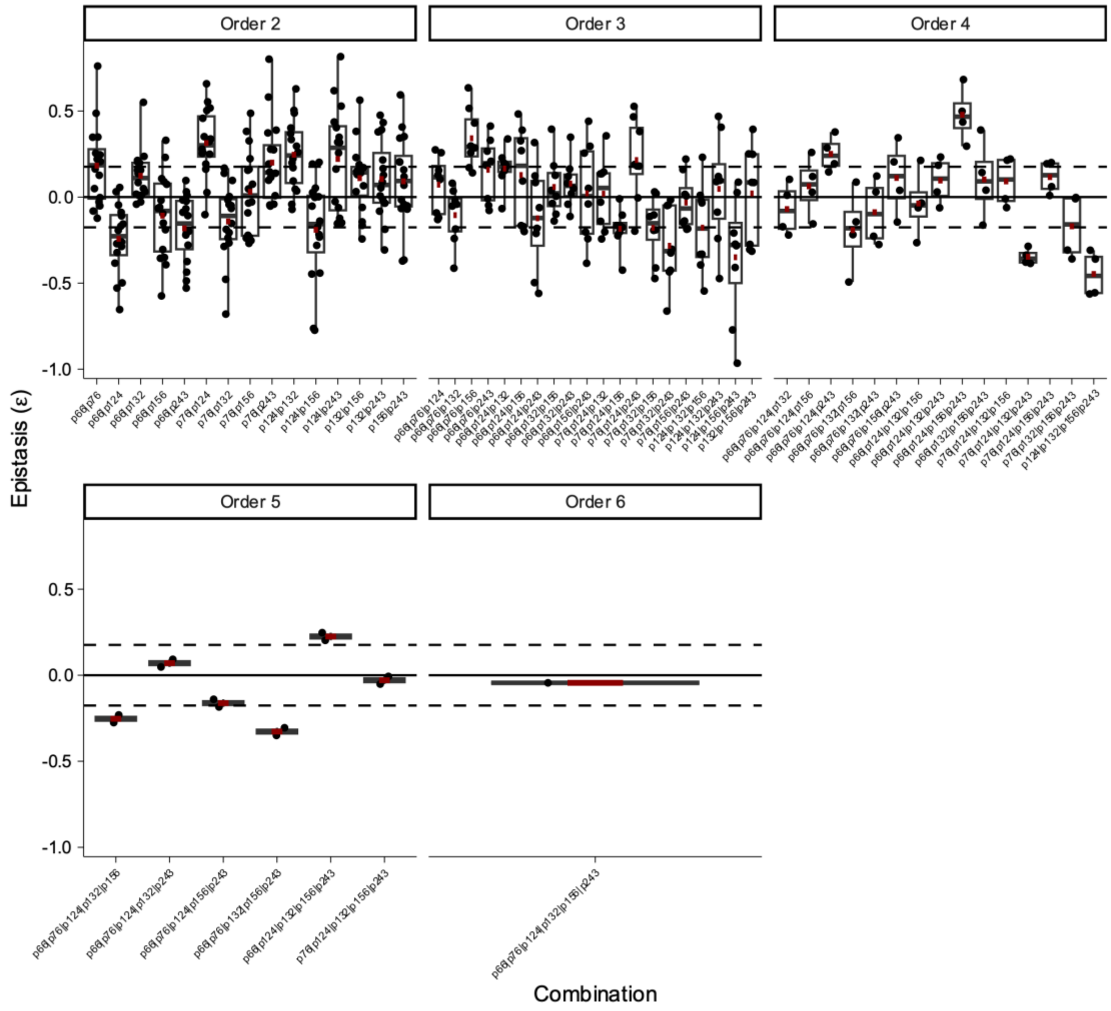

Box Plot 42 | Epistasis of combinatorial mutations in beta lactamase OXA-48 for ceftazidime hydrolysis in trajectory 2 from Fröhlich et al.

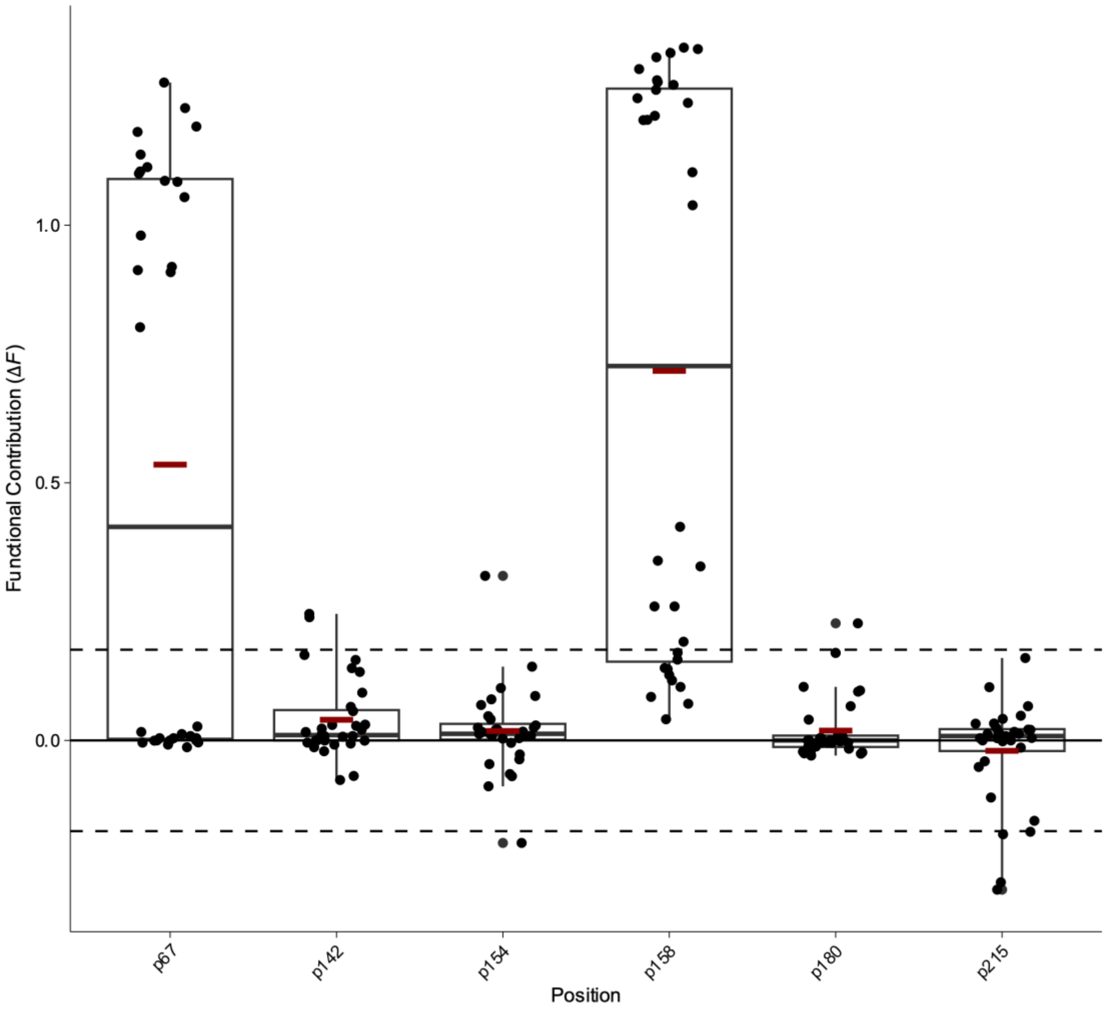

Box Plot 43 | Functional contribution of positional mutations in beta lactamase OXA-48 for ceftazidime hydrolysis in trajectory 3 from Fröhlich et al.

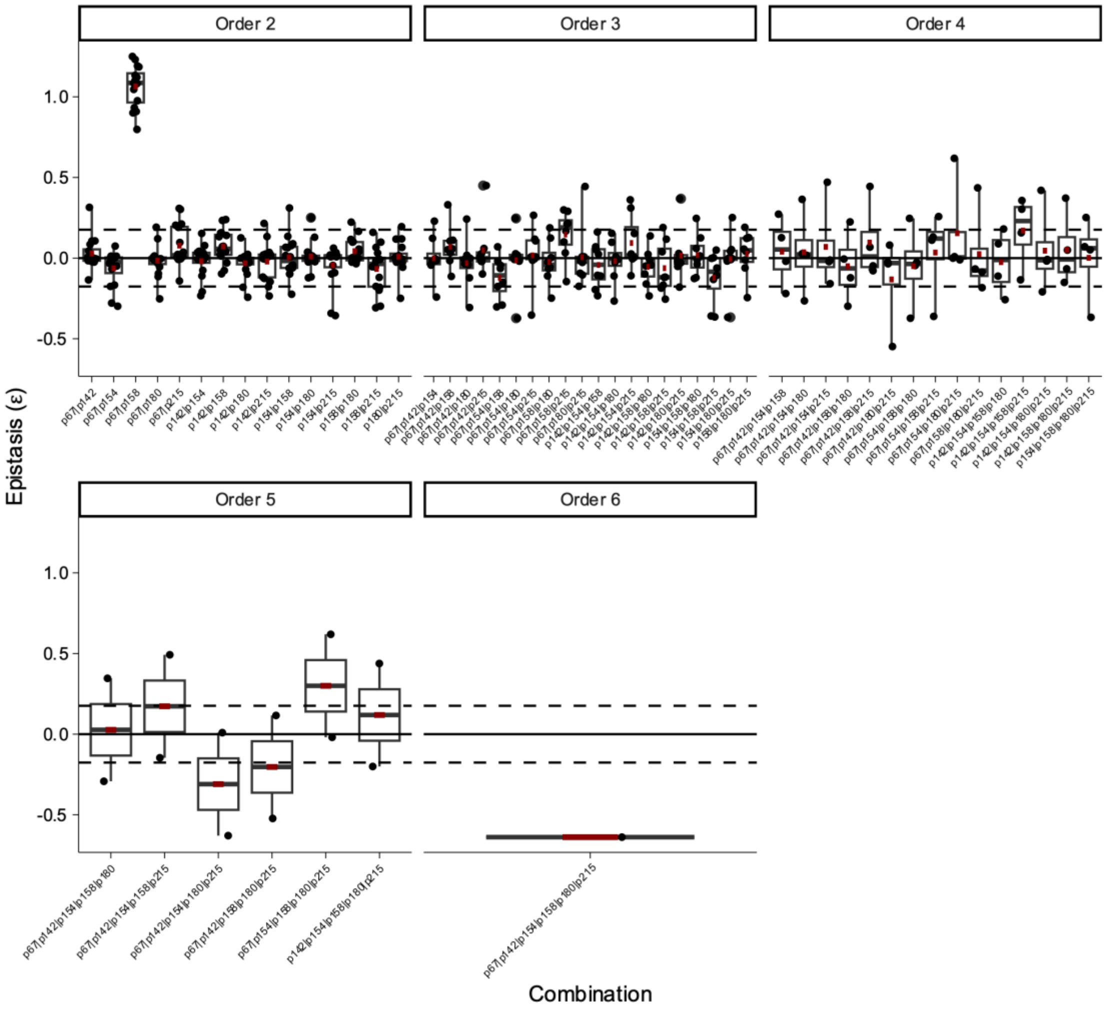

Box Plot 44 | Epistasis of combinatorial mutations in beta lactamase OXA-48 for ceftazidime hydrolysis in trajectory 3 from Fröhlich et al.

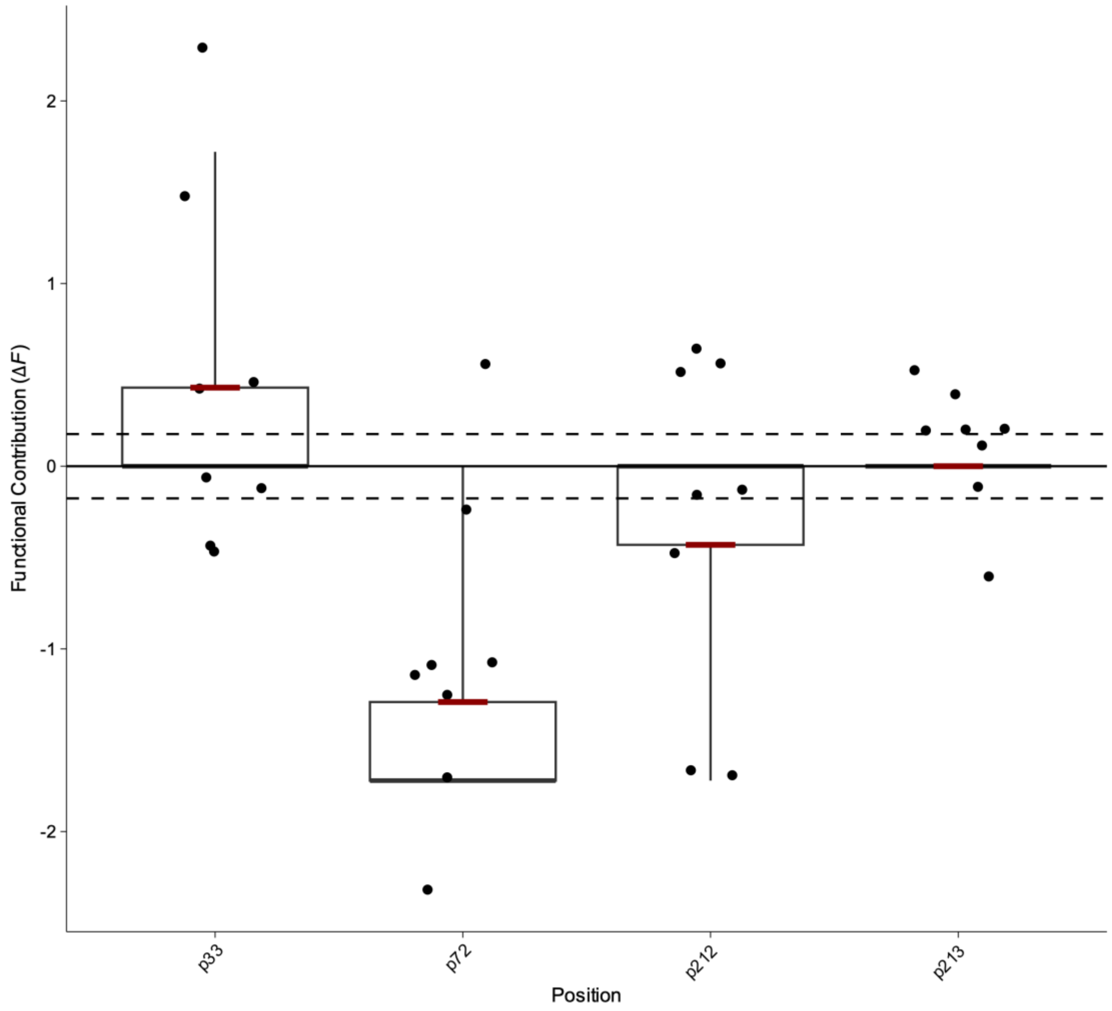

Box Plot 45 | Functional contribution of positional mutations in beta lactamase OXA-48 for piperacillin hydrolysis in trajectory 1 from Fröhlich et al.

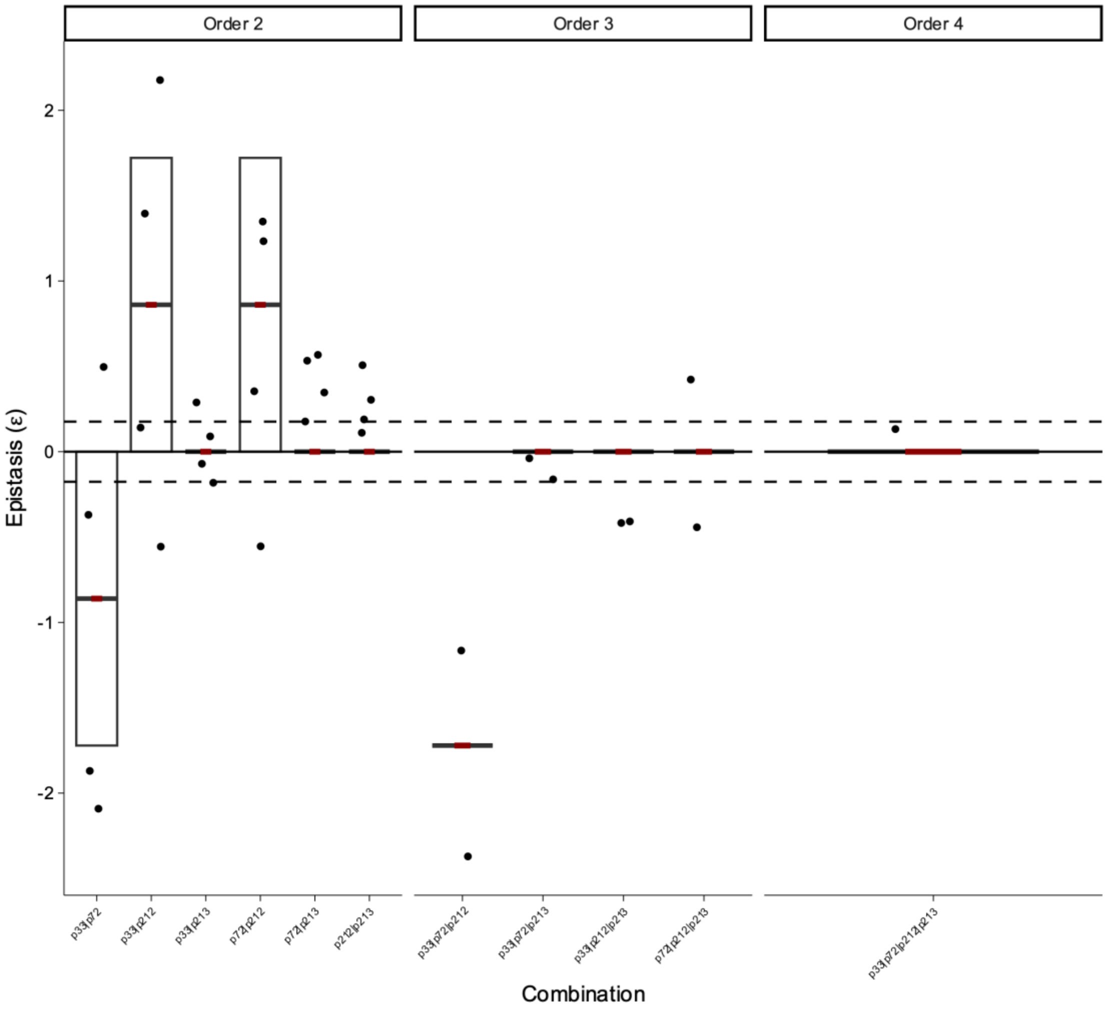

Box Plot 46 | Epistasis of combinatorial mutations in beta lactamase OXA-48 for piperacillin hydrolysis in trajectory 1 from Fröhlich et al.

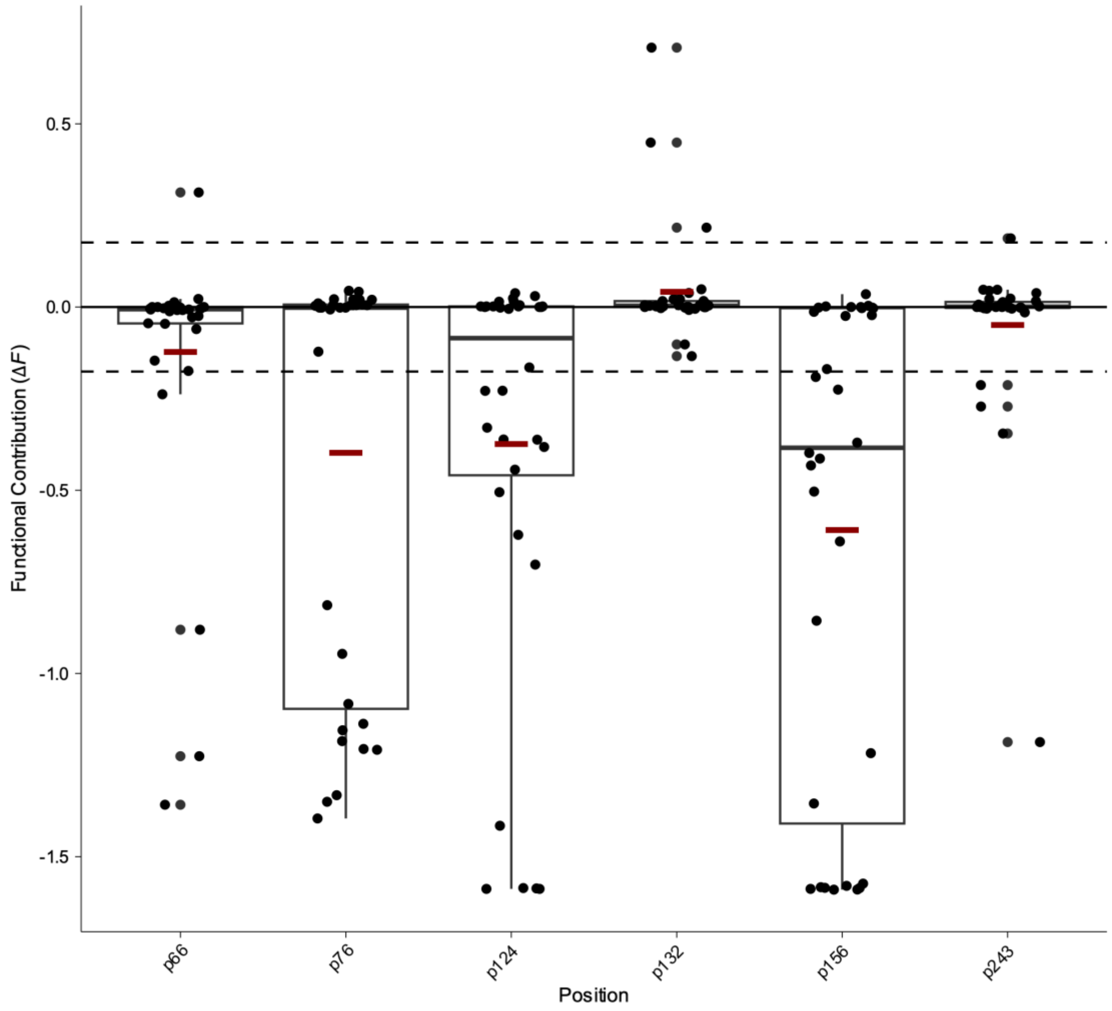

Box Plot 47 | Functional contribution of positional mutations in beta lactamase OXA-48 for piperacillin hydrolysis in trajectory 2 from Fröhlich et al.

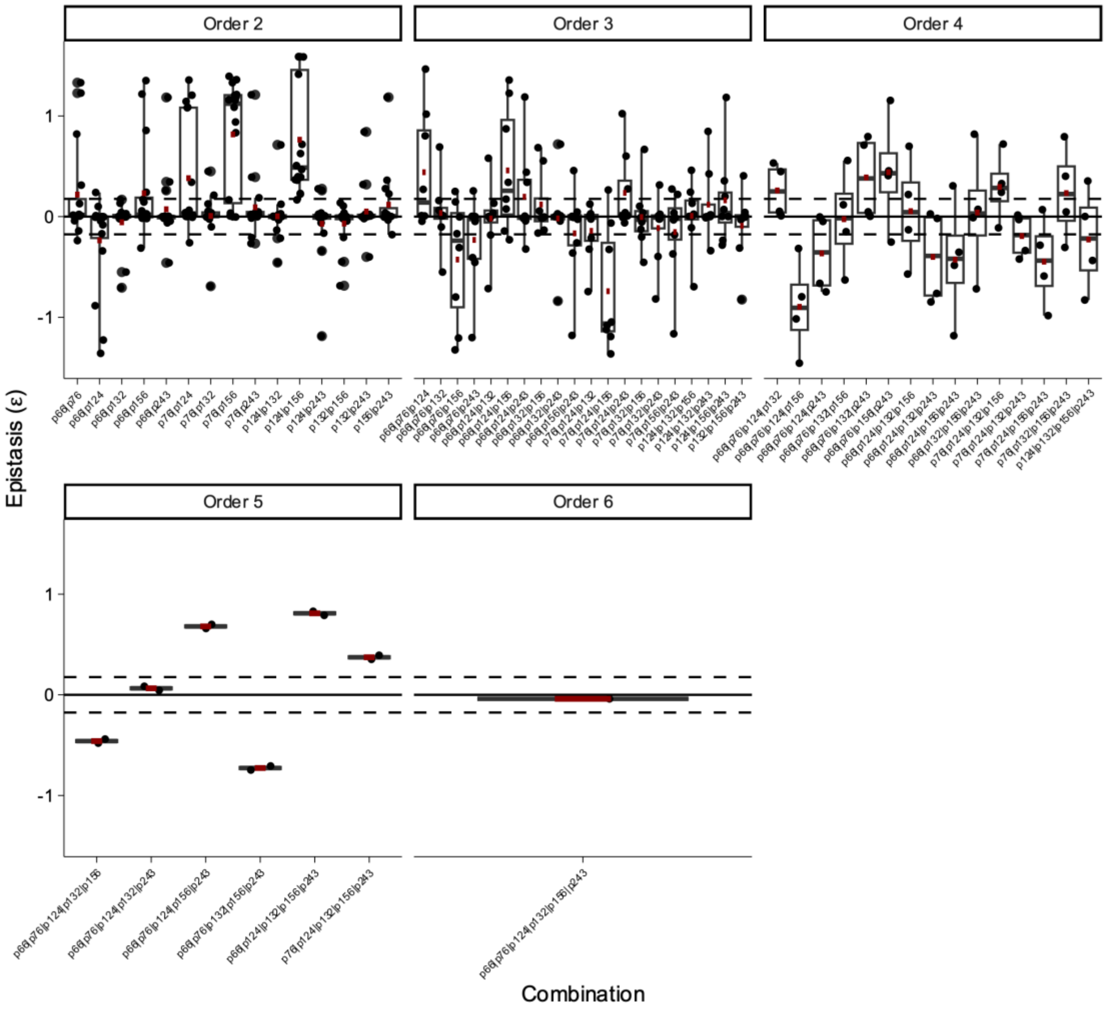

Box Plot 48 | Epistasis of combinatorial mutations in beta lactamase OXA-48 for piperacillin hydrolysis in trajectory 2 from Fröhlich et al.

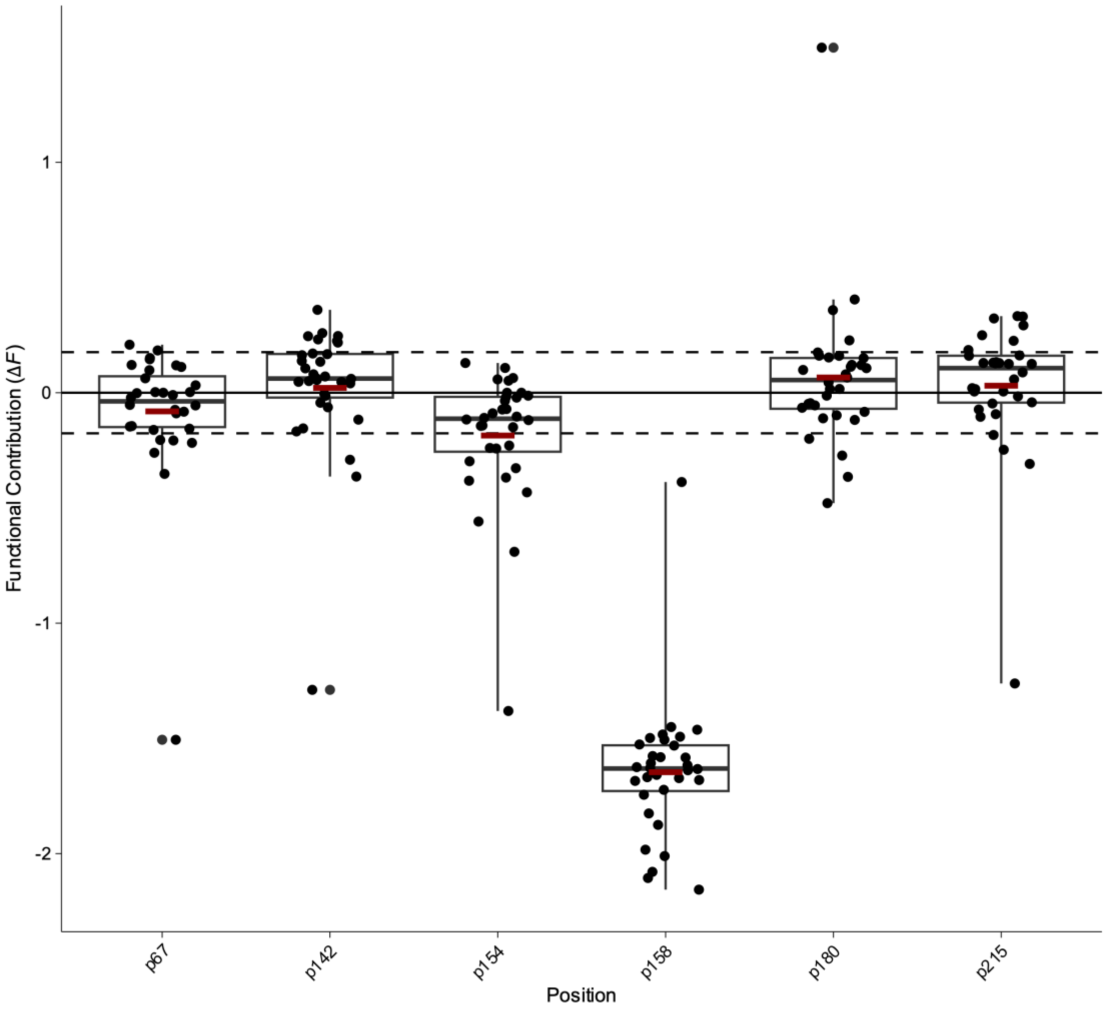

Box Plot 49 | Functional contribution of positional mutations in beta lactamase OXA-48 for piperacillin hydrolysis in trajectory 3 from Fröhlich et al.

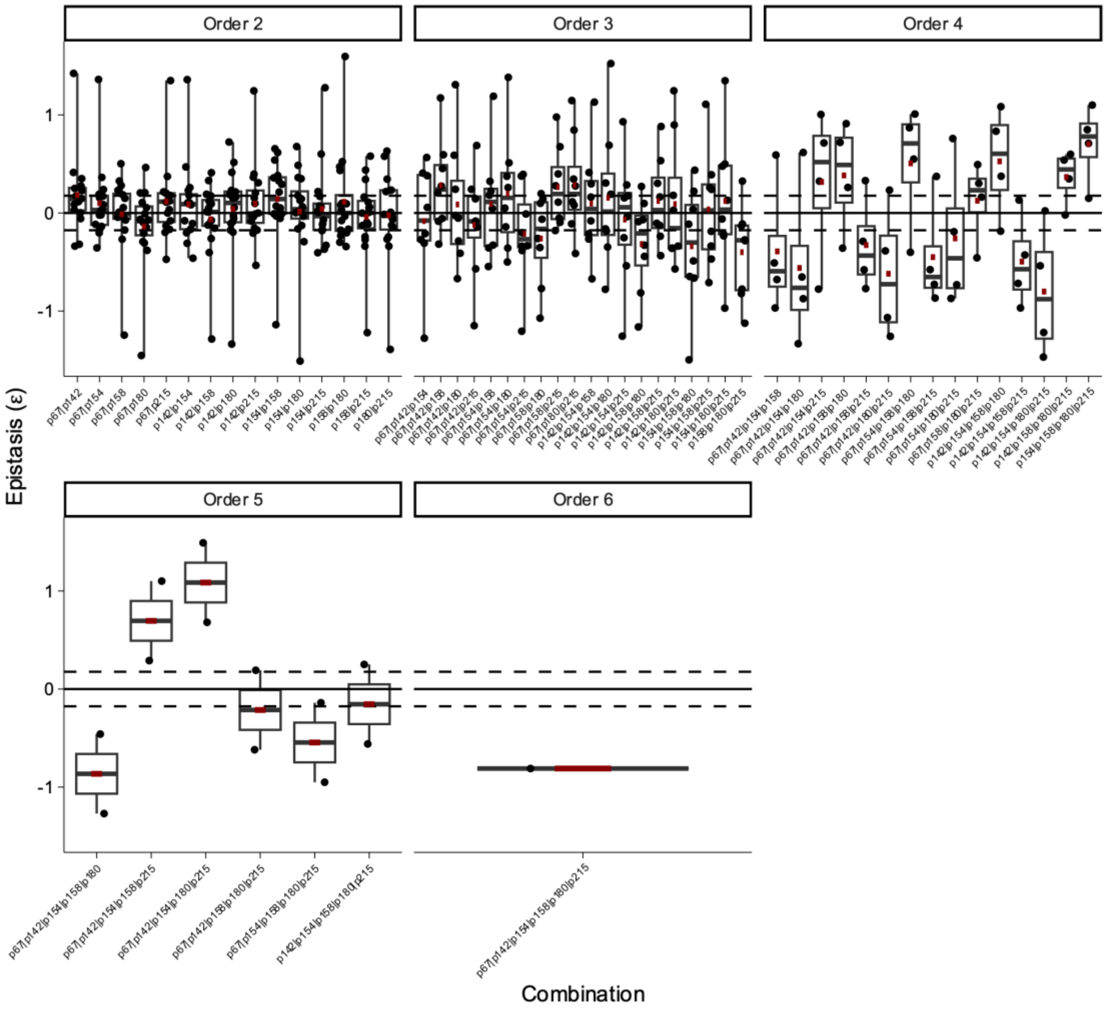

Box Plot 50 | Epistasis of combinatorial mutations in beta lactamase OXA-48 for piperacillin hydrolysis in trajectory 3 from Fröhlich et al.

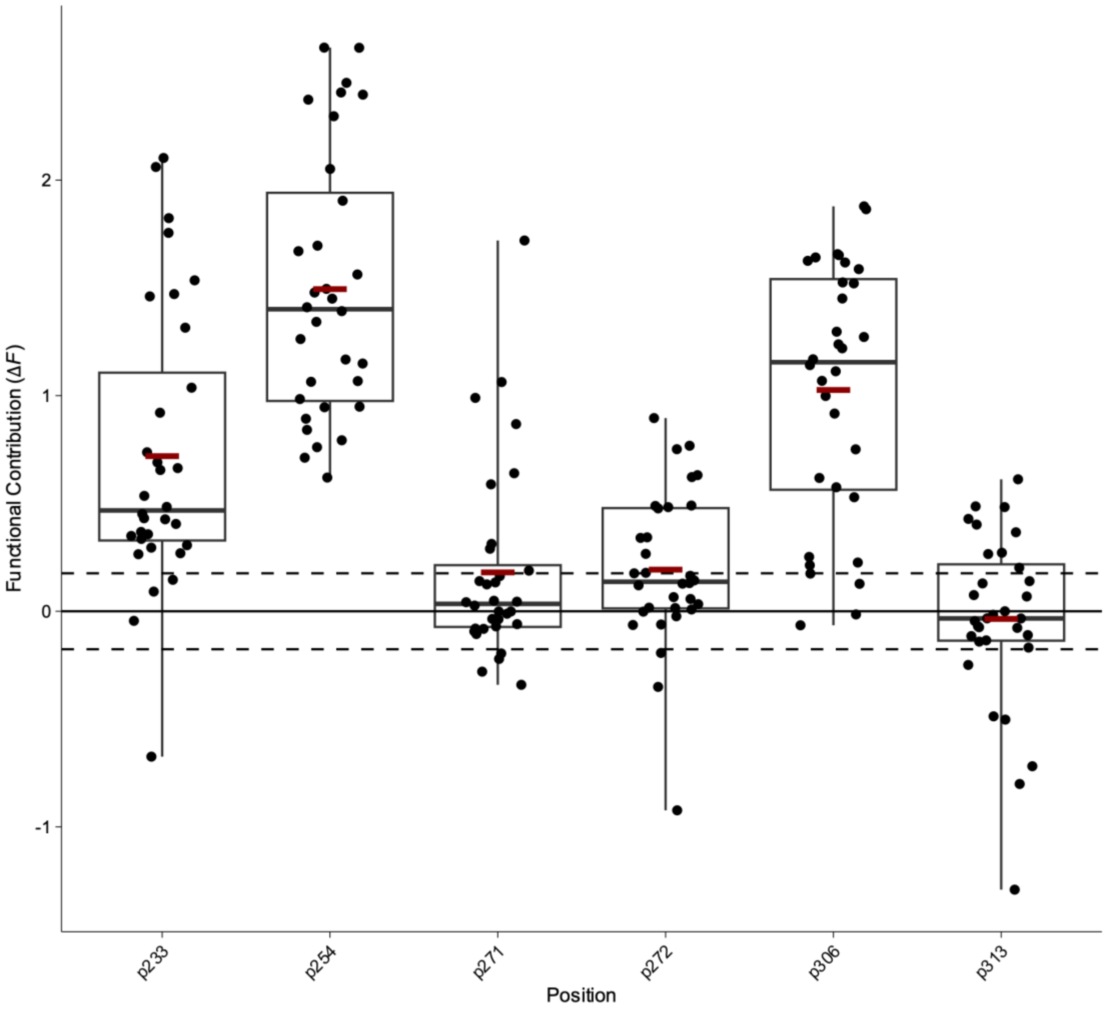

Box Plot 53 | Functional contribution of positional mutations in phosphotriesterase (PTE) for 2-napthyl hexanoate (2NH) hydrolysis from this publication

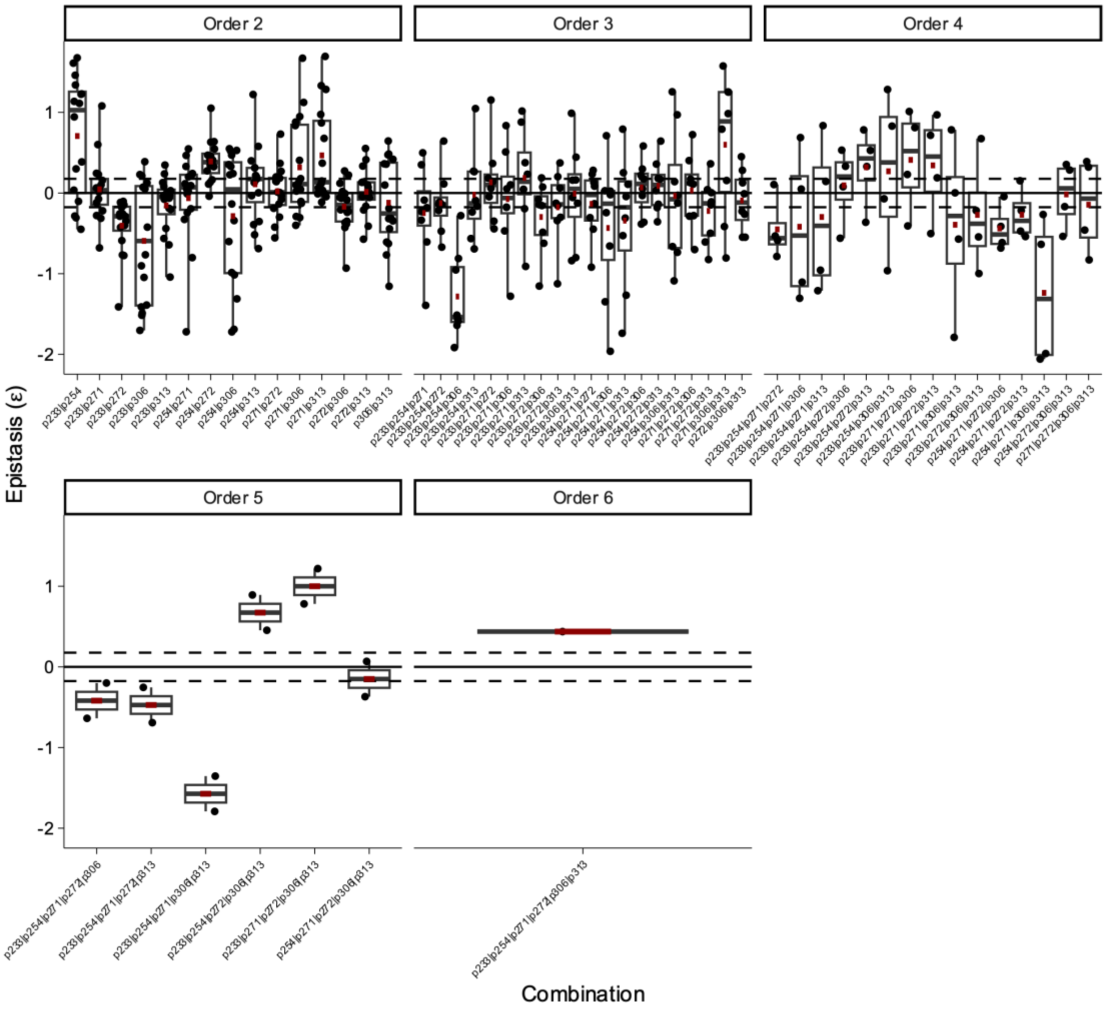

Box Plot 52 | Epistasis of combinatorial mutations in phosphotriesterase (PTE) for 2-napthyl hexanoate (2NH) hydrolysis from this publication

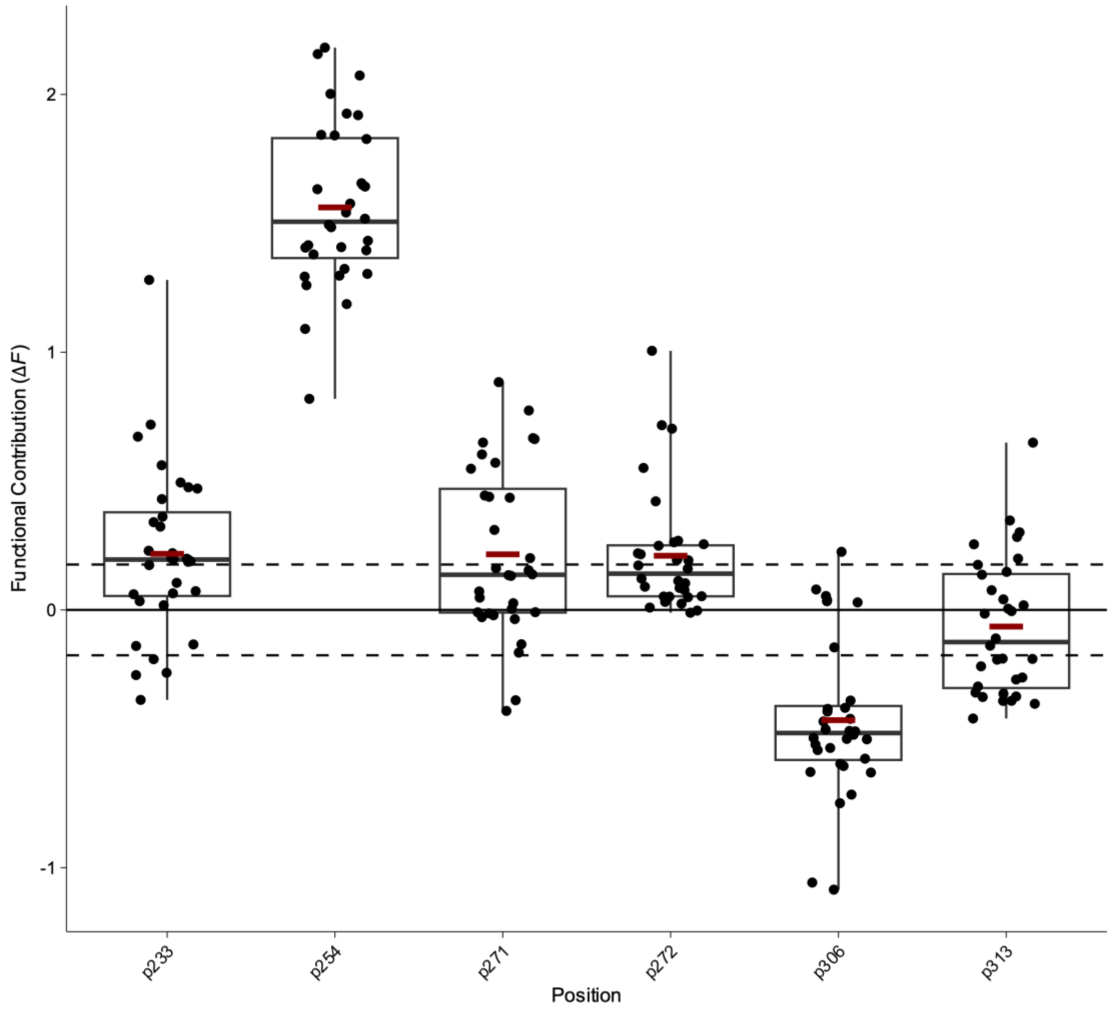

Box Plot 53 | Functional contribution of positional mutations in phosphotriesterase (PTE) for butyrate hydrolysis from Miton *et al.*

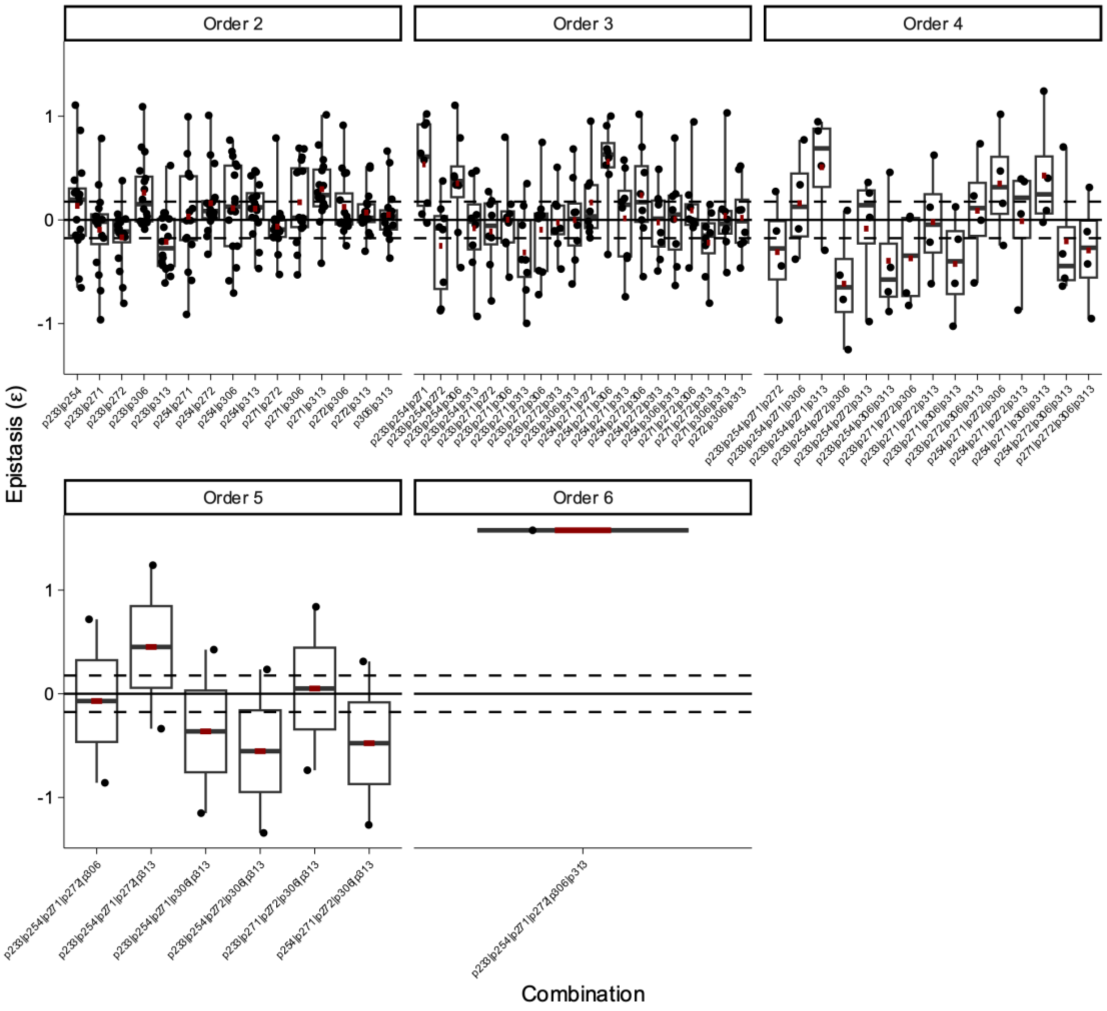

Box Plot 54 | Epistasis of combinatorial mutations in phosphotriesterase (PTE) for butyrate hydrolysis from Miton *et al.*

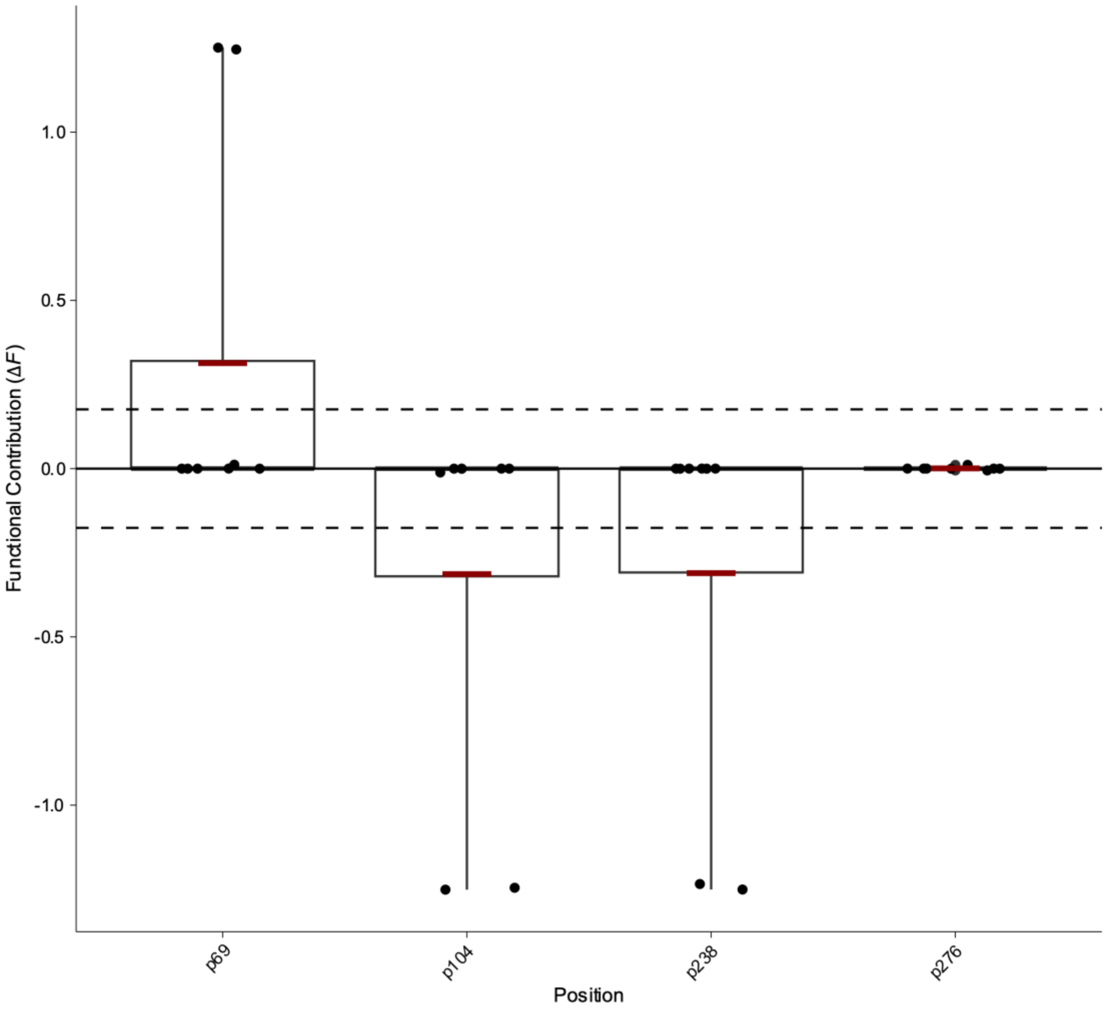

Box Plot 55 | Functional contribution of positional mutations in of beta lactamase TEM in AM antibiotic from Mira *et al.*

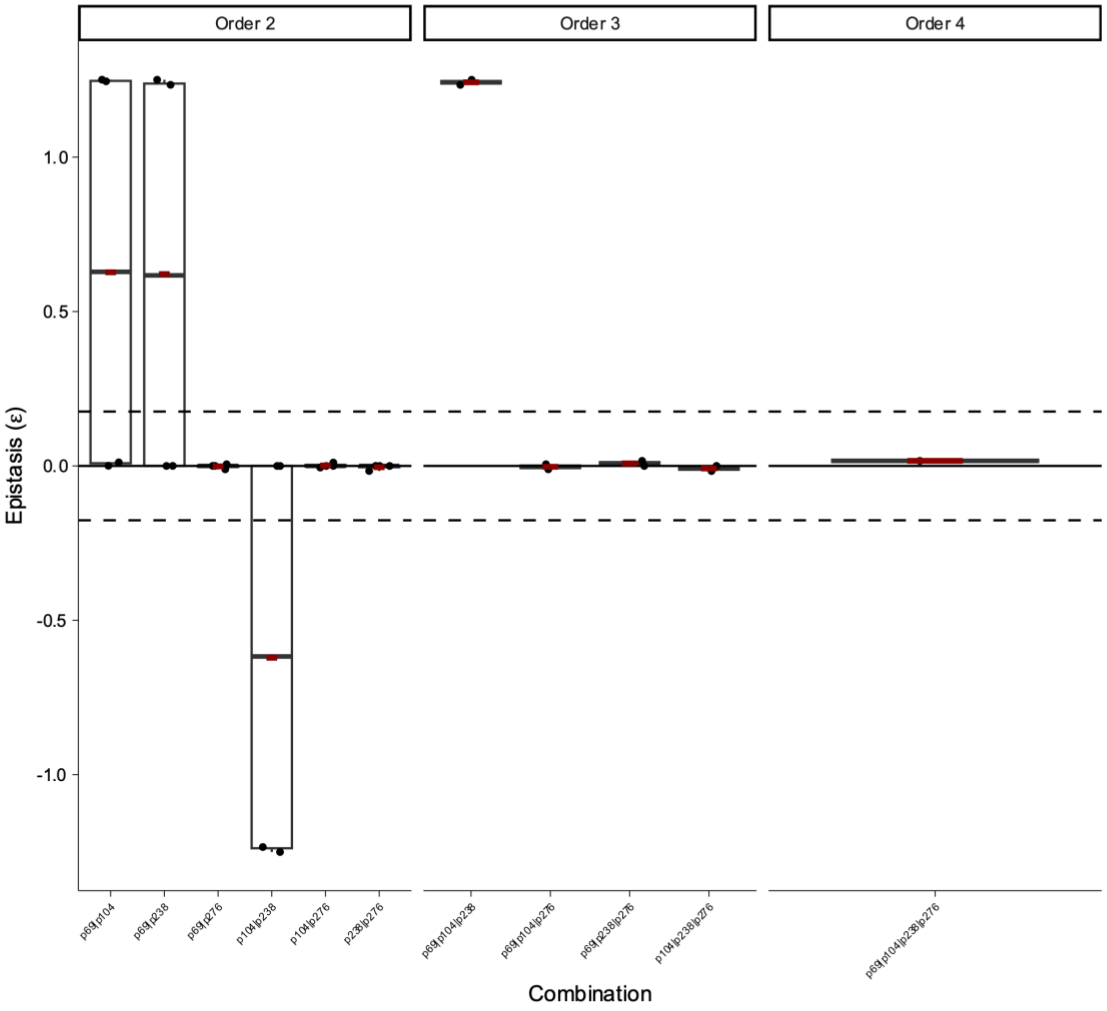

Box Plot 56 | Epistasis of combinatorial mutations in beta lactamase TEM in AM antibiotic Mira *et al.*

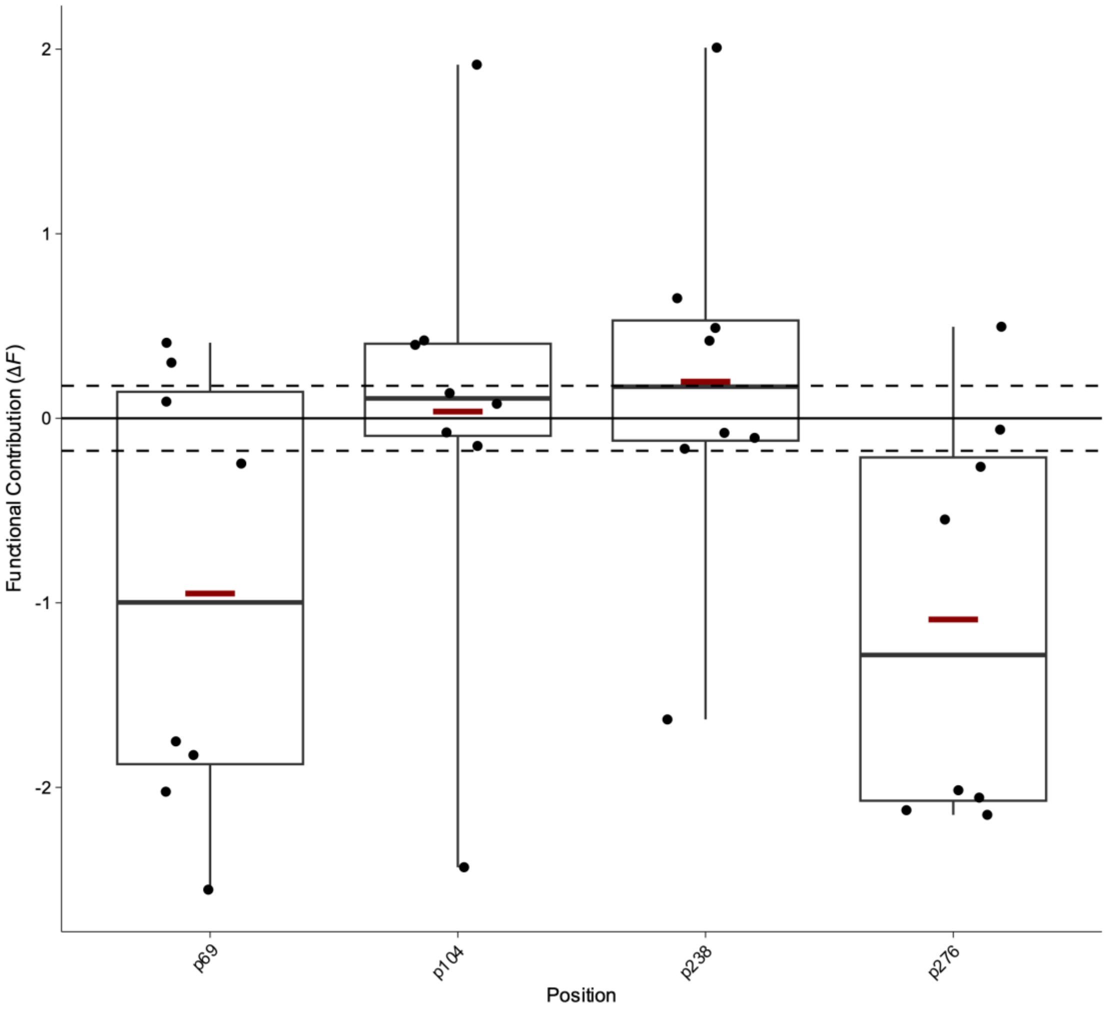

Box Plot 57 | Functional contribution of positional mutations in of beta lactamase TEM in CEC antibiotic Mira *et al.*

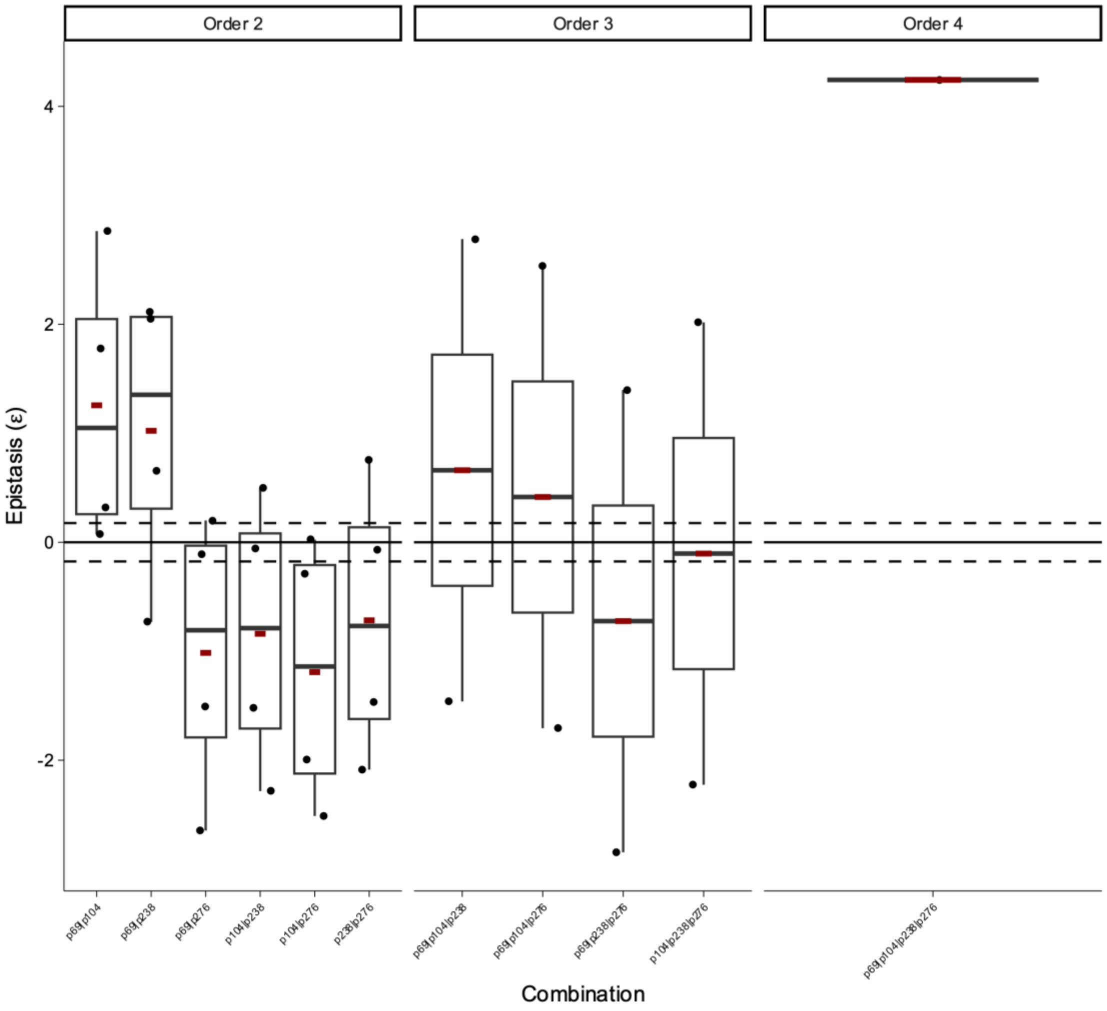

Box Plot 58 | Epistasis of combinatorial mutations in beta lactamase TEM in CEC antibiotic Mira *et al.*

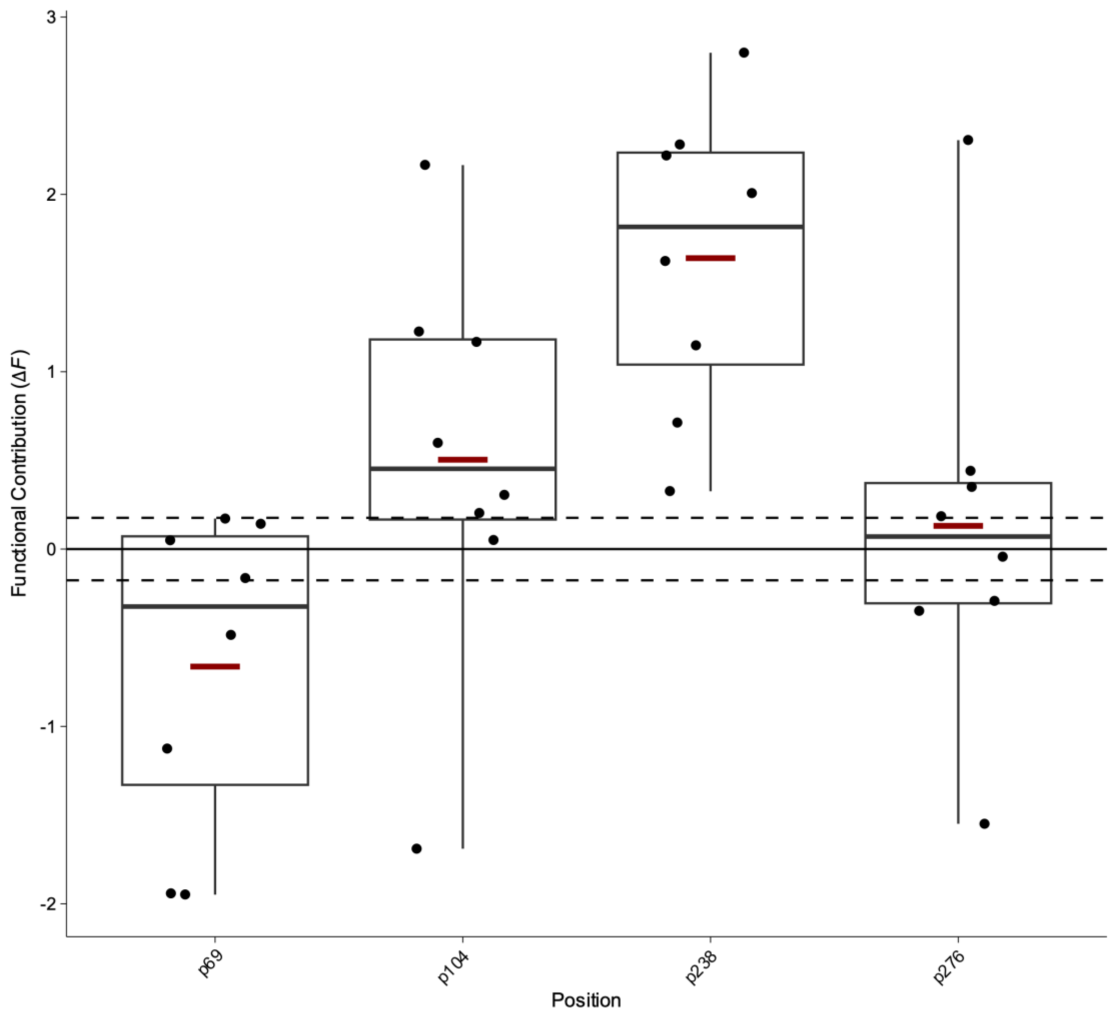

Box Plot 59 | Functional contribution of positional mutations in of beta lactamase TEM in CPD antibiotic Mira *et al.*

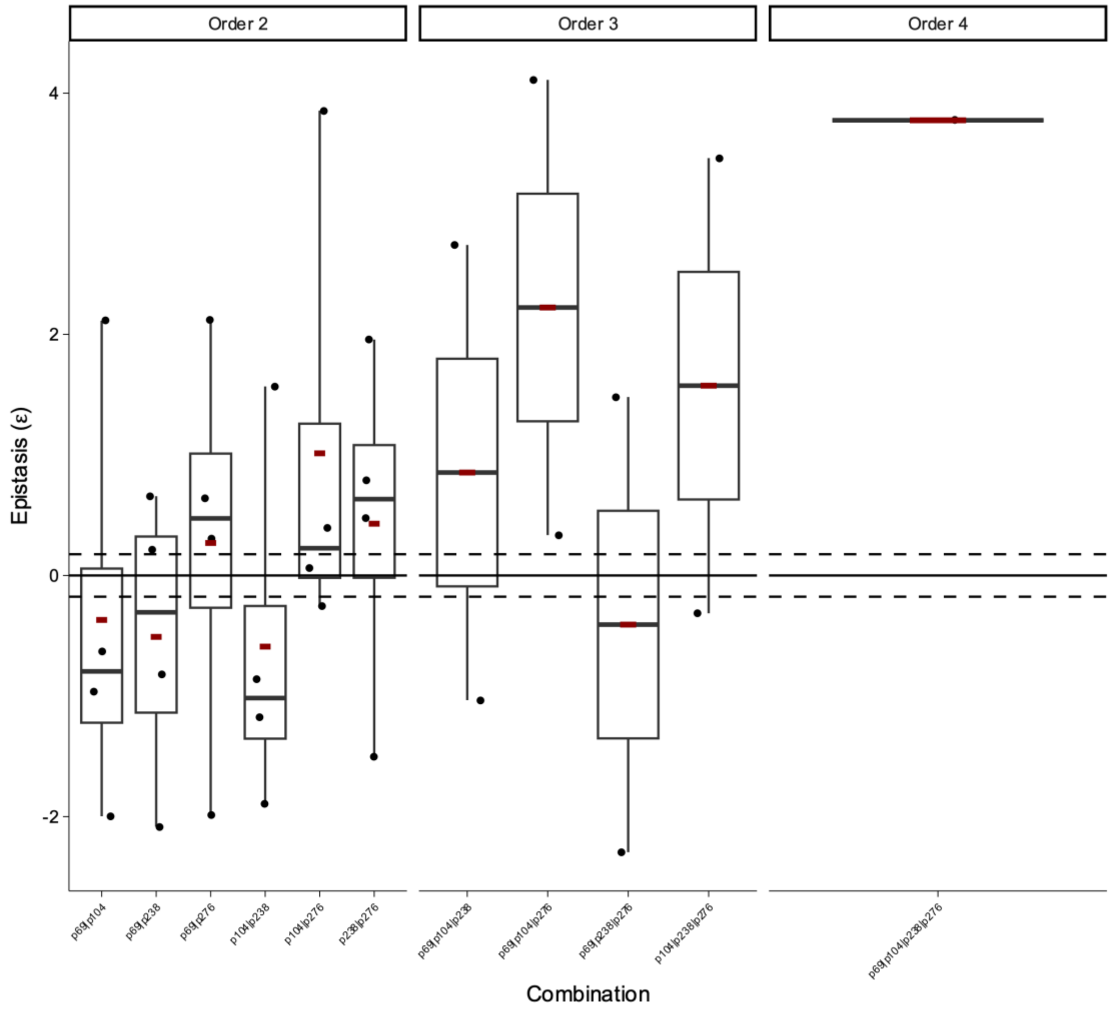

Box Plot 60 | Epistasis of combinatorial mutations in beta lactamase TEM in CPD antibiotic Mira *et al.*

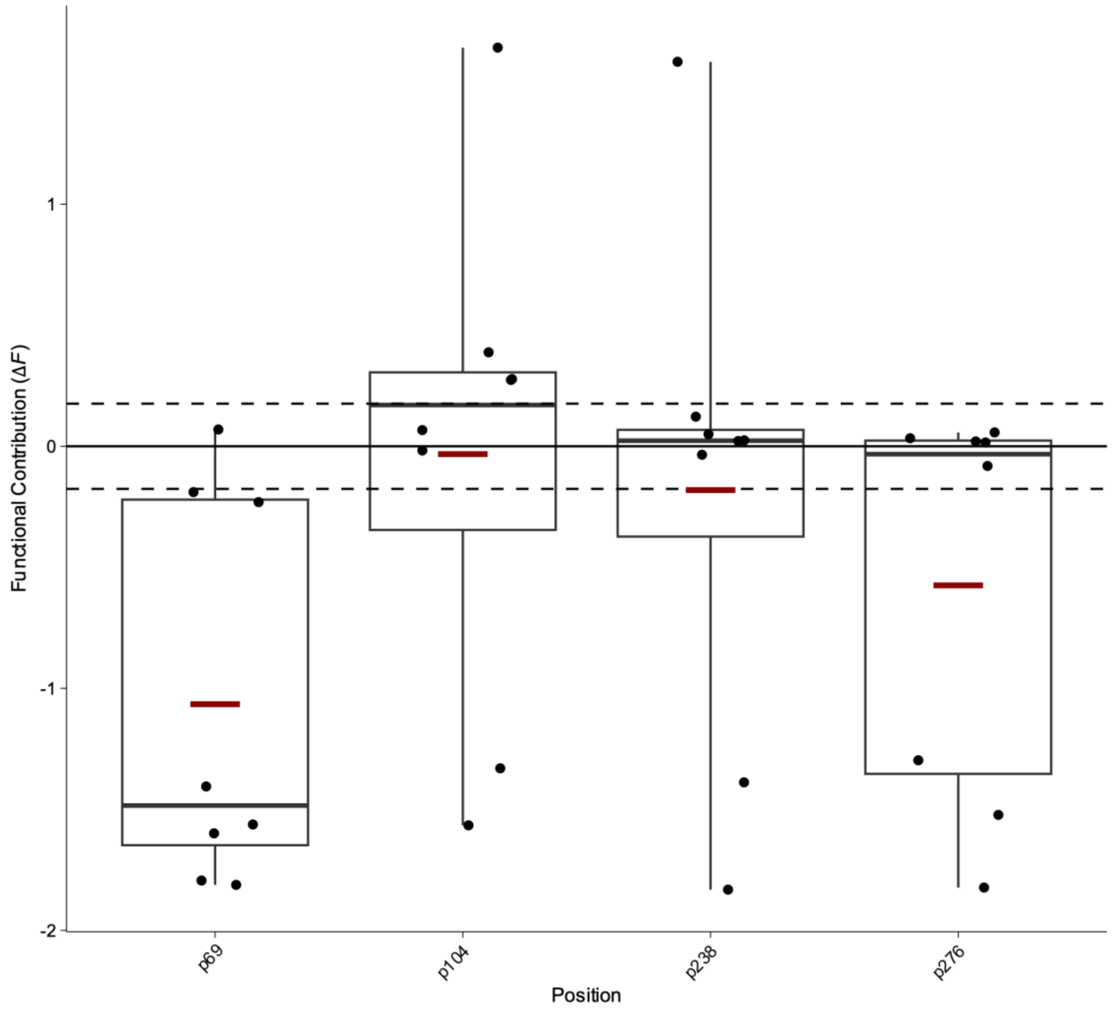

Box Plot 61 | Functional contribution of positional mutations in of beta lactamase TEM in CPR antibiotic Mira *et al.*

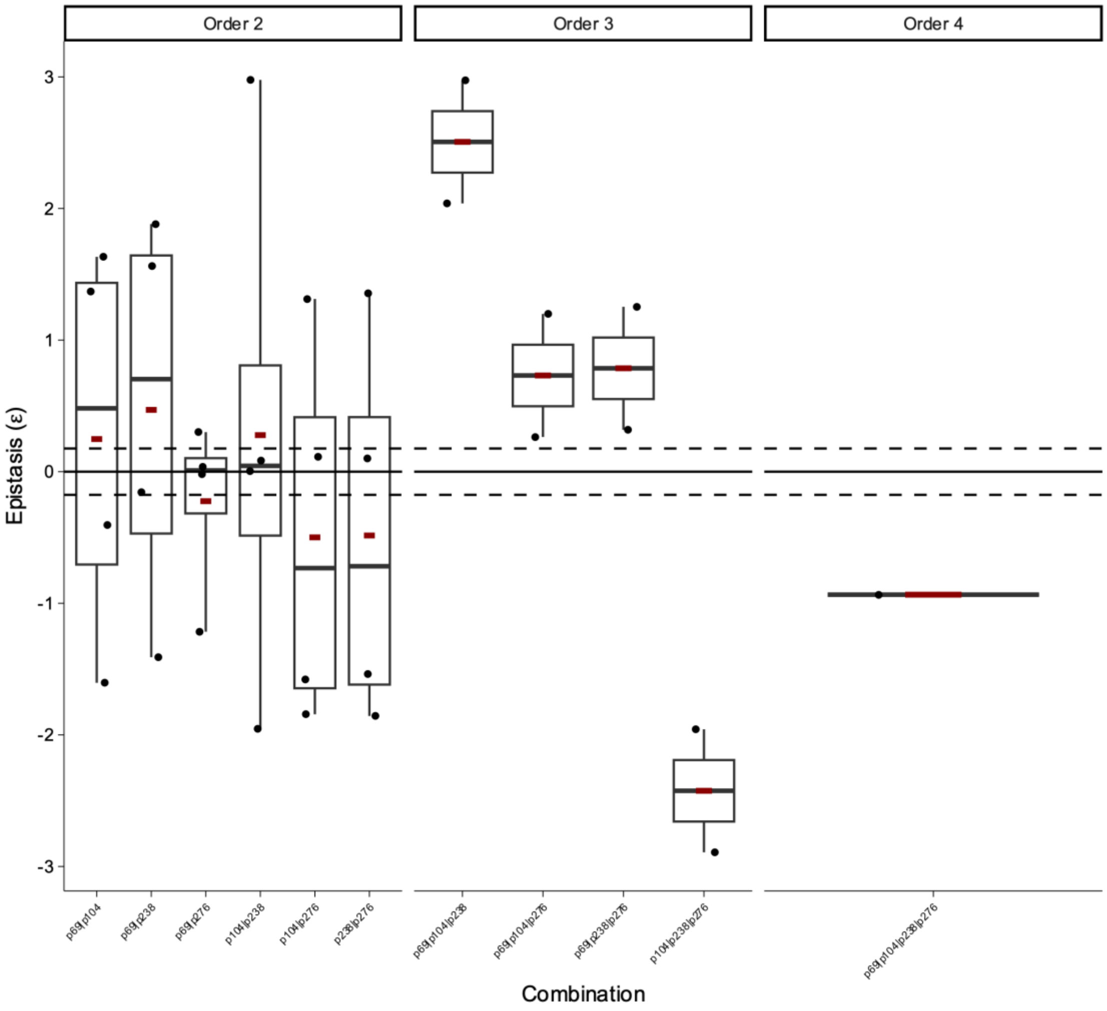

Box Plot 62 | Epistasis of combinatorial mutations in beta lactamase TEM in CPR antibiotic Mira *et al.*

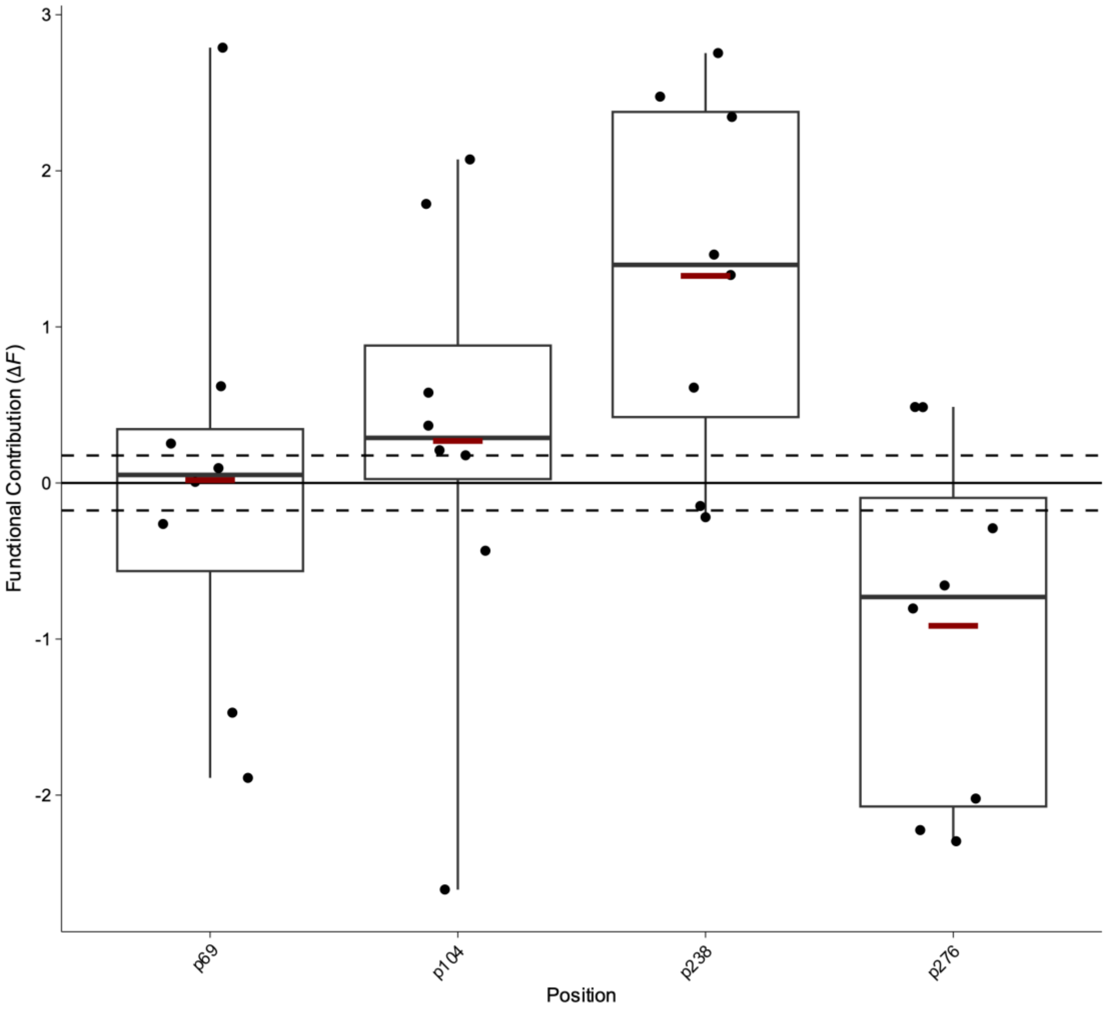

Box Plot 63 | Functional contribution of positional mutations in of beta lactamase TEM in CRO antibiotic Mira *et al.*

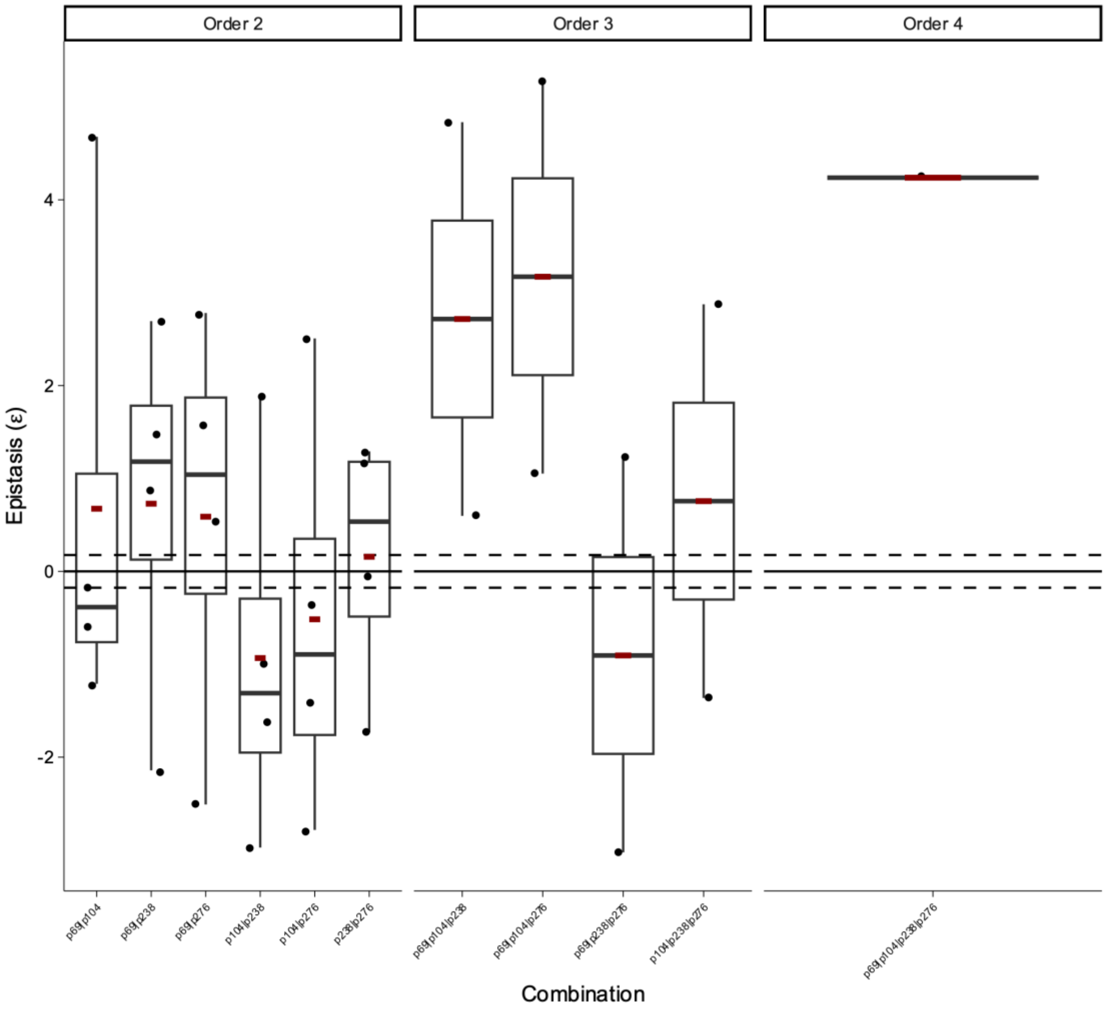

Box Plot 64 | Epistasis of combinatorial mutations in beta lactamase TEM in CRO antibiotic Mira *et al.*

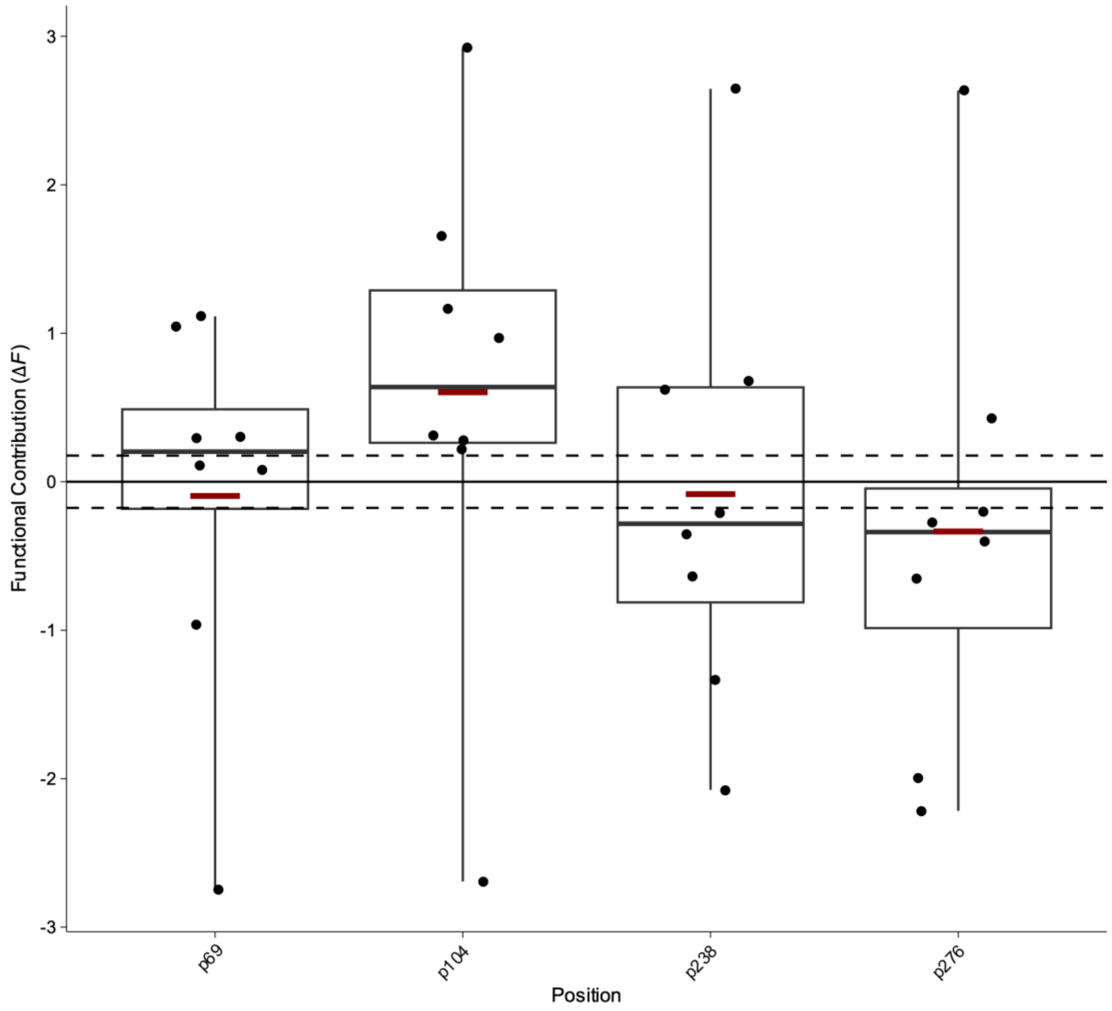

Box Plot 65 | Functional contribution of positional mutations in of beta lactamase TEM in CTT antibiotic Mira *et al.*

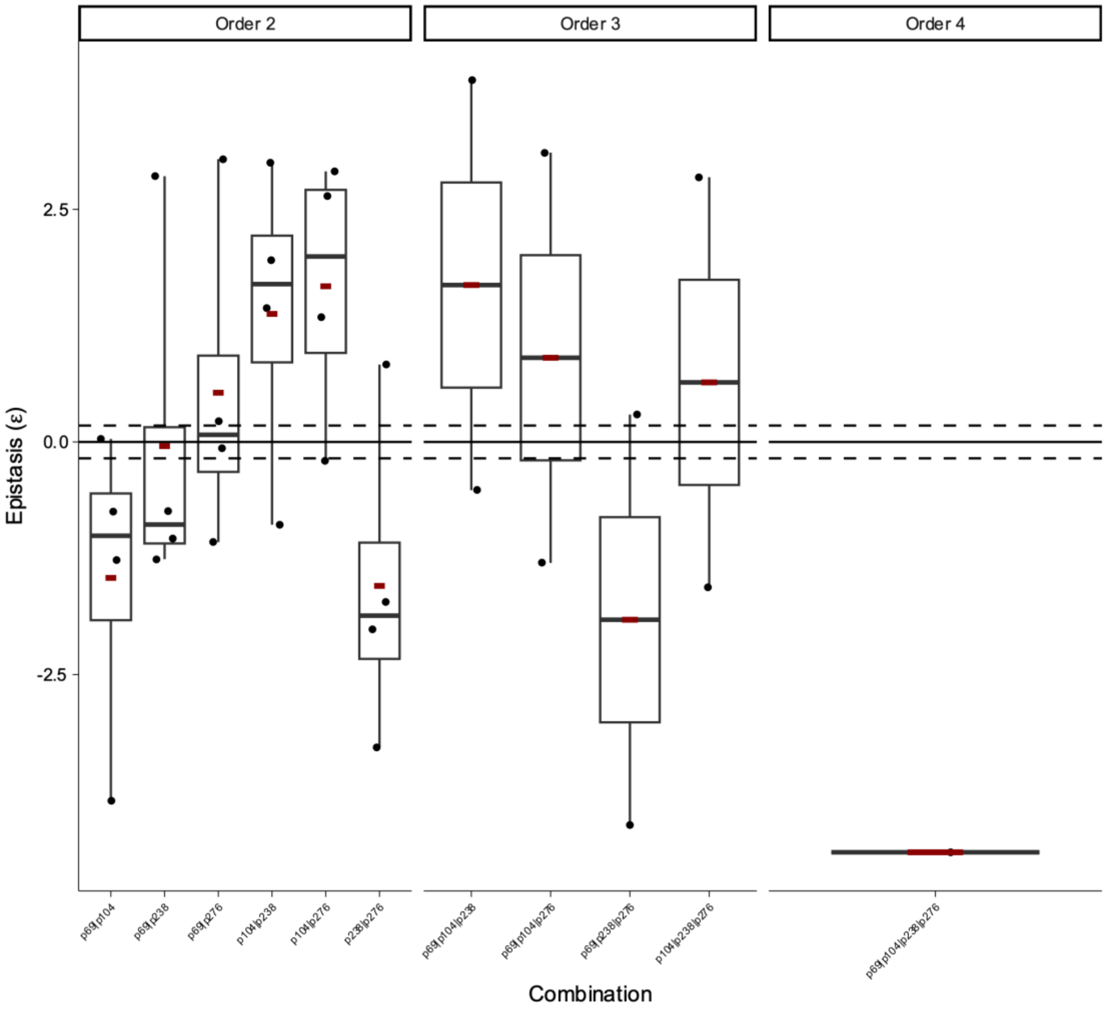

Box Plot 66 | Epistasis of combinatorial mutations in beta lactamase TEM in CTT antibiotic Mira *et al.*

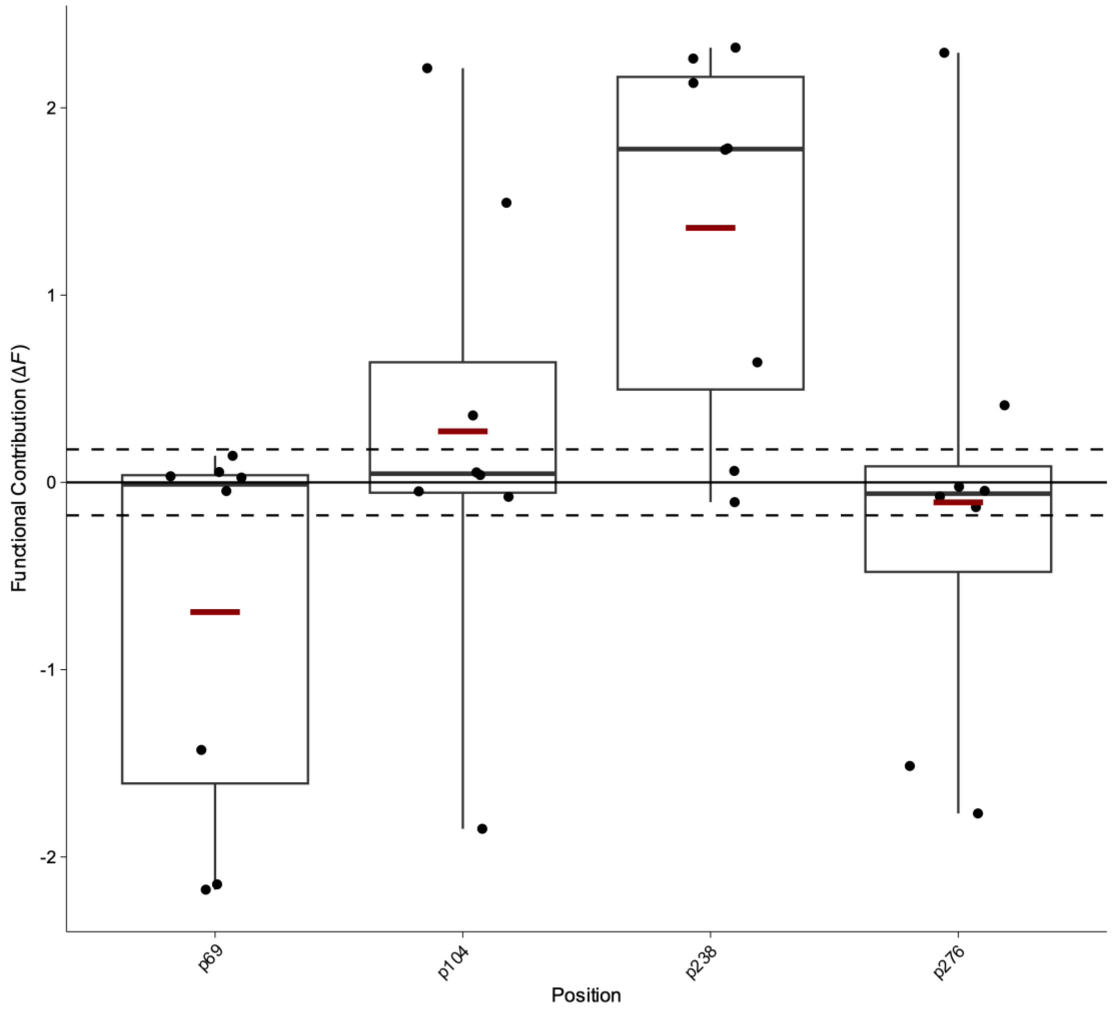

Box Plot 67 | Functional contribution of positional mutations in of beta lactamase TEM in CTX antibiotic Mira *et al.*

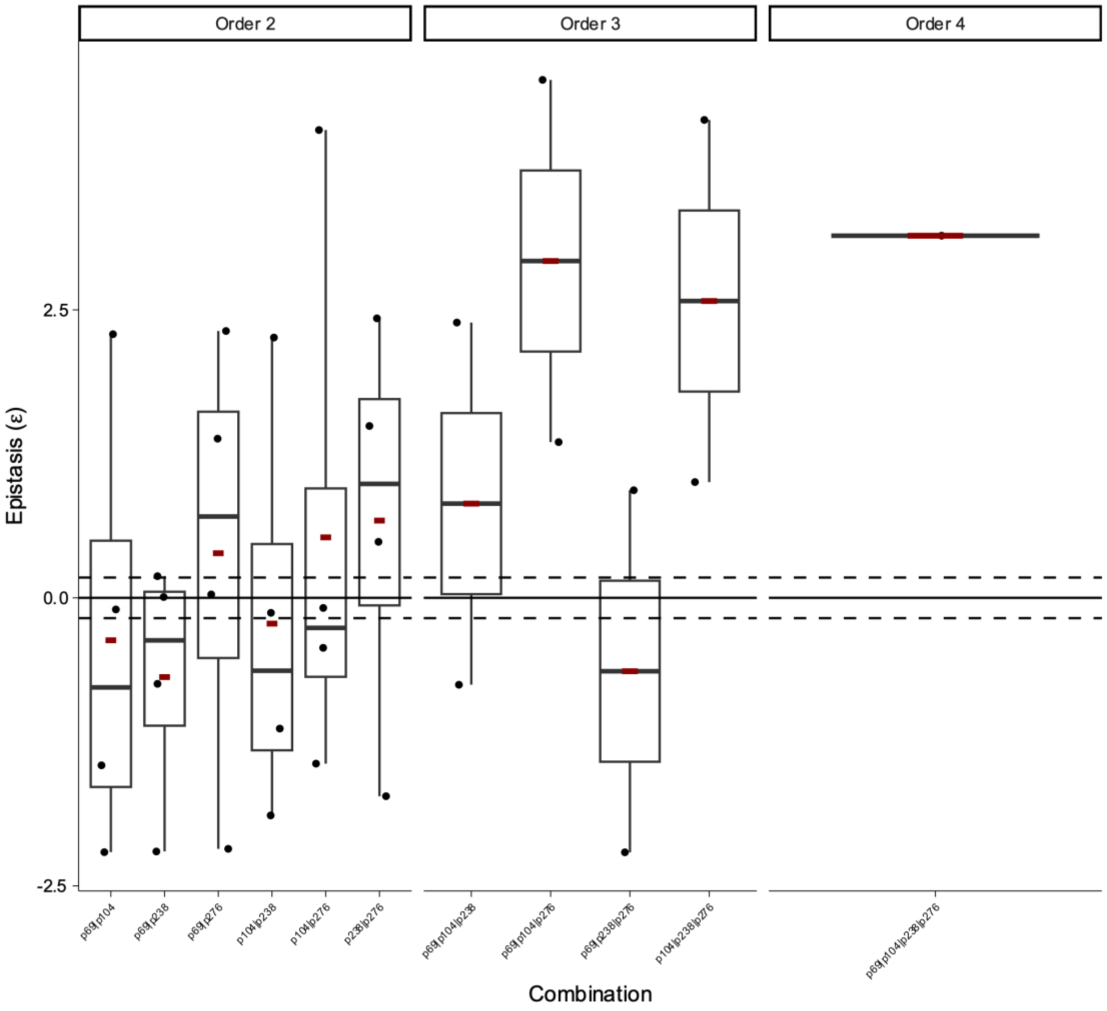

Box Plot 68 | Epistasis of combinatorial mutations in beta lactamase TEM in CTX antibiotic Mira *et al.*

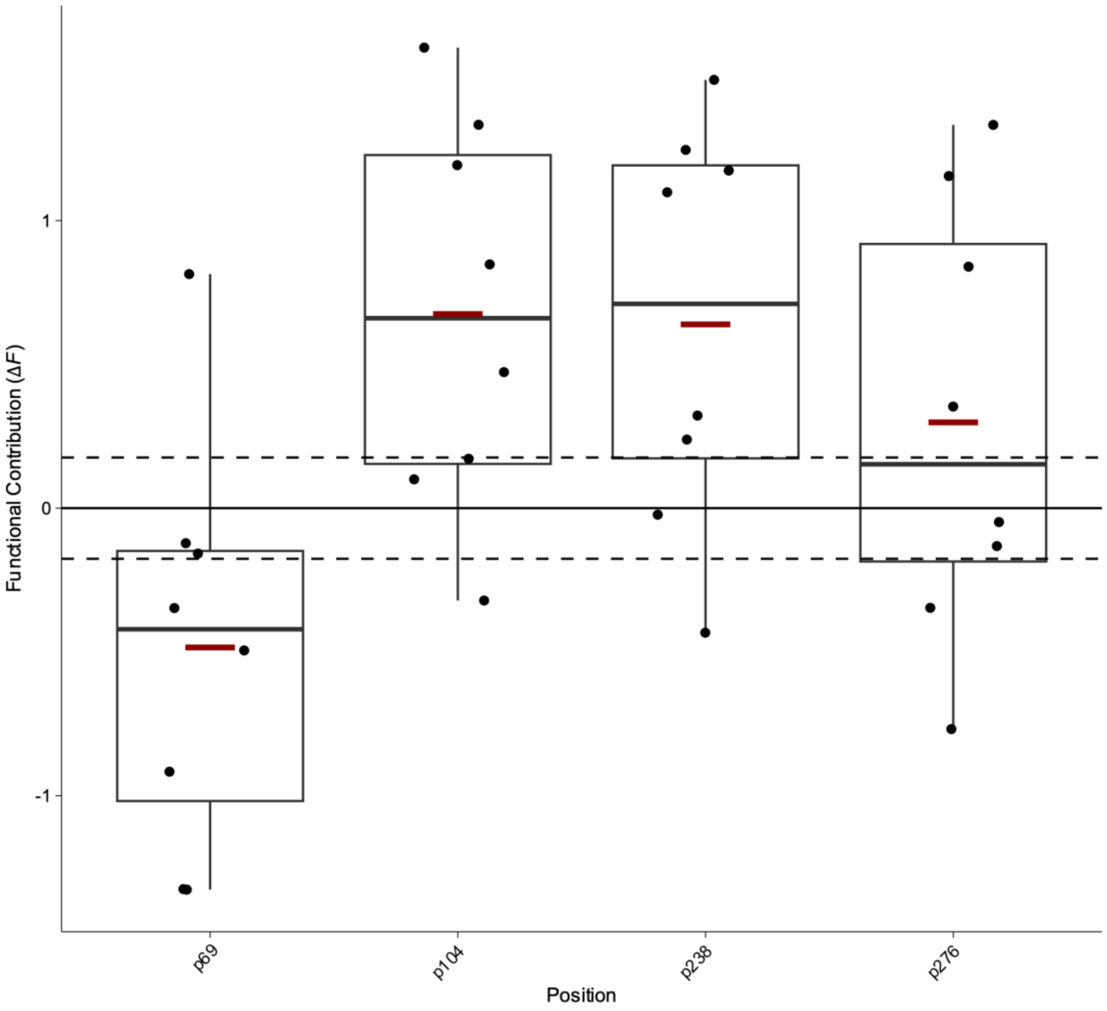

Box Plot 69 | Functional contribution of positional mutations in of beta lactamase TEM in CXM antibiotic Mira *et al.*

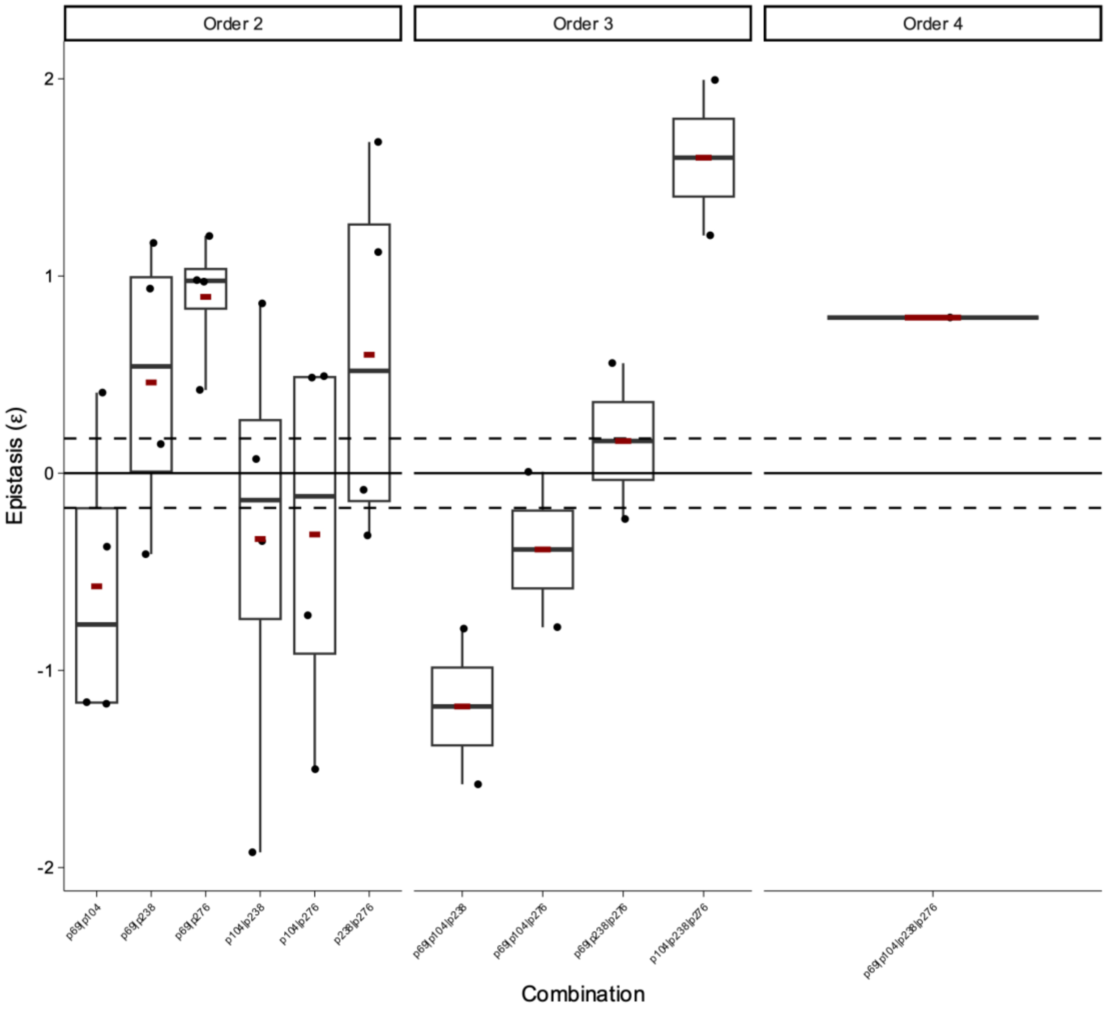

Box Plot 70 | Epistasis of combinatorial mutations in beta lactamase TEM in CXM antibiotic Mira *et al.*

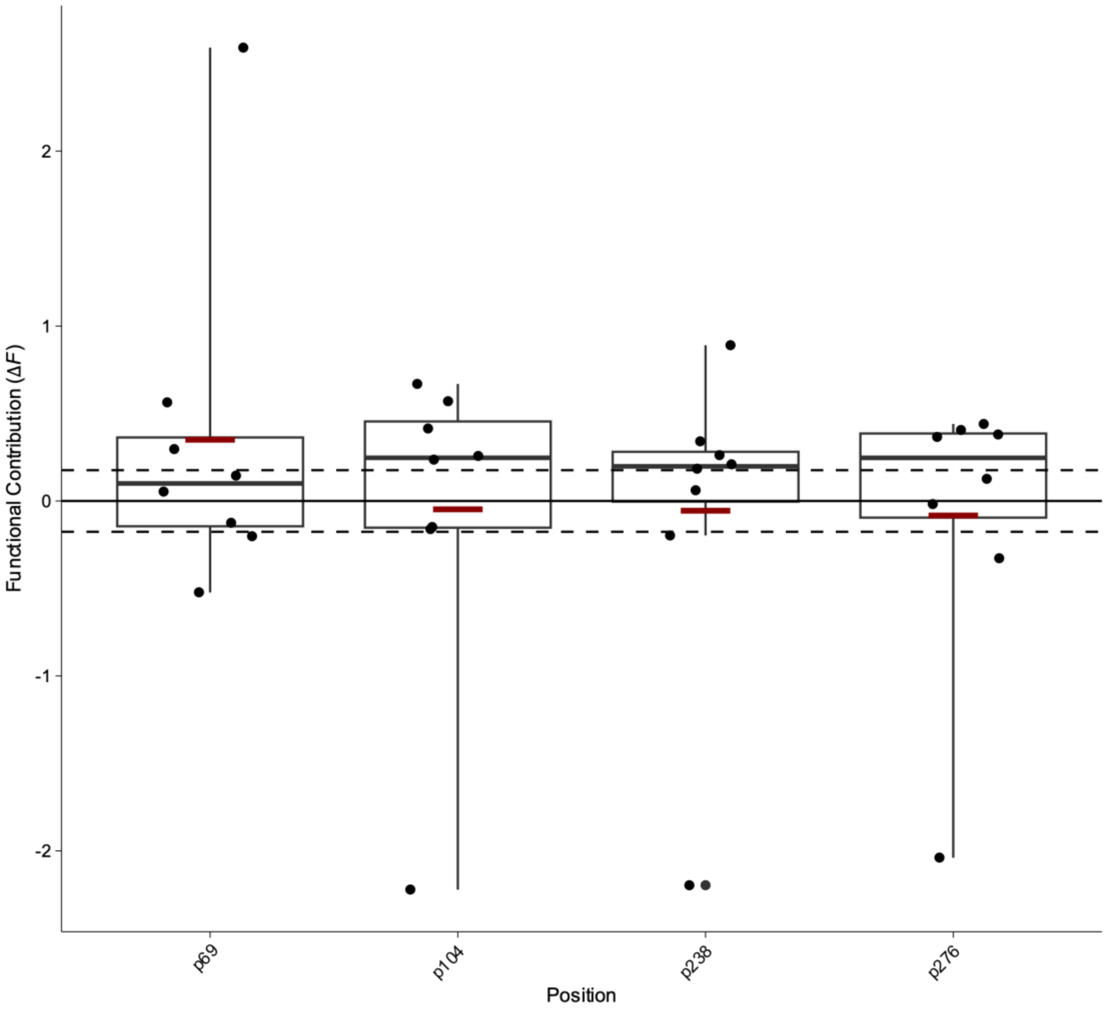

Box Plot 71 | Functional contribution of positional mutations in of beta lactamase TEM in FEP antibiotic Mira *et al.*

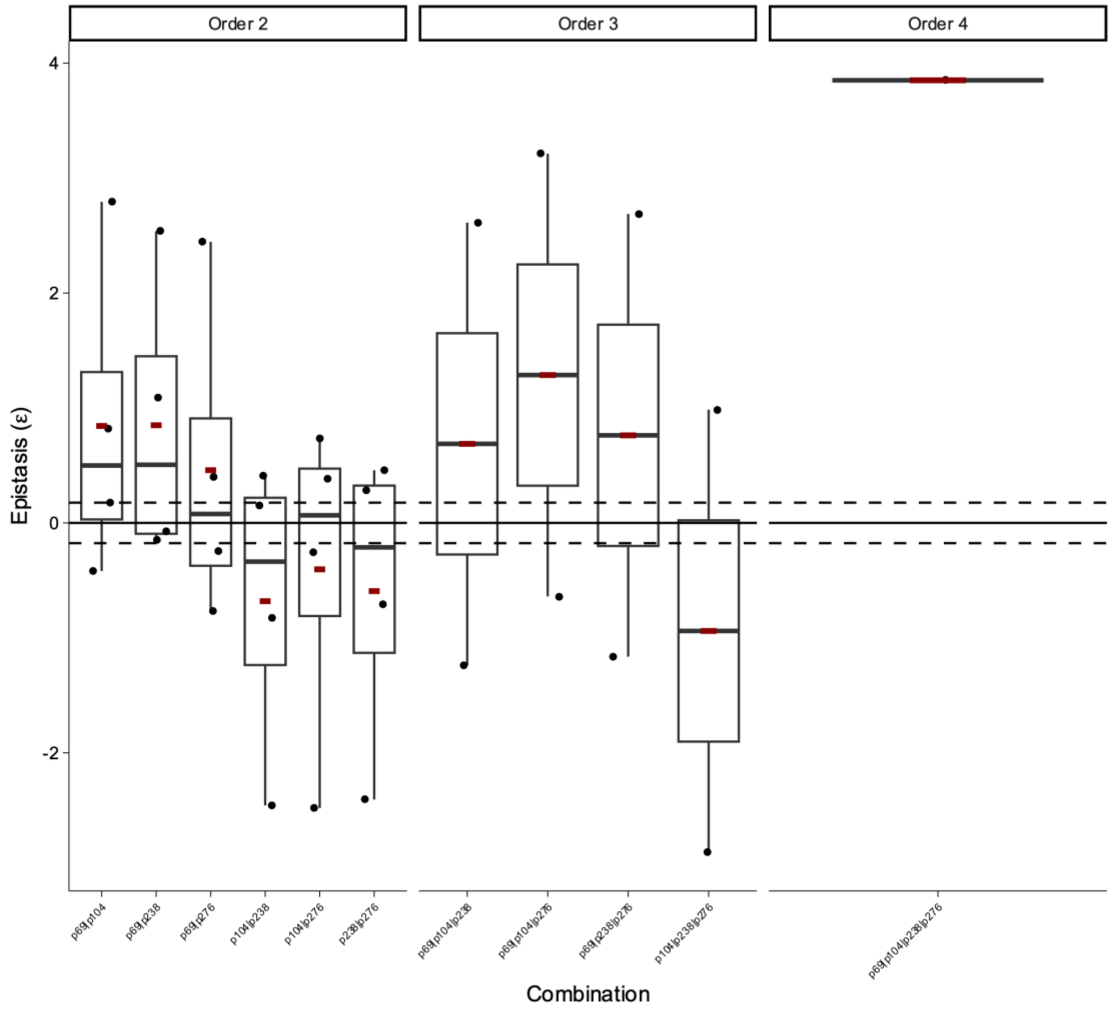

Box Plot 72 | Epistasis of combinatorial mutations in beta lactamase TEM in FEP antibiotic Mira *et al.*

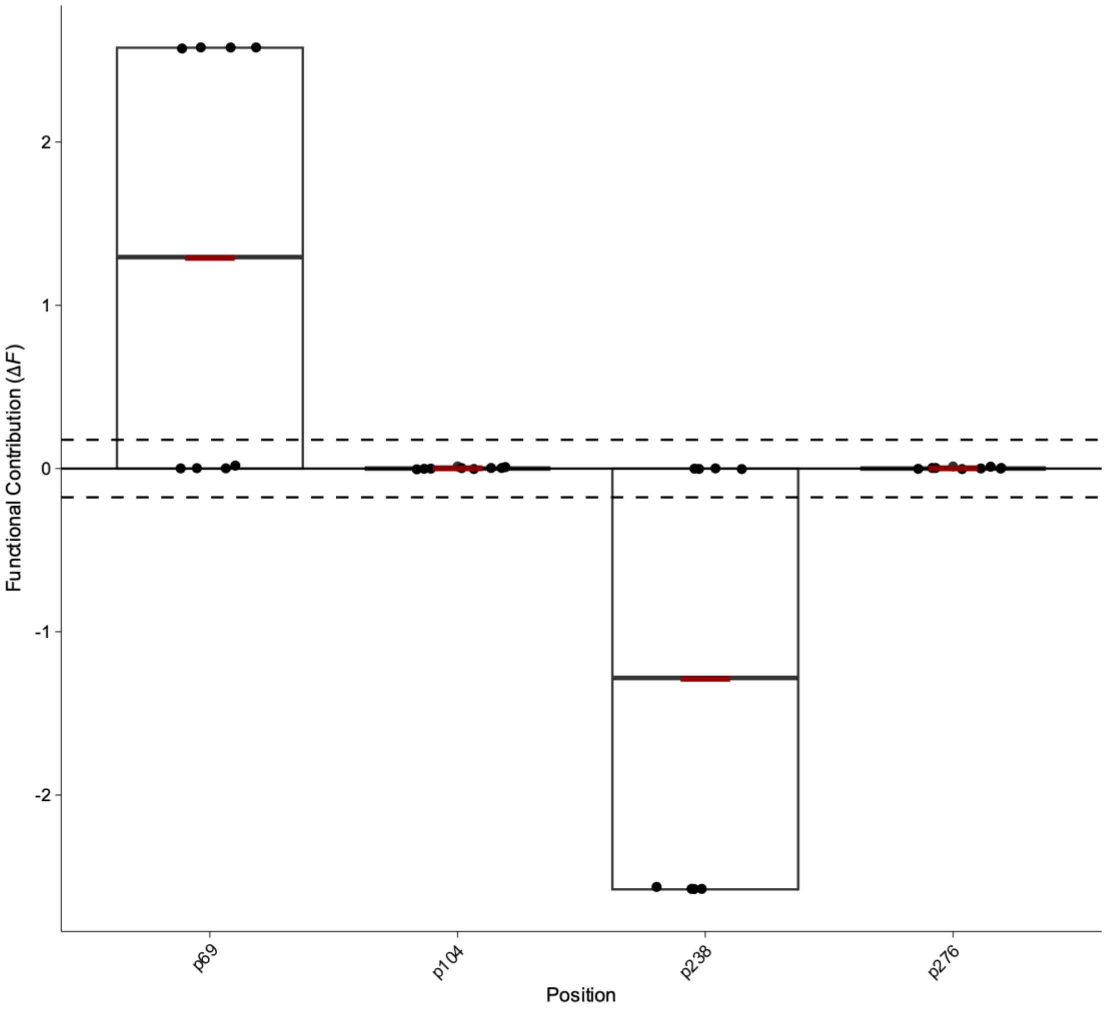

Box Plot 73 | Functional contribution of positional mutations in of beta lactamase TEM in SAM antibiotic Mira *et al.*

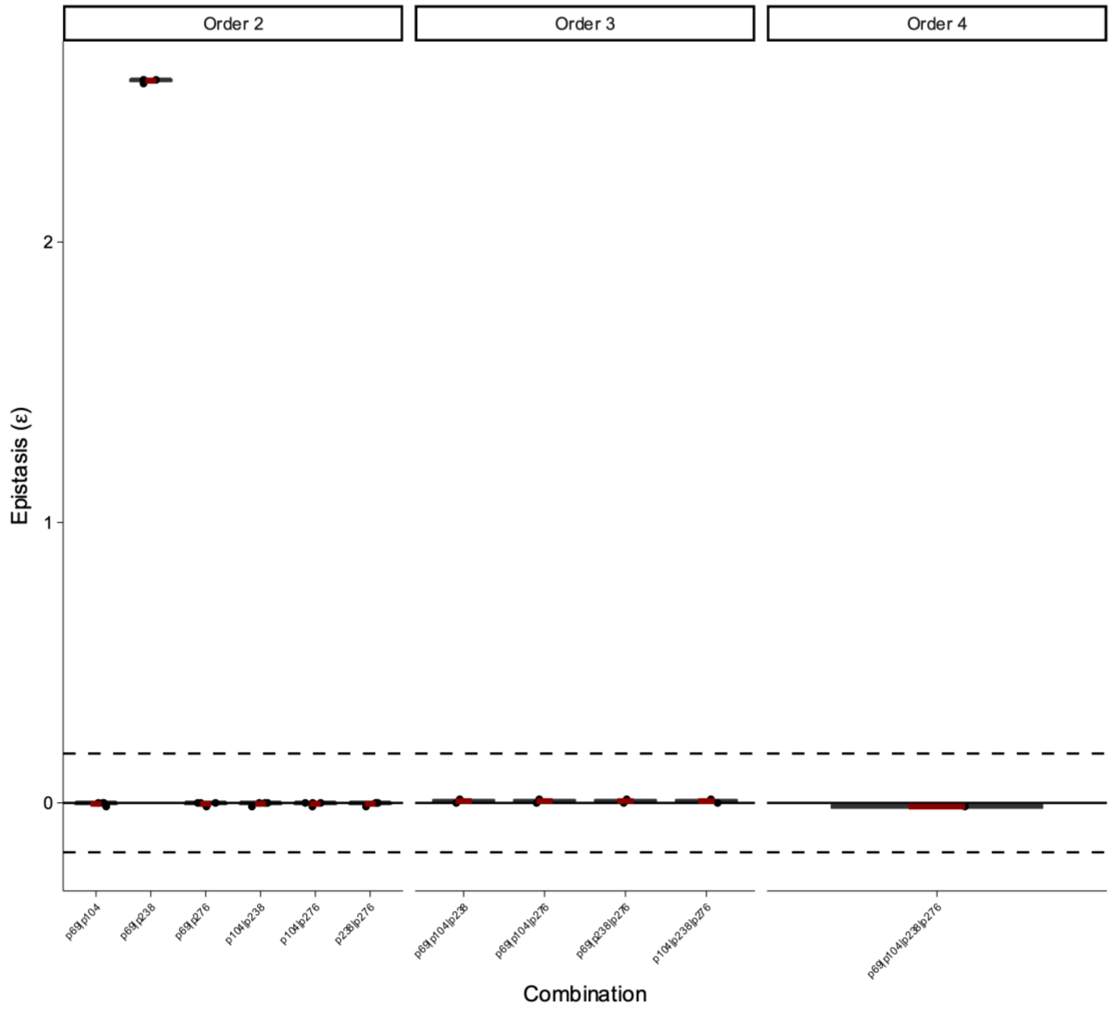

Box Plot 74 | Epistasis of combinatorial mutations in beta lactamase TEM in SAM antibiotic Mira *et al.*

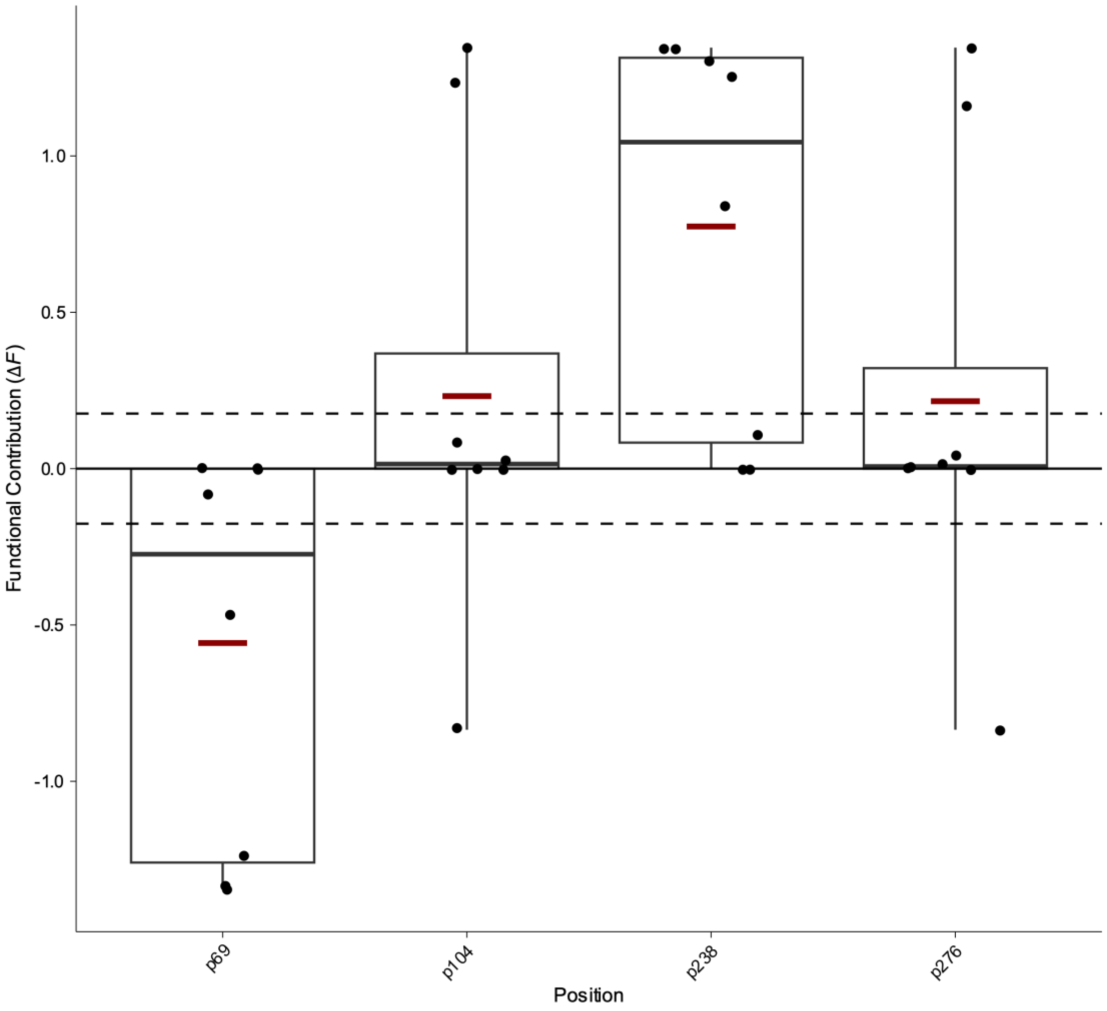

Box Plot 75 | Functional contribution of positional mutations in beta lactamase TEM in ZOX antibiotic Mira *et al.*

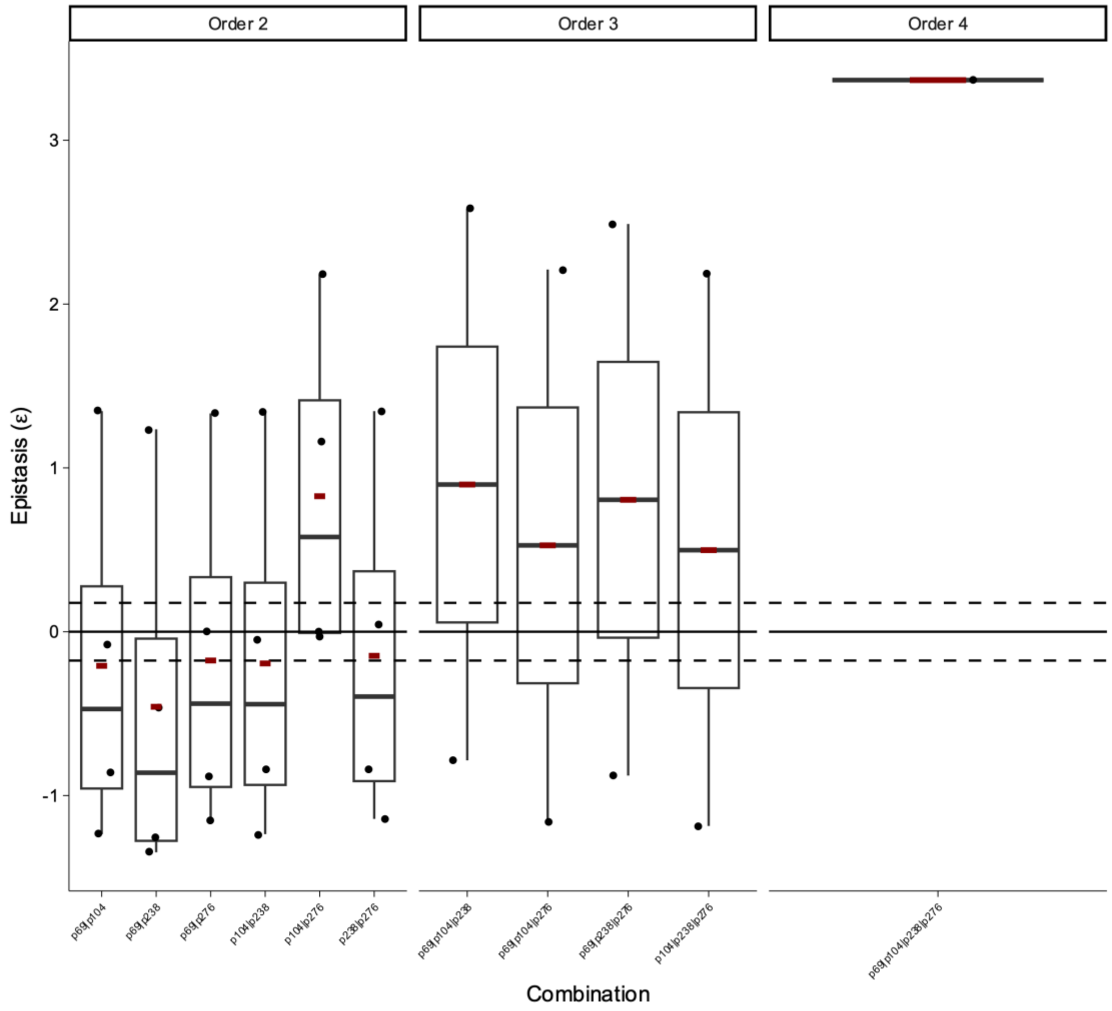

Box Plot 76 | Epistasis of combinatorial mutations in beta lactamase TEM in ZOX antibiotic Mira *et al.*

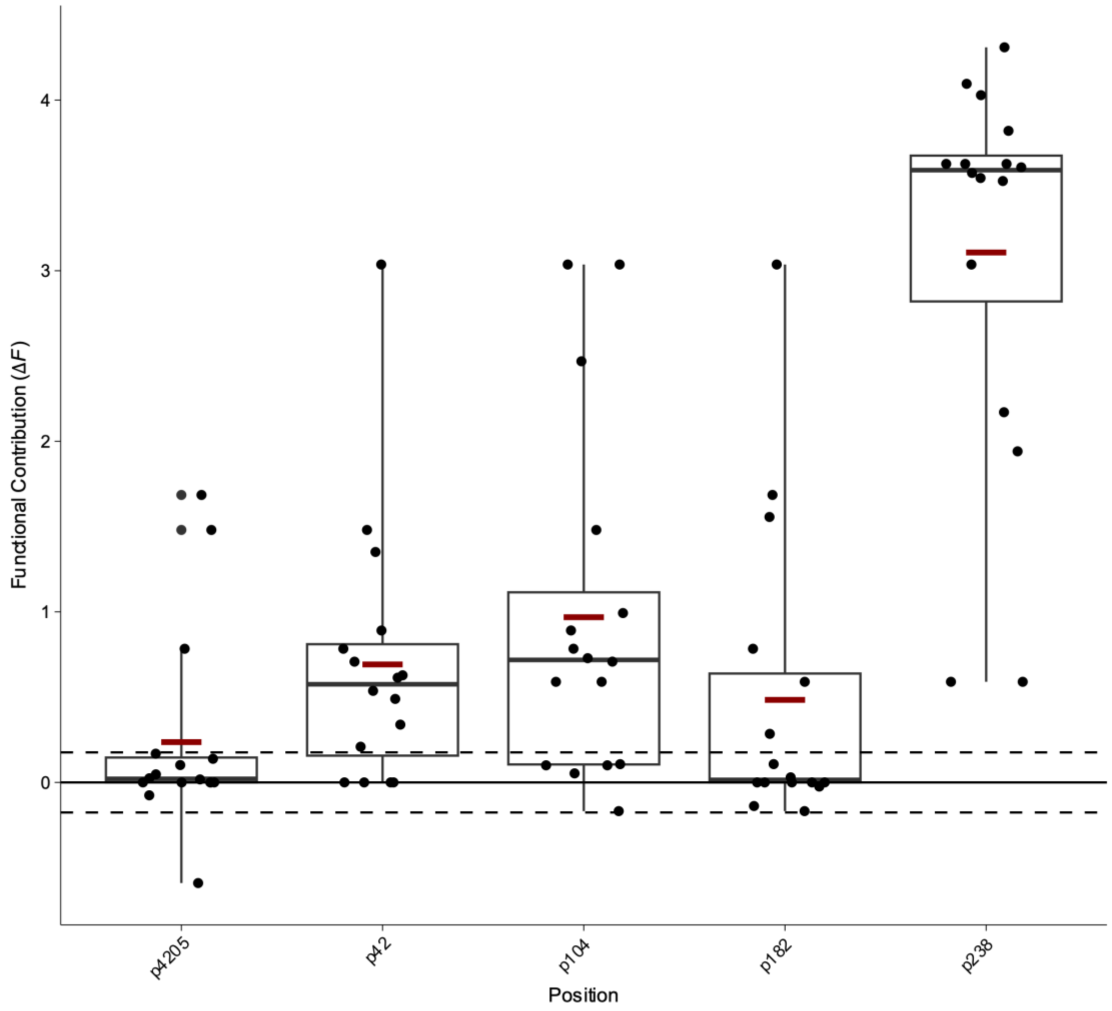

Box Plot 77 | Functional contribution of positional mutations in beta lactamase TEM from Weinreich *et al.*

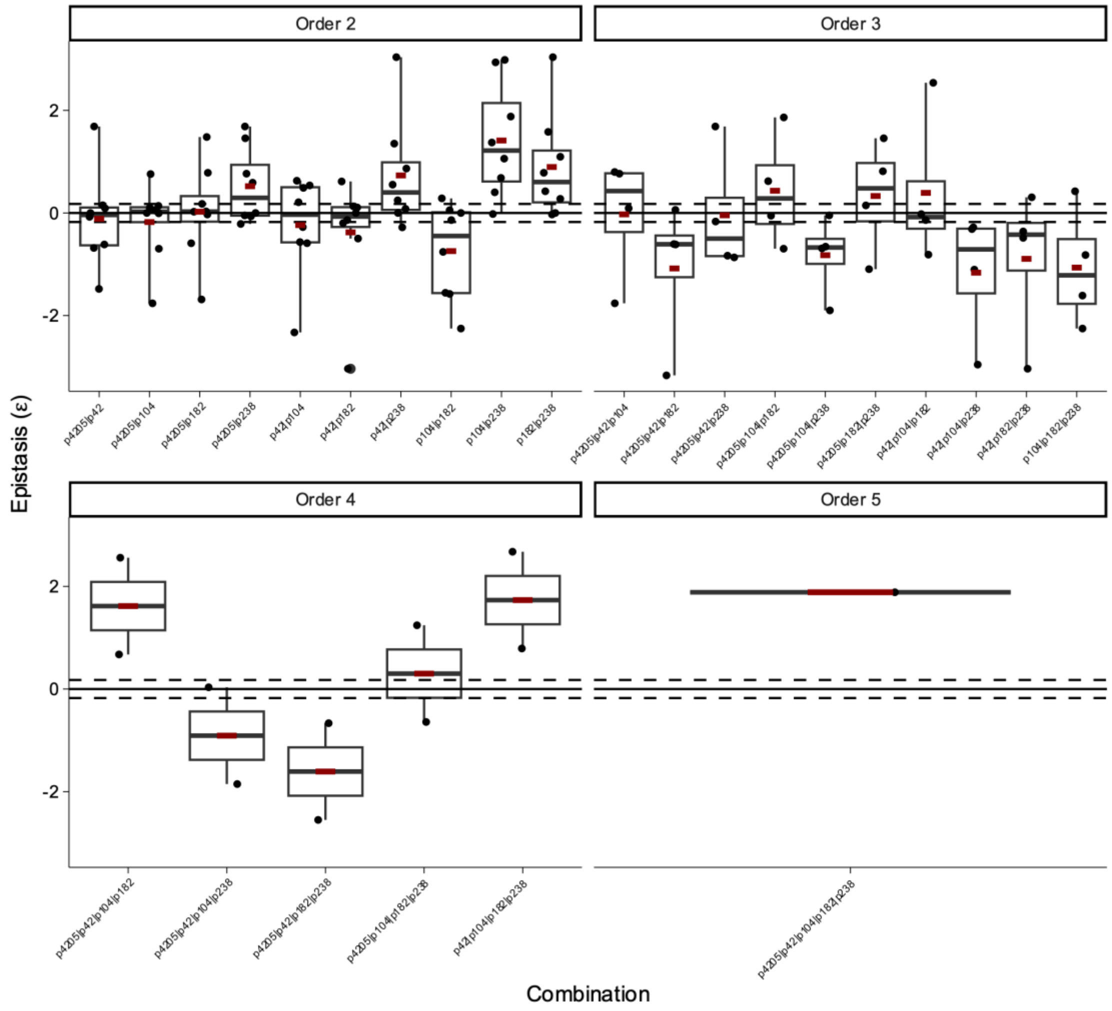

Box Plot 78 | Epistasis of combinatorial mutations in beta lactamase TEM from Weinreich *et al.*

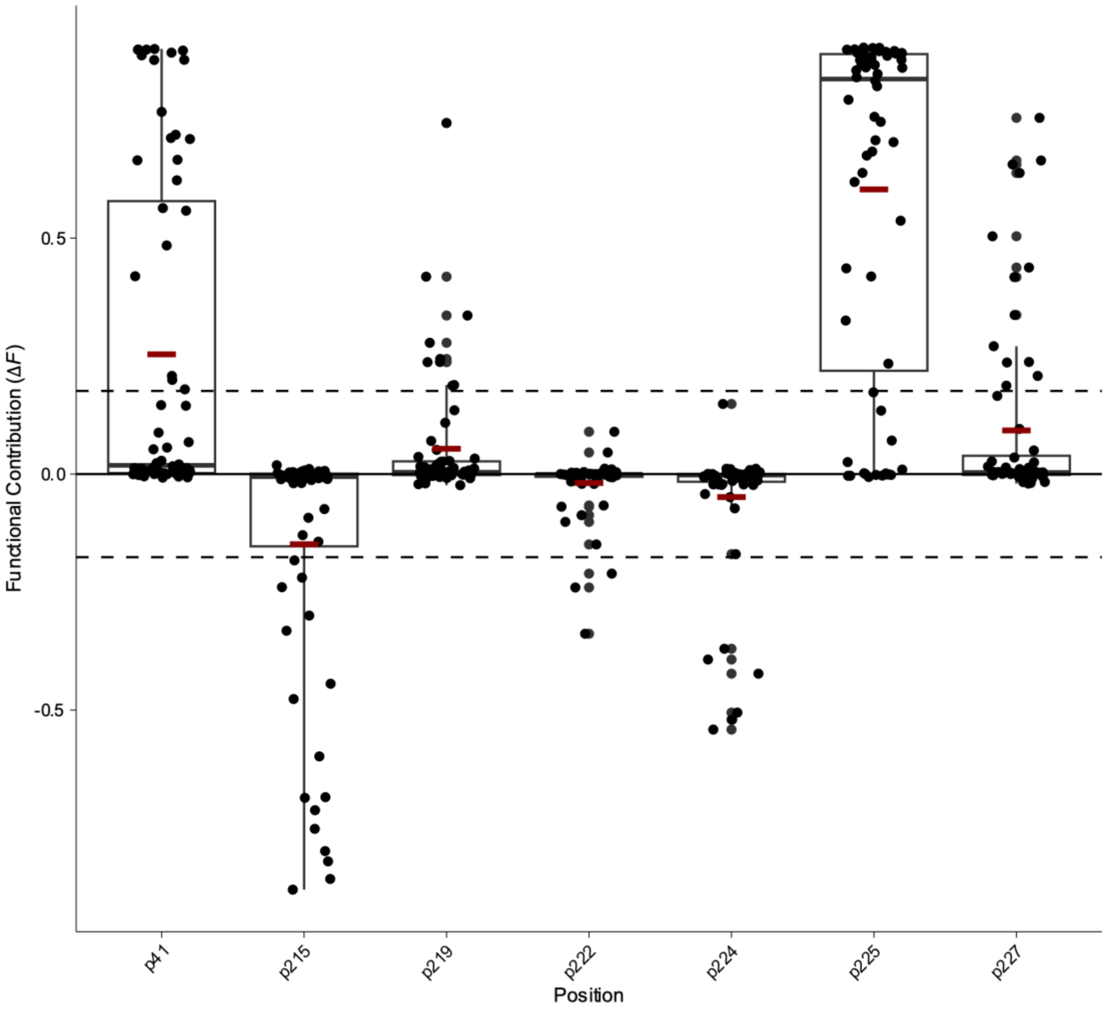

Box Plot 79 | Functional contribution of positional mutations in nitroreductase NfsA in the 20_39 trajectory from Hall *et al.*

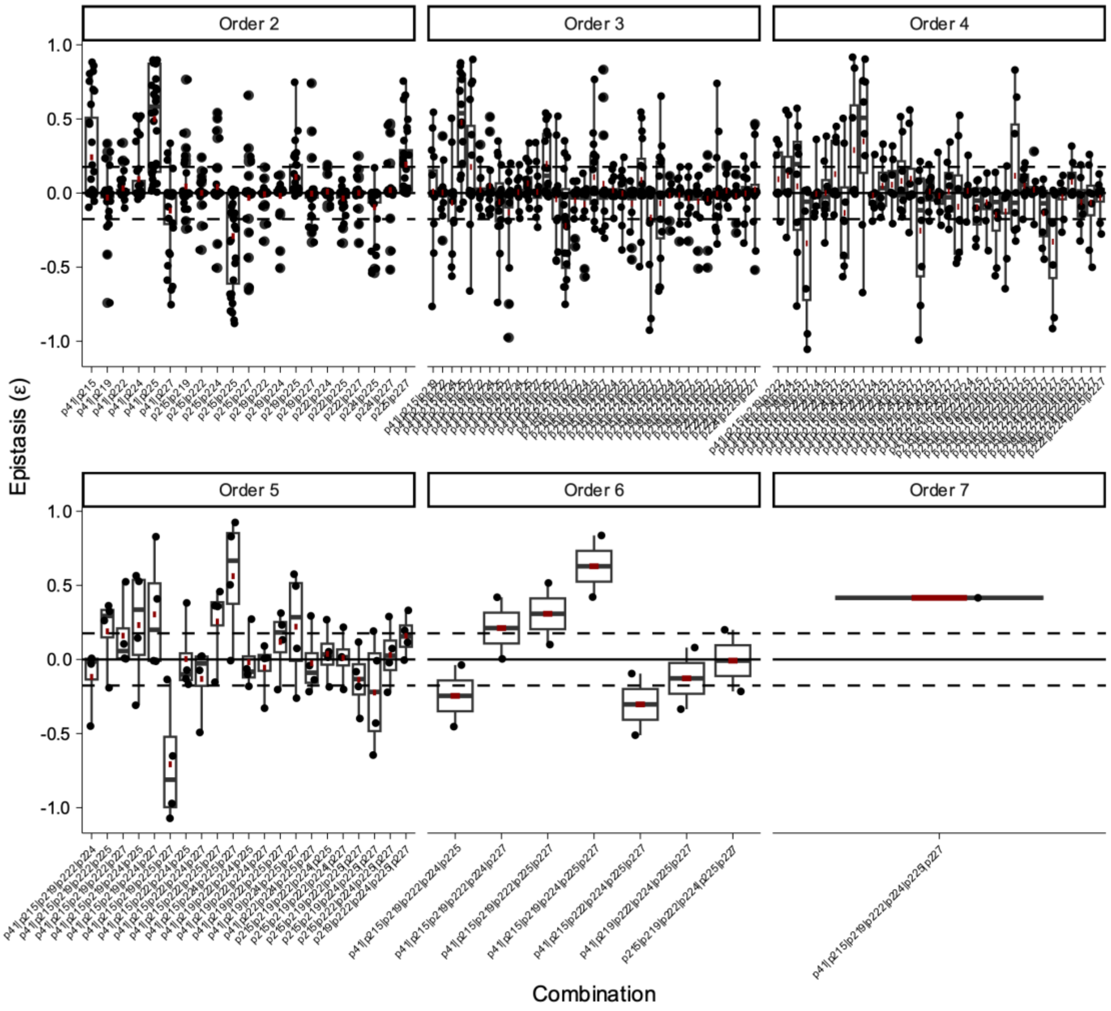

Box Plot 80 | Epistasis of combinatorial mutations in nitroreductase NfsA in the 20_39 trajectory from Hall *et al.*

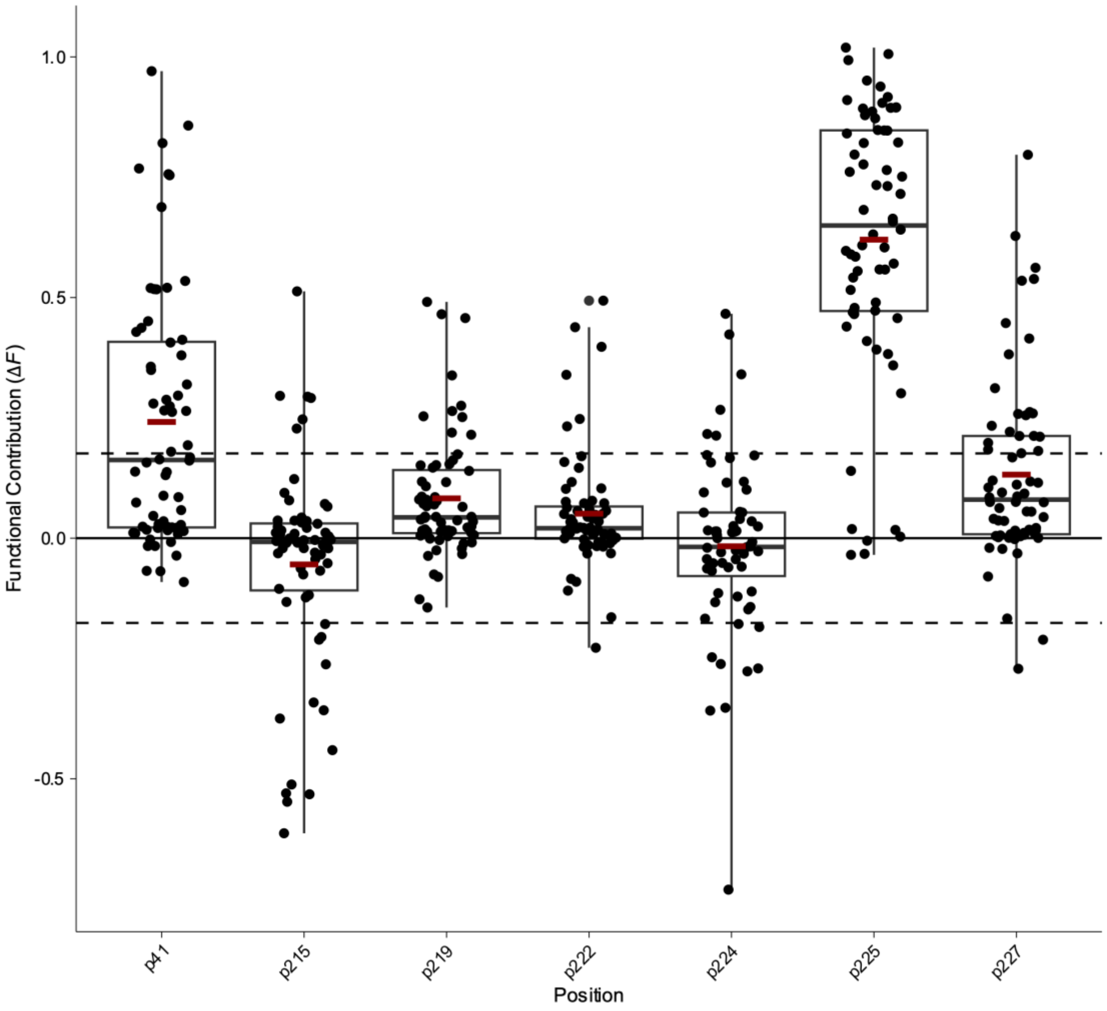

Box Plot 81 | Functional contribution of positional mutations in nitroreductase NfsA in the 36_37 trajectory from Hall *et al.*

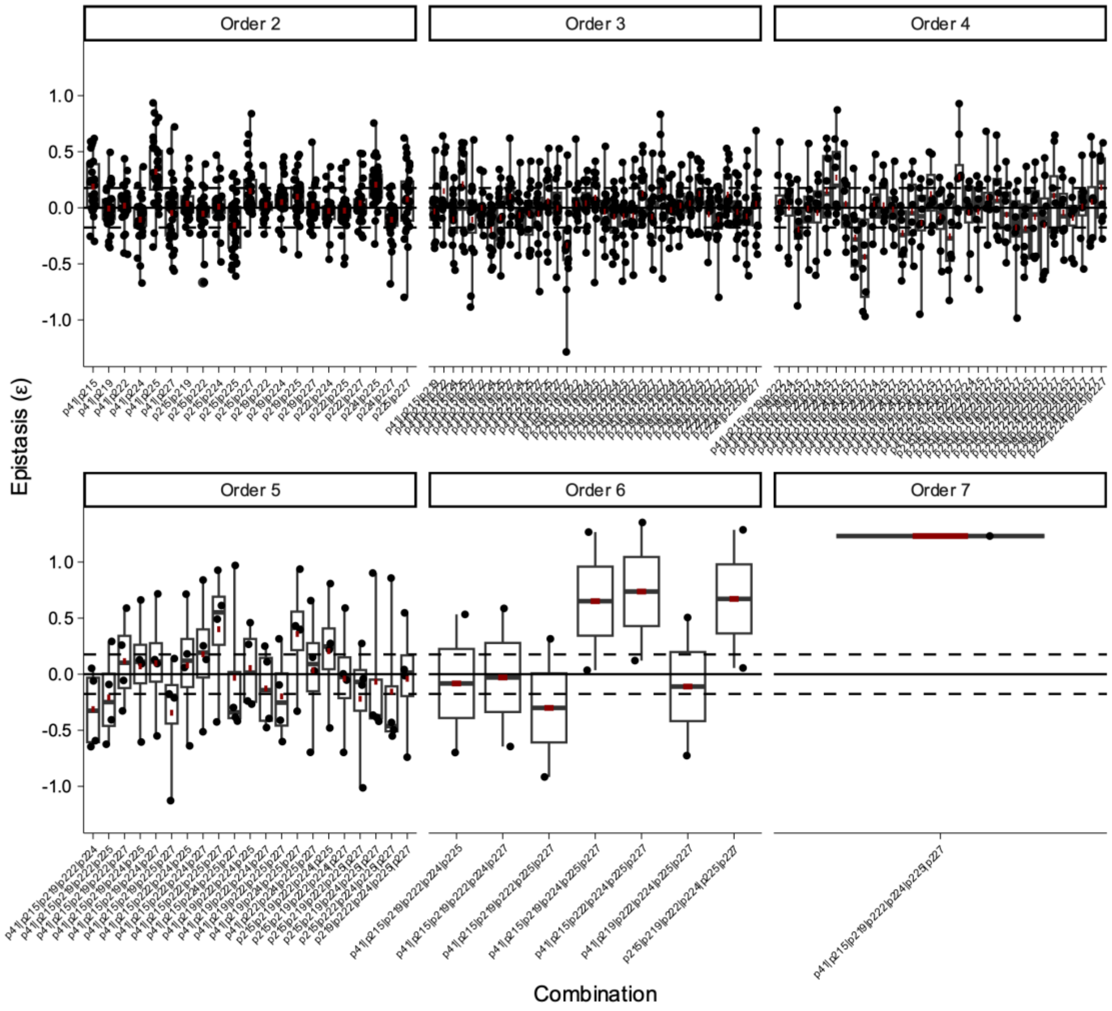

Box Plot 82 | Epistasis of combinatorial mutations in nitroreductase NfsA in the 36_37 trajectory from Hall *et al.*
