# Supplementary material for: Pervasive epistasis exposes intramolecular networks in adaptive enzyme evolution
Source: Nat Commun. 2023 Dec 21;14:8508. doi: 10.1038/s41467-023-44333-5 (PMC10739712; doi:10.1038/s41467-023-44333-5)
Supplement: Supplementary file 10 — Reporting Summary [file 41467_2023_44333_MOESM10_ESM.pdf]

nature portfolio

Corresponding author(s): Prof. Nobuhiko Tokuriki

Last updated by author(s): Nov 29, 2023

Reporting Summary

Nature Portfolio wishes to improve the reproducibility of the work that we publish. This form provides structure for consistency and transparency in reporting. For further information on Nature Portfolio policies, see our Editorial Policies and the Editorial Policy Checklist.

Statistics

For all statistical analyses, confirm that the following items are present in the figure legend, table legend, main text, or Methods section.

n/a	Confirmed	
☐	☒	The exact sample size (*n*) for each experimental group/condition, given as a discrete number and unit of measurement
☐	☒	A statement on whether measurements were taken from distinct samples or whether the same sample was measured repeatedly
☐	☒	The statistical test(s) used AND whether they are one- or two-sided<br>*Only common tests should be described solely by name; describe more complex techniques in the Methods section.*
☒	☐	A description of all covariates tested
☐	☒	A description of any assumptions or corrections, such as tests of normality and adjustment for multiple comparisons
☐	☒	A full description of the statistical parameters including central tendency (e.g. means) or other basic estimates (e.g. regression coefficient) AND variation (e.g. standard deviation) or associated estimates of uncertainty (e.g. confidence intervals)
☐	☒	For null hypothesis testing, the test statistic (e.g. *F*, *t*, *r*) with confidence intervals, effect sizes, degrees of freedom and *P* value noted<br>*Give P values as exact values whenever suitable.*
☒	☐	For Bayesian analysis, information on the choice of priors and Markov chain Monte Carlo settings
☒	☐	For hierarchical and complex designs, identification of the appropriate level for tests and full reporting of outcomes
☒	☐	Estimates of effect sizes (e.g. Cohen's *d*, Pearson's *r*), indicating how they were calculated

Our web collection on statistics for biologists contains articles on many of the points above.

Software and code

Policy information about availability of computer code

Data collection	No software or code was used in the collection of data.
Data analysis	Code for individual combinatorial landscape analysis and the global statistical analysis are publicly available via GitHub at https://doi.org/10.5281/zenodo.10202238. The scripts utilize the R language version 4.1.2 (https://www.R-project.org/), along with R packages outlined on GitHub.

For manuscripts utilizing custom algorithms or software that are central to the research but not yet described in published literature, software must be made available to editors and reviewers. We strongly encourage code deposition in a community repository (e.g. GitHub). See the Nature Portfolio guidelines for submitting code & software for further information.

Data

Policy information about availability of data

All manuscripts must include a data availability statement. This statement should provide the following information, where applicable:

- Accession codes, unique identifiers, or web links for publicly available datasets
- A description of any restrictions on data availability
- For clinical datasets or third party data, please ensure that the statement adheres to our policy

All raw data can be found in the corresponding references outlined in Table 1. Data for all combinatorial landscapes are provided in log10-transformed wt-normalized format (Supplementary Data 2). Processed data for functional contributions (Supplementary Data 4) and epistasis (Supplementary Data 6) are also available. The structural data for PTE-R1, PTE-R2, PTE-R8, PTE-R18, MPH-Anc, and MPH* are available under PDB accession numbers 4XAF [https://doi.org/10.2210/

pdb4XAF/pdb], 4XD5 [https://doi.org/10.2210/pdb4XD5/pdb], 4XAY [https://doi.org/10.2210/pdb4XAY/pdb], 4E3T [https://doi.org/10.2210/pdb43ET/pdb], 6C2C [https://doi.org/10.2210/pdb6C2C/pdb], and 1P9E [https://doi.org/10.2210/pdb1P9E/pdb], respectively. These data are sufficient for the reproduction of all results presented in our work. Data presented in all figures can be found in the Source Data file.

Research involving human participants, their data, or biological material

Policy information about studies with human participants or human data. See also policy information about sex, gender (identity/presentation), and sexual orientation and race, ethnicity and racism.

Reporting on sex and gender	No data analysis nor discussion concerned sex or gender.
Reporting on race, ethnicity, or other socially relevant groupings	No data analysis nor discussion concerned race, ethnicity, or other socially relevant groupings.
Population characteristics	No data included human research participants.
Recruitment	No human participants were included.
Ethics oversight	No bioethical oversight was necessary in this study.

Note that full information on the approval of the study protocol must also be provided in the manuscript.

Field-specific reporting

Please select the one below that is the best fit for your research. If you are not sure, read the appropriate sections before making your selection.

☐ Life sciences ☐ Behavioural & social sciences ☒ Ecological, evolutionary & environmental sciences

For a reference copy of the document with all sections, see nature.com/documents/nr-reporting-summary-flat.pdf

Ecological, evolutionary & environmental sciences study design

All studies must disclose on these points even when the disclosure is negative.

Study description	The study explores the effect of enzyme mutations - both single mutational effects and mutational interactions (epistasis) - on measured functions. Data were analyzed using descriptive statistical parameters (mainly standard deviation and threshold analysis) and two predictive models.
Research sample	Data were collected from ten studies obtained via a manually curated literature search. The search aimed to collect all fully annotated combinatorial landscapes of adaptive enzyme evolution present in literature, thus no larger sample could be acquired. The studies spanned 41 protein fitness landscapes in total. All datasets consisted of landscapes that fully characterised a combinatorial subset of 'n' single adaptive mutations, where $3 < n < 8$.
Sampling strategy	No statistical methods were used for sampling. Studies containing combinatorial landscape data were filtered based on: adaptive evolutionary trajectories of enzymes, probing four or more mutations, functionally characterizing all combinations of these mutations, and measuring a continuous variable for functional characterization. To our knowledge, we have collected all possible studies available in the literature that satisfy these requirements.
Data collection	Data were collected from main texts or supplementary data of the studies outlined in Table 1. Data was manually inputed into separate spreadsheets by the first author of this study.
Timing and spatial scale	Data were collected from November 2020 until September 2022 with no specific frequency and/or periodicity of sampling, as all datasets were publically available at this time. To our knowledge, no new adaptive enzyme combinatorial landscapes have been published in peer reivewed articles between the 1st of November 2020 and 29th of November 2023. Spacial scale is not relevant to the data collection in this study.
Data exclusions	Due to a small subset of studies that probed five or more mutations, statistical overviews of epistasis omitted the analysis of combinations at the fifth and higher orders due to the insufficient sample size. From the original 45 landscapes, we excluded 4 datasets obtained from the TEM study by Mira et al. (2015) due to the resulting binary distribution of fold-change values upon applying a non-linear transformation to the data.
Reproducibility	The majority of the data was acquired from external sources, and replicates were only used when provided. For the PTE combinatorial landscape generated in this study, data used were sourced from a minimum of two technical replicates with up to three biological replicates for each genotype. In all cases where available, replicates were succesful.
Randomization	Randomization was not relevant to our study – all datasets were pooled together for global analysis. Later analyses extracted specific datasets for the purpose of unraveling specific molecular mechanisms within an enzyme. Cross-landscape correlation was controlled by analyzing a parallel 'reduced' dataset consisting of data with entirely unique genotype-phenotype measurements.
Blinding	Blinding was not relevant to data acquisition as the dataset represents an exhaustive list of studies that satisfied the criteria. Blinding was achieved during data analysis by pooling all datasets and analyzing mutational and epistasic effects collectively.

Did the study involve field work? ☐ Yes ☒ No

Reporting for specific materials, systems and methods

We require information from authors about some types of materials, experimental systems and methods used in many studies. Here, indicate whether each material, system or method listed is relevant to your study. If you are not sure if a list item applies to your research, read the appropriate section before selecting a response.

Materials & experimental systems

n/a	Involved in the study
☒	☐ Antibodies
☒	☐ Eukaryotic cell lines
☒	☐ Palaeontology and archaeology
☒	☐ Animals and other organisms
☒	☐ Clinical data
☒	☐ Dual use research of concern
☒	☐ Plants

Methods

n/a	Involved in the study
☒	☐ ChIP-seq
☒	☐ Flow cytometry
☒	☐ MRI-based neuroimaging

Plants

Seed stocks

Report on the source of all seed stocks or other plant material used. If applicable, state the seed stock centre and catalogue number. If plant specimens were collected from the field, describe the collection location, date and sampling procedures.

Novel plant genotypes

Describe the methods by which all novel plant genotypes were produced. This includes those generated by transgenic approaches, gene editing, chemical/radiation-based mutagenesis and hybridization. For transgenic lines, describe the transformation method, the number of independent lines analyzed and the generation upon which experiments were performed. For gene-edited lines, describe the editor used, the endogenous sequence targeted for editing, the targeting guide RNA sequence (if applicable) and how the editor was applied.

Authentication

Describe any authentication procedures for each seed stock used or novel genotype generated. Describe any experiments used to assess the effect of a mutation and, where applicable, how potential secondary effects (e.g. second site T-DNA insertions, mosiacism, off-target gene editing) were examined.
